# Conformational constraints in [Ni(P^R^_2_N^R′^_2_)_2_] complexes for tuning H_2_ production and oxidation: a DFT-based ligand design study

**DOI:** 10.1039/d5ra05545h

**Published:** 2025-09-29

**Authors:** Sarinya Hadsadee, Manussada Ratanasak, Phiphob Naweephattana, Sumiko Morita, Ray Miyazaki, Kenji Iida, Akira Nakayama, Siriporn Jungsuttiwong, Jun-ya Hasegawa

**Affiliations:** a Institute for Catalysis, Hokkaido University N21 W10 Kita-ku Sapporo Hokkaido 001-0021 Japan hasegawa@cat.hokudai.ac.jp; b Department of Chemistry and Center of Excellence for Innovation in Chemistry, Ubon Ratchathani University Ubon Ratchathani 34190 Thailand

## Abstract

[Ni(P^R^_2_N^R′^_2_)_2_] complexes catalyze H_2_ production or oxidation depending on the nature of the two bidentate P_2_N_2_ ligands. Ligand design plays a crucial role in determining the reaction direction and catalytic properties. In this study, density functional theory (DFT) calculations were performed to analyze the structural and energetic properties of Ni complexes with (R, R′) = (H, H), (Me, Me), (Cy, Me), (^*t*^Bu, Me), (CF_3_, H), and (NH_2_, H). Based on the structural features, relative stabilities of the Ni^II^ and Ni^0^ complexes, and their energy profiles, these Ni complexes were classified into three groups, I–III. In group I, the reaction is slightly exothermic toward H_2_ production. In group II, the presence of ^*t*^Bu and CF_3_ groups introduces steric hindrance, forcing the Ni complexes into a tetrahedral conformation. This geometric constraint destabilizes the product state in the Ni^II^ oxidation state, shifting the thermodynamics toward H_2_ oxidation. Conversely, destabilizing the reactant state in the Ni^0^ oxidation state can be achieved using a tetradentate ligand in which the two bidentate P_2_N_2_ ligands are connected by trimethylene, –(CH_2_)_3_–, units at the P atoms. This ligand, classified as group III, maintains a square planar conformation, rendering H_2_ production highly exothermic. These findings align with experimental observations of similar complexes and underscore the importance of ligand geometry and substituent effects.

## Abstract 2

Ligand design for H_2_ oxidation/production activity of Ni complex *via* tuning dihedral angle. Tetrahedral constraint leads to H_2_ oxidation while square-planar constraint switches to H_2_ production.

## Graphical abstract

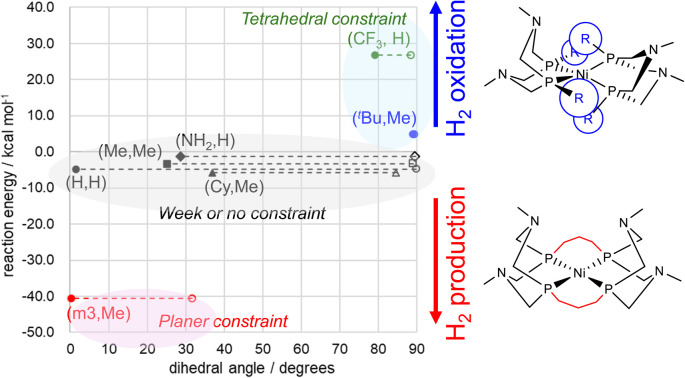

## 1. Introduction

**Scheme 1:**
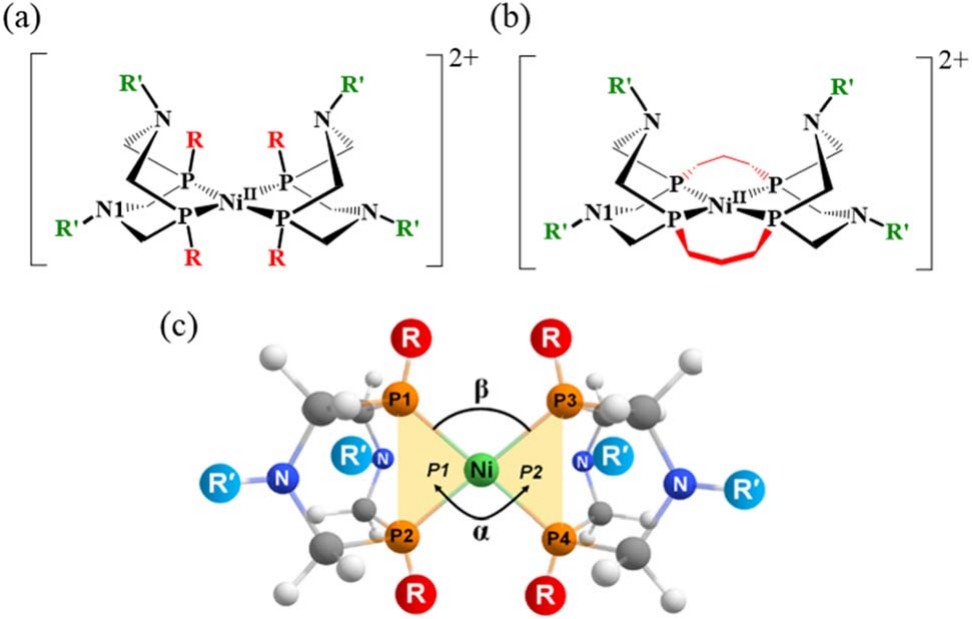
(a) Structure of Ni-diphosphine complexes [Ni(P^R^_2_N^R′^_2_)_2_]^2+^ with substituents R and R′ at the phosphorus and nitrogen atoms, respectively. Each Ni complex is denoted as (R, R′), and the combinations of substituents (R, R′) used in this study are (H, H), (Me, Me), (Cy, Me), (^*t*^Bu, Me), (CF_3_, H), and (NH_2_, H). (b) A tetradentate [m3, Me] complex, in which the –(CH_2_)_3_– unit connects P1 to P3 and P2 to P4. (c) Definition of the dihedral angle (*α*) between planes P1 and P2, and the P–Ni–P angle (*β*).

Hydrogen (H_2_) production and oxidation through catalytic processes have attracted significant interest as key technologies for alternative renewable energy systems intended to replace conventional fossil fuel-based systems.^1–4^ Ni diphosphine complexes of the form [Ni(P^R^_2_N^R′^_2_)_2_] (P^R^_2_N^R′^_2_ = 1,5-(di-R′)-3,7-(di-R)-1,5-diaza-3,7-diphosphacyclooctane, see Scheme 1a) have been developed.^5–12^ A unique feature of these complexes is the presence of two layers of coordination spheres, inspired by natural hydrogenase enzymes.^7–9^ The first layer is the Ni redox center, which serves as a hydride donor, while the second layer involves N atoms in pendant amines on the phosphine ligands, functioning as proton donors or acceptors. These two spheres operate cooperatively to deliver protons and enable H–H bond formation and cleavage in H_2_ molecules. Some Ni complexes^9,13^ have demonstrated exceptionally high turnover frequencies, exceeding 10^6^ s^−1^, and have continued to attract attention for the development of molecular photocatalysts^14,15^ and electrocatalysts that promote H_2_ production^6,13,16–18^ and oxidation.^19^

The mechanistic details and design concepts of the Ni complexes have been investigated both experimentally^10,20,21^ and theoretically.^21–23^ The controlling factors for H_2_ production and oxidation were examined from a thermodynamic perspective.^10^ Two key parameters—hydricity (hydride donor ability)^24,25^ and acidity (proton donor ability)—were analyzed in relation to ligand design. The reaction mechanism was studied using spectroscopic and electrochemical measurements^6,26,27^ as well as theoretical calculations.^21–23^ In a density functional theory (DFT) study, conformational stabilities and protonation states were explored using *ab initio* molecular dynamics (MD) trajectories, and a plausible reaction pathway was identified.^23^

Another mechanistic aspect of H_2_ production and oxidation by Ni complexes is two-state reactivity,^28,29^ which involves a spin-forbidden reaction pathway enabled by intersystem crossing (ISC). Valence d-electrons in transition metals frequently facilitate ISC between potential energy surfaces of different spin multiplicities.^28–31^ Additionally, specific ligand coordination modes can generate electronic states that permit spin flipping during the reaction. Previous theoretical studies^32,33^ have shown that, in the active site of [NiFe] hydrogenase—which inspired the development of DuBois's Ni complexes,^7–9^ ISC can occur during the H_2_ binding process. The torsional angle between S–Ni–S planes was proposed as a reaction coordinate for ISC. However, the role of two-state reactivity in the H_2_ production and oxidation processes of DuBois's complexes has not yet been explored.

This study investigates the role of substituents in Ni diphosphine complexes [Ni(P^R^_2_N^R′^_2_)_2_]^2+^ where R and R′ are located on the phosphorus and nitrogen atoms, respectively, and which catalyze H_2_ production and oxidation. By introducing bulkier substituents at the R position, the dihedral angle between the P–Ni–P planes becomes tunable, influencing the relative stability of the singlet and triplet states and the possibility of ISC along the reaction pathway. Based on these findings, ligand design guidelines favorable for H_2_ production and oxidation are proposed.

## 2. Computational details

All structures (reactants, intermediates, and transition states) were fully optimized without symmetry constraints using DFT with the B3LYP^34^ exchange-correlation functional and Grimme's D3 (ref. 35) dispersion correction (B3LYP-D3). For Ni, the Stuttgart–Dresden relativistic effective core potential (ECP) and corresponding basis set, SDD,^36^ was used. For all other atoms, the 6-31G(d,p) basis set was employed for geometry optimization, normal mode analysis, and intrinsic reaction coordinate (IRC) calculations, while the 6-311++G(2d,p) basis set was used for single-point energy calculations. Hereafter, the SDD combined with 6-31G(d,p) and SDD combined with 6-311++G(2d,p) are referred to as BS1 and BS2, respectively. Basis set convergence for energy and structural parameters was evaluated using the B3LYP-D3/BS2 results as the reference. The results, presented in Section S1 of the SI, show that the energy deviations were within 0.4 kcal mol^−1^ (Table S1). The stability of the self-consistent field (SCF) solutions was confirmed through stability analysis.^37^ Zero-point vibrational energy corrections at the BS1 level were added to the potential energy. The choice of DFT exchange–correlation functionals was validated by comparison with single-point CCSD(T) calculations for the (Me, Me) complex in a previous study.^38^ See Table S2 in Section S2 of SI for details. Transition states were confirmed by identifying a single imaginary frequency in harmonic frequency analysis. IRC calculations were performed to verify the reliability of the reaction pathways. Structure optimizations of energy minima, transition states, minimum energy crossing points (MECPs), normal mode analyses, and IRC calculations were conducted using the GRRM23 program,^39,40^ which interfaces with Gaussian16 (ref. 41) for computing potential energy, energy gradients, and Hessians. Solvation effects were included using the SMD method^42^ with parameters for acetonitrile. Spin–orbit coupling (SOC) matrix elements between spin states were calculated using the MolSOC program.^43,44^

## 3. Results and discussion

### 3.1 Relative stability of singlet and triplet states of the Ni^II^ and Ni^0^ complex with different substituents

The Ni complex, [Ni^II^(P^R^_2_N^R′^_2_)_2_]^2+^, with substituent groups at the R and R′ positions is referred to as (R, R′) as shown in Scheme 1a. This Ni^II^ complex corresponds to the C and P intermediates in the reaction mechanism. Six complexes—(H, H), (Me, Me), (Cy, Me), (^*t*^Bu, Me), (CF_3_, Me), and (NH_2_, Me)—were investigated. Based on the results, an additional complex was examined featuring a tetradentate ligand in which two bidentate ligands are connected at the phosphorus atoms by a –(CH_2_)_3_– unit. This complex is denoted as the [m3, Me] complex. Following the findings of a previous study by Raugei *et al.*,^23^ possible conformations of the (H, H) complex were optimized (Fig. S1 and Table S3). Among the optimized structures, the lowest-energy conformation—schematically shown in Scheme 1a—was selected. In this conformation, the two six-membered (Ni–P–C–N–C–P) rings in the bidentate ligands adopt boat and chair conformations, and energy of the conformation is 2.5 kcal mol^−1^ more stable than the other conformers, in qualitative agreement with the earlier study.^23^ For the other complexes, the relative stabilities of the conformers were also evaluated and are listed in Table S4. The (Me, Me), (NH_2_, Me), (CF_3_, H) and [m3, Me] complexes adopt boat–chair conformations, while in the (Cy, Me) and (^*t*^Bu, Me) complexes, the boat–boat conformation is slightly more stable.

**Fig. 1:**
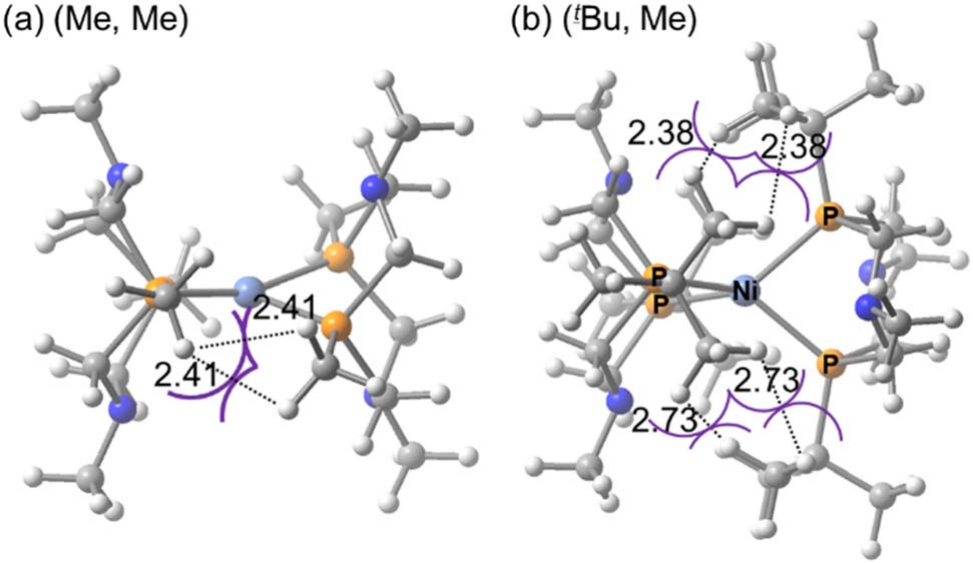
Structures of the (a) Ni^II^ (Me, Me) and (b) (^*t*^Bu, Me) complexes in singlet states (atomic distances are in Å).

The optimized structures of the [Ni^II^(P^R^_2_N^R′^_2_)_2_]^2+^ complexes in both singlet and triplet states are presented in Fig. S2. In the reaction pathway, this is a product state (P) after reductive elimination of H_2_ is completed. The relative energies Δ*E* (kcal mol^−1^), dihedral angles (°), and P–Ni–P angles (°) for the singlet (S) and triplet (T) ground states are summarized in Table 1. The definitions of the dihedral and P–Ni–P angles are provided in Scheme 1b. Structure optimization revealed that the N^II^ (H, H), (Me, Me), (Cy, Me) adopt square planar geometries in the singlet state and tetrahedral geometries in the triplet state. As a representative example, the structures of the (Me, Me) complex in singlet states is shown in Fig. 1a. In contrast, the (^*t*^Bu, Me) complex exhibits an exceptional behavior: as shown in Fig. 1b, its optimized structure is tetrahedral in both singlet and triplet states. The dihedral angle (*α*), reported in Table 1, supports this observation. Only the (^*t*^Bu, Me) complex, with a bulky ^*t*^Bu group at each P atom, exhibits a dihedral angle of 72.8°, indicating a tetrahedral configuration. Complexes with H, Me, or Cy substituents exhibit dihedral angles ranging from 0° to 30.8°, indicative of nearly square planar geometries. In the triplet state, the dihedral angles range from 74.2° to 84.3°, in clear contrast to those in the singlet state. The unusually large dihedral angle in the singlet state of the (^*t*^Bu, Me) complex arises from steric repulsion between the ^*t*^Bu groups.

**Table 1:** Relative energies Δ*E* (kcal mol^−1^), dihedral angles *α* (°), and P–Ni–P angles *β* (°) of the [Ni^II^(P^R^_2_N^R′^_2_)_2_]^2+^ complex in singlet (S) and triplet (T) states (B3LYP-D3//BS1 results)

(R, R′)	Δ*E* (kcal mol^−1^)		*α* (°)		*β* (°)	
	S	T	S	T	S^a^	T (P1–Ni–P3)/(P2–Ni–P4)
(H, H)	0.0	11.4	0.0	81.6	96.2	164.9/106.3
(Me, H)	0.0	5.4	24.4	84.3	99.4	107.9/107.8
(Me, Me)	0.0	5.2	26.5	78.6	100.5	104.9/104.9
(Cy, Me)	0.0	7.4	30.8	74.2	104.0	117.3/117.3
(^*t*^Bu, Me)	14.9	0.0	72.8	78.5	113.8	142.6/111.4

a P1–Ni–P3 and P2–Ni–P4 angles are equal to the first decimal place in the singlet state.

The structural constraint in the Ni^II^ (^*t*^Bu, Me) complex significantly affects the relative stability of the singlet and triplet states. In the (H, H), (Me, Me), and (Cy, Me) complexes, the singlet state is more stable than the triplet state. In contrast, this trend is reversed in the (^*t*^Bu, Me) complex, where the triplet state is 14.9 kcal mol^−1^ more stable than the singlet state, as shown in Table 1. Compared with the (Me, Me) complex, the relative energy difference is reversed by 20.1 kcal mol^−1^ (5.2 + 14.9). From a ligand design perspective, it is important to note that introducing a ^*t*^Bu group can reverse the singlet–triplet stability by approximately 20 kcal mol^−1^.

In contrast to the (^*t*^Bu, Me) complex, the (Cy, Me) complex exhibits a less distorted dihedral angle (30.8°) and does not adopt a tetrahedral conformation in the singlet state. This indicates that the Cy groups can avoid steric repulsion even at relatively small dihedral angles. Each Cy group interacts with the other through a C–H moiety adjacent to the P atom. In the (^*t*^Bu, Me) complex, this corresponding carbon is a tertiary carbon bearing a methyl group. Additionally, the Cy groups exhibit attractive interactions with the N atoms of the opposing ligands, with an H–N distance of 2.46 Å, as shown in Fig. S2. Therefore, the dihedral angle in the (Cy, Me) complex is governed by a balance between repulsive H–H and attractive H–N interactions, resulting in a nearly planar geometry in the singlet state.

**Fig. 2:**
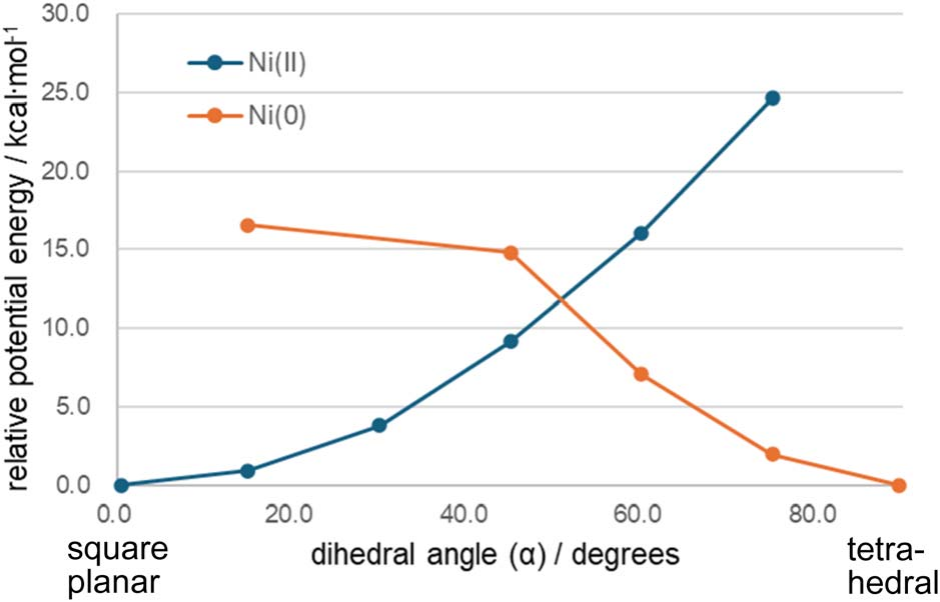
Potential energy curves of Ni^0^ and Ni^II^ (H, H) complexes. The energy reference for each complex is its fully optimized structure (*α* = 0.4° for Ni^II^ and *α* = 89.4° for Ni^0^). For all other structures, only the dihedral angle was fixed, while all other structural parameters were optimized.

We also calculated the structure of the Ni^0^ (H, H) complex, Ni^0^(P^H^_2_N^H^_2_)_2_, in singlet state for comparison. In the reaction pathway, this Ni^0^ complex corresponds to the R intermediate which is a resting state before accepting protons. The Ni^0^ center has a d^10^ electron configuration, and isotropic ligand coordination leads to a tetrahedral geometry. The dihedral angle (*α*) between the two Ni–P–P planes is 89.5°, in clear contrast to the Ni^II^ case (0.4 degrees). Constrained structure optimization with fixed *α* was performed, and the results are shown in Fig. 2 and S3. By twisting the dihedral angle by 45°, the Ni^II^ complex is destabilizes by approximately 9 kcal mol^−1^. Similarly, the 45° twist from the tetrahedral structure destabilizes the Ni^0^ complex by about 15 kcal mol^−1^. The variation in stability with structural distortion should be recognized as a valuable feature in molecular design.

### 3.2 Energy profiles of H_2_ production and oxidation

**Scheme 2:**
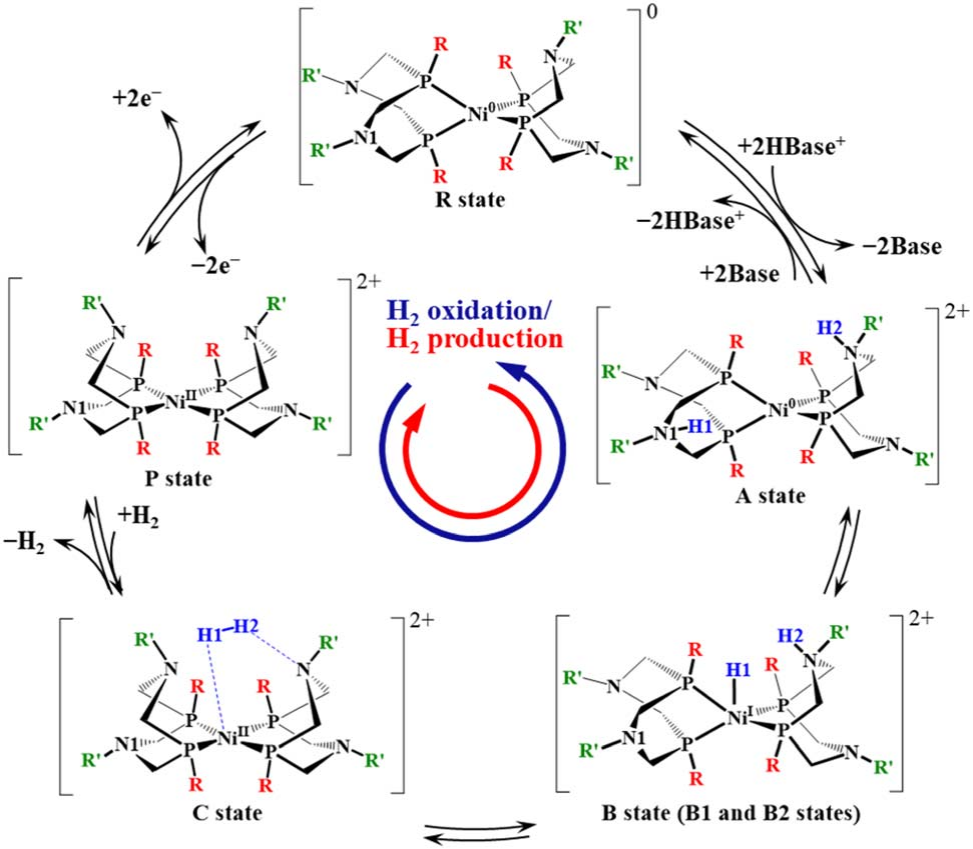
Reaction pathway considered in this study.

The reaction cycle is described below in the direction of H_2_ production. The reaction pathway for H_2_ production/oxidation by [Ni(P^R^_2_N^R′^_2_)_2_]^2+^ complexes has been proposed in previous studies.^23,45–47^ Two major mechanisms—EECC and ECEC—have been discussed, where E and C represent electron and proton transfers, respectively. Depending on the applied potential, either the EECC or ECEC mechanism may dominate. According to a kinetic study,^47^ the EECC pathway proceeds faster than the ECEC pathway. In this study, the EECC mechanism shown in Scheme 2 was adopted. H_2_ production begins with the adsorption of two protons (H1 and H2) at the pendant amine ligands (N1 and N2) of the Ni^0^ resting state (R), forming the diprotonated intermediate (A) with a Ni^0^ metal center. A Brønsted acid (protonated base, HBase^+^) serves as the proton source. The A intermediate then undergoes an intramolecular proton-transfer transition state (TS_B1_), resulting in the hydride–proton intermediate (B1), where one proton (H1) migrates from the amine (N1) to the Ni center. Following a transition state (TS_B2_) with a very low energy barrier, the hydride at the Ni center adopts a new conformation (B2 intermediate) favorable for H–H bond formation. This conformational change was observed only in the (H, H), (Cy, Me), and (^*t*^Bu, Me) complexes. The B2 intermediate then undergoes a second intramolecular proton transfer (H2) *via* a transition state (TS_C_), producing the H_2_ complex (C) with a Ni^II^ center. Finally, in the product state P, the Ni^II^ complex and the generated H_2_ molecule are treated as isolated species. The P state accepts two electrons to regenerate the R state, followed by two proton transfers to form the A intermediate again.

**Fig. 3:**
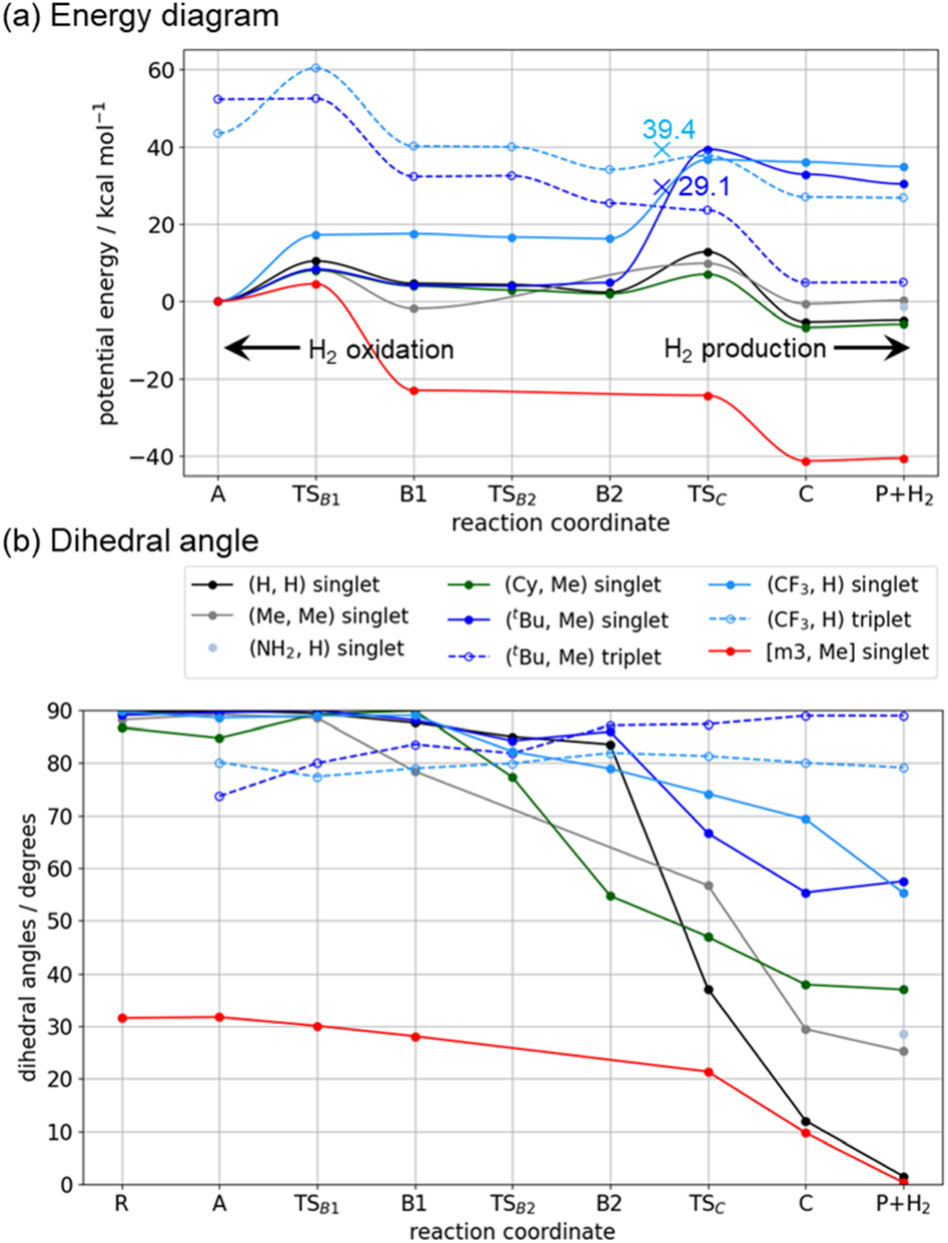
(a) Potential energy diagram with zero-point vibrational energy correction, calculated at the B3LYP-D3/BS2//B3LYP-D3/BS1 level. The legends are consistent with those in Fig. 3a. The “×” symbol indicates the minimum-energy intersystem crossing point. The energy of the crossing point does not include zero-point energy correction. (b) Dihedral angle (*α*) of the Ni complex along the reaction pathway (for definition, see Fig. 2).

According to the [NiFe] hydrogenase model,^32,33^ a low-lying triplet state appears along the reaction pathway. Energy diagrams for both singlet and triplet states were obtained, and the results for the (H, H), (Me, Me), (Cy, Me) and (^*t*^Bu, Me) complexes are summarized in Fig. 3 and S4 of the SI. For clarity, some of the triplet-state diagrams are omitted in Fig. 3. The potential energies of the singlet and triplet states are closely aligned around the TS_C_ region. For the (H, H) complex, CCSD/6-311+G(2d,p) results indicate that the ^1^TS_C_ state is 4.3 kcal mol^−1^ more stable than the corresponding triplet state. DFT results for the (H, H), (Me, Me), and (Cy, Me) complexes also show no intersystem crossing, as illustrated in Fig. S4. An exception case is the (^*t*^Bu, Me) complex, which will be discussed later.

#### 3.2.1 (H, H), (Me, Me), (Cy, Me) and (^*t*^Bu, Me) cases

An overview of the energy profile and structural changes is presented in this section, while specific features of each complex are discussed in the following sections. These Ni complexes are classified into two groups. The first group includes the (H, H), (Me, Me), and (Cy, Me) complexes, in which the reaction proceeds through the singlet state. In this group, the ^1^A and ^1^(P+H_2_) states are nearly isoenergetic, and the H_2_ production process is calculated to be slightly exothermic, with reaction energies ranging from −3.2 to −5.9 kcal mol^−1^. The rate determining step (RDS) is either ^1^TS_B1_ (Ni–H formation) or ^1^TS_C_ (reductive elimination), depending on the substituents. For the (H, H) and (Me, Me) complexes, the RDS is ^1^TS_C_, with apparent activation energies of 12.8 kcal mol^−1^ and 9.2 kcal mol^−1^, respectively. In the (Cy, Me) complexes, the RDS is ^1^TS_B1_, with an activation energy of 8.0 kcal mol^−1^.

**Fig. 4:**
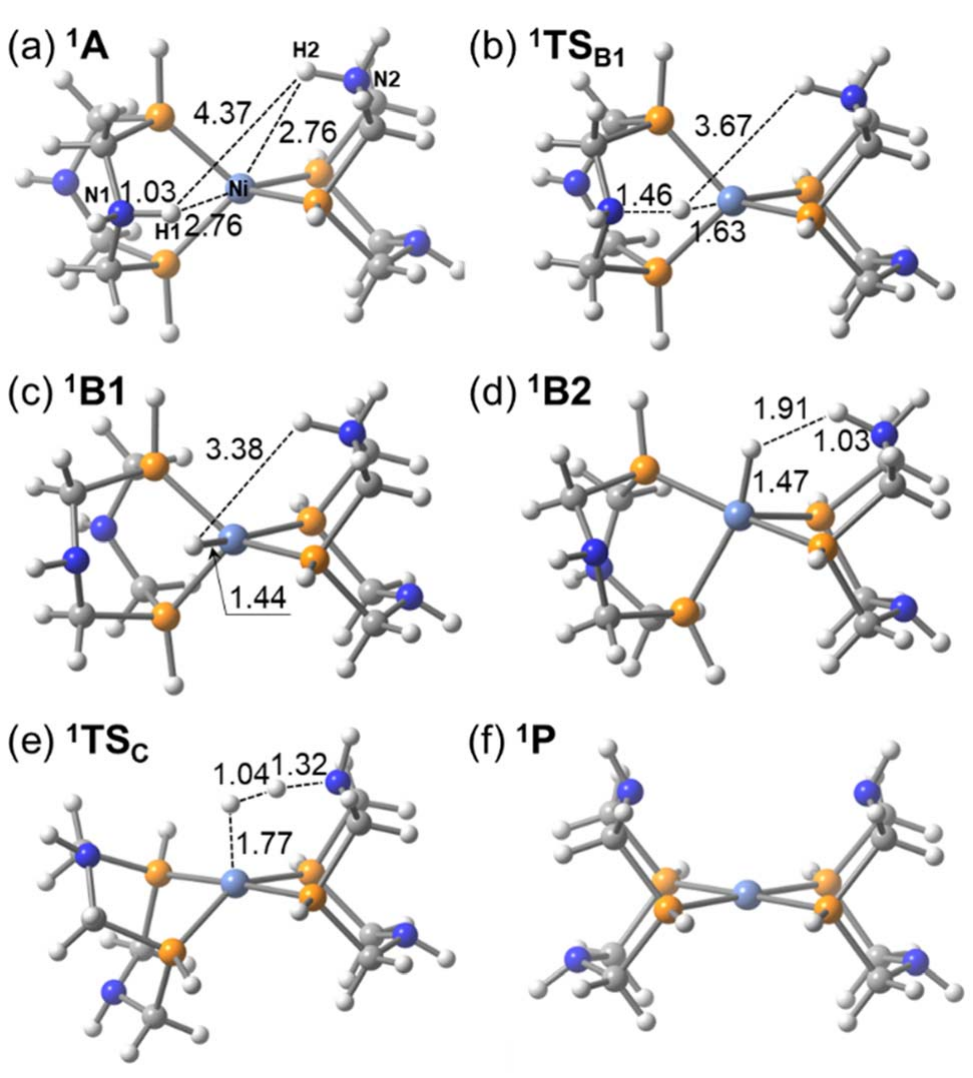
Optimized structures of the (H, H) complex in (a) ^1^A, (b) ^1^TS_B1_, (c) ^1^B1, (d) ^1^B2, (e) ^1^TS_C_ and (f) ^1^P (bond lengths are in Å).

As a representative case, the (H, H) complex was selected, and the optimized structures of the intermediates and transition states are shown in Fig. 4. For the other complexes, qualitatively similar structures were obtained, as shown in Fig. S5(a)–(i) in SI. Structural changes are described along the H_2_ production pathway. The ^1^A state is formed after the ^1^R state accepts two protons at the nitrogen atoms of the pendant amines. Both the H1–Ni and H2–Ni distances are 2.76 Å. One of the protons (H1) is transferred to the Ni center *via*^1^TS_B1_, during which the H1–Ni distance shortens to 1.63 Å. The structure of the ^1^B1 state adopts a pseudo-trigonal bipyramidal geometry. The complex then undergoes structural isomerization to reach the ^1^B2 state, where the H1–H2 distance becomes 1.91 Å, indicating that interaction for H–H bond formation has already begun (see Fig. 4d). The H_2_ molecule is formed *via* the ^1^TS_C_ state, where the dihedral angle *α* between the two Ni–P–P planes decreases to below 40°. H_2_ formation completes at the ^1^C and ^1^(P+H_2_) states, where the dihedral angle approaches 90°, reflecting the characteristic structure of the Ni^II^ complex.

The second group comprises the (^*t*^Bu, Me) complex. The primary distinction from the (H, H), (Me, Me), and (Cy, Me) complexes lies in the behavior of the singlet surface. As shown in Fig. 3a, the singlet states of the (^*t*^Bu, Me) complex in ^1^TS_C_, ^1^C, and ^1^(P+H_2_) are less stable than their corresponding triplet states by 15.8, 28.1, and 25.5 kcal mol^−1^, respectively. In contrast, the energy levels of the ^1^A, ^1^TS_B1_, ^1^B1, ^1^TS_B2_ and ^1^B2 states are comparable to those of the other complexes. This instability arises from structural distortion caused by the steric hindrance of the bulky ^*t*^Bu ligands. As shown in Fig. 1b, the ^*t*^Bu groups on the two P_2_N_2_ ligands avoid each other while coordinating to the Ni center, resulting in a tetrahedral conformation. In the ^1^TS_C_, ^1^C, and ^1^(P+H_2_) states, after two H^+^ are reduced, the metal center is oxidized to Ni^II^. As shown in Table 1 and Fig. 2, the Ni^II^ complexes of (H, H), (Me, Me) and (Cy, Me)—which lack steric hindrance—favor a square planar structure in the singlet state. The dihedral angle *α* in the ^1^(P+H_2_) state is less than 40°, as seen in Fig. 3b. In contrast, the dihedral angle in the (^*t*^Bu, Me) complex is 57.5°, and is constrained toward a tetrahedral conformation due to the presence of the ^*t*^Bu group.

As the relative stability of the singlet and triplet states reverses around the ^1^TS_C_ state, intersystem crossing is expected. The intersystem crossing point was located at 29.1 kcal mol^−1^ (relative to the ^1^A state, without zero-point energy correction) between the ^1^B2 and ^1^TS_C_ states, and calculated SOC matrix element is 44.9 cm^−1^ (for more details, see Table S5).

A key finding from the (^*t*^Bu, Me) case is that the thermodynamics of H_2_ production and oxidation in Ni complexes can be tuned by imposing square planar or tetrahedral constraints through steric hindrance of the substituents. As a result, the (^*t*^Bu, Me) complex is more favorable for H_2_ oxidation than the other complexes. Along the reaction pathway involving intersystem crossing, ^3^(P+H_2_) →→ ^3^TS_C_ → ^1^B2 →→ ^1^A, H_2_ oxidation is exothermic, with a calculated reaction energy of −4.9 kcal mol^−1^. The rate-determining step is the intersystem crossing between the ^3^TS_C_ and ^1^B2 states, with a barrier height of 24.2 kcal mol^−1^.

#### 3.2.2 Controlling the dihedral angle of the Ni complex for improving H_2_ production capability

In the (H, H), (Me, Me), and (Cy, Me) complexes, the reaction energy for H_2_ production ranges from −3.2 to −5.9 kcal^−1^. With the introduction of ^*t*^Bu ligands the reaction energy increases to +4.9 kcal mol^−1^, making the (^*t*^Bu, Me) complex thermodynamically unfavorable for H_2_ production but favorable for H_2_ oxidation. This section explores ligand design strategies to enhance the suitability of Ni complexes for H_2_ production.

To modify the energy profile to favor exothermic H_2_ production, the energy level of the ^1^A state can be raised relative to that of the ^1^C state. As noted, this relative energy can be adjusted by the dihedral angle *α*. As shown in Fig. 2, constraining the dihedral angle to 45 degrees effectively raises the energy level of the Ni^0^ complex by approximately +15 kcal mol^−1^. The ^1^A state is in Ni^0^ oxidation state, which prefers a tetrahedral geometry. Based on this, the Ni^0^ complex was forced into a square planar geometry by linking the two bidentate phosphine ligands. In the [Ni(P^R^_2_N^R′^_2_)_2_] structure, a trimethylene, –(CH_2_)_3_–, unit was introduced at the R position to connect the two P_2_N_2_ ligands. A methyl (Me) group was used for the R′ substituent. This new complex is referred to as [m3, Me] and is shown in Scheme 1b.

**Fig. 5:**
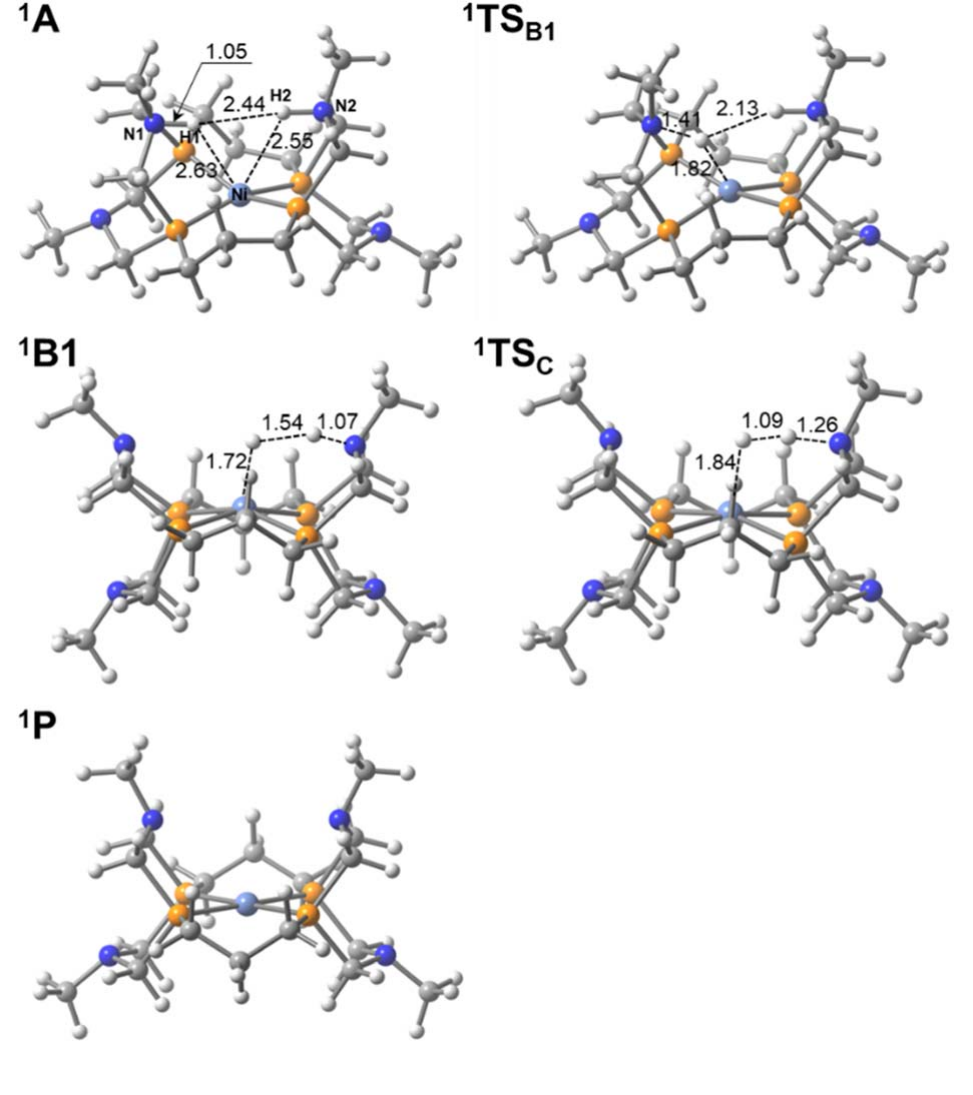
Optimized structures of the [m3, Me] complex in the singlet state (bond lengths are in Å).

The potential energy profiles for H_2_ production/oxidation by the [m3, Me] complex in the singlet and triplet states were calculated and are shown in Fig. 3a (singlet state) and Fig. S4 (both singlet and triplet states). The optimized structures of the intermediates and transition states in singlet states are presented in Fig. 5 and S5f. H_2_ production proceeds along the reaction pathway in the singlet state, with an energy barrier of 4.5 kcal mol^−1^ at ^1^TS_B1_. This is the lowest barrier among all Ni complexes examined in this study. Following the ^1^B1 intermediate, the complex passes through ^1^TS_C_ with a negligible barrier and proceeds to ^1^C and ^1^P to form the H–H bond. The reason for the low-barrier heights of the transition states is in the structural similarities between ^1^A and ^1^TS_B1_ and between ^1^B1 and ^1^TS_C_. Especially, the H–H distance in ^1^B1 is 1.54 Å and is close to that in ^1^TS_C_ (1.09 Å). This is in clear contrast to those in the (H, H) complex, where the H–H distance in ^1^B2 is 1.91 Å as shown in Fig. 4. The tetradentate P_4_N_4_ ligand not only constrains square planar geometry but also imposes the structures of ^1^A and ^1^B1 close to that of ^1^TS_C_. The reaction energy for H_2_ production is −40.6 kcal mol^−1^, making it highly exothermic and thermodynamically the most favorable among the studied Ni complexes. The structural constraint effectively raises the energy levels of the ^1^A and ^1^B1 states. The dihedral angle of the [m3, Me] complex along the reaction pathway is also shown in Fig. 3b. The trend in dihedral angle differs markedly from that of the other complexes, ranging from 31.7° to 0.3° across the ^1^A, ^1^TS_B1_, ^1^B1, ^1^TS_C_, ^1^C, and ^1^P states.

Previous studies reported experimental results for Co^48,49^ and Ni^13^ complexes bearing tetradentate phosphine ligands. Wiedner and co-workers synthesized Co complexes with P_4_N_2_-type tetradentate phosphine ligands^48^ and successfully achieved H_2_ production with a high turnover frequency (1.8 × 10^4^ s^−1^) among the highest for molecular catalysts.^49^ This turnover frequency was further enhanced in a tetradentate Ni complex, reaching up to 1.6 × 10^6^ s^−1^.^13^ Thus, the proposed molecular design concept is supported both theoretically by proof-of-concept DFT calculations and experimentally by the high catalytic activity of tetradentate ligands.

Notably, previous studies demonstrated a linear correlation between the dihedral angle (*α*)^50^ and the hydride donor ability 
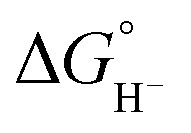
 of [HM(LH)]^2+^, where M and L represent the metal and ligand, respectively.^51^
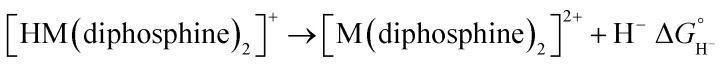


A smaller dihedral angle increases donor ability, contributing to the exothermicity of the elementary step from the ^1^B to ^1^C states. However, 
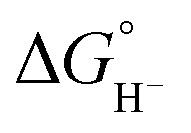
 is only one factor in explaining the reactivity. The energy difference between the ^1^B and ^1^(P+H_2_) states also includes the energy required for proton release ([HM(LH)]^2+^ → [HML]^+^ + H^+^) and H_2_ formation (H^+^ + H^−^ → H_2_).^49,52^ Moreover, 
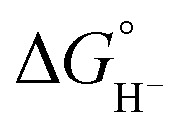
 is not related to the energy difference between the ^1^A and ^1^B1 states. The results for the [m3, Me] complex indicate that enforcing a square planar geometry in the ^1^A and ^1^B1 states is a key strategy for promoting efficient H_2_ production.

#### 3.2.3 Classification of the Ni complexes

**Fig. 6:**
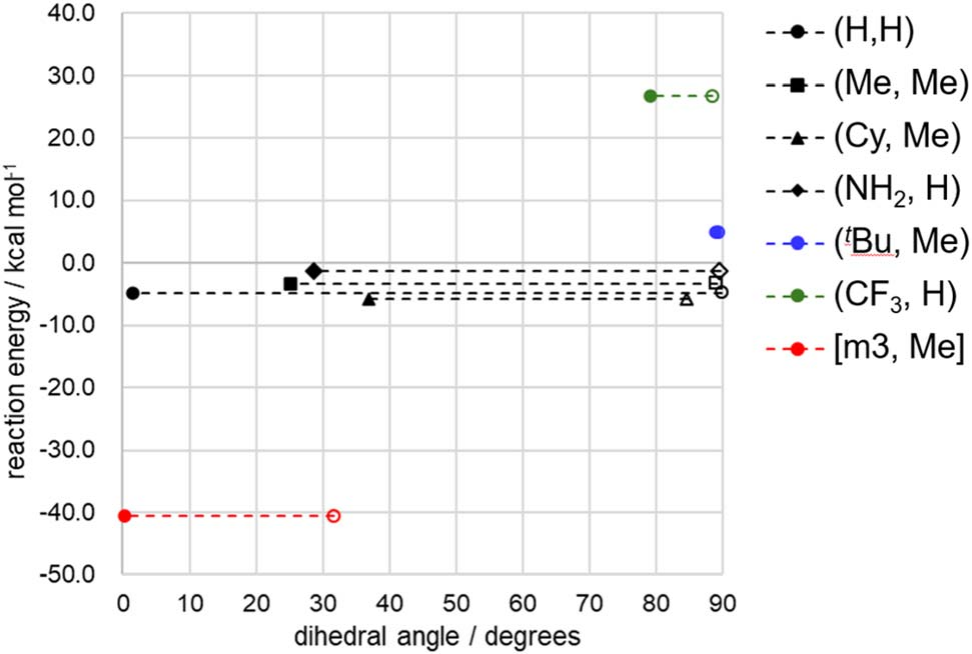
Reaction energies for the H_2_ production (from A to P+H_2_ state) is plotted against the dihedral angles of the A and P+H_2_ states. Filled and open symbols represent the P+H_2_ and A states, respectively. The dashed line connects the A to P+H_2_ states, indicating the change in dihedral angle during the reaction. Single-point B3LYP-D3/BS2 calculations with zero-point vibrational correction were used to determine the reaction energies. For the (^*t*^Bu, Me) and (CF_3_, H) complexes, pathways *via* the intersystem crossing point were used, and the reaction energy is defined as the energy difference between the triplet ^3^(P+H_2_) state and the singlet ^1^A state. Numerical data are provided in Table S6 of the SI.

The five complexes discussed above are classified into three categories (I–III) based on ligand rigidity and the reaction energy associated with H_2_ production/oxidation. In Fig. 6, the reaction energy for H_2_ production is plotted against the change in dihedral angle from the A to P+H_2_ states. The dashed horizontal lines in Fig. 6, connecting the A to P+H_2_ states for each complex, represent the change in dihedral angle along the reaction pathway. Group I includes the (H, H), (Me, Me), and (Cy, Me) complexes, which exhibit slightly exothermic reaction energies for H_2_ production. These ligands are relatively less bulky and flexible enough to accommodate structural changes between tetrahedral and square planar geometries depending on the oxidation state of the Ni center. This is reflected by the characteristic long horizontal lines observed in Fig. 6. Group II consists of the (^*t*^Bu, Me) complex, which thermodynamically favors H_2_ oxidation. The dihedral angles remain around 90° due to steric hindrance, which restricts structural rearrangement of the Ni complex. Group III comprises the [m3, Me] complex featuring a tetradentate ligand. The dihedral angles range from 0° to 30°, indicating strong structural enforcement toward planar configurations. This complex shows a highly exothermic profile for H_2_ production.

The ligands were selected to control the dihedral angles of the Ni complexes through structural constraints. As shown in Fig. 2, these dihedral angles clearly influence the relative stability of the tetrahedral and square planar geometries in the Ni^0^ and Ni^II^ complexes. However, additional factors should be considered in ligand design. In particular, the electron-withdrawing and electron-donating properties of the substituents may influence the trends observed in Fig. 6. To examine these effects, the (CF_3_, H) and (NH_3_, H) complexes were analyzed. The (CF_3_, H) complex was compared with the (^*t*^Bu, Me) complex, as the CF_3_ group is similarly bulky but more electron-withdrawing than the ^*t*^Bu group. The (NH_2_, H) complex was compared with the group I complexes to assess the impact of the electron-donating nature of the NH_2_ group.

The results are also presented in Fig. 6. The CF_3_ group clearly constrains the structure to a tetrahedral geometry. The dihedral angles in the ^1^A and ^3^(P+H_2_) states are 88.6° and 79.2°, respectively. The reaction energy for H_2_ oxidation is exothermic by −26.8 kcal mol^−1^, which is significantly greater than that of the (^*t*^Bu, Me) complex (−4.9 kcal mol^−1^). This difference arises because the triplet state energy levels in the ^3^C and ^3^(P+H_2_) states are higher for the CF_3_-substituted complex than for the (^*t*^Bu, Me) complex, as shown in Fig. 3a. Energy diagram of the (CF_3_, H) complex shown in Fig. 3b also indicates that the H_2_ oxidation is feasible. As in the (^*t*^Bu, Me) case, the initial state of the (CF_3_, H) complex is in triplet state, ^3^(P+H_2_) state. In TS_C_, singlet and triplet states are nearly degenerate, and the energy difference is only 1.0 kcal mol^−1^. Minimum energy ISC point was located at 39.4 kcal mol^−1^, which is higher than both ^1^TS_C_ and ^3^TS_C_ by 3.3 and 1.7 kcal mol^−1^, respectively. This ISC point is at 12.6 kcal mol^−1^ from the ^3^(P+H_2_) state and by 13.9 kcal mol^−1^ smaller than that of the (^*t*^Bu, Me) case. Structure of the ISC point is halfway between ^1^B2 and ^3^TS_C_ as shown in Fig. S5g. Calculated SOC is 26.1 cm^−1^, which is in the same order as that of the (^*t*^Bu, Me) case (44.9 cm^−1^). After ISC, the reaction should go through the singlet surface until two protons are formed at ^1^A state. The electron-withdrawing nature of the CF_3_ group enhances the H_2_ oxidation capability of the Ni complex. In contrast, the (NH_2_, H) complex exhibits behavior similar to that of the group I complexes in both structural changes and reaction energy toward H_2_ production.

As the reaction barrier in the reaction pathway would block the H_2_ reduction and oxidation reaction, we summarized the reaction barrier of the Ni complexes in Table S7. The result show that the activation barrier would be small enough not to block the H_2_ reduction in the groups I and III complexes and the H_2_ oxidation in the group II complexes. One exception is the H_2_ oxidation by the (^*t*^Bu, Me) case in which calculated activation barrier is 24.2 kcal mol^−1^. Those for the other complexes are less than 13 kcal mol^−1^, illustrating the catalytic ability of the Ni complexes.

The final part of this section discusses the influence of acids and bases on the reaction energy. In H_2_ oxidation experiments, bases such as triethylamine, NEt_3_, are often used as proton acceptor.^5,19,24^ H_2_ production experiments commonly employ [DMF(H)][OTf] as a proton donor.^6,13,16^ In this study, NEt_3_ and [DMF(H)][OTf] were adopted as representative base and acid, respectively. As shown in Table 2, the acid and base strongly promote H_2_ production and oxidation, respectively. In group I, the acid and base play a decisive role. The energy profile of the (Me, Me) complex becomes exothermic toward H_2_ production and oxidation in the presence of [DMF(H)][OTf] and NEt_3_, respectively. In contrast, the (CF_3_, H) complex in group II is exothermic toward H_2_ oxidation under both NEt_3_ and [DMF(H)][OTf]. The [m3, Me] complex in group III favors H_2_ production regardless of the presence of acid or base. These results indicate that the properties of the complexes can be tuned through ligand design to surpass the influence of external acids and bases.

**Table 2:** Reaction energies including the proton donor in H_2_ production and the proton acceptor in H_2_ oxidation (values are given in kcal mol^−1^)

	Δ*E* (H_2_ oxidation)^a^		Δ*E* (H_2_ production)^a^	
	H^+^ acceptor: NEt_3_		H^+^ donor: [DMF(H)][OTf]	
	B3LYP-D3	ωB97XD	B3LYP-D3	ωB97XD
(Me, Me)	−13.7	1.5	−14.9	−25.2
(^*t*^Bu, Me)	−20.4	−5.2	−8.2	−18.5
(CF_3_, H)	−61.6	−44.1	33.0	20.4
[m3, Me]	26.8	43.3	−55.4	−67.0

a Reaction energy with zero-point vibration energy correction. For H_2_ oxidation, Δ*E* = *E*(**R**) + 2*E*(H^+^NEt_3_) − *E*(**P**) − *E*(H_2_) − 2*E*(NEt_3_). For H_2_ production, Δ*E* = *E*(**P**) + *E*(H_2_) + 2*E*([DMF][OTf]^−^) − *E*(**R**) − 2*E*([DMF(H)][TOf]).

## 4. Conclusion

The Ni complexes developed by DuBois *et al.*^5–12^ have demonstrated strong potential for electrocatalytic H_2_ production and oxidation. As these two reactions proceed in opposite directions, ligand design plays a critical role in controlling their directionality. In this study, DFT calculations were performed for [Ni(P^R^_2_N^R′^_2_)_2_] complexes with (R, R′) = (H, H), (Me, Me), (Cy, Me), (^*t*^Bu, Me), (CF_3_, H) and (NH_2_, H) to investigate how ligands influence H_2_ production and oxidation activity. Based on the analysis of conformational changes during the reaction, a [m3, Me] complex featuring a P_4_N_4_-type tetradentate ligand is proposed. A proof-of-concept calculation was conducted to evaluate its energy profile.

To understand fundamental properties, the structures of the Ni^II^ and Ni^0^ complexes were examined. In the singlet state, the Ni^II^ complex adopts a square planar conformation, whereas the Ni^0^ complex prefers a tetrahedral conformation. A notable exception is the Ni^II^ (^*t*^Bu, Me) complex, which exhibits a tetrahedral structure in the singlet state due to steric repulsion between the bulky ^*t*^Bu groups, forcing the complex into a tetrahedral geometry. This steric hindrance in the Ni^II^ state selectively destabilizes the energy levels of the TS_C_, C, and P states in the singlet state. As a result, the triplet state becomes the ground state for these intermediates in the Ni^II^ (^*t*^Bu, Me) complex. This finding provides a rationale for designing a tetradentate ligand.

The energy diagrams of the (H, H), (Me, Me), and (Cy, Me) complexes are slightly exothermic—by −3.2 to −5.9 kcal mol^−1^—toward H_2_ production, whereas that of the (^*t*^Bu, Me) complex is exothermic by −4.9 kcal mol^−1^ toward H_2_ oxidation. Structural analysis suggests a ligand design in which two bidentate P^R^_2_N^R′^_2_ ligands are connected by trimethylene –(CH_2_)_3_– units to enforce a square planar geometry in the Ni^0^ complex. This ligand design effectively destabilizes the ^1^A, ^1^TS_B1_ and ^1^B1 states of the [m3, Me] complex, shifting the reaction energy for H_2_ production to a highly exothermic value of −40.6 kcal mol^−1^. The calculated barrier height at the rate-determining ^1^TS_B1_ state is 4.5 kcal mol^−1^. These findings are consistent with experimental studies on related systems^13^ and offer valuable guidance for the design of high-performance catalysts.

To verify this understanding, an electron-withdrawing CF_3_ group and an electron-donating NH_2_ group were introduced. The reaction energy of the (NH_2_, H) complex remains consistent with the trend observed in group I complexes. The sterically bulky CF_3_ group enforces a tetrahedral conformation and destabilizes the ^3^P state, increasing the reaction energy for H_2_ oxidation to −26.8 kcal mol^−1^.

These Ni complexes were classified into three groups. Group I included the (H, H), (Me, Me), (Cy, Me), and (NH_2_, H) complexes. Group II comprised the (^*t*^Bu, Me) and (CF_3_, H) complexes, which exhibited exothermic behavior toward H_2_ oxidation. The [m3, Me] complex belonged to group III and favored H_2_ production. The effect of proton donors and acceptors on H_2_ production and oxidation was examined by introducing [DMF(H)][OTf] and NEt_3_, respectively. The presence of acid and base strongly influences the direction of the reaction toward H_2_ production or oxidation, respectively. However, our calculations indicate that group II Ni complexes—particularly the (CF_3_, H) complex—can thermodynamically favor H_2_ oxidation even in the presence of acid. In contrast, the [m3, Me] complex prefers H_2_ production even under the presence of base.

The results of this study show that the conformation of the Ni complex can effectively and selectively shift specific energy levels along the reaction pathway. Designing the ligand structure is essential for controlling the directionality of H_2_ production or oxidation.

## Conflicts of interest

There are no conflicts to declare.

## Supplementary Material

RA-015-D5RA05545H-s001

## Data Availability

The data supporting this article (basis-sets and DFT-functional dependences, structures of Ni complexes, potential energy of the Ni complex in singlet and triplet states, spin-orbit coupling constants at intersystem crossing points, summary of reaction barrier and reaction energy, and atomic coordinates) are provided in the supplementary information (SI). Supplementary information is available. See DOI: https://doi.org/10.1039/d5ra05545h.
