# Supplementary material for: Conformational constraints in [Ni(PR2NR′2)2] complexes for tuning H2 production and oxidation: a DFT-based ligand design study
Source: RSC Adv. 2025 Sep 29;15(43):35961–70. doi: 10.1039/d5ra05545h (PMC12477667; doi:10.1039/d5ra05545h)
Supplement: RA-015-D5RA05545H-s001 [file RA-015-D5RA05545H-s001.pdf]

Supplementary Information (SI) for RSC Advances.
This journal is © The Royal Society of Chemistry 2025

Supplementary Information

Conformational Constraints in $[Ni(P^{R}_2N^{R'}_2)_2]$ Complexes for Tuning H_2 Production and Oxidation: A DFT-Based Ligand Design Study

Sarinya Hadsadee,[1,2] Manussada Ratanasak,[1,3] Phiphob Naweephattana,[1] Sumiko Morita,[1] Ray Miyazaki,[1] Kenji Iida,[1] Akira Nakayama,[1,4] Siriporn Jungsuttiwong ,[2*] Jun-ya Hasegawa,[1*]

[1]Institute for Catalysis, Hokkaido University, N21 W10 Kita-ku, Sapporo, Hokkaido 001-0021, Japan

[2]Department of Chemistry and Center of Excellence for Innovation in Chemistry, Ubon Ratchathani University, Ubon Ratchathani 34190, Thailand.

[3]Present address: Center for Computational Sciences, University of Tsukuba, 1-1-1 Tennodai, Tsukuba, Ibaraki 305-8577, Japan

[4]Present address: Department of Chemical System Engineering, Graduate School of Engineering, The University of Tokyo, 7-3-1 Hongo, Bunkyo 113-8656, Tokyo

Contents

S1. Basis sets convergence in the structure optimization

S2. Exchange-correlation functional for the single-point calculations

S3. Optimized structures of the Ni^{II} complex in singlet and triplet spin multiplicities

S4. Structure of the Ni^{II} complexes

S5. Structure optimization of the complex with Ni^{0} and Ni^{II} : Potential energy vs dihedral angles

S6. Potential energy of the H_2 production/oxidation catalyzed by the Ni complexes

S7. Structures of intermediate and transition states of the Ni complexes

S8. Singlet-triplet spin-orbit coupling at the intersystem crossing point in the potential energy surface of (^{t}Bu, Me) complex

S9. Reaction energy and change of dihedral angles in the H_2 production

S10. Atomic coordinates

S1. Basis sets convergence in the structure optimization

The basis set convergence of the optimized structures was evaluated. For the target system in the examination, the simplest (H, H) complex was chosen. For key intermediates and transition states, optimized structures obtained using the 6-31G(d,p), 6-31+G(d,p), and 6-311+G(2d,p) basis sets were compared with those obtained using the 6-311++G(2d,p) basis sets. For Ni, Stuttgart/Dresden type effective core potential[1] was used. The B3LYP exchange-correlation functional with the D3-type empirical dispersion[2] was employed. The results are summarized in **Table S1**. The optimized structures obtained using the 6-31G(d,p) basis set already exhibit good agreement with those obtained using the 6-311++G(2d,p) basis set. While some root-mean-square deviations (RMSD) for dihedral angles are relatively large, they remain within 2 degrees. The dihedral angles with larger deviations involve three nearly collinear atoms, such as P–Ni–P. In these cases, even a small structural change can lead to a large deviation.

We also evaluated the accuracy of potential energy at the optimized structures obtained using the reduced basis sets. Potential energy from single-point calculations with the 6-311++G(2d,p) basis sets was compared with that obtained from structure optimization at the 6-311++G(2d,p) level. The results are also presented in **Table S1**. The potential energy from the single-point calculation with the 6-31G(d,p) optimized structure shows good agreement with that from the 6-311++G(2d,p) structure optimization. The largest deviation was 0.42 kcal mol^{-1} for $^{1}TS_{C}$ in the singlet spin state. These results indicate that the 6-31G(d,p) basis sets show sufficient convergence in structure optimization of the present system.

Table S1. RMSD in structural parameters and deviation ΔE from the B3LYP-D3/6-311++G(2d,p) optimized structure and potential energy.

Basis sets	RMSD[a]			ΔE[b]
	B	A	D	
Singlet state				
[1]A intermediate				
6-31G(d,p)	0.00401	0.285222	0.86143	0.28
6-31+G(d,p)	0.00408	0.192031	0.49334	0.23
6-311+G(2d,p)	0.00006	0.021773	0.05991	0.00
$^{1}TS_C$				
6-31G(d,p)	0.01522	0.854461	1.74943	0.42
6-31+G(d,p)	0.00507	0.214556	0.66928	0.41
6-311+G(2d,p)	0.00015	0.013483	0.02792	0.20
[1]C intermediate				
6-31G(d,p)	0.00729	0.464234	1.15656	0.32
6-311+G(2d,p)	0.00015	0.013483	0.28035	0.00
Triplet state				
[3]A intermediate				
6-31G(d,p)	0.01197	0.306142	0.68892	0.31
6-311+G(2d,p)	0.00191	0.050028	0.13354	0.01
$^{3}TS_C$				
6-31G(d,p)	0.00928	0.370698	0.91984	0.32
6-311+G(2d,p)	0.01011	0.280744	0.83558	0.00
[3]C intermediate				
6-31G(d,p)	0.01026	0.414772	0.93478	
6-311+G(2d,p)	0.01011	0.280744	0.83558	

[a] "B", "A", and "D" denote bond length, bond angle, and dihedral angles, respectively. Units for the length and angles are in Å and degrees, respectively. [b]Deviation in total energy between single-point calculation of each basis set from the 6-311+G(2d,p) level calculation. Units are in kcal mol^{-1}.

S2. Exchange-correlation functional for the single-point calculations

In a previous study by Chen et al.,[3] potential energies at the CCSD(T) level were reported for the (Me, Me) complex. This result was used as a reference for the examination. The result is shown in **Table S2**. In the root mean square deviation (RMSD) and maximum absolute deviation (MAD), B3LYP-D3 showed the best result. We also compared the apparent activation energy of the H_2 oxidation (E_a^{ox}) and production (E_a^{red}). The ωB97XD functional overestimated the E_a^{ox} value by 3.3 kcal mol^{-1}, while B3LYP-D3 also overestimated by 3.4 kcal mol^{-1}. For E_a^{red}, the deviation in the ωB97XD and B3LYP-D3 results were 5.3 kcal mol^{-1} and 2.8 kcal mol^{-1}, respectively. These results indicate that the B3LYP-D3 is suitable for the calculation of these Ni complexes. However, for some key results, we also add the ωB97XD results for the reference.

Table S2. Potential energy of the (Me, Me) complex relative to the **^{1}A** intermediate. Comparison of the present ωB97XD//B3LYP-D3 and B3LYP-D3//B3LYP-D3 result with the CCSD(T)[b]//B3P86 one.

Computation	**^{1}TS$_{B1}$**	**^{1}B1**	**^{1}TS$_C$**	**^{1}C**	**^{1}(P+H$_2$)**[c]	E_a^{ox}	E_a^{red}	RMSD[d]	MAD[d]
Present									
B3LYP[a]	12.2	2.2	15.6	4.8	2.7	12.9	15.6	2.7	4.5
B3LYP-D3[a]	12.5	1.7	15.0	3.6	6.4	11.5	15.0	1.9	2.8
ωB97XD[a]	13.2	1.7	17.5	7.7	9.9	11.4	17.5	3.2	5.3
Reference									
CCSD[b]	5.6	-9.5	1.6	-6.3	-7.5	15.1	11.1		
CCSD(T)[b]	10.0	1.9	12.2	5.4	7.2	8.1	12.2		

[a] Basis sets for the single-point calculation and structure optimization were 6-311++G(2d,p) and 6-31G(d,p) sets, respectively.

[b] Reference [3]. For Ni atom, Ahlrichs' triple-VTZ basis set. For C and H atoms, the 3-21G except for P, N, and H atoms in H_2 where the 6-31G** basis sets were given.

[c] Summation of the potential energy of the isolated ^{1}P and $^1H_2$.

[d] RMSD and MAD for the relative potential energy of the five states.

S3. Optimized structures of the Ni^{II} complex in singlet and triplet spin multiplicities

The conformers of the Ni^{II} (H, H) complex were optimized. The structures are given in **Figure S1**, and their potential energy is summarized in **Table S3**. The bidentate ligand has two Ni-P-C-N-C-P rings. The conformer is classified by the number of the chair and boat conformations of the six member rings. For the classification of the conformation, see **Table S3**. The relative stability was also investigated for the Ni^{II} (Ph, Ph) complexes by Raugei and co-workers.[4] We also performed conformation search and found the same trend as seen in **Table S3**. A combination of boat and chair conformations in the same ligand leads to a stable structure. Under this combination, hydrogen bonds between the upper and lower rings stabilizes the conformation. For example, conformer 5S has two bidentate ligands. Each ligand is in the boat and chair conformations in the upper and lower rings, respectively, and this conformation is the most stable among the conformers. In conformer 1S, the left ligands takes the boat-chair combination. However, the boat-boat combination in the right side of 1S induces steric repulsions.

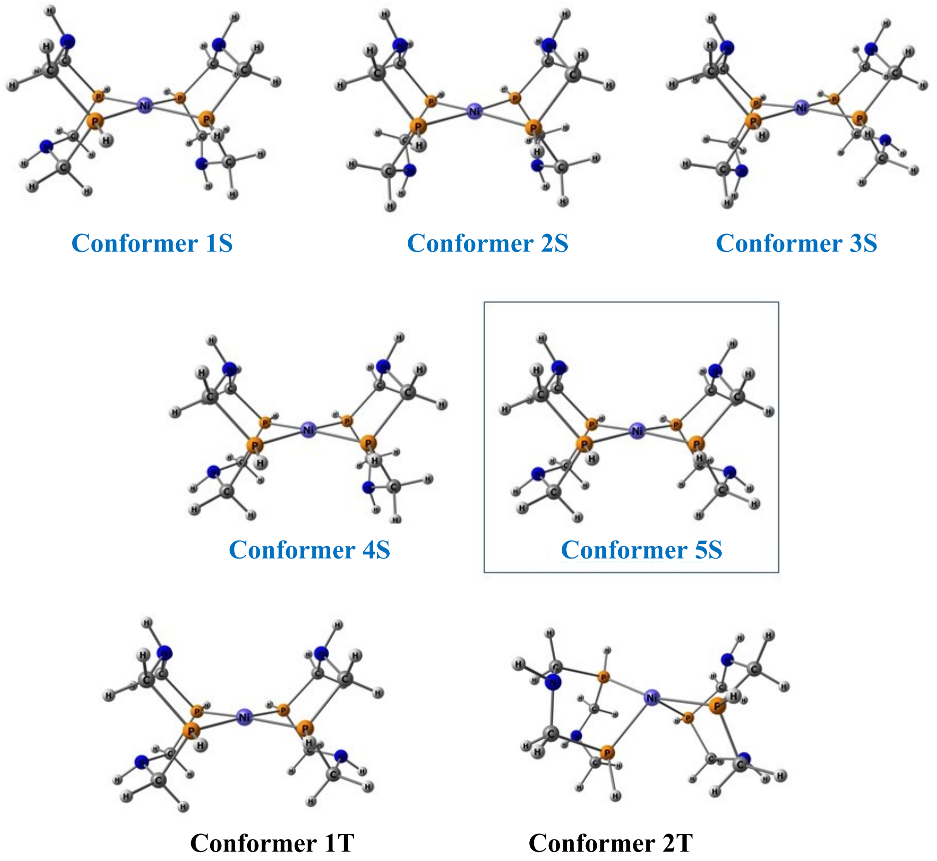

Figure S1. The conformers of the Ni complexes in singlet (S) and triplet (T) states.

Table S3. Relative Energies (kcal/mol) of the Singlet (S) and Triplet (T) Conformers of Bare Active Site for $[Ni^{II}(P_2^HN_2^H)_2]^{2+}$

**Spin states**	**$[Ni^{II}(P_2^HN_2^H)_2]^{2+}$ conformation[a]**	**Energy**	**ΔE kcal mol^{-1}**
Singlet	Conformer 1S (b,c,b,b)	-2074.4615	2.61
	Conformer SS (b,b,b,b)	-2074.4569	5.47
	Conformer 3S (b,b,b,c)	-2074.4615	2.58
	Conformer 4S (b,c,b,b)	-2074.4614	2.64
	**Conformer 5S (b,c,b,c)**	**-2074.4656**	**0.00**
Triplet	Conformer 1T (b,c,b,c), sq	-2074.4010	40.5
	Conformer 2T (b,c,b,c), td	-2074.4475	11.4

[a] Symbols in parenthesis indicate conformation of the six-member ring. "b" and "c" denotes boat and chair conformations, respectively. The four symbols in the parenthesis corresponds the positions of the ring in **Figure S1**: (upper left, lower left, upper right, lower right). "sq" and "td" denote square-planer and tetrahedral conformations, respectively.

Table S4. Relative Gibbs energy (kcal mol^{-1}) of the conformers of the Ni complexes in the ^{1}**A** state.

(R, R')	(b,c,b,c)	(b,b,b,b)
(Me, Me)	0.0	3.7
(NH_2, H)	0.0	6.2
(Cy, Me)	0.0	–0.2
(^{t}Bu, Me)	0.0	–0.0
(CF_3, H)	0.0	0.2
[m3, Me]	0.0	6.3

S4. Structure of the Ni^{II} complexes

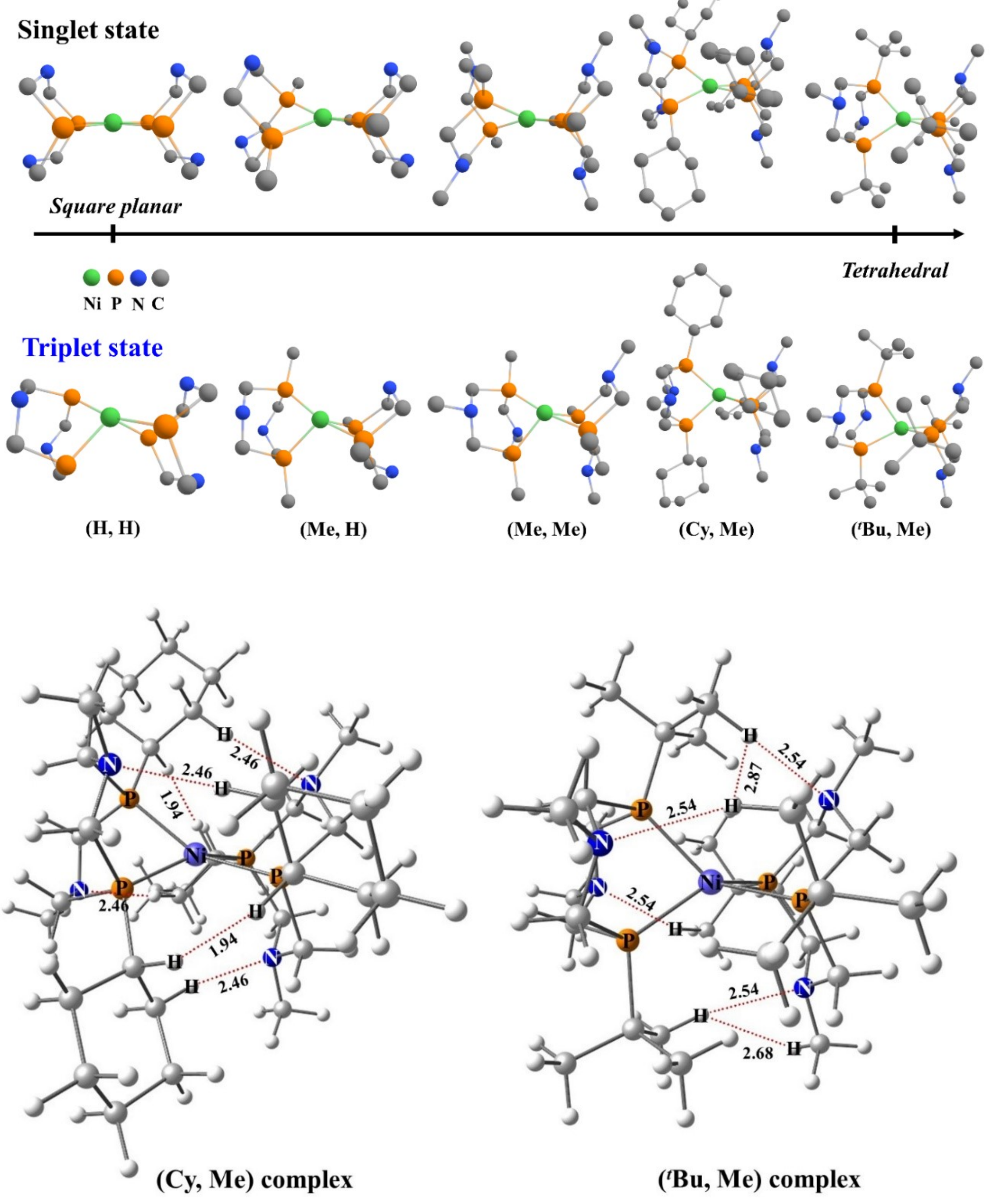

Figure S2. The optimized structure of the Ni^{II} complexes in singlet and triplet states. For the (Cy, Me) and (^{t}Bu, Me) complexes in singlet state, some interatomic distances between the substituent groups are given (distances in Å).

S5. Structure optimization of the complex with Ni^0 and Ni^{II} : Potential energy vs dihedral angles

Potential energy curve against the dihedral angle α was obtained as shown in **Figure 2** and **Figure S3a**. Other structural parameters were minimized. For the definition of α, please refer **Scheme 1c** in the main text. Optimized structure of the Ni complex depends on the oxidation number of the Ni center. The Ni^0 and Ni^{II} prefer tetrahedral and square planer conformations, respectively. Regarding the plot for the Ni^0 complex at the 15 degrees, optimization was ill-converged, and a result with a generous criterion (maximum force 0.02 Hartree/Bohr, root mean square force 0.005 Hartree/Bohr) was given. In this structure, one of the Ni-P bond was nearly dissociated in the optimized structure. Similarly, at the 90 degrees of the Ni^{II} case, the structure was not properly obtained. The result, however, clearly shows the oxidation number dependence in the optimized structure.

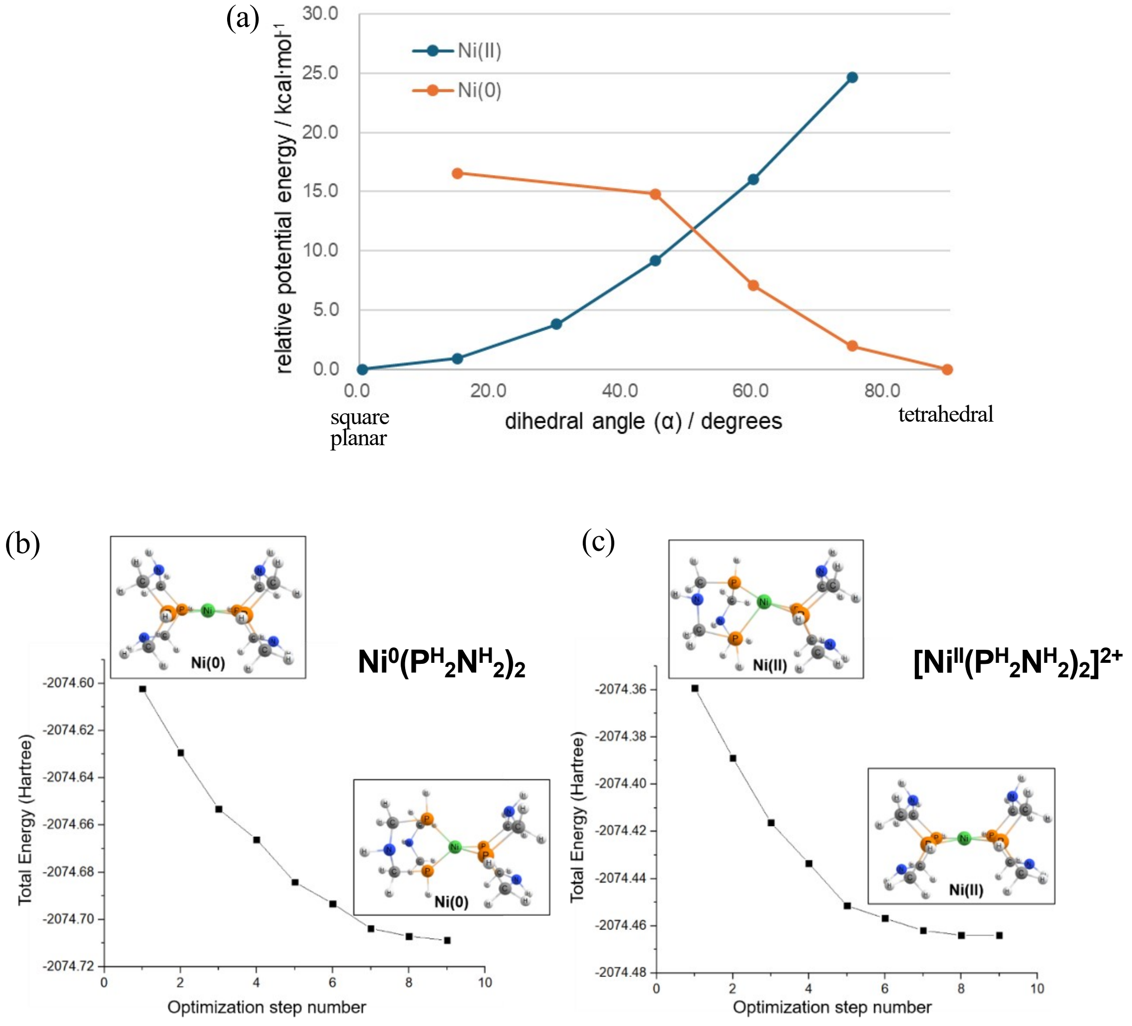

Figure S3. (a) Potential energy curves of Ni^0 and Ni^{II} (H,H) complexes. The optimization profiles of (b) $Ni^0(P_2{}^HN_2{}^H)_2$ and (c) $[Ni^{II}(P_2{}^HN_2{}^H)_2]^{2+}$.

S6. Potential energy of the H_2 production and oxidation catalyzed by the Ni complexes

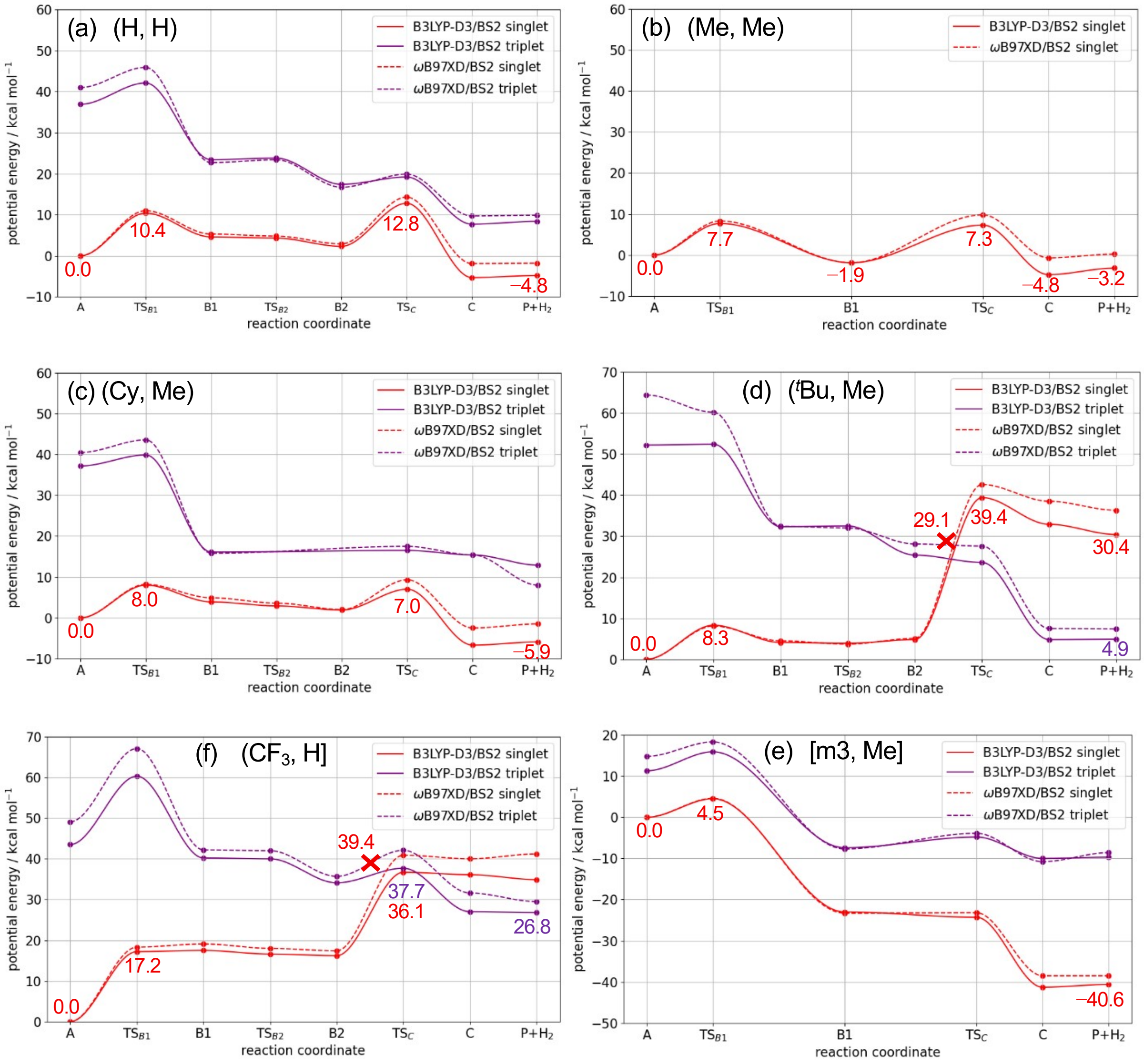

Figure S4. Energy diagram of H_2 oxidation/production in singlet (red) and triplet (purple) states catalyzed by (a) the (H, H), (b) (Me, Me), (c) (Cy, Me), (d) (tBu, Me), (e) (CF_3, H) and (f) [m3, Me] catalysts. The results obtained with B3LYP-D3/BS2//B3LYP-D3/BS1 (solid line) and ωB97XD/BS2//B3LYP-D3/BS1 (dashed line) are given. Zero-point vibrational energy correction is included. The "×" symbol indicates the minimum-energy intersystem crossing point. The energy of the crossing point does not include zero-point energy correction. The numbers in the diagram are the energy relative to the ^{1}A state. The units are in kcal mol^{-1}.

The energy diagram of the H_2 oxidation and production in singlet and triplet states of the (H, H), (Me, Me), (Cy, Me), (^{t}Bu, Me), (CF_3, H) and [m3, Me] catalysts is shown in **Figure S4**. The result is summarized as follows.

- B3LYP-D3/BS2//B3LYP-D3/BS1 and ωB97XD/BS2//B3LYP-D3/BS1 results are close to each other. The energy levels by the B3LYP-D3/BS2 calculation are lower than those by the ωB97XD/BS2 one at the **C** and **P+H_2** states. The CCSD(T) result for the (Me, Me) complex[3] are in the middle of the two functionals as seen in **Table S2**.
- The reaction pathway in triple state lies higher in energy than that in singlet state except for the (^{t}Bu, Me) and (CF_3, H) cases where the singlet state of **TS_C**, **C**, and **P+H_2** becomes higher in energy than the corresponding triplet state. The calculated minimum energy intersystem crossing point for the (^{t}Bu, Me) and (CF_3, H) cases at the B3LYP-D3/BS2//B3LYP-D3/BS1 level was 29.1 kcal mol^{-1} and 39.4 kcal mol^{-1}, respectively.

S7. Structures of intermediate and transition states of the Ni complexes

In this section, the optimized structures of the reactant, intermediate, transition, and product states of the (H, H), (Me, H), (Me, Me), (Cy, Me), (^{t}Bu, Me), [m3, Me], (CF_3, H) and (NH_2, H) catalysts are provided.

(a) (H,H) in singlet state

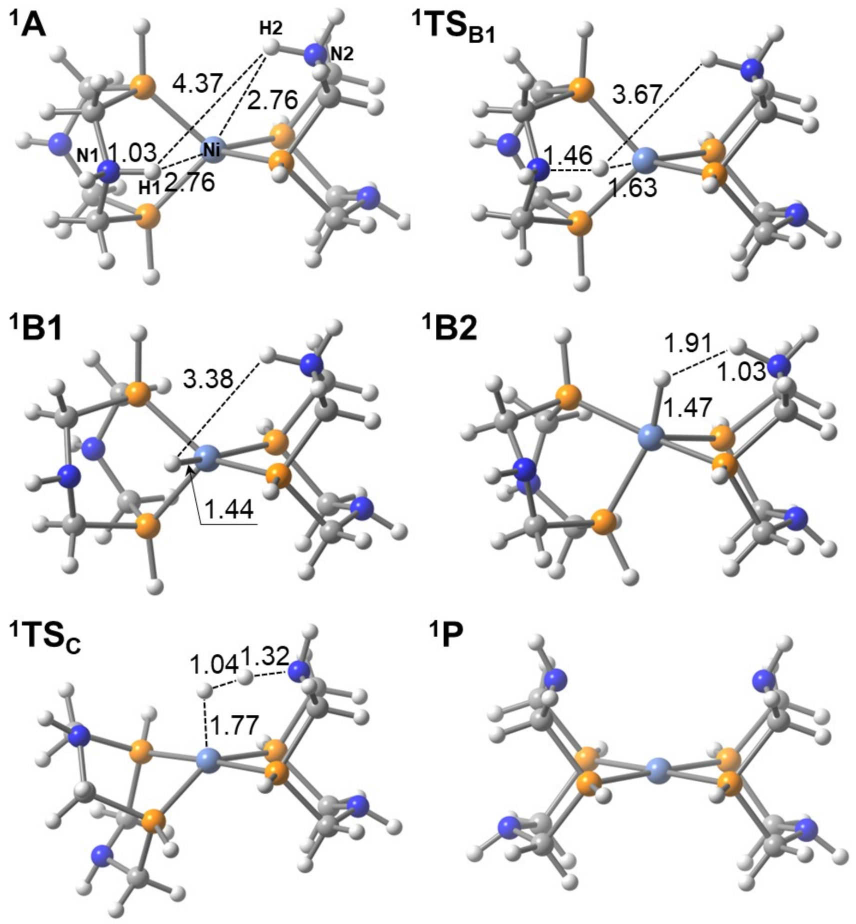

(b) (Me, Me) in singlet state

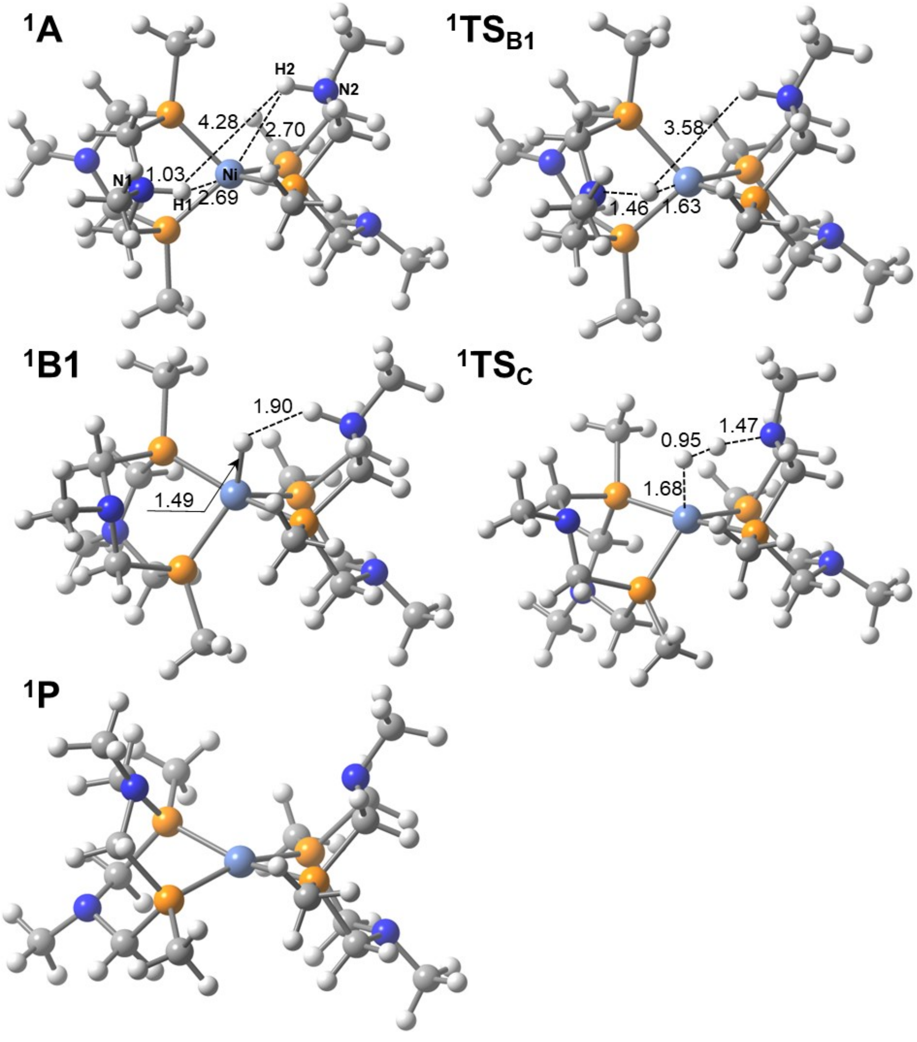

(c) (Cy, Me) in singlet state

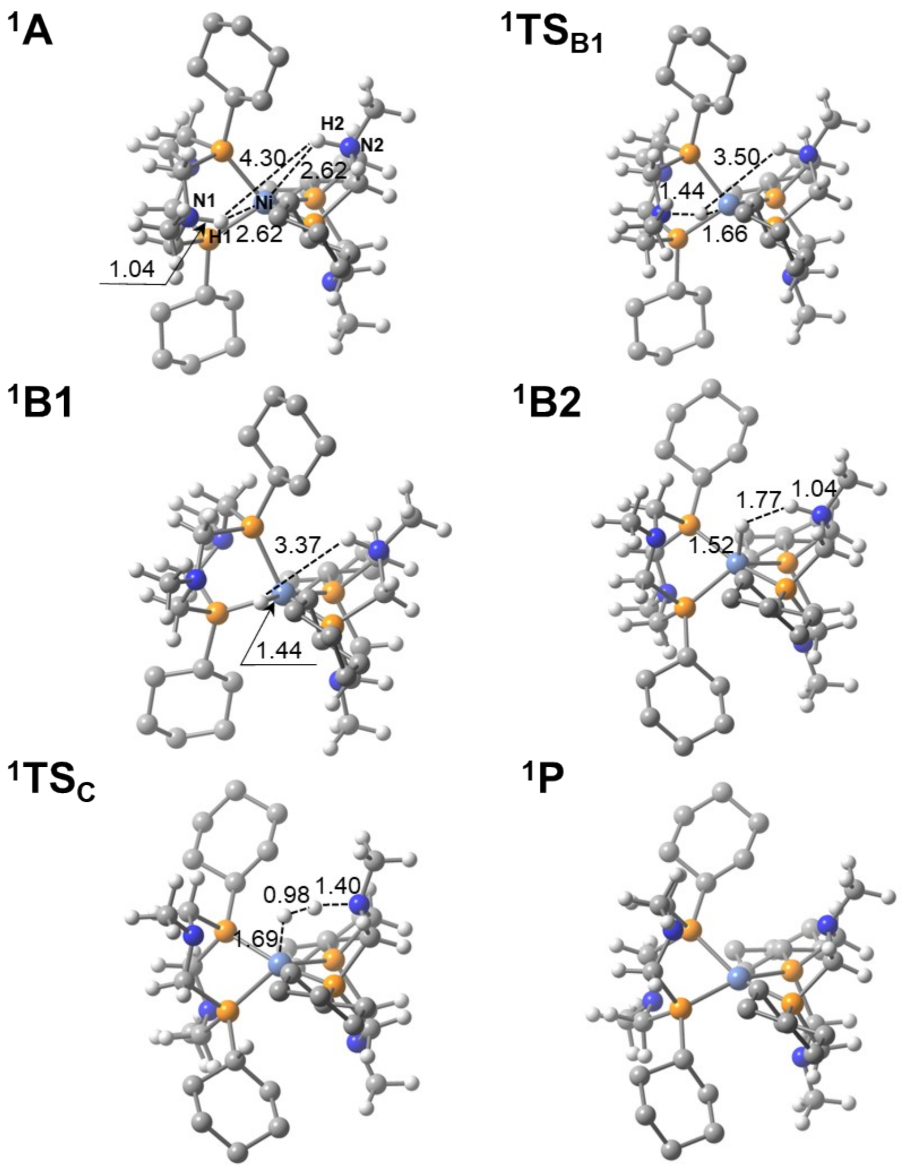

(d) (^{t}Bu, Me) in singlet state

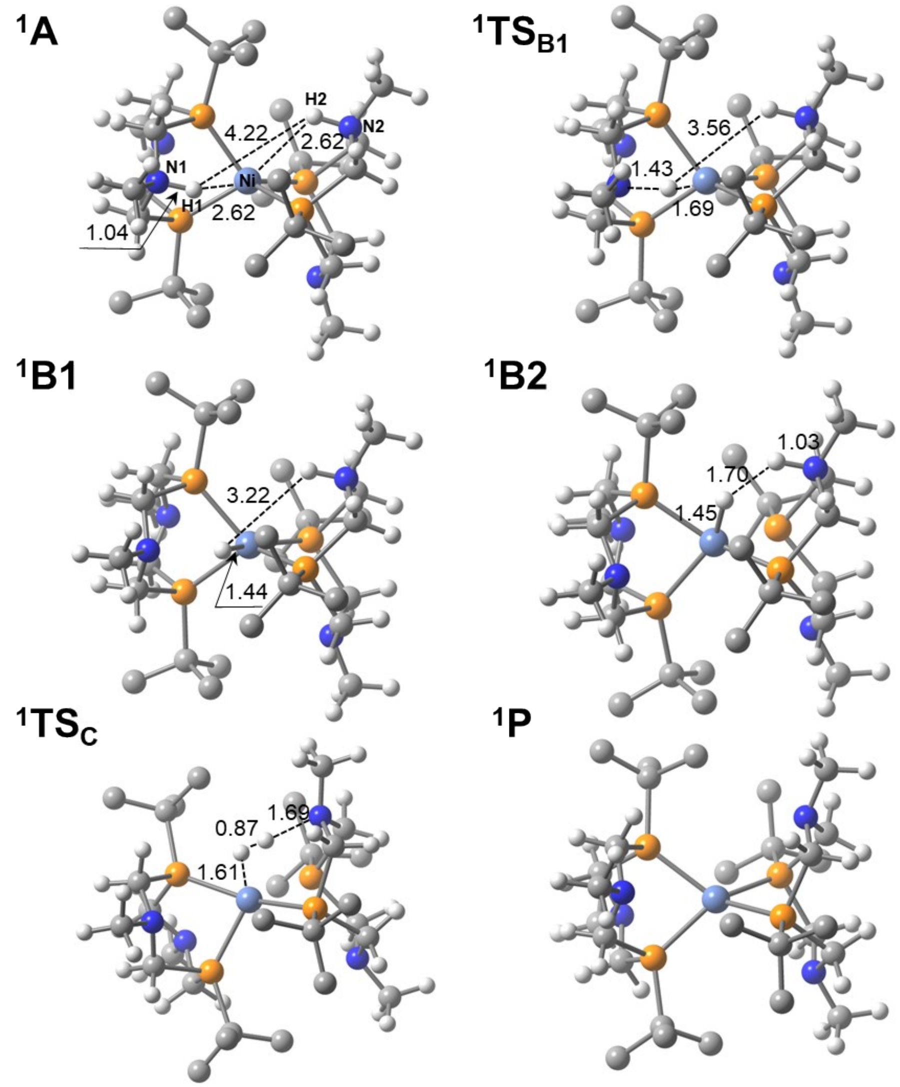

(e) (^{t}Bu, Me) in triplet state

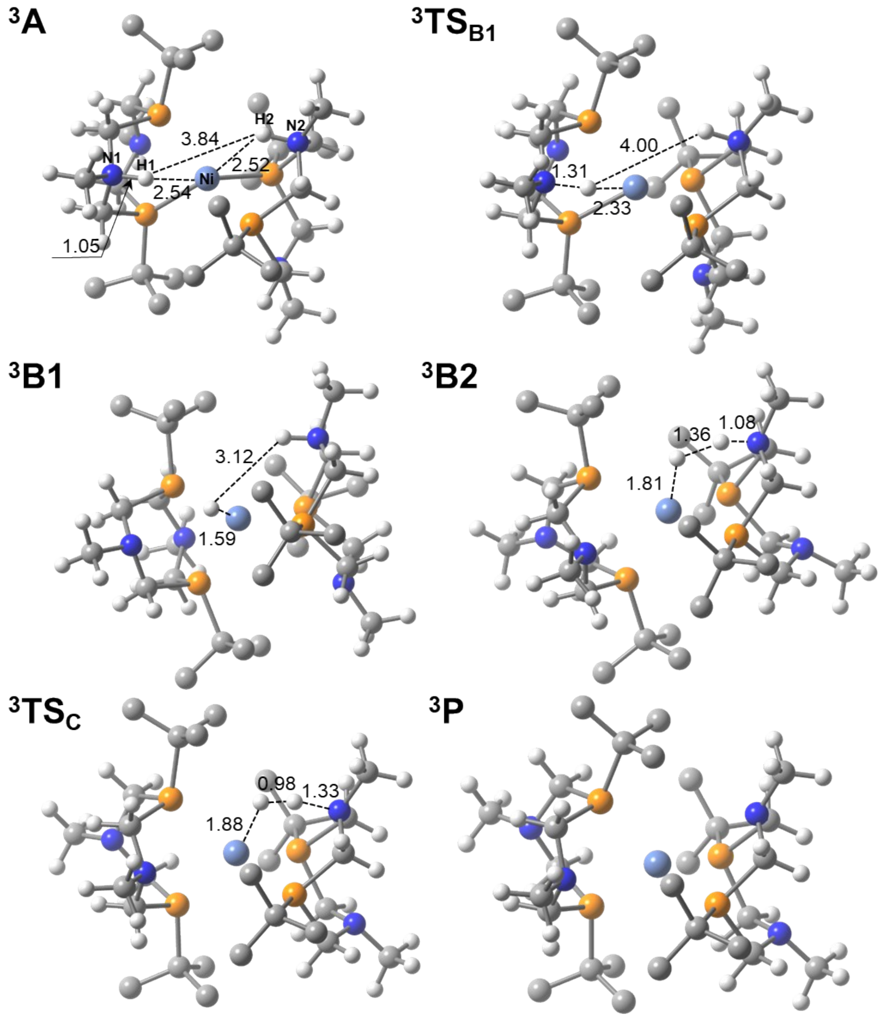

(f) [m3, Me] in singlet state

^{1}A

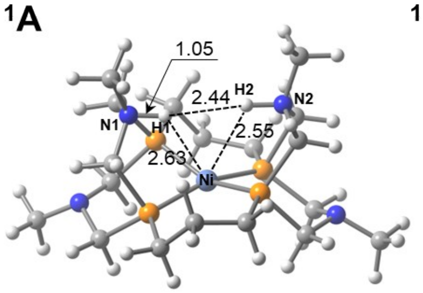

$^{1}TS_{B1}$

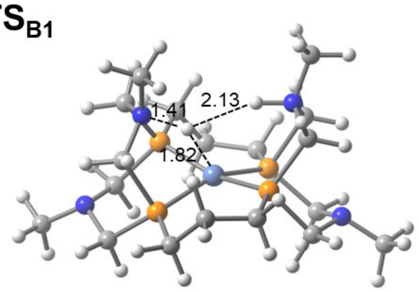

^{1}B1

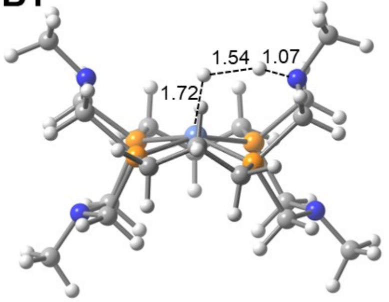

$^{1}TS_{C}$

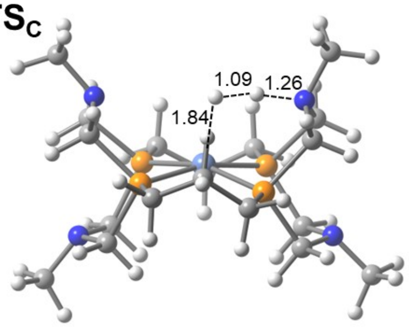

^{1}P

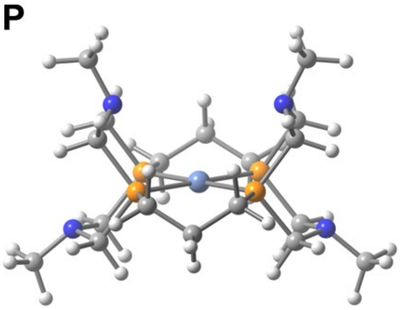

(g) (CF_3, H) in singlet state

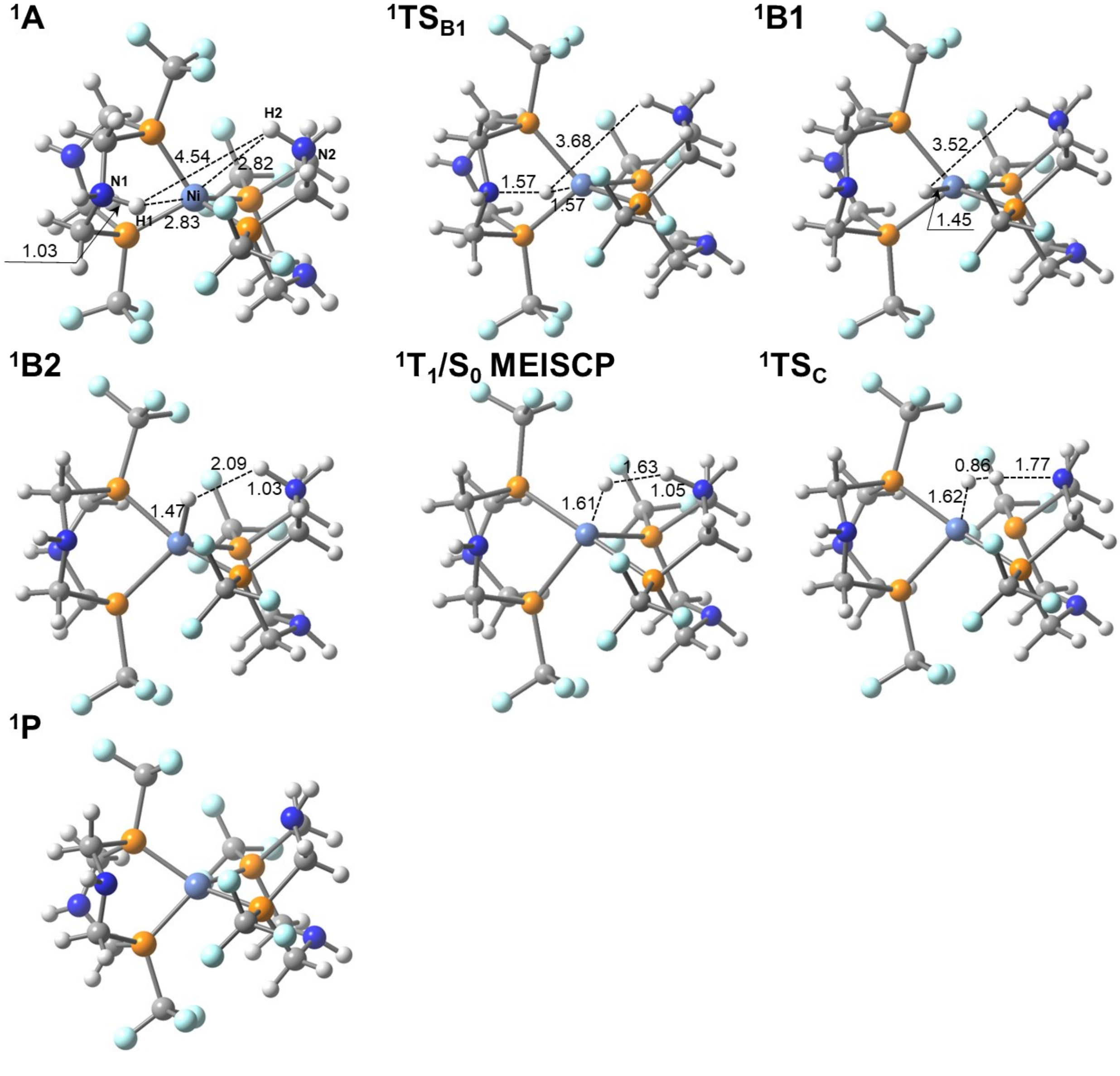

(h) (CF_3, H) in triplet state

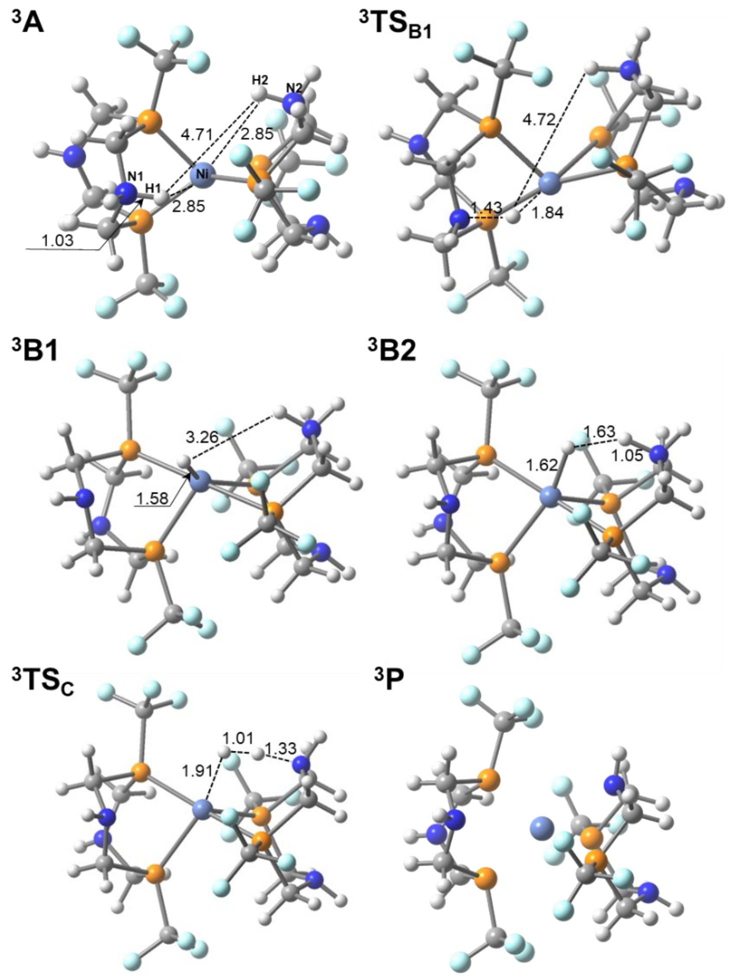

(i) (NH_3, H) in singlet state

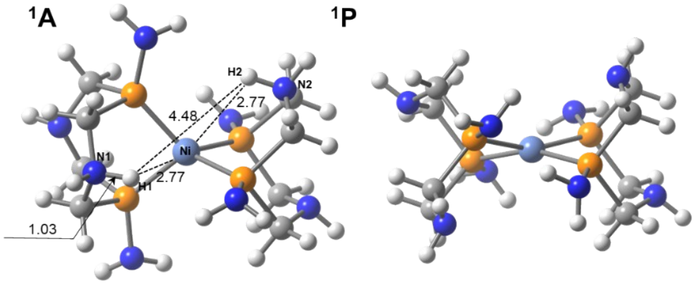

Figure S5. The optimized structures of (a) (H, H) in singlet state, (b) (Me, H) in singlet state, (c) (Me, Me) in singlet state, (e) (Cy, Me) in singlet state, (g) (^{t}Bu, Me) in singlet and (d) triplet states, (f) [m3, Me] in singlet state, (g) (CF_3, H) in singlet and (h) triplet states and (i) (NH_2, H) in singlet state. Distances are in Å units.

S8. Singlet-triplet spin-orbit coupling at the intersystem crossing point in the potential energy surface of (^{t}Bu, Me) complex

As shown in **Figure 3** and **Figure S4**, the reaction pathway of the H_2 oxidation catalyzed by the (^{t}Bu, Me) and (CF_3, H) complexes involve intersystem crossing between singlet and triplet states. During the dissociative adsorption of H_2 to the Ni complex (from **C** to **B2** states), triplet to singlet transition is expected to happen after a transition state (**^{3}TS$_C$**). This point was located at 29.1 kcal mol^{-1} and 36.1 kcal mol^{-1} from **^{1}A** state in the (^{t}Bu, Me) and (CF_3, H) complexes, respectively, with the help of the GRRM23 program.[5,6] This minimum energy intersystem crossing point approximates the transition state for the H_2 oxidation obtained with the relativistic quantum chemical calculation, and the activation energy from the **^{3}(P+H$_2$)** state is 24.2 kcal mol^{-1} and 12.6 kcal mol^{-1}, respectively.

We also evaluated spin-orbit coupling (SOC) at the crossing point. As the Kohn-Sham DFT calculation is employed in this study, SOC is evaluated in the same way as in the wave function theory such as $\langle {}^1\Psi_{DFT} | \hat{H}_{SO} | {}^3\Psi_{DFT} \rangle$. Single point calculations with B3LYP-D3/6-31G** at the optimized geometry were performed for obtaining the ${}^1\Psi_{DFT}$ and ${}^3\Psi_{DFT}$. In these DFT calculations, effective core potential (ECP) was not adopted because the combined use of the ECP in the SOC calculation has not been verified yet. We used the MolSOC program[7,8] in which the Breit-Pauli SOC operator is adopted. The result is given in **Table S5**. The $|\hat{H}_{SO}|$ value is averaged over the triply degenerated M_S states and calculated to be 44.9 cm^{-1} and 26.1 cm^{-1}, respectively, which are not a large value but enough to verify the reaction pathway involving the intersystem crossing.

Table S5. One- and two-electron spin-orbit coupling matrix elements (cm^{-1}) for the (a) (^{t}Bu, Me) and (b) (CF_3, Me) complexes calculated using the B3LYP-D3 functional and the 6-31G(d,p) basis set for all atoms.

${}^1\Psi_{DFT}$	${}^3\Psi_{DFT}$	1-electron term		2-electron term		total		$\lvert\hat{H}_{SO}\rvert$
M_S	M_S	real	imaginary	real	imaginary	real	imaginary	
(a) (^{t}Bu, Me) complex								
0	0	73.3170	0.0000	-30.7934	0.0000	42.5236	0.0000	42.5236
0	1	-11.0713	-80.8073	2.3800	35.5025	-8.6913	-45.3048	46.1309
0	-1	11.0713	-80.8073	-2.3800	35.5025	8.6913	-45.3048	46.1309
(b) (CF_3, Me) complex								
0	0	54.9989	0.0000	-23.9853	0.0000	31.0135	0.0000	31.0135
0	1	-46.8776	1.5897	23.2511	-0.3805	-23.6264	1.2092	23.6573
0	-1	46.8776	1.5897	-23.2511	-0.3805	23.6264	1.2092	23.6573

S9. Reaction energy and change of dihedral angles in the H_2 production

The following table is the numerical data for **Figure 6** in the main text.

Table S6. Reaction energy (potential energy with zero-point vibrational energy correction) and change of dihedral angle in the A and P+H_2 states of the H_2 production.

Complexes	ΔE / kcal mol^{-1}	dihedral angle / degrees	
		**A**	**P+H_2**
(H,H)	-4.8	89.9	1.5
(Me, Me)	-3.2	89.2	25.2
(NH_2, H)	-1.3	89.7	28.6
(Cy, Me)	-5.9	84.8	37.0
(^{t}Bu, Me)[a]	4.9	89.5	89.0
(CF_3, H)[a]	26.8	88.6	79.2
[m3, Me]	-40.6	31.7	0.36

[a] For the (^{t}Bu, Me) and (CF_3, H) complexes, the pathway via intersystem crossing was adopted. The reaction energy is defined as the difference between the triplet **P+H_2** state and the singlet **A** state.

S10. Summary of reaction energy and activation energy for the H_2 production and oxidation

Table S7. Reaction energy and apparent activation energy (potential energy with zero-point vibrational energy correction) of the H_2 production and oxidation of the Ni complexes. Units are in kcal mol^{-1}.

Complexes	H_2 production		H_2 oxidation	
	ΔE	E_a	ΔE	E_a
(H,H)	−4.8	12.8	4.8	17.6
(Me, Me)	−3.2	7.7	3.2	12.5
(NH_2, H)	−1.3			
(Cy, Me)	−5.9	8.0	5.9	13.9
(^{t}Bu, Me)[a]	4.9	29.1	−4.9	24.2
(CF_3, H)[a]	26.8	39.4	−26.8	12.6
[m3, Me]	−40.6	4.5	40.6	45.1

[a] For the (^{t}Bu, Me) and (CF_3, H) complexes, the pathway via intersystem crossing was adopted. The reaction energy is defined as the difference between the triplet **P+H$_2$** state and the singlet **A** state.

S11. Atomic coordinates

(H, H) complex in the singlet spin state

Ni	0.000000000	0.000097000	0.057031000
P	1.653421000	-1.481938000	-0.012921000
P	1.653407000	1.481878000	-0.018459000
N	2.564622000	0.003947000	2.101463000
N	3.393458000	-0.002745000	-1.468134000
C	2.870868000	-1.232744000	1.383163000
H	2.749119000	-2.074216000	2.068098000
H	3.881151000	-1.267402000	0.948908000
C	2.870866000	1.237939000	1.378536000
H	3.881148000	1.270973000	0.944154000
H	2.749111000	2.081972000	2.060313000
C	2.640407000	-1.242400000	-1.582639000
H	3.339941000	-2.076320000	-1.674433000
H	1.937352000	-1.277887000	-2.429184000
C	2.640362000	1.236444000	-1.587288000
H	1.937284000	1.268716000	-2.433943000
H	3.339865000	2.070035000	-1.682246000
P	-1.653411000	-1.481940000	-0.013193000
P	-1.653417000	1.481877000	-0.018183000
N	-3.393456000	-0.002475000	-1.468133000
N	-2.564624000	0.003524000	2.101465000
C	-2.640360000	-1.242079000	-1.582888000
H	-1.937277000	-1.277355000	-2.429420000
H	-3.339861000	-2.076004000	-1.674891000
C	-2.640406000	1.236766000	-1.587039000
H	-3.339942000	2.070353000	-1.681792000
H	-1.937354000	1.269247000	-2.433707000
C	-2.870870000	-1.233031000	1.382929000
H	-3.881153000	-1.267609000	0.948671000
H	-2.749115000	-2.074633000	2.067702000
C	-2.870867000	1.237652000	1.378771000
H	-2.749120000	2.081556000	2.060711000
H	-3.881148000	1.270765000	0.944391000
H	-1.344753000	2.853037000	-0.015915000
H	1.344762000	-2.853081000	-0.005785000
H	3.060944000	0.005609000	2.988257000
H	-1.344748000	-2.853085000	-0.006350000
H	4.189052000	-0.003908000	-2.095949000
H	-4.189051000	-0.003539000	-2.095947000
H	-3.060947000	0.005021000	2.988259000
H	1.344741000	2.853038000	-0.016488000

(H, H) complex in the triplet spin state

Ni	-0.001262000	0.086020000	-0.566027000
P	1.951680000	1.418338000	-0.669788000
P	1.421118000	-1.387297000	0.644369000
N	2.714002000	-0.979823000	-1.744788000
N	3.245971000	0.438806000	1.526405000
C	3.218103000	0.387812000	-1.584407000
H	3.332080000	0.829162000	-2.576786000
H	4.176692000	0.454184000	-1.051765000
C	2.781999000	-1.840849000	-0.560361000
H	3.742477000	-1.797239000	-0.027971000
H	2.598259000	-2.870212000	-0.874652000
C	2.695083000	1.690746000	1.031305000
H	3.506987000	2.413798000	0.928072000
H	1.918493000	2.134038000	1.673522000
C	2.315125000	-0.543033000	2.060447000
H	1.560616000	-0.132060000	2.748620000
H	2.883890000	-1.307232000	2.595225000
P	-1.412129000	1.122435000	1.042112000
P	-1.961444000	-1.132461000	-1.073337000
N	-3.226552000	-0.895511000	1.333726000
N	-2.740428000	1.479272000	-1.335435000
C	-2.269732000	-0.142583000	2.128958000
H	-1.493553000	-0.757671000	2.609876000
H	-2.810543000	0.398101000	2.908906000
C	-2.702153000	-1.930007000	0.455119000
H	-3.528635000	-2.566441000	0.132188000
H	-1.931807000	-2.569608000	0.912523000
C	-2.791994000	1.921719000	0.060597000
H	-3.743545000	1.706963000	0.566770000
H	-2.614191000	2.998835000	0.084570000
C	-3.236877000	0.128102000	-1.609574000
H	-3.357381000	0.020400000	-2.689542000
H	-4.191216000	-0.109907000	-1.119312000
H	-2.017599000	-2.154713000	-2.039681000
H	2.002105000	2.693095000	-1.264234000
H	3.203952000	-1.429393000	-2.511950000
H	-0.992828000	2.110706000	1.952581000
H	3.997517000	0.613049000	2.183084000
H	-3.966899000	-1.264354000	1.918673000
H	-3.243780000	2.144750000	-1.914381000
H	1.007605000	-2.612676000	1.202226000

(Me, H) complex in the singlet spin state

Ni	0.000005000	0.000000000	0.031047000
P	-1.707179000	1.436504000	0.344660000
P	-1.649282000	-1.476822000	-0.398472000
N	-2.554139000	-0.618087000	2.025861000
N	-3.462095000	0.327557000	-1.413859000
C	-2.852418000	0.764773000	1.668612000
H	-2.691848000	1.389197000	2.550939000
H	-3.880782000	0.924937000	1.306903000
C	-2.882720000	-1.597499000	0.991415000
H	-3.890723000	-1.482904000	0.564951000
H	-2.788564000	-2.600352000	1.414728000
C	-2.784062000	1.589830000	-1.166541000
H	-3.530877000	2.363854000	-0.967939000
H	-2.141201000	1.932791000	-1.994326000
C	-2.612797000	-0.772105000	-1.844959000
H	-1.883772000	-0.505714000	-2.627669000
H	-3.247442000	-1.575355000	-2.227914000
P	1.649255000	1.476851000	-0.398442000
P	1.707222000	-1.436467000	0.344700000
N	3.461724000	-0.327623000	-1.414266000
N	2.554623000	0.618080000	2.025689000
C	2.612356000	0.772055000	-1.845176000
H	1.883092000	0.505657000	-2.627661000
H	3.246926000	1.575259000	-2.228351000
C	2.783750000	-1.589883000	-1.166759000
H	3.530610000	-2.363900000	-0.968299000
H	2.140692000	-1.932875000	-1.994378000
C	2.883013000	1.597466000	0.991154000
H	3.890911000	1.482815000	0.564460000
H	2.789002000	2.600329000	1.414476000
C	2.852821000	-0.764776000	1.668347000
H	2.692518000	-1.389210000	2.550714000
H	3.881086000	-0.924903000	1.306342000
H	-3.053778000	-0.852643000	2.879320000
H	-4.226393000	0.459154000	-2.067708000
H	4.225898000	-0.459220000	-2.068257000
H	3.054467000	0.852628000	2.879031000
C	-1.473881000	3.180178000	0.870386000
H	-0.715521000	3.244000000	1.652951000
H	-1.181877000	3.794221000	0.018621000
H	-2.426216000	3.552468000	1.258077000
C	1.367541000	3.209504000	-0.938498000
H	0.641572000	3.236806000	-1.753713000
H	1.009952000	3.812537000	-0.105008000
H	2.316292000	3.623766000	-1.290503000
C	1.473958000	-3.180109000	0.870527000
H	0.715834000	-3.243860000	1.653321000
H	1.181658000	-3.794157000	0.018872000
H	2.426395000	-3.552463000	1.257910000
C	-1.367683000	-3.209480000	-0.938583000
H	-0.641816000	-3.236849000	-1.753878000

H	-1.010049000	-3.812519000	-0.105117000
H	-2.316503000	-3.623702000	-1.290453000

(Me, H) complex in the triplet spin state

Ni	-0.017156000	-0.194189000	-0.510565000
P	-1.643246000	1.445614000	-0.705009000
P	-1.639818000	-1.477667000	0.513080000
N	-2.074803000	0.853851000	1.979158000
N	-2.855657000	-0.784879000	-1.958240000
C	-2.507149000	1.780435000	0.942368000
H	-2.194945000	2.786546000	1.237589000
H	-3.600889000	1.803376000	0.787383000
C	-2.554968000	-0.514175000	1.862815000
H	-3.643971000	-0.596602000	1.695530000
H	-2.325267000	-1.034229000	2.797141000
C	-3.049777000	0.684285000	-1.721347000
H	-4.026198000	0.830788000	-1.256434000
H	-3.048629000	1.154185000	-2.706831000
C	-3.009086000	-1.698352000	-0.780393000
H	-2.935605000	-2.714870000	-1.172563000
H	-4.002215000	-1.546956000	-0.353945000
C	-1.626196000	3.132446000	-1.459775000
C	-1.680277000	-3.193401000	1.208228000
P	2.068627000	-0.552561000	-1.335679000
P	1.302764000	0.706401000	1.444034000
N	2.273461000	-1.943509000	1.080803000
N	2.607395000	2.060373000	-0.623359000
C	3.016327000	-1.749626000	-0.218998000
H	3.071501000	-2.730323000	-0.693369000
H	4.025441000	-1.412110000	0.020705000
C	2.298042000	-0.782611000	2.045395000
H	3.344095000	-0.548379000	2.251055000
H	1.831218000	-1.145145000	2.963166000
C	3.184024000	0.962880000	-1.390046000
H	4.199754000	0.674508000	-1.069835000
H	3.241911000	1.267350000	-2.438546000
C	2.637934000	1.890628000	0.824750000
H	2.397133000	2.854398000	1.282077000
H	3.620594000	1.577853000	1.217804000
C	2.389552000	-1.330130000	-2.974121000
C	0.936285000	1.439639000	3.104298000
H	-1.248767000	-3.886155000	0.481640000
H	-2.701305000	-3.507033000	1.446036000
H	-1.078574000	-3.214876000	2.120368000
H	-0.954176000	3.763388000	-0.872805000
H	-2.623737000	3.581431000	-1.475516000
H	-1.241496000	3.062585000	-2.480068000
H	1.879881000	-2.296312000	-3.011554000
H	3.460711000	-1.463275000	-3.155381000
H	1.962484000	-0.682200000	-3.744021000
H	0.423576000	2.390742000	2.944022000
H	1.846095000	1.607232000	3.688662000
H	0.259165000	0.773759000	3.641472000
H	-1.845448000	-0.916780000	-2.329434000
H	1.286707000	-2.141476000	0.850477000
H	-3.490855000	-1.091726000	-2.701775000
H	-2.315738000	1.225569000	2.892079000
H	2.642285000	-2.773310000	1.555914000
H	3.079673000	2.925572000	-0.869184000

(Me, Me) complex in the singlet spin state

Ni	-0.000031000	-0.000073000	-0.000030000
P	1.698071000	0.329479000	-1.437820000
P	1.698001000	-0.329638000	1.437836000
N	2.442838000	-2.164637000	-0.540015000
N	2.442890000	2.164434000	0.540037000
C	2.920304000	-1.088960000	-1.403877000
H	2.978717000	-1.460867000	-2.429354000
H	3.922941000	-0.714204000	-1.133495000
C	2.607547000	-1.861216000	0.876447000
H	3.666637000	-1.747818000	1.175617000
H	2.185255000	-2.683337000	1.461158000
C	2.607713000	1.860995000	-0.876412000
H	3.666827000	1.747511000	-1.175469000
H	2.185539000	2.683144000	-1.461166000
C	2.920293000	1.088746000	1.403930000
H	2.978684000	1.460657000	2.429406000
H	3.922923000	0.713960000	1.133577000
C	1.517933000	0.692058000	-3.228168000
C	1.517882000	-0.692181000	3.228190000
C	3.072550000	-3.449170000	-0.871055000
H	2.636758000	-4.234424000	-0.247708000
H	4.163741000	-3.442143000	-0.711586000
C	3.072480000	3.449007000	0.871142000
H	2.636696000	4.234228000	0.247748000
H	4.163688000	3.442053000	0.711789000
P	-1.697854000	0.329652000	1.437775000
P	-1.698030000	-0.329628000	-1.437822000
N	-2.443283000	-2.164372000	0.540174000
N	-2.442673000	2.164599000	-0.540191000
C	-2.920432000	-1.088472000	1.403945000
H	-2.978957000	-1.460247000	2.429466000
H	-3.922977000	-0.713492000	1.133541000
C	-2.607749000	-1.861066000	-0.876354000
H	-3.666793000	-1.747624000	-1.175688000
H	-2.185431000	-2.683276000	-1.460922000
C	-2.607209000	1.861336000	0.876347000
H	-3.666267000	1.748159000	1.175719000
H	-2.184651000	2.683440000	1.460887000
C	-2.920197000	1.088870000	-1.403961000
H	-2.978613000	1.460691000	-2.429470000
H	-3.922837000	0.714181000	-1.133542000
C	-1.517649000	0.692218000	3.228115000
C	-1.517725000	-0.692234000	-3.228148000
C	-3.073429000	-3.448676000	0.871266000
H	-2.637856000	-4.234109000	0.247995000
H	-4.164607000	-3.441289000	0.711732000
C	-3.072346000	3.449130000	-0.871279000
H	-2.636459000	4.234407000	-0.248027000
H	-4.163525000	3.442173000	-0.711723000
H	-2.873214000	3.691262000	-1.918305000
H	-2.874356000	-3.690891000	1.918283000
H	2.873143000	3.691215000	1.918111000
H	2.873344000	-3.691395000	-1.918044000
H	1.189136000	0.198139000	3.763778000
H	2.491203000	-1.002696000	3.619425000
H	0.799777000	-1.501297000	3.373460000
H	1.189374000	-0.198293000	-3.763804000
H	2.491184000	1.002806000	-3.619393000
H	0.799642000	1.501024000	-3.373376000
H	-1.189024000	-0.198139000	3.763711000
H	-2.490917000	1.002886000	3.619362000
H	-0.799425000	1.501233000	3.373358000
H	-1.189070000	0.198121000	-3.763726000
H	-2.490935000	-1.002931000	-3.619512000
H	-0.799455000	-1.501227000	-3.373300000

(Me, Me) complex in the triplet spin state

Ni	0.002644000	0.613377000	0.032048000
P	1.406172000	-0.682692000	-1.344129000
P	1.942789000	0.819161000	1.322367000
N	1.968188000	-1.978792000	1.068150000
N	2.724969000	1.766657000	-1.152869000
C	2.171905000	-2.081736000	-0.375815000
H	1.662955000	-2.985050000	-0.724080000
H	3.236503000	-2.172694000	-0.658677000
C	2.680800000	-0.864494000	1.692985000
H	3.757113000	-0.843810000	1.445577000
H	2.602544000	-0.980500000	2.777174000
C	2.803898000	0.470278000	-1.831071000
H	3.777029000	-0.032710000	-1.694249000
H	2.674825000	0.641109000	-2.902731000
C	3.169539000	1.719058000	0.241859000
H	3.202336000	2.742189000	0.625796000

H	4.177592000	1.285125000	0.363209000
C	0.961951000	-1.477437000	-2.936181000
C	2.032101000	1.695215000	2.933257000
C	2.325499000	-3.246565000	1.719651000
H	2.108939000	-3.181246000	2.789376000
H	3.390338000	-3.505804000	1.596001000
C	3.455140000	2.799848000	-1.900270000
H	3.049154000	2.869490000	-2.912801000
H	4.535942000	2.592466000	-1.969286000
P	-1.934236000	0.946619000	-1.231057000
P	-1.401488000	-0.809873000	1.270876000
N	-2.731935000	1.646184000	1.323676000
N	-1.968940000	-1.859739000	-1.263484000
C	-3.226799000	1.615554000	-0.055761000
H	-3.414602000	2.643382000	-0.376382000
H	-4.172613000	1.057739000	-0.166732000
C	-2.717452000	0.327590000	1.962271000
H	-3.700336000	-0.175938000	1.933623000
H	-2.449979000	0.465692000	3.013484000
C	-2.585830000	-0.682572000	-1.875320000
H	-3.687507000	-0.678196000	-1.790019000
H	-2.344553000	-0.691382000	-2.942248000
C	-2.281327000	-2.028888000	0.155427000
H	-1.917275000	-3.012469000	0.463582000
H	-3.364957000	-2.001500000	0.367867000
C	-2.018570000	2.051827000	-2.694245000
C	-0.956703000	-1.825086000	2.732003000
C	-3.495125000	2.605775000	2.134307000
H	-3.059424000	2.661760000	3.135317000
H	-4.558473000	2.329650000	2.230786000
C	-2.339321000	-3.069547000	-2.011786000
H	-2.053962000	-2.952652000	-3.060901000
H	-3.420751000	-3.282057000	-1.970318000
H	-1.799576000	-3.928143000	-1.603265000
H	-3.433394000	3.596951000	1.677447000
H	3.317989000	3.766789000	-1.409099000
H	1.721704000	-4.053933000	1.296008000
H	1.690765000	2.723715000	2.796660000
H	3.055872000	1.695468000	3.317802000
H	1.374332000	1.193622000	3.646503000
H	0.197716000	-2.229637000	-2.743378000
H	1.841868000	-1.941017000	-3.390896000
H	0.560112000	-0.720672000	-3.613589000
H	-1.758876000	3.066684000	-2.383866000
H	-3.021428000	2.046911000	-3.130773000
H	-1.293209000	1.711534000	-3.436988000
H	-0.261069000	-2.601480000	2.415664000
H	-1.852539000	-2.274733000	3.169785000
H	-0.467905000	-1.190544000	3.474234000

(Cy, Me) complex in the singlet spin state

Ni	0.000038000	-0.000033000	0.000004000
P	-1.384353000	-0.495776000	-1.701808000
P	1.384366000	0.497348000	-1.701474000
N	0.830965000	-2.046470000	-2.490754000
N	-0.830975000	2.048664000	-2.489202000
C	-0.500118000	-1.633444000	-2.927185000
H	-1.124078000	-2.523901000	-3.012161000
H	-0.501453000	-1.149382000	-3.918515000
C	1.839087000	-1.005461000	-2.689267000
H	1.975786000	-0.730623000	-3.750034000
H	2.802515000	-1.365089000	-2.316874000
C	-1.839102000	1.007802000	-2.688430000
H	-1.975852000	0.733766000	-3.749399000
H	-2.802519000	1.367139000	-2.315737000
C	0.500079000	1.635910000	-2.925982000
H	1.124069000	2.526398000	-3.010383000
H	0.501333000	1.152546000	-3.917653000
C	-2.945715000	-1.411798000	-1.296612000
H	-3.493701000	-0.733888000	-0.632412000
C	-3.856368000	-1.714196000	-2.503048000
H	-4.112287000	-0.791888000	-3.035437000
H	-3.332682000	-2.363773000	-3.215575000
C	-5.137584000	-2.421594000	-2.023414000
H	-5.713523000	-1.727273000	-1.396432000
H	-5.765051000	-2.662409000	-2.888576000
C	-4.820009000	-3.691008000	-1.218800000
H	-5.747813000	-4.152677000	-0.863063000
H	-4.332890000	-4.423392000	-1.877440000
C	-3.889244000	-3.386593000	-0.035676000
H	-3.634760000	-4.307783000	0.500397000
H	-4.409097000	-2.734770000	0.680574000
C	-2.602042000	-2.693320000	-0.507815000
H	-2.046566000	-3.384443000	-1.155586000
H	-1.943471000	-2.465446000	0.336936000
C	2.945697000	1.413145000	-1.295643000
H	3.493875000	0.734745000	-0.632107000
C	2.601977000	2.693953000	-0.505711000
H	2.046290000	3.385544000	-1.152798000
H	1.943615000	2.465242000	0.338974000
C	3.889185000	3.387001000	-0.033233000
H	3.634686000	4.307703000	0.503668000
H	4.409256000	2.734629000	0.682357000
C	4.819700000	3.692524000	-1.216266000
H	5.747486000	4.154062000	-0.860311000
H	4.332351000	4.425349000	-1.874244000
C	5.137355000	2.423799000	-2.021931000
H	5.713466000	1.729060000	-1.395570000
H	5.764680000	2.665394000	-2.886978000
C	3.856158000	1.716632000	-2.501958000
H	3.332327000	2.366731000	-3.213896000
H	4.112103000	0.794775000	-3.035117000
C	1.246870000	-3.307546000	-3.115937000
H	2.213807000	-3.608887000	-2.703804000
H	1.341959000	-3.230798000	-4.211292000
C	-1.246887000	3.310191000	-3.113454000
H	-2.213824000	3.611218000	-2.701091000
H	-1.341986000	3.234260000	-4.208867000
P	-1.384393000	0.495690000	1.701834000
P	1.384329000	-0.497431000	1.701458000
N	0.830893000	2.046322000	2.491031000
N	-0.831029000	-2.048818000	2.488954000
C	-0.500185000	1.633191000	2.927381000
H	-1.124170000	2.523611000	3.012542000
H	-0.501504000	1.148962000	3.918629000
C	1.839030000	1.005302000	2.689391000
H	1.975773000	0.730344000	3.750123000
H	2.802444000	1.364991000	2.317019000
C	-1.839155000	-1.007972000	2.688315000
H	-1.975878000	-0.734062000	3.749318000
H	-2.802579000	-1.367259000	2.315587000
C	0.500030000	-1.636161000	2.925806000
H	1.123998000	-2.526682000	3.010029000
H	0.501302000	-1.152955000	3.917555000
C	-2.945673000	1.411847000	1.296624000
H	-3.493740000	0.734026000	0.632401000
C	-3.856332000	1.714298000	2.503051000
H	-4.112381000	0.791981000	3.035365000
H	-3.332610000	2.363771000	3.215641000
C	-5.137439000	2.421894000	2.023407000
H	-5.713456000	1.727691000	1.396367000
H	-5.764904000	2.662760000	2.888557000
C	-4.819647000	3.691308000	1.218881000
H	-5.747376000	4.153140000	0.863158000
H	-4.332424000	4.423576000	1.877571000
C	-3.888916000	3.386815000	0.035754000
H	-3.634306000	4.307991000	-0.500282000
H	-4.408865000	2.735091000	-0.680516000
C	-2.601808000	2.693339000	0.507861000
H	-2.046225000	3.384352000	1.155652000
H	-1.943302000	2.465378000	-0.336918000
C	2.945752000	-1.413084000	1.295626000
H	3.493828000	-0.734599000	0.632089000
C	2.602239000	-2.693934000	0.505668000

H	2.046685000	-3.385647000	1.152745000
H	1.943796000	-2.465330000	-0.338984000
C	3.889546000	-3.386749000	0.033135000
H	3.635183000	-4.307467000	-0.503805000
H	4.409500000	-2.734264000	-0.682436000
C	4.820117000	-3.692181000	1.216150000
H	5.747982000	-4.153539000	0.860167000
H	4.332894000	-4.425129000	1.874085000
C	5.137546000	-2.423448000	2.021897000
H	5.713556000	-1.728583000	1.395583000
H	5.764891000	-2.664981000	2.886947000
C	3.856234000	-1.716502000	2.501936000
H	3.332468000	-2.366707000	3.213828000
H	4.112044000	-0.794646000	3.035158000
C	1.246746000	3.307307000	3.116412000
H	2.213734000	3.608686000	2.704428000
H	1.341710000	3.230439000	4.211770000
C	-1.246982000	-3.310437000	3.113013000
H	-2.213849000	-3.611453000	2.700477000
H	-1.342236000	-3.234615000	4.208418000
H	-0.517558000	-4.089209000	2.876689000
H	0.517417000	4.086334000	2.880636000
H	-0.517527000	4.089013000	-2.877101000
H	0.517503000	-4.086539000	-2.880163000

(Cy, Me) complex in the triplet spin state

Ni	-0.005773000	0.000005000	0.000003000
P	1.424656000	0.383756000	-1.855152000
P	-1.423399000	-0.779686000	-1.727145000
N	-0.859607000	1.740243000	-2.703900000
N	0.872038000	-2.286161000	-2.260527000
C	0.498746000	1.400453000	-3.135695000
H	1.061854000	2.328723000	-3.251571000
H	0.528538000	0.884509000	-4.110238000
C	-1.810345000	0.639734000	-2.868356000
H	-1.880029000	0.280321000	-3.909844000
H	-2.803772000	0.995027000	-2.578697000
C	1.821103000	-1.246038000	-2.661044000
H	1.887925000	-1.120752000	-3.755794000
H	2.815532000	-1.526922000	-2.301337000
C	-0.489320000	-2.051382000	-2.750413000
H	-1.047341000	-2.984667000	-2.652775000
H	-0.523625000	-1.767714000	-3.815842000
C	3.043786000	1.239998000	-1.583544000
H	3.569376000	0.560671000	-0.897748000
C	3.924715000	1.434016000	-2.832326000
H	4.106435000	0.476668000	-3.332901000
H	3.406783000	2.080605000	-3.552817000
C	5.262646000	2.083653000	-2.434941000
H	5.821136000	1.383339000	-1.798666000
H	5.870135000	2.249436000	-3.331712000
C	5.053768000	3.403904000	-1.677301000
H	6.020662000	3.821411000	-1.374365000
H	4.589115000	4.136876000	-2.351567000
C	4.149369000	3.210805000	-0.450342000
H	3.970270000	4.169560000	0.049745000
H	4.652593000	2.560737000	0.279265000
C	2.807877000	2.574662000	-0.844627000
H	2.265141000	3.267332000	-1.502363000
H	2.174240000	2.419496000	0.035812000
C	-3.053550000	-1.558302000	-1.314952000
H	-3.597317000	-0.748231000	-0.809414000
C	-2.841668000	-2.698927000	-0.298068000
H	-2.270286000	-3.509927000	-0.770095000
H	-2.241684000	-2.348402000	0.548898000
C	-4.194135000	-3.253335000	0.175114000
H	-4.029413000	-4.081614000	0.873654000
H	-4.727528000	-2.469216000	0.730435000
C	-5.051834000	-3.712405000	-1.013956000
H	-6.025527000	-4.072212000	-0.662832000
H	-4.557621000	-4.562227000	-1.505203000
C	-5.243300000	-2.582204000	-2.036487000
H	-5.823591000	-1.769955000	-1.577464000
H	-5.821463000	-2.937860000	-2.896639000
C	-3.894697000	-2.019361000	-2.519988000
H	-3.354977000	-2.800642000	-3.070816000
H	-4.059332000	-1.190723000	-3.216989000
C	-1.349136000	2.955571000	-3.366725000
H	-2.328154000	3.219426000	-2.956935000
H	-1.447509000	2.838988000	-4.458598000
C	1.360696000	-3.614246000	-2.652182000
H	2.344708000	-3.782385000	-2.205793000
H	1.448025000	-3.733945000	-3.744657000
P	1.424620000	-0.383758000	1.855184000
P	-1.423421000	0.779704000	1.727131000
N	-0.859658000	-1.740228000	2.703907000
N	0.872022000	2.286164000	2.260538000
C	0.498694000	-1.400456000	3.135714000
H	1.061792000	-2.328732000	3.251587000
H	0.528484000	-0.884517000	4.110260000
C	-1.810381000	-0.639706000	2.868353000
H	-1.880060000	-0.280282000	3.909838000
H	-2.803813000	-0.994988000	2.578697000
C	1.821063000	1.246032000	2.661085000
H	1.887854000	1.120748000	3.755837000
H	2.815506000	1.526904000	2.301405000
C	-0.489351000	2.051410000	2.750395000
H	-1.047360000	2.984700000	2.652721000
H	-0.523685000	1.767766000	3.815829000
C	3.043745000	-1.240008000	1.583580000
H	3.569340000	-0.560678000	0.897789000
C	3.924674000	-1.434042000	2.832360000
H	4.106397000	-0.476701000	3.332945000
H	3.406739000	-2.080638000	3.552843000
C	5.262602000	-2.083679000	2.434965000
H	5.821093000	-1.383358000	1.798699000
H	5.870092000	-2.249476000	3.331733000
C	5.053720000	-3.403920000	1.677308000
H	6.020613000	-3.821424000	1.374364000
H	4.589069000	-4.136901000	2.351566000
C	4.149316000	-3.210806000	0.450355000
H	3.970215000	-4.169555000	-0.049743000
H	4.652538000	-2.560731000	-0.279249000
C	2.807828000	-2.574664000	0.844652000
H	2.265096000	-3.267339000	1.502387000
H	2.174185000	-2.419490000	-0.035781000
C	-3.053574000	1.558309000	1.314926000
H	-3.597334000	0.748228000	0.809396000
C	-2.841704000	2.698925000	0.298030000
H	-2.270338000	3.509939000	0.770050000
H	-2.241710000	2.348401000	-0.548928000
C	-4.194179000	3.253303000	-0.175165000
H	-4.029470000	4.081575000	-0.873717000
H	-4.727556000	2.469166000	-0.730476000
C	-5.051890000	3.712376000	1.013895000
H	-6.025588000	4.072159000	0.662762000
H	-4.557695000	4.562215000	1.505129000
C	-5.243339000	2.582189000	2.036445000
H	-5.823617000	1.769922000	1.577435000
H	-5.821508000	2.937849000	2.896590000
C	-3.894728000	2.019375000	2.519955000
H	-3.355019000	2.800674000	3.070768000
H	-4.059350000	1.190747000	3.216972000
C	-1.349210000	-2.955548000	3.366731000
H	-2.328228000	-3.219391000	2.956932000
H	-1.447593000	-2.838961000	4.458603000
C	1.360689000	3.614246000	2.652192000
H	2.344713000	3.782369000	2.205824000
H	1.447994000	3.733955000	3.744668000
H	0.678480000	4.377934000	2.269895000
H	-0.661521000	-3.780531000	3.163412000
H	0.678468000	-4.377927000	-2.269908000
H	-0.661437000	3.780544000	-3.163396000

(^{t}Bu, Me) complex in the singlet spin state

Ni	-0.000086000	-0.000001000	0.000469000
P	-1.712786000	-1.251419000	-0.839764000
P	-1.719492000	1.241394000	0.840990000
N	-2.512070000	1.337007000	-1.742151000
N	-2.502466000	-1.351697000	1.744012000
C	-2.916022000	-0.065296000	-1.715833000
H	-2.975217000	-0.411105000	-2.746827000
H	-3.917160000	-0.215340000	-1.277512000
C	-2.766890000	2.018596000	-0.475465000
H	-3.830290000	1.998881000	-0.180094000
H	-2.474676000	3.067231000	-0.568688000
C	-2.754450000	-2.034825000	0.477601000
H	-3.818216000	-2.021417000	0.183232000
H	-2.455922000	-3.081701000	0.570548000
C	-2.914874000	0.048146000	1.718069000
H	-2.975377000	0.393593000	2.749108000
H	-3.917241000	0.192131000	1.280519000
C	-1.382294000	-2.541527000	-2.187897000
C	-1.395527000	2.533439000	2.188773000
C	-3.127007000	2.058957000	-2.863088000
H	-2.744986000	3.083168000	-2.879158000
H	-4.226511000	2.094257000	-2.797866000
C	-3.111740000	-2.077311000	2.865664000
H	-2.723453000	-3.099167000	2.881381000
H	-4.211080000	-2.119329000	2.801676000
P	1.720088000	-1.241396000	0.839621000
P	1.711862000	1.251438000	-0.840922000
N	2.504425000	1.351658000	1.741944000
N	2.510797000	-1.337014000	-1.743990000
C	2.916939000	-0.048118000	1.714800000
H	2.979472000	-0.393914000	2.745607000
H	3.918473000	-0.191867000	1.275262000
C	2.754410000	2.035313000	0.475412000
H	3.817840000	2.022761000	0.179774000
H	2.455239000	3.081957000	0.568975000
C	2.765994000	-2.019156000	-0.477652000
H	3.829602000	-2.000299000	-0.182969000
H	2.472932000	-3.067555000	-0.570928000
C	2.914857000	0.065272000	-1.717384000
H	2.973978000	0.411281000	-2.748314000
H	3.916060000	0.215104000	-1.279143000
C	1.397161000	-2.532786000	2.188233000
C	1.380198000	2.540924000	-2.189317000
C	3.115571000	2.076906000	2.862829000
H	2.727345000	3.098773000	2.879494000
H	4.214807000	2.118915000	2.797027000
C	3.125315000	-2.058638000	-2.865375000
H	2.743259000	-3.082834000	-2.881626000
H	4.224840000	-2.093998000	-2.800532000
H	2.844659000	-1.576553000	-3.805353000
H	2.838651000	1.593056000	3.803007000
H	-2.833292000	-1.593745000	3.805537000
H	-2.846656000	1.577170000	-3.803309000
C	-0.493554000	3.645114000	1.635090000
H	-0.266880000	4.349609000	2.442390000
H	0.450255000	3.236199000	1.276946000
H	-0.967592000	4.211142000	0.830030000
C	-2.725413000	3.146907000	2.672264000
H	-2.500316000	3.884829000	3.450110000
H	-3.253698000	3.666005000	1.866963000
H	-3.403657000	2.411076000	3.110738000
C	-0.653503000	1.820192000	3.337920000
H	-0.437188000	2.555270000	4.120606000
H	-1.241114000	1.020892000	3.796658000
H	0.295785000	1.405991000	2.987779000
C	2.727287000	-3.146707000	2.670463000
H	2.502725000	-3.884265000	3.448807000
H	3.254401000	-3.666317000	1.864721000
H	3.406368000	-2.411033000	3.107907000
C	0.494020000	-3.644254000	1.636005000
H	0.267553000	-4.348073000	2.443954000
H	-0.449821000	-3.234987000	1.278332000
H	0.967113000	-4.211103000	0.830974000
C	0.656811000	-1.818691000	3.337956000
H	0.440723000	-2.553408000	4.121043000
H	1.245493000	-1.019717000	3.795891000
H	-0.292531000	-1.403886000	2.988690000
C	-2.708923000	-3.163005000	-2.670062000
H	-3.392051000	-2.431265000	-3.107811000
H	-2.480206000	-3.899541000	-3.448164000
H	-3.233228000	-3.685273000	-1.864213000
C	-0.645709000	-1.823783000	-3.337756000
H	0.301502000	-1.404067000	-2.988568000
H	-0.425890000	-2.557481000	-4.120760000
H	-1.238459000	-1.027872000	-3.795775000
C	-0.472941000	-3.647655000	-1.635164000
H	0.468689000	-3.232882000	-1.278009000
H	-0.942627000	-4.216671000	-0.829662000
H	-0.242734000	-4.350690000	-2.442740000
C	2.706302000	3.162648000	-2.672549000
H	3.389482000	2.430964000	-3.110305000
H	2.476895000	3.898725000	-3.450885000
H	3.230835000	3.685499000	-1.867225000
C	0.643142000	1.822371000	-3.338372000
H	1.236172000	1.026913000	-3.796809000
H	-0.303341000	1.401847000	-2.988186000
H	0.421907000	2.555731000	-4.121292000
C	0.470889000	3.647003000	-1.636431000
H	-0.470293000	3.232144000	-1.278173000
H	0.941154000	4.216632000	-0.831710000
H	0.239707000	4.349502000	-2.444197000

(^{t}Bu, Me) complex in the triplet spin state

Ni	-0.002379000	0.272084000	-0.030709000
P	-0.496504000	-0.867131000	1.989545000
P	-2.213705000	1.041005000	0.221960000
N	-2.870331000	-1.612472000	0.650300000
N	-0.884339000	1.839649000	2.514005000
C	-2.156299000	-1.751208000	1.918841000
H	-1.937785000	-2.810998000	2.060113000
H	-2.760152000	-1.434158000	2.786063000
C	-3.485408000	-0.298479000	0.464801000
H	-4.159667000	-0.018873000	1.292710000
H	-4.091246000	-0.331054000	-0.443887000
C	-0.761247000	0.546491000	3.193266000
H	-1.617036000	0.327633000	3.853672000
H	0.129542000	0.581264000	3.823084000
C	-2.169728000	2.006469000	1.826258000
H	-2.278512000	3.059634000	1.562005000
H	-3.033312000	1.740177000	2.457620000
C	0.636845000	-2.072643000	2.902681000
C	-2.869262000	2.225076000	-1.085973000
C	-3.876783000	-2.673110000	0.500652000
H	-4.340569000	-2.593037000	-0.485992000
H	-4.667678000	-2.621953000	1.266464000
C	-0.634150000	2.950315000	3.442594000
H	0.358686000	2.836685000	3.884905000
H	-1.376752000	3.001220000	4.255039000
P	2.200027000	1.008566000	-0.424946000
P	0.508064000	-1.245251000	-1.774939000
N	0.844764000	1.309978000	-2.817012000
N	2.907067000	-1.660870000	-0.332112000
C	2.136712000	1.619766000	-2.193487000
H	2.241005000	2.704919000	-2.154788000
H	2.995896000	1.233123000	-2.764896000
C	0.725707000	-0.090192000	-3.234833000
H	1.570113000	-0.423339000	-3.860725000
H	-0.178639000	-0.184011000	-3.839162000
C	3.499451000	-0.325075000	-0.390240000
H	4.183710000	-0.191046000	-1.245832000
H	4.087732000	-0.172771000	0.517909000
C	2.200996000	-2.045586000	-1.552620000
H	2.017804000	-3.119950000	-1.502980000

H	2.794518000	-1.868195000	-2.465786000
C	2.828026000	2.448619000	0.612147000
C	-0.587638000	-2.641581000	-2.424638000
C	0.571430000	2.217847000	-3.940145000
H	-0.426677000	2.014912000	-4.336150000
H	1.302369000	2.112698000	-4.757692000
C	3.922645000	-2.660136000	0.026569000
H	4.375290000	-2.385473000	0.983120000
H	4.721292000	-2.749392000	-0.727665000
H	3.445204000	-3.636044000	0.146814000
H	0.590842000	3.250506000	-3.582890000
H	-0.653456000	3.893189000	2.890166000
H	-3.389343000	-3.649069000	0.567185000
C	-2.940113000	1.457293000	-2.419169000
H	-3.260791000	2.146240000	-3.207783000
H	-1.958683000	1.063930000	-2.694460000
H	-3.654963000	0.630319000	-2.396052000
C	-4.252293000	2.777651000	-0.699562000
H	-4.594081000	3.463164000	-1.483108000
H	-4.998986000	1.983408000	-0.605502000
H	-4.224893000	3.338112000	0.239812000
C	-1.835522000	3.361456000	-1.224921000
H	-2.152226000	4.028154000	-2.034101000
H	-1.747370000	3.967446000	-0.318769000
H	-0.848487000	2.963267000	-1.477768000
C	4.203750000	2.935558000	0.124697000
H	4.525408000	3.776604000	0.749121000
H	4.966095000	2.154251000	0.202997000
H	4.175840000	3.286134000	-0.911304000
C	2.906919000	1.976577000	2.076452000
H	3.223882000	2.817839000	2.701939000
H	1.929592000	1.642499000	2.433105000
H	3.629010000	1.168866000	2.224139000
C	1.771051000	3.567321000	0.510468000
H	2.069056000	4.395016000	1.162984000
H	1.674891000	3.968909000	-0.502366000
H	0.791946000	3.208136000	0.840892000
C	0.045759000	-2.457949000	4.271964000
H	-0.925444000	-2.953246000	4.183034000
H	0.728115000	-3.159353000	4.765213000
H	-0.067810000	-1.591498000	4.930421000
C	0.814626000	-3.319337000	2.015201000
H	1.223366000	-3.044589000	1.040712000
H	1.524161000	-3.998800000	2.499654000
H	-0.115908000	-3.874123000	1.867797000
C	2.008549000	-1.396115000	3.081161000
H	2.428361000	-1.118220000	2.112993000
H	1.966921000	-0.511131000	3.721719000
H	2.693168000	-2.107927000	3.554922000
C	0.013582000	-3.264879000	-3.699013000
H	1.001086000	-3.701793000	-3.523948000
H	-0.645556000	-4.070704000	-4.041071000
H	0.097376000	-2.540196000	-4.514300000
C	-0.721959000	-3.704638000	-1.316384000
H	0.228662000	-4.184437000	-1.067998000
H	-1.146802000	-3.273214000	-0.408186000
H	-1.400182000	-4.490779000	-1.664993000
C	-1.979849000	-2.053546000	-2.719450000
H	-2.399644000	-1.590499000	-1.824779000
H	-1.966175000	-1.320148000	-3.529980000
H	-2.648648000	-2.864183000	-3.028038000

The optimized structure to obtain the energy diagram in Figure 3a.

E_{tot} and E_{ZPVE} is the total energy and zero-point vibration energy obtained at the B3LYP-D3/BS1 level. E_{SPt} is the total energy obtained at the B3LYP-D3/BS2//B3LYP-D3/BS1 level.

H_2

E_{tot} = -1.178021445592
E_{zpve} = 0.010142913686
E_{SP} = -1.17913177692

H	-0.021741260241	0.000000000000	0.000000000000
H	0.721741260241	0.000000000000	0.000000000000

(H, H) complex in the ^{1}R state

E_{tot} = -2074.71734102401
E_{zpve} = 0.336658486058
E_{SP} = -2074.99478036

Ni	0.000022477189	-0.028226046570	-0.208345415894
P	1.707544647153	0.837374299518	-1.284811514974
P	1.496156608485	-0.940773677038	1.116245262188
N	2.691056238737	-1.770709852359	-1.255130030746
N	3.401792937175	1.058346146347	0.901058983264
C	3.003771243110	-0.441964726985	-1.795692654283
H	2.970989130728	-0.513406500453	-2.887281141124
H	3.999650006736	-0.072264826880	-1.503541389096
C	2.821674986148	-1.925993361698	0.198452543817
H	3.811701820265	-1.634818096446	0.584824525953
H	2.659250843685	-2.982188184980	0.434856012092
C	2.730355650437	1.893521162648	-0.094137235968
H	3.502456443268	2.431709189972	-0.652807503857
H	2.038778761828	2.633998940524	0.344503563988
C	2.562059857071	0.405556736952	1.904568313468
H	1.872888160027	1.096428567266	2.421604261250
H	3.224114255170	-0.042290503360	2.652389700144
P	-1.496493227235	1.230764973154	0.795831449914
P	-1.708567584273	-1.163061724750	-0.989742871018
N	-3.403335787458	-0.745175523402	1.168742138034
N	-2.686972871983	1.341768434100	-1.718144048005
C	-2.560308592013	0.167005599038	1.940045775434
H	-1.868164167402	-0.346822389536	2.630479791845
H	-3.219718354257	0.810280851978	2.531323002682
C	-2.735693011661	-1.831052228720	0.451804645803
H	-3.510794933623	-2.502121524026	0.068439877444
H	-2.048077349832	-2.420888211195	1.083168906126
C	-2.820547426198	1.908860501375	-0.370181973437
H	-3.811013107964	1.739721188396	0.081721006828
H	-2.660122838781	2.988764208738	-0.446681810854
C	-3.003683974001	-0.085813076866	-1.851066006197
H	-2.973972692414	-0.334371668975	-2.916389312288
H	-3.999943566798	-0.351650118973	-1.463263820188
H	-1.376252754436	2.361446412316	1.655321325783
H	-4.107821996293	-1.141733941307	1.784224779635
H	-1.807292414855	-2.312340710250	-1.827514982025
H	3.307578673977	-2.446347420977	-1.701726886304
H	1.374900894773	-1.774451009846	2.265940350961
H	1.809033136478	1.693772799638	-2.420097479022
H	4.108297948118	1.614341696669	1.374513355329
H	-3.302322099073	1.860927216968	-2.341045896709

(H, H) complex in the ^{1}A state

E_{tot} = -2075.6811815054
E_{zpve} = 0.368533495332
E_{SP} = -2075.94658148

Ni	-0.000081955575	0.000058888767	0.054101859261
P	1.560610800243	0.961375821186	-1.144921847399
P	1.585518613647	-0.858118110496	1.289292066749
N	2.431786030492	-1.699385757011	-1.243466463371
N	3.527032654765	1.028557862691	0.784582235943
C	2.721813644156	-0.348348452693	-1.850808846232
H	2.546018547299	-0.453015696290	-2.922888350248
H	3.770925011469	-0.120090172175	-1.660916106929
C	2.749374883628	-1.887067919448	0.219953783868
H	3.795238503808	-1.616283150084	0.366371547230
H	2.600155223789	-2.947506842197	0.430446758044
C	2.785865924634	1.932549813113	-0.091334617209
H	3.503183668509	2.436164364220	-0.745215731465
H	2.205080676810	2.699580033645	0.448262345533
C	2.798624436723	0.444378907598	1.908141472361
H	2.211533864659	1.176417545141	2.487481522938
H	3.524266734108	-0.027919614885	2.576027592088
P	-1.578017352608	1.184805353831	0.991712559497
P	-1.569447070203	-1.252776309119	-0.823129369848
N	-3.537340526069	-0.751392221207	1.038321933897
N	-2.416392074990	1.275000250493	-1.680008005421
C	-2.813914945635	0.132921987764	1.948860342800
H	-2.243693124031	-0.399702857364	2.728461035714
H	-3.541599282754	0.787339302564	2.436686868077
C	-2.794412418863	-1.872950654986	0.469194988526
H	-3.509815221053	-2.548776723319	-0.007340752648
H	-2.211510791794	-2.443524770943	1.211502504985
C	-2.722867883418	1.885841121837	-0.334188683143
H	-3.774819326968	1.696668045713	-0.118717485975
H	-2.545632086991	2.957593386030	-0.439759354710

C	-2.727344651174	-0.189870818694	-1.866710408420
H	-2.567471710871	-0.402709264365	-2.925138593558
H	-3.775146284881	-0.339285612529	-1.605280256432
H	-1.545241556933	2.328406869787	1.829407531099
H	-4.370056541536	-1.101960667595	1.502146580177
H	-1.543748448559	-2.382365066309	-1.681389886225
H	2.940546467420	-2.411378598725	-1.779104564868
H	1.562985012900	-1.717972724987	2.416689840486
H	1.524765014651	1.801093223158	-2.288051934788
H	4.355122377982	1.502716662393	1.132270692300
H	-2.918918836777	1.807056159898	-2.399087202990
H	1.425245925052	-1.888128838286	-1.370768216478
H	-1.408275643372	1.404878750229	-1.858281269238

(H, H) complex in the $^1TS_{B1}$ state

E_{tot} = -2075.657269
E_{zpve} = 0.360781294732
E_{SP} = -2075.92220108

Ni	0.024933103808	-0.003033770203	-0.225064495074
P	-1.731751241692	0.079125018185	-1.490281155084
P	-1.353597759335	-0.250279900015	1.489871158917
N	-2.404588323293	2.080760342786	0.348666817163
N	-3.457210701733	-1.321106259834	0.109842611588
C	-2.863747452666	1.487720379736	-0.961158389931
H	-2.815575880996	2.293631941372	-1.695130833783
H	-3.893616056711	1.155289055309	-0.828376322221
C	-2.517405352383	1.219791615933	1.583182551609
H	-3.558881560904	0.909904509727	1.675764696909
H	-2.236526273053	1.854956062958	2.425060441994
C	-2.884495974120	-1.380971839256	-1.232414441714
H	-3.701031210742	-1.322058937419	-1.957135694514
H	-2.299971566764	-2.293792915598	-1.431823390542
C	-2.575567628032	-1.647628610854	1.227202882399
H	-1.985986358060	-2.567493725652	1.081337110579
H	-3.187031514540	-1.762101178783	2.126077421791
P	1.576345083699	-1.585235036966	-0.155924334254
P	1.565862665970	1.523670201425	0.251757332494
N	3.397840352036	-0.199341633076	1.371105426698
N	2.323695062157	0.245983771708	-2.012664768887
C	2.661207862499	-1.460448667936	1.371802253240
H	2.002400032220	-1.591701841326	2.246444956257
H	3.386370407135	-2.278690762598	1.358926912945
C	2.651968045261	1.012925088277	1.696508516408
H	3.370675963836	1.812624950555	1.895340336523
H	1.994037860371	0.910137834450	2.575778795049
C	2.755341023026	-1.083911410448	-1.547631859218
H	3.791145436522	-1.085499588919	-1.200334311950
H	2.636790288929	-1.787785987127	-2.373499042631
C	2.747868591965	1.412807542964	-1.220045673215
H	2.622581475599	2.306260064548	-1.834733213788
H	3.784447450804	1.332483341022	-0.884709573157
H	1.516066714243	-2.990872389269	-0.313189803286
H	4.194917180508	-0.278039855248	1.995090232432
H	1.502372282321	2.922089076756	0.468614653663
H	-2.934715563742	2.944300955676	0.513442719323
H	-1.041523855354	-0.389568321310	2.859474054333
H	-1.796439732720	0.247467935391	-2.890022302635
H	-4.288377194898	-1.902845206063	0.151607990912
H	2.489482223163	0.376786886748	-3.008225176029
H	-1.417037070380	2.356553858666	0.241708227311
H	0.929207557012	0.187260427630	-1.572712030428

(H, H) complex in the $^1B1$ state

E_{tot} = -2075.6673839954
E_{zpve} = 0.362576821272
E_{SP} = -2075.93327107

Ni	0.022173634142	0.211218920285	0.389232599094
P	1.900144048043	0.360117547690	1.460376648923
P	1.245272441704	-0.478302599327	-1.333209096052
N	2.506582924595	1.948708897401	-0.754585338869
N	3.373662601501	-1.451009722142	0.056604271837
C	3.037968089746	1.582314974226	0.608226299473
H	3.079279463474	2.506812018132	1.185760851332
H	4.039263714510	1.171728680112	0.474532768050
C	2.472861117655	0.857151155472	-1.795628862573
H	3.482628427778	0.456016641086	-1.886693475312
H	2.165441648532	1.328562045700	-2.730205703347
C	2.921450164631	-1.206286679438	1.421224786110
H	3.796436979283	-1.068169228966	2.061624235783
H	2.293403684157	-2.007575723225	1.842676761488
C	2.368411410394	-1.906139571691	-0.897267498655
H	1.724638303913	-2.720075104930	-0.527303914514
H	2.874341088764	-2.242376845297	-1.805726705851
P	-1.561710507142	-1.437777251642	0.617361785141
P	-1.601709851665	1.407650527062	-0.544228748174
N	-3.095951925435	-0.666951541653	-1.537907451174
N	-2.920763050056	0.687555573116	1.795172178649
C	-2.333137404313	-1.815362142629	-1.057537219508
H	-1.522989076577	-2.132905456789	-1.734069231426

H	-3.026681661973	-2.652172991155	-0.937928548353
C	-2.350901111055	0.497466417480	-2.004560995766
H	-3.052359502844	1.172570786576	-2.502619473915
H	-1.538248448136	0.262842251344	-2.710393466661
C	-3.032447394790	-0.754327800479	1.574861145677
H	-3.945854106634	-1.053062710714	1.039401911322
H	-3.030291927293	-1.243002628818	2.553057531077
C	-3.066451983531	1.552120554664	0.626741507675
H	-3.096063544169	2.588191889105	0.974804454827
H	-3.973075846965	1.355261212859	0.036143202883
H	-1.470786846634	-2.745668617274	1.148826732006
H	-3.740760399665	-0.967813765167	-2.261978172675
H	-1.500748920310	2.718668085702	-1.062543841557
H	3.076953476837	2.719532746285	-1.122807248810
H	0.729282873613	-0.830258136786	-2.593254438896
H	1.998828317637	0.778289814590	2.797505415005
H	4.160853661816	-2.091931634983	0.061916039586
H	-3.599173332492	0.968220487255	2.498562533519
H	1.550991495353	2.319724667846	-0.640922560469
H	-0.511880796909	0.828780534942	1.579274198144

(H, H) complex in the $^1TS_{B2}$ state

E_{tot} = -2075.66730646471
E_{zpve} = 0.362344318853
E_{SP} = -2075.93353944

Ni	-0.029669072800	0.301355311697	-0.393699849493
P	-1.865170875005	0.031355726046	-1.506709403106
P	-1.254882742494	-0.121781468884	1.418316106713
N	-2.714914718866	1.984461113812	0.300712586695
N	-3.222308694543	-1.572786257127	0.223470955031
C	-3.146406850575	1.284882472561	-0.963403265165
H	-3.250565908380	2.056508337423	-1.727442301724
H	-4.108355523864	0.809778197331	-0.767919725571
C	-2.621948575697	1.149677144999	1.553904936132
H	-3.592499357229	0.677489795252	1.708441823983
H	-2.400044618786	1.838872322571	2.369966335076
C	-2.732258802068	-1.585797868783	-1.149294488138
H	-3.585447682662	-1.677158660643	-1.826532819316
H	-2.014986134860	-2.392345565019	-1.369989005310
C	-2.225926536986	-1.713938838568	1.278433731921
H	-1.497405589970	-2.522747485678	1.110025455138
H	-2.740559906714	-1.894563407126	2.225393591296
P	1.498206139450	-1.489260407163	-0.419666249157
P	1.667644697855	1.469514030308	0.352804166329
N	3.076515798822	-0.509346225284	1.617239821754
N	2.937861436674	0.388778019795	-1.874666164463
C	2.259228086364	-1.675317670446	1.293730386741
H	1.436057723112	-1.856782057903	2.004440733492
H	2.911496511557	-2.552589164847	1.293248639740
C	2.383673062353	0.736784962343	1.922666634111
H	3.114170302198	1.446004510437	2.321907486928
H	1.565654477022	0.634518511875	2.653233291923
C	2.997099011629	-1.012837268649	-1.453958403910
H	3.901368397407	-1.266829652464	-0.880622176437
H	2.973595580441	-1.633003007998	-2.354257751249
C	3.121829041670	1.396770955612	-0.833549446863
H	3.182337400519	2.375690491055	-1.317125677215
H	4.025768245302	1.251718475355	-0.223558631909
H	1.354566464751	-2.853262517323	-0.766628901618
H	3.710359453674	-0.738361150412	2.376480532800
H	1.598115408082	2.841111869558	0.678349331878
H	-3.373850574101	2.751708813736	0.479635121419
H	-0.770368438490	-0.143889691813	2.738677210844
H	-1.934944869767	0.142644366941	-2.904771289720
H	-3.952402342362	-2.268427123264	0.339174862552
H	3.634903312510	0.542850035600	-2.599015278692
H	-1.796492978983	2.421160594057	0.130248249760
H	0.415398587183	0.932580256481	-1.622997100514

(H, H) complex in the $^1B2$ state

E_{tot} = -2075.67148412071
E_{zpve} = 0.363005367728
E_{SP} = -2075.93731241

Ni	-0.042287922808	0.359081959392	-0.456473735351
P	1.668474472449	1.510955401143	0.280786632888
P	1.425275042727	-1.528743231292	-0.290392134030
N	2.952406382362	0.230681875246	-1.869514025802
N	3.059639898675	-0.412730801590	1.622007103891
C	3.126264685949	1.353273595206	-0.883038372127
H	3.215228533699	2.269057923593	-1.469403372823
H	4.045803234677	1.168951298626	-0.327165445668
C	2.945105616983	-1.181730269564	-1.336499648814
H	3.865595727511	-1.321808098665	-0.769302720870
H	2.938237983086	-1.834121158238	-2.210895263716
C	2.405928443656	0.865049105744	1.873125692051
H	3.158724138231	1.582309527596	2.210485591796
H	1.599957774528	0.824421423035	2.620974310393
C	2.201016663588	-1.576632019291	1.426625019932

H	1.374502723059	-1.649075433256	2.150661866097
H	2.818184369100	-2.474082906740	1.510669218508
P	-1.277886716316	0.134866556859	1.396826940110
P	-1.900953334010	-0.122252168400	-1.475827906484
N	-3.056296116742	-1.692371308190	0.439581074858
N	-2.860058433130	1.952938308905	0.058917090810
C	-2.055732003456	-1.560611934345	1.492517726889
H	-1.238575141957	-2.298912086589	1.439623817906
H	-2.554822243958	-1.661926669944	2.460012609473
C	-2.562532672322	-1.783594922656	-0.929261156907
H	-3.396902456186	-2.050919432360	-1.583517632397
H	-1.751649026934	-2.516321520032	-1.073617875610
C	-2.744813995533	1.299047039004	1.365558475821
H	-3.636695463541	0.725171853202	1.659483743891
H	-2.560128639123	2.069008654277	2.119109521813
C	-3.268776660363	1.082082830175	-1.045866481829
H	-3.440907515559	1.702412676661	-1.929193078738
H	-4.176903572132	0.494359651531	-0.842174928173
H	-0.739730817711	0.298862692211	2.686611259808
H	-3.664380040451	-2.480680743138	0.638790925672
H	-1.945953303397	-0.220940541730	-2.876912161107
H	3.707094564321	0.302727686674	-2.561858284689
H	1.304334735548	-2.909909075945	-0.564735017546
H	1.643739442976	2.905902822173	0.476627220600
H	3.735644747067	-0.601900685834	2.355001481990
H	-3.535217928786	2.710431977742	0.136579838879
H	2.069237700230	0.390356499079	-2.378914675387
H	0.328867578636	0.900002351469	-1.775530588510

(H, H) complex in the $^{1}TS_{C}$ state

E_{tot} = -2075.6473667851
E_{zpve} = 0.355461159374
E_{SP} = -2075.91314718

Ni	-0.005006418921	0.113253359659	-0.323175649494
P	1.634275021150	1.490996679869	0.126975674434
P	1.534960236768	-1.482453836251	-0.009024926607
N	2.591179233589	0.075667067446	-2.022767717591
N	3.304977204030	-0.120271792179	1.536099136802
C	2.920317223417	1.274049237964	-1.225502333060
H	2.879838185712	2.143265158742	-1.884124862263
H	3.907441888286	1.189892968385	-0.765182326592
C	2.815799238775	-1.233116285108	-1.366682704622
H	3.822523051301	-1.296234260383	-0.945801292526
H	2.677271730730	-2.011512237600	-2.119014676062
C	2.560261930402	1.121422971509	1.699511108960
H	3.267731711957	1.936826970721	1.872263777225
H	1.823047419246	1.113362090856	2.518424195183
C	2.533060961885	-1.357194603767	1.559202593665
H	1.829016045742	-1.441013124170	2.402557508180
H	3.227308751136	-2.201241631567	1.594551340351
P	-1.556402341703	1.283281476720	0.709027755190
P	-1.649980066189	-1.274939882268	-0.684264314957
N	-3.078967653131	-0.802990932060	1.568713448078
N	-2.917954341462	0.966144088093	-1.654730730772
C	-2.272350052153	0.282323039983	2.114292274307
H	-1.428199925186	-0.046143821193	2.742149793124
H	-2.919384033617	0.935154556337	2.706601320935
C	-2.359774750273	-1.894018999802	0.921825764530
H	-3.066364157317	-2.698344111848	0.699199781977
H	-1.524972231467	-2.307500018034	1.511089322499
C	-3.022978573363	1.682275605915	-0.383386193587
H	-3.936016878748	1.458181336132	0.189095244670
H	-2.988911228965	2.755764069543	-0.585442426687
C	-3.092257615087	-0.483384627905	-1.572480720130
H	-3.105940559383	-0.892719110871	-2.585837481463
H	-4.006788224716	-0.799344985962	-1.047845486170
H	-1.219981783540	2.504931449254	1.319521622037
H	-3.696365069799	-1.171000157565	2.285701862065
H	-1.345959110559	-2.442389031835	-1.405118645494
H	3.055575591398	0.108675889567	-2.930714099320
H	1.224347929030	-2.850505132486	-0.103042957578
H	1.376443402119	2.871231695032	0.183410824036
H	4.057541489740	-0.166383578074	2.215756233165
H	-3.607127307583	1.342168831119	-2.301731987340
H	1.287543875330	0.185576212719	-2.162044078805
H	0.262608441458	0.337913559507	-2.054086630107

(H, H) complex in the ^{1}C state

E_{tot} = -2075.67225467343
E_{zpve} = 0.354015123985
E_{SP} = -2075.94053794

Ni	-0.000829971016	0.022572672151	0.170173120718
P	-1.647179645188	1.467732136975	-0.173632979784
P	-1.641491894482	-1.466472946461	0.178041949400
N	-2.834272180213	0.236359028730	1.977753241032
N	-3.193758806592	-0.200684485141	-1.655323930855
C	-3.030365855926	1.371232144850	1.078485780097
H	-2.993446276832	2.292580995372	1.664707159698
H	-3.975225564753	1.345910670617	0.514418646928
C	-3.022423387853	-1.081737298250	1.376438775029
H	-3.968453889440	-1.199809699434	0.826142280382
H	-2.975970899901	-1.836215801324	2.165413613282
C	-2.434024223516	1.031872083642	-1.810405516988
H	-3.119493979161	1.838428771297	-2.082773323492
H	-1.627114080112	0.983430563635	-2.559268535283
C	-2.427979089034	-1.431375893917	-1.515910984889
H	-1.619463207065	-1.555420474832	-2.254066343753
H	-3.108908102935	-2.282960762030	-1.591925525225
P	1.647103033990	1.468241305401	-0.174262816263
P	1.639342852220	-1.466767100354	0.166954717026
N	3.233721479158	-0.191239552129	-1.621855202049
N	2.784856924324	0.230235692792	2.000767196721
C	2.473514402627	1.039041503784	-1.791781000193
H	1.685319088093	0.990784329716	-2.560410478660
H	3.162565154917	1.848921434417	-2.044675294082
C	2.467339244540	-1.423813976764	-1.505616052430
H	3.151615215486	-2.274182239883	-1.564950822911
H	1.679084706344	-1.548656491608	-2.265099418183
C	2.999916980360	1.370357795627	1.112673379851
H	3.959288073596	1.352241143052	0.573530843459
H	2.943672739512	2.288200374172	1.702830960778
C	2.991096077154	-1.085467913991	1.400093076847
H	2.925966210348	-1.842054514545	2.185790252821
H	3.951218069459	-1.200635561921	0.874488751865
H	1.337523892401	2.834172184455	-0.295575732390
H	3.926298307121	-0.278820860463	-2.358935528899
H	1.323492060100	-2.822901464534	0.359911220252
H	-3.465436762508	0.330378250176	2.769784043668
H	-1.327390280614	-2.820824992996	0.385874794127
H	-1.336565205753	2.833875694542	-0.289697532928
H	-3.868326342550	-0.292487086558	-2.408402239925
H	3.393494502311	0.322770012978	2.810385635099
H	-0.379883426925	0.388322148525	2.867500568747
H	0.370291470896	0.384987605655	2.854906906167

(H, H) complex in the ^{1}P state

E_{tot} = -2074.49036294972
E_{zpve} = 0.341184026993
E_{SP} = -2074.75791003

Ni	0.004072132852	-0.051908017308	0.143721891462
P	1.672365127649	1.359204202507	0.506761242019
P	1.638094960547	-1.489026008771	-0.286716255126
N	2.384539076262	0.515323998436	-2.004604431034
N	3.512616084871	-0.437367426040	1.372201058630
C	2.758489498597	1.516776848912	-1.003812711601
H	2.578287074961	2.510419720516	-1.420793095598
H	3.805145819643	1.454959597731	-0.669587452110
C	2.732879070269	-0.865967586609	-1.664976831371
H	3.777755604823	-1.005320272363	-1.349339795766
H	2.538389578659	-1.497975172029	-2.534801086455
C	2.782167186726	0.722011744273	1.864327909183
H	3.495719485131	1.511709143371	2.113241341532
H	2.149566930570	0.527385958277	2.745401005604
C	2.749784451207	-1.665673868558	1.202597211934
H	2.111905695400	-1.933563920469	2.060273858039
H	3.442242459212	-2.489002828213	1.009686859710
P	-1.620180532323	1.378569895707	0.625562677265
P	-1.673491095385	-1.464865450473	-0.186821516143
N	-3.438297396082	-0.396800119037	1.577263226520
N	-2.484054167583	0.563994099433	-1.847088311865
C	-2.661411202985	0.745166556229	2.037833253679
H	-1.984111395364	0.530725703255	2.880073263756
H	-3.346187624752	1.544189301384	2.333370392066
C	-2.707496621194	-1.636329084570	1.357098271640
H	-3.423443652952	-2.445741207594	1.192895695888
H	-2.031354154937	-1.924234140381	2.178167643405
C	-2.787113448382	1.563262258459	-0.820231322749
H	-3.814135368056	1.514449115138	-0.428387935986
H	-2.615354863898	2.556901056110	-1.240699798506
C	-2.831362743053	-0.815865874095	-1.500730861630
H	-2.689786812759	-1.441972996427	-2.384882355471
H	-3.860348684605	-0.945991336866	-1.133173937604
H	-1.277888966877	2.687009027585	1.008817019125
H	-4.224387728628	-0.559098043355	2.198789533269
H	-1.381054752159	-2.789935249588	-0.554068207021
H	2.835235789571	0.754578233483	-2.885115331920
H	1.307024980216	-2.811658250999	-0.629131420596
H	1.370259799619	2.675288555646	0.897623205783
H	4.327539448662	-0.609307640690	1.952729830103
H	-2.977718283468	0.817084977990	-2.700261352057

(Me, Me) complex in the ^{1}R state

E_{tot} = -2389.29316094426
E_{zpve} = 0.562786476124
E_{SP} = -2389.63798269

Ni	-0.000843831508	-0.046827777080	-0.099942959843

P	-1.511185343151	1.295666947689	0.727653187877
P	-1.695010864910	-1.223231663989	-0.811216031842
N	-3.517165383929	-0.610992587218	1.244136767750
N	-2.654918265025	1.223863034297	-1.846084720935
C	-2.658495756020	0.373897982139	1.905888419048
H	-2.025811707005	-0.080097448068	2.692675930809
H	-3.312053678600	1.108850598599	2.390258330025
C	-2.817875602491	-1.732877489361	0.613377163550
H	-3.579308257821	-2.417631595463	0.221786889466
H	-2.195152027600	-2.300336229078	1.331544060194
C	-2.774625300043	1.886145331503	-0.541442518634
H	-3.790370623541	1.795259573401	-0.115353876596
H	-2.576378327604	2.952541746312	-0.695375462293
C	-2.924479771101	-0.219963747940	-1.833127275969
H	-2.825848912641	-0.580262315907	-2.862979638966
H	-3.951310470338	-0.451587300591	-1.496127438112
P	1.676138888483	0.663787442578	-1.299170433962
P	1.523550308623	-0.800486150401	1.269365384858
N	2.689484388411	-1.958818513797	-1.020051412044
N	3.491569092467	1.154394209376	0.793645903030
C	2.930736852159	-0.685112026135	-1.710551833233
H	2.831489568872	-0.872330283534	-2.785341309662
H	3.951197136222	-0.299234484959	-1.532925841042
C	2.810141131764	-1.903763440461	0.442499668503
H	3.819589635005	-1.595584105431	0.770219428941
H	2.632578013099	-2.915268590103	0.823958284614
C	2.782420571673	1.818287895465	-0.302124627812
H	3.537478948631	2.236287532980	-0.977996883201
H	2.150595944842	2.655679339002	0.051516837745
C	2.639549069023	0.604305644011	1.849269550931
H	1.985991732627	1.371526948495	2.307148831893
H	3.296440922013	0.214864836032	2.635671434941
C	1.686625299650	1.599583134368	-2.904194310493
H	1.120548818684	1.033098947309	-3.650199208866
H	1.187138907973	2.562461760477	-2.754844770492
H	2.702131034319	1.777212908089	-3.277358079431
C	1.334785965373	-1.683211240354	2.893043316395
H	0.806397846953	-1.025834238123	3.591352149753
H	0.728124873082	-2.581644694972	2.743648451838
H	2.299972269848	-1.965115977310	3.329868139458
C	-1.726118729942	-2.818194321976	-1.763630369187
H	-1.126231614421	-2.702231094204	-2.671560523636
H	-1.270898266945	-3.603204355217	-1.150686625479
H	-2.743420203555	-3.122014716125	-2.036949701914
C	-1.298832825681	2.853312100473	1.717450975050
H	-0.792583969413	2.609412526187	2.657234426328
H	-0.664759037336	3.546905120682	1.156867678105
H	-2.255437090558	3.338841419150	1.943403415273
C	-3.512211434489	1.887550303762	-2.828694543323
H	-3.360189584078	1.440037041695	-3.816589070495
H	-4.588465487813	1.813524813407	-2.582921087129
H	-3.252685536830	2.949725529548	-2.890553767493
C	3.567616346931	-2.997786409217	-1.559222571286
H	3.414657926209	-3.089363590690	-2.639781111320
H	4.640298104451	-2.791105531204	-1.383202116844
H	3.329694287131	-3.961768327184	-1.097004977623
C	4.500945795603	2.046525599348	1.363390584000
H	4.061392350875	2.942753192021	1.840952582954
H	5.085130445761	1.512197970796	2.119901722449
H	5.185581884465	2.382734218053	0.577643258783
C	-4.539149402878	-1.100547537551	2.168680876531
H	-5.117608115071	-0.258414727950	2.562649181659
H	-5.226077234888	-1.770980368882	1.641725281187
H	-4.112173803988	-1.653873226768	3.026621957262

(Me, Me) complex in the ^{1}A state

E_{tot} = -2390.2642598465
E_{zpve} = 0.594825966499
E_{SP} = -2390.59933323

Ni	0.001333307773	-0.006911368082	0.000016397870
P	1.551214964950	-1.096354132029	-1.063525796736
P	1.588491984400	1.036330053587	1.062808520420
N	3.605872967083	-0.884296676935	0.821214025946
N	2.356518448855	1.552231735066	-1.603304736940
C	2.820737945263	-1.874059968295	0.079092814232
H	2.277953767982	-2.572250851035	0.742322440371
H	3.512116119531	-2.461304515315	-0.534521079105
C	2.856470114391	-0.121192188000	1.822079312748
H	3.571880269677	0.485143310477	2.387881995381
H	2.320074894964	-0.770097081203	2.538651073212
C	2.671568211078	0.119329178974	-1.973159195106
H	3.729636692313	-0.047759948973	-1.765954051399
H	2.481077694389	0.029477739197	-3.044395425972
C	2.699873885999	1.924928938409	-0.177787177385
H	2.526250481673	2.999237873465	-0.089918672094
H	3.757715233503	1.704089679215	-0.027301551040
C	1.397246031431	-2.418458776181	-2.345998011433
C	1.482992202121	2.328918101906	2.381555287982
C	4.783870068291	-1.510829616494	1.427381498297
H	5.404356693622	-0.744701085717	1.902487134664
H	5.379237734230	-2.001970952961	0.651526634087
C	2.978779929867	2.507210246994	-2.574964922626
H	2.644746950989	2.249164631632	-3.580130241731
H	4.062874183329	2.418213716615	-2.500335556998
P	-1.585880915676	1.046662901060	-1.051965088067
P	-1.548445703671	-1.108909766708	1.051279152365
N	-2.355140197810	1.534318671026	1.617971085038
N	-3.601914209578	-0.878196334439	-0.832459088861
C	-2.698890223470	1.921608377368	0.196667591318
H	-2.526246687318	2.996987613605	0.120085991202
H	-3.756515057456	1.701473450602	0.043691554507
C	-2.669665863779	0.097365799269	1.972642751429
H	-3.727540437876	-0.068107261453	1.763127680787
H	-2.479772177933	-0.003473417887	3.042924477549
C	-2.852011933229	-0.103945795426	-1.824469955878
H	-3.567320971974	0.507511748005	-2.384809406891
H	-2.314015954275	-0.744924723622	-2.546936581861
C	-2.816926140454	-1.875301402927	-0.100207296484
H	-2.273394574512	-2.566325609162	-0.770320098937
H	-3.508510614637	-2.469332190137	0.506667573654
C	-1.479790774164	2.352984054241	-2.357177005898
C	-1.395879932462	-2.443731418378	2.320676647239
C	-2.976928758058	2.478680855551	2.600169679983
H	-2.642759025808	2.209402649176	3.602402019627
H	-4.061079966140	2.391063186208	2.524750814539
C	-4.778930825959	-1.498873790047	-1.446473001752
H	-5.398875658837	-0.728123407884	-1.914754512112
H	-5.375303904881	-1.997847655740	-0.676408457944
H	-4.514949069977	-2.245391599605	-2.216996106042
H	-2.660000386832	3.493151935154	2.357540564543
H	2.662483446045	3.519220188663	-2.321512358720
H	4.521057907654	-2.265070520596	2.190741016992
H	0.819867217337	3.130049761318	2.041616068981
H	2.461973973622	2.747492940244	2.638273637729
H	1.043234322418	1.870893144297	3.273343550118
H	0.953673703409	-3.301220346097	-1.874588038165
H	2.364234645930	-2.690113567922	-2.782237729552
H	0.721740764322	-2.072167417606	-3.133479176113
H	-0.817657439464	3.151016282643	-2.008061526822
H	-2.458718990429	2.773617387932	-2.610717721083
H	-1.038600250045	1.904743393089	-3.253213382955
H	-0.952542753714	-3.322041099774	1.840828622070
H	-2.363433894752	-2.719017685527	2.753345693550
H	-0.720831155207	-2.105716962907	3.112121005073
H	1.328295773205	1.637851281016	-1.673938899635
H	-1.326855131258	1.619460159040	1.689063898824

(Me, Me) complex in the $^1TS_{B1}$ state

E_{tot} = -2390.24476205255
E_{zpve} = 0.587147900483
E_{SP} = -2390.5794015

Ni	0.031970449383	-0.069707538105	-0.167724754039
P	-1.311470605209	0.095585282182	1.553574147129
P	-1.726475527166	-0.308166568231	-1.378351964590
N	-3.473158211304	-1.323989656626	0.537643236841
N	-2.380654044594	2.116441477122	-0.098443213588
C	-2.536553172044	-1.309507339891	1.662914815271
H	-1.955213933702	-2.244739814633	1.752995746956
H	-3.113055086337	-1.179781070958	2.584343228094
C	-2.875948202958	-1.649316959477	-0.758869756031
H	-3.684314618730	-1.756600817272	-1.489375703028
H	-2.300435926378	-2.592293049068	-0.744536011884
C	-2.482939819380	1.541829616482	1.297235383133
H	-3.525088364689	1.263577815832	1.461680658743
H	-2.198778616742	2.342424134071	1.982342180457
C	-2.852098074540	1.189513798659	-1.196497063715
H	-2.817647520037	1.764877342827	-2.123356326077
H	-3.881159224772	0.907479556529	-0.969134044212
P	1.584989453736	1.487563584435	-0.115315310599
P	1.588686958524	-1.586822042159	0.188900880606
N	2.221040895827	-0.256405455145	-2.109106578935
N	3.539137132383	0.078334168443	1.303015619465
C	2.689509989806	1.041770476731	-1.584000095935
H	2.537260347997	1.802203914172	-2.352831072842
H	3.744784954361	1.015652699407	-1.299976924544
C	2.682367537216	-1.434020464524	-1.346027136407
H	3.741361816089	-1.365079774213	-1.083105517377
H	2.516765744102	-2.325142833223	-1.955267143898
C	2.756839475678	1.316138270995	1.333984334907
H	3.454666857301	2.158724844202	1.291539932764
H	2.157876601223	1.420143430748	2.257096442163
C	2.775581133508	-1.143851217218	1.565649735140
H	2.189874240163	-1.088334262406	2.501660120800
H	3.485970945013	-1.971123145164	1.665712290835
H	-1.374231349033	2.269659559655	-0.270835978798
H	0.868414087033	-0.197605897772	-1.566000455917
C	1.421723239156	3.321172981797	-0.224315434847
H	0.798298878700	3.570774292767	-1.087763680464
H	0.924699867132	3.683589608713	0.681161250172
H	2.395244698289	3.813326737325	-0.320731720586

C	1.433263831260	-3.407546361338	0.427522300436
H	0.974702254392	-3.597364904860	1.403097551629
H	0.777152844486	-3.811399989541	-0.348672060782
H	2.406867359343	-3.906735963896	0.384204797971
C	-1.758059695490	-0.581875485667	-3.195814954369
H	-1.178189281302	0.207003423344	-3.682336476433
H	-1.280420344402	-1.544150304614	-3.403601876981
H	-2.779093807037	-0.590071723032	-3.589969879368
C	-0.814082556789	0.325577703805	3.307994861839
H	-0.278239274544	-0.571088251301	3.633640095628
H	-0.135279337380	1.180208740208	3.374130023443
H	-1.679234487064	0.488031961354	3.958486327668
C	-3.073307491052	3.443615106875	-0.181515749186
H	-2.911531904653	3.856935913081	-1.177362965705
H	-4.137285799496	3.289589871905	0.000186754214
H	-2.648664494702	4.103733847326	0.575078214754
C	2.309558179869	-0.398765829810	-3.568993025885
H	1.814214367554	0.454622457166	-4.038947965507
H	3.350328560852	-0.444108756424	-3.911847659434
H	1.792866564916	-1.314468914467	-3.866927592682
C	4.683606692287	0.172381155121	2.214117718188
H	4.381418827340	0.269231954181	3.272429045401
H	5.305609050372	-0.722575460392	2.116023849980
H	5.292674658190	1.042477307094	1.950651949683
C	-4.610601319642	-2.204079960066	0.821236973877
H	-5.096498844200	-1.887652495464	1.749268835020
H	-5.340603156495	-2.132529885991	0.009287206781
H	-4.314090951898	-3.262456061694	0.928406877699

(Me, Me) complex in the ^{1}B1 state

E_{tot} = -2390.26111062519
E_{zpve} = 0.588952693231
E_{SP} = -2390.59655339

Ni	-0.032556648164	0.186294526134	-0.481360174976
P	1.388794867474	-1.464366263747	0.321653017204
P	1.688287934962	1.493533282735	-0.278954717693
N	3.211210447628	0.181260855608	1.633107512606
N	2.824420491324	-0.514626433450	-1.921523941601
C	2.295289540202	-0.935508513514	1.872009547837
H	1.537547152107	-0.716815316310	2.644107376362
H	2.886184890278	-1.790864181528	2.213744821635
C	2.558741202132	1.462763059986	1.367843398457
H	3.330795426280	2.237227751580	1.316792573802
H	1.838684452865	1.756528194646	2.146312243746
C	2.835090310254	-1.599197330398	-0.867701535902
H	3.794600676631	-1.544836500587	-0.350798233309
H	2.753576063078	-2.546562069953	-1.403347852089
C	3.060456838753	0.879817298621	-1.399074724990
H	3.102853754562	1.536763251785	-2.269510349120
H	4.020614718991	0.882854388216	-0.881628201634
P	-1.816513291933	-0.760614658367	-1.246450286150
P	-1.364360491837	0.770260848955	1.206835253283
N	-2.933002449515	1.765934372699	-0.884371317795
N	-3.121602279602	-1.382700576882	1.122029847196
C	-3.206421883278	0.459666698891	-1.492447532759
H	-3.302670671325	0.599283616536	-2.573508304133
H	-4.138322389694	-0.001034412223	-1.121823206328
C	-2.839768717350	1.736010632694	0.579627101243
H	-3.751219157069	1.338250447379	1.058080614736
H	-2.695464552391	2.763252827326	0.927603791420
C	-2.537063969569	-2.003812390514	-0.063839038907
H	-3.330118108168	-2.530507823824	-0.604789936470
H	-1.743689779262	-2.733722655396	0.171568302179
C	-2.154110413730	-0.724501166993	1.997240241158
H	-1.338156981427	-1.394487864164	2.323830262184
H	-2.682250257751	-0.376707787492	2.890714180993
H	1.869710892029	-0.503482064611	-2.319170454998
H	0.229513601335	0.367145214480	-1.938864475593
C	-1.721329514670	-1.710527867468	-2.807333804321
H	-1.370526063707	-1.048950951762	-3.602878493168
H	-1.002237398667	-2.524839792853	-2.679001909703
H	-2.699665505960	-2.124378263441	-3.069416825451
C	-0.847319165617	1.724783745350	2.687799557027
H	-0.137046325762	1.132890596503	3.270526798281
H	-0.367863821490	2.655932344150	2.376484142062
H	-1.720927232918	1.952905909771	3.306720928072
C	1.603251859833	3.278598095719	-0.675869921296
H	1.170431277349	3.397708428654	-1.672319337634
H	0.947160944261	3.761602621952	0.054144404007
H	2.595607507772	3.737982975857	-0.638211623233
C	1.054266316729	-3.241361984688	0.669942838996
H	0.381369511277	-3.306106359605	1.529373549521
H	0.559824920252	-3.688923853875	-0.196254521613
H	1.976710518927	-3.787629912946	0.891517859130
C	3.787438632739	-0.833739709069	-3.026646983479
H	3.688683902944	-0.073128147593	-3.801315957523
H	4.797060739755	-0.832412473154	-2.615185368764
H	3.538945359003	-1.816354228390	-3.427969197690
C	-3.944690299881	2.740058689788	-1.306986968166
H	-3.959273350570	2.802723556470	-2.399386129662
H	-4.960738703760	2.478642522227	-0.962002818679
H	-3.690782843551	3.727045103233	-0.908296978296
C	-3.928015519316	-2.350863012769	1.869592403541
H	-3.326874984258	-3.186147034284	2.270912952991
H	-4.420908484205	-1.848124406424	2.707305679063
H	-4.700124705616	-2.767140687620	1.215257362216
C	4.178869533931	0.310854978931	2.727342133671
H	3.702287092285	0.564866783554	3.690044772505
H	4.720915625879	-0.631616080929	2.849019384088
H	4.902705086549	1.094505655163	2.483979613391

(Me, Me) complex in the $^{1}TS_{C}$ state

E_{tot} = -2390.24009498938
E_{zpve} = 0.582498520284
E_{SP} = -2390.57536828

Ni	0.011781291506	-0.087904456529	-0.384302969089
P	-1.487229144215	1.425079931425	0.463874709950
P	-1.709240592764	-1.463791234673	-0.348838654757
N	-3.399093198786	-0.319085867048	1.506567903042
N	-2.547447535950	0.626800042549	-1.973145501511
C	-2.550651686170	0.812519734097	1.868708099429
H	-1.879792499445	0.597066287761	2.720127631181
H	-3.198281209877	1.647119318277	2.155900016682
C	-2.665523588480	-1.551439601236	1.239498654060
H	-3.383298774674	-2.369042455099	1.118102488412
H	-1.964446987066	-1.830611960258	2.044346606059
C	-2.726801033487	1.659224971070	-0.927921165006
H	-3.751910273774	1.660125507356	-0.539478150643
H	-2.515186752265	2.631624426008	-1.379634472556
C	-2.946724332017	-0.728900358588	-1.559063332412
H	-2.942092076623	-1.371272712556	-2.442577537405
H	-3.940367433035	-0.740291316087	-1.099701586864
P	1.732933519091	1.212669305989	-0.722776869602
P	1.462934872732	-1.249133859535	0.800843972915
N	2.980380871503	-1.152266529026	-1.521337628742
N	3.080655169861	0.857670038660	1.637172918557
C	3.165831561719	0.300265629986	-1.494904774757
H	3.228764691050	0.658077632493	-2.526413926978
H	4.084660817105	0.607277300560	-0.966646708955
C	2.958474315811	-1.760142500693	-0.188329803947
H	3.863124451623	-1.538205840922	0.403075735254
H	2.884066140497	-2.845051001870	-0.306978013060
C	2.434989737149	1.902149026514	0.846047314655
H	3.187049102713	2.645493811291	0.562282421214
H	1.624404768077	2.421003078281	1.383546000671
C	2.166538241335	-0.160948026304	2.146941495460
H	1.313549639385	0.257070552704	2.711498662605
H	2.726496535081	-0.817769483233	2.819889790918
H	-1.097066724595	0.392992255372	-2.048680562648
H	-0.206768218453	0.054773507450	-2.041623735837
C	1.463148750410	2.650728094313	-1.816370259625
H	1.085375796322	2.285428154757	-2.774291395341
H	0.727933852670	3.325182889971	-1.375276876028
H	2.406579357674	3.184125738828	-1.966631541668
C	1.020302092939	-2.759351542056	1.740427635305
H	0.203007165905	-2.536040599431	2.430195191121
H	0.708777565088	-3.543488797014	1.048712382312
H	1.891505307439	-3.102551829636	2.306783065606
C	-1.552721270948	-3.211850842318	-0.864119262194
H	-0.911150104255	-3.271025511044	-1.746464406497
H	-1.112135864453	-3.794034491702	-0.052587500010
H	-2.544544173207	-3.612360641475	-1.095718684837
C	-1.148089765755	3.137657682046	1.031172153244
H	-0.601931351220	3.089952208661	1.977061380895
H	-0.541045524905	3.669555484221	0.297992310101
H	-2.088617516268	3.675942406053	1.184786073198
C	-3.122993892950	1.023443976915	-3.270165194210
H	-2.864147385017	0.270112912874	-4.017930607843
H	-4.214039538441	1.116837316751	-3.211102653682
H	-2.693868929675	1.983072233172	-3.567386827234
C	4.013383180954	-1.779603788610	-2.353300861189
H	3.974361705258	-1.358695563451	-3.362446022721
H	5.031619067884	-1.630491680770	-1.953787079738
H	3.823761295132	-2.855035114697	-2.419944779058
C	3.875540955299	1.442149103559	2.720490822390
H	3.256898427750	1.983988341052	3.457100022161
H	4.420086879322	0.649876538033	3.242506795021
H	4.604117813018	2.142100775109	2.300472233695
C	-4.441404296048	-0.537112040929	2.514321435692
H	-4.029682212702	-0.815533176449	3.499823049542
H	-5.030840477384	0.376917378733	2.632149913544
H	-5.109407119864	-1.336558630310	2.180444178960

(Me, Me) complex in the ^{1}C state

E_{tot} = -2390.25639796663
E_{zpve} = 0.581514161279
E_{SP} = -2390.59365552

Ni	-0.000263100038	0.003081836265	-0.064883231723

P	1.658944092381	-1.430139057045	0.410833409111
P	1.701443240342	1.436410331372	-0.293368162053
N	3.404887293466	0.381484478886	1.602105057473
N	2.731787865124	-0.577706837837	-1.977372214524
C	2.519593434079	-0.733378351907	1.918522821036
H	1.739916640650	-0.480215923613	2.659009166280
H	3.121504309241	-1.549307293532	2.328465152200
C	2.703816806174	1.611280116071	1.257121163447
H	3.439781977306	2.400220921748	1.069481321787
H	2.020279733054	1.963866408976	2.051158411488
C	2.974605603801	-1.534648755236	-0.898920778705
H	3.961925100040	-1.419496352629	-0.421136414923
H	2.907342247898	-2.545572958506	-1.310520475200
C	2.910826706541	0.815405107815	-1.577797286212
H	2.738724602514	1.443773656163	-2.456085601492
H	3.924485748873	1.032055536049	-1.196776391100
P	-1.695470665815	-1.432856206150	-0.309068684409
P	-1.660958385573	1.430200344105	0.425286795307
N	-2.754387571551	0.592797514056	-1.960621839895
N	-3.383363108761	-0.405436676683	1.615021242963
C	-2.922018504473	-0.804450088984	-1.573871768889
H	-2.756182057037	-1.423392437532	-2.460209051835
H	-3.930396072724	-1.030426872824	-1.183824643633
C	-2.991708074467	1.535917489425	-0.869907058114
H	-3.971654409973	1.406254891559	-0.380569431159
H	-2.940021915485	2.551363475749	-1.272634462267
C	-2.673268656305	-1.626445938273	1.255104488619
H	-3.403475755673	-2.422595023557	1.078044511858
H	-1.972281459563	-1.972660218415	2.036654474431
C	-2.508521042775	0.717462301872	1.934316043369
H	-1.724234610254	0.468653901140	2.671330554976
H	-3.117937035095	1.525059869886	2.349683756490
H	0.322012982618	-0.007925149280	-2.831866623307
H	-0.390151130529	0.225528312496	-2.802554056194
C	-1.455325549441	-3.166477245391	-0.852660227240
H	-0.710290860799	-3.207613603206	-1.650305429988
H	-1.134749603990	-3.781038080831	-0.011121254778
H	-2.411865376150	-3.546260832866	-1.224388207701
C	-1.395948996752	3.170020076118	0.934012530001
H	-0.635352251836	3.217167335164	1.717068305590
H	-1.081014370938	3.764271481192	0.076191621408
H	-2.340196153088	3.568095740298	1.317890521384
C	1.457828846655	3.176335897046	-0.808524908144
H	0.702687023597	3.227122818144	-1.595886871645
H	1.144535279984	3.775428186788	0.046793330405
H	2.409642928558	3.563508853714	-1.184453834593
C	1.381122819702	-3.170061657716	0.914405243000
H	0.607236828763	-3.213781804378	1.684639954714
H	1.078607005012	-3.762267497836	0.050716988177
H	2.317679715426	-3.571056104400	1.313920554539
C	3.566402179310	-0.883688628715	-3.144047569515
H	3.312442474825	-0.202176782691	-3.961421234129
H	4.645724118755	-0.788101743902	-2.933757978796
H	3.368177730245	-1.907692307660	-3.474188326659
C	-3.600119242919	0.907783847247	-3.116491955872
H	-3.350227613337	0.236997772853	-3.943909158640
H	-4.677127965612	0.804464060889	-2.898186344061
H	-3.409526745754	1.936374347951	-3.436247893320
C	-4.337861153311	-0.656994325783	2.699316902033
H	-3.843924443508	-0.964374394763	3.637370696935
H	-4.917154768592	0.250179541041	2.893897539859
H	-5.028998188807	-1.449478645603	2.397457069348
C	4.370314852146	0.616044762325	2.680780350573
H	3.886015100796	0.915256606362	3.626431833356
H	4.947326139766	-0.295834947527	2.859872906811
H	5.062227720500	1.408807960608	2.381654952189

(Me, Me) complex in the ^{1}P state

E_{tot} = -2389.07335159901
E_{zpve} = 0.569250700127
E_{SP} = -2389.40994598

Ni	-0.000174709501	-0.001569623998	0.058388333553
P	1.684009175055	-1.436675174714	0.425213109346
P	1.682198381229	1.454334827401	-0.197713541605
N	3.488792131962	0.340744513637	1.573240087592
N	2.593247960630	-0.505662381059	-2.001034644767
C	2.629580432018	-0.789668256404	1.903020482525
H	1.894938703394	-0.567051313440	2.697655761256
H	3.256981672505	-1.616410988930	2.247075006577
C	2.758654154972	1.573289649547	1.309781433973
H	3.475832596725	2.377691369431	1.119097670809
H	2.109887732121	1.886308316481	2.148422197653
C	2.912893703998	-1.491436509156	-0.966727397413
H	3.927824860595	-1.375074073158	-0.551434630512
H	2.831043776961	-2.491407693541	-1.401448234310
C	2.814711565318	0.873014685325	-1.566860669458
H	2.600973208409	1.533366189028	-2.411836632290
H	3.851935888255	1.063446608574	-1.238954819728
P	-1.684290079396	-1.454817946153	-0.203200335590
P	-1.682368764995	1.430133284653	0.447583555197
N	-2.606971661923	0.522204935795	-1.981863991562
N	-3.479183932941	-0.358574257574	1.590621768376
C	-2.825043731650	-0.860778117212	-1.559909932972
H	-2.615825237482	-1.512775193696	-2.412529999447
H	-3.860306209486	-1.054939864234	-1.227877661604
C	-2.920459401929	1.497291757437	-0.935596334878
H	-3.932504365714	1.375771038352	-0.514802026383
H	-2.842430192428	2.501672410667	-1.360714100986
C	-2.751490486254	-1.588669693717	1.309608695408
H	-3.470468994248	-2.390591636277	1.115204554726
H	-2.097618333449	-1.910831290829	2.140793403587
C	-2.617385844050	0.768369793112	1.925539244625
H	-1.877039046791	0.537647753005	2.712527921346
H	-3.242076666606	1.591724580852	2.282394096044
C	-1.460544609817	-3.213870883068	-0.662741961291
H	-0.719533939481	-3.303272547263	-1.460084152418
H	-1.147752682173	-3.792847761729	0.206713161594
H	-2.424062095069	-3.594891655210	-1.015155332832
C	-1.450633248570	3.182709819736	0.928489904218
H	-0.736750342828	3.252221191121	1.752826304631
H	-1.094676076473	3.762076598377	0.077255313753
H	-2.417703896806	3.580563007724	1.251121032019
C	1.456284724814	3.217935453112	-0.638677888507
H	0.710651001850	3.315432272607	-1.430709556500
H	1.148702487252	3.788205470301	0.238365715964
H	2.417813845694	3.602339607426	-0.992859383452
C	1.454096350592	-3.193271545477	0.892168994120
H	0.744394462156	-3.269363525889	1.719533370216
H	1.093074510964	-3.765004760809	0.037914116387
H	2.422494804032	-3.594676712569	1.206187740554
C	3.358880469887	-0.777605210032	-3.223974792282
H	3.061731320980	-0.070836157549	-4.004135390567
H	4.448319720451	-0.690029779194	-3.070951647065
H	3.138645283745	-1.790619080720	-3.572712049164
C	-3.381000137255	0.805849154420	-3.196859947268
H	-3.088924561044	0.107034179768	-3.986016916701
H	-4.469337229854	0.716203692140	-3.037263554441
H	-3.163585558594	1.822393415905	-3.537009759803
C	-4.494017130189	-0.577009809502	2.626391934391
H	-4.054518744804	-0.854497601423	3.600071601408
H	-5.082725757757	0.335258855049	2.758588782785
H	-5.167514874254	-1.379151741608	2.310412040045
C	4.510971425232	0.549122643474	2.603816306760
H	4.078474303941	0.817157230293	3.583255725374
H	5.100580107308	-0.364366220178	2.722942727792
H	5.182308228769	1.354254591565	2.290836171199

(Cy, Me) complex in the ^{1}R state

E_{tot} = -3170.86581885764
E_{zpve} = 1.058610829279
E_{SP} = -3171.38591464

Ni	0.002335383935	-0.002426099304	0.031972598898
P	0.994491916031	1.301518632251	1.491112151858
P	-1.022105581752	1.823906802773	-0.608881534777
N	-1.657275428610	2.032100705074	2.124506318633
N	1.592376326552	2.864709254518	-0.773696501380
C	-0.277460833025	2.171808769960	2.593894177671
H	-0.217261659607	1.689988228486	3.574587987644
H	0.006068654649	3.232048685032	2.735693800065
C	-1.919019504094	2.667985804169	0.832095110906
H	-1.689863626497	3.750480878768	0.847758290043
H	-2.990468027415	2.568890988001	0.628351020907
C	1.841871302060	2.783159175274	0.666002289084
H	1.567567765846	3.719819793609	1.187014804550
H	2.918295262649	2.638683935022	0.805891101837
C	0.204979263403	3.171443174930	-1.121797100826
H	0.150889396810	3.241394910375	-2.212521151689
H	-0.115730014235	4.150168022526	-0.717098884692
C	2.309385252381	0.842565869627	2.757081777305
H	3.167168232880	0.571884718132	2.121928225885
C	2.758839208847	1.957714159468	3.720867103284
H	3.093624131157	2.840787368823	3.164801358179
H	1.906415875179	2.273778631396	4.336616800955
C	3.885258652934	1.466534560652	4.647179428414
H	4.773344161108	1.238899784230	4.040264539768
H	4.173144712218	2.265243539378	5.342395705689
C	3.467335907343	0.208011118481	5.421137225343
H	4.293258810595	-0.143104386220	6.052255974212
H	2.637938339179	0.462288232427	6.097188636430
C	3.011504213431	-0.903943651258	4.465070905781
H	2.675200161991	-1.781577653616	5.031725163212
H	3.866896975908	-1.229874835039	3.855842598864
C	1.890049664715	-0.420656407232	3.532653300214
H	0.995972946460	-0.201999256427	4.133446569662
H	1.617723116671	-1.209528200940	2.823874101460
C	-2.323593641700	2.005043334389	-1.957494015824

H	-3.160141650930	1.405486346993	-1.566936267890
C	-1.843106582557	1.337561699296	-3.260300120872
H	-0.973432328642	1.884444703023	-3.651149024906
H	-1.512095265506	0.316339059246	-3.047544621760
C	-2.950732844280	1.331249381437	-4.325362644200
H	-2.576156463991	0.880646614819	-5.253297763346
H	-3.776734818260	0.695139205846	-3.975755486966
C	-3.481004798352	2.747292879736	-4.593640433802
H	-4.297969005414	2.718166845032	-5.325375198077
H	-2.678043158684	3.352060259715	-5.039732083684
C	-3.951618812536	3.419267376511	-3.295649047980
H	-4.819569724002	2.872316112208	-2.900069468322
H	-4.288454091717	4.444098173067	-3.496673700886
C	-2.839766886044	3.430638670515	-2.231507741590
H	-2.011638632160	4.055508608331	-2.591390378924
H	-3.211128533726	3.894869658949	-1.311004553895
C	-2.594080487864	2.507906462901	3.139687716861
H	-3.622560107129	2.324675233368	2.810649900031
H	-2.491214224764	3.589077432387	3.353093541779
C	2.504253478701	3.811028410851	-1.411527264941
H	3.540878227805	3.521507959801	-1.208615271843
H	2.365210156954	4.851985126916	-1.061905393140
P	1.038694593557	-1.291108481145	-1.409018375713
P	-1.005461381028	-1.844745872318	0.655553813208
N	-1.593375897831	-2.059593696441	-2.084554167496
N	1.623467681947	-2.843595542310	0.865355269128
C	-0.204258589043	-2.181281915606	-2.530392404710
H	-0.134190084677	-1.701264671977	-3.511171517171
H	0.095914728820	-3.237939026282	-2.664359567036
C	-1.867124316170	-2.701450831047	-0.798061112734
H	-1.622084686842	-3.780517981273	-0.811586486881
H	-2.942836724592	-2.617468778222	-0.610689622330
C	1.895653595704	-2.759127859547	-0.570164144345
H	1.644050086203	-3.699959902561	-1.095084884216
H	2.971952420637	-2.598739678313	-0.692941414625
C	0.235105870240	-3.171933788083	1.189649083013
H	0.163236964195	-3.242553940728	2.279320419283
H	-0.063011464964	-4.155607202323	0.779922123371
C	2.362856945925	-0.814979073095	-2.659299725150
H	3.209457270226	-0.530685630185	-2.015272361764
C	2.840353952780	-1.924436436037	-3.616106494128
H	3.182765295141	-2.801247461347	-3.054761738776
H	2.000196127808	-2.254848653048	-4.241027240028
C	3.969314630879	-1.416942397303	-4.530560118577
H	4.846901007692	-1.174503813498	-3.914178532178
H	4.277399720374	-2.212079023074	-5.221194648371
C	3.540618186378	-0.166113226751	-5.311158897285
H	4.367744105556	0.196601273498	-5.934053657312
H	2.722273598615	-0.433926382727	-5.995374869374
C	3.057587987629	0.940101004573	-4.361748705915
H	2.714310872467	1.811817336704	-4.933344831403
H	3.901057620352	1.279612278823	-3.743426568212
C	1.933041232499	0.440477323212	-3.441807304725
H	1.050144397418	0.206514654042	-4.053208623188
H	1.638716014591	1.225772222271	-2.737744175537
C	-2.325446137410	-2.048032742127	1.983165483036
H	-3.166709680361	-1.466072496548	1.576457039150
C	-1.880256779347	-1.368993346599	3.292270875399
H	-1.004440775826	-1.895568833651	3.697224009319
H	-1.568812745845	-0.340968925345	3.082805604624
C	-3.003523571731	-1.386027651002	4.340727351800
H	-2.652416356100	-0.927149493634	5.273767877376
H	-3.837774762906	-0.767901262729	3.978617638336
C	-3.507590375621	-2.812933165609	4.602129162990
H	-4.335653970261	-2.800798999170	5.321776264037
H	-2.698709650397	-3.400451939640	5.060445920067
C	-3.944740593065	-3.494877197832	3.297872981972
H	-4.817999057535	-2.966484591626	2.888912658015
H	-4.262997104865	-4.526475457665	3.494513892129
C	-2.817259572340	-3.483561185326	2.250409160242
H	-1.982269860719	-4.091798157020	2.622572947345
H	-3.165360462859	-3.955099089858	1.324545150140
C	-2.507428208621	-2.544896012070	-3.115860167871
H	-3.543256120870	-2.374774375468	-2.803228081434
H	-2.387927148954	-3.624294123288	-3.329513533901
C	2.538951179345	-3.775716924481	1.518764870626
H	3.574179558433	-3.469266055227	1.334394743563
H	2.422917755853	-4.818485951210	1.166186615482
H	2.367132500179	-3.764465176005	2.600509477877
H	-2.337234864004	-1.995976150333	-4.048185507777
H	2.351702938893	3.796459026883	-2.496144149465
H	-2.432427570445	1.962900350050	4.075850368584

(Cy, Me) complex in the ^{1}A state

E_{tot} = -3171.83890528586
E_{zpve} = 1.091362001576
E_{SP} = -3172.34941269

Ni	0.032034372328	-0.000051872672	0.000019229685
P	1.093095642722	-1.252736376303	-1.441655324316
P	-1.135189142617	0.768405542503	-1.692248973905
N	1.467604776157	1.304849710825	-2.581505429817
N	-1.494594709967	-1.960633692648	-2.240432500768
C	1.862328716207	-0.139233888265	-2.774845342897
H	2.947951717173	-0.176075391153	-2.684973297305
H	1.579380743769	-0.421727197428	-3.790373078779
C	0.030349714575	1.585478397998	-2.934554006224
H	-0.123199248442	1.266541397795	-3.966844316636
H	-0.099155428098	2.667306148845	-2.865713730214
C	-0.090575123567	-2.301450291645	-2.466954083384
H	0.187198895898	-2.260176588434	-3.535853975929
H	0.060374024544	-3.332159588858	-2.129857546822
C	-1.873791967540	-0.632437190057	-2.723022786618
H	-2.958971104701	-0.542181090998	-2.622852162306
H	-1.630578648564	-0.486631208950	-3.790884222482
C	2.503434993356	-2.455752844200	-1.150759854305
H	2.039909469554	-3.180899416304	-0.464112241441
C	3.034120929560	-3.222331057153	-2.378173690935
H	2.219552027992	-3.722393923322	-2.912030665044
H	3.491360878742	-2.512480817752	-3.080964057601
C	4.088503316836	-4.260123181632	-1.951639698056
H	3.602488974728	-5.021174602537	-1.325003405253
H	4.477060504076	-4.779207726933	-2.836222596815
C	5.237704334294	-3.615438280197	-1.162887490745
H	5.947863547238	-4.382435908785	-0.831167709689
H	5.791081780708	-2.935261632437	-1.825887570572
C	4.710338620613	-2.817572990507	0.038741145595
H	5.536591007212	-2.318561513475	0.559694711706
H	4.245556800588	-3.504251554060	0.760250999767
C	3.670201148384	-1.779393882464	-0.406199268231
H	4.158677973558	-1.055726492131	-1.071990187258
H	3.308217615600	-1.202889771449	0.450518103245
C	-2.500703088112	2.054125789181	-1.686039903831
H	-1.991857305308	2.942400913055	-1.282490357397
C	-3.610709341224	1.657324858389	-0.695943513501
H	-4.102496751380	0.741522269567	-1.049025973608
H	-3.173957284942	1.432536773955	0.280945270991
C	-4.660565098660	2.771434637877	-0.565821219718
H	-5.453196376286	2.455508857026	0.123728492625
H	-4.186802290239	3.658024749682	-0.120514031152
C	-5.253644614327	3.144395603918	-1.931750399057
H	-5.971475342540	3.966871202134	-1.825752775183
H	-5.812734978747	2.284134416406	-2.327229039160
C	-4.150058817035	3.527317994645	-2.927235222965
H	-3.655025741912	4.446426974395	-2.582699518165
H	-4.581405711742	3.749574651618	-3.910977790798
C	-3.096076240932	2.413341185472	-3.061727745651
H	-3.570858046842	1.524106535527	-3.496768580856
H	-2.310985030420	2.729179166452	-3.757879621555
C	2.407680016150	2.234404060515	-3.281186465127
H	2.111652089739	3.259801733266	-3.057359627646
H	2.349532353897	2.047105662833	-4.353834170646
C	-2.375190860402	-2.986687112474	-2.799795109292
H	-2.123835704402	-3.961281822444	-2.369198617385
H	-2.301163890699	-3.067651054085	-3.899828552616
P	-1.135240391497	-0.768345155170	1.692332881298
P	1.093174452559	1.252639597347	1.441655492677
N	-1.494522845891	1.960722247818	2.240340698743
N	1.467503458610	-1.304942403882	2.581574676870
C	-1.873865990691	0.632590147464	2.722953137178
H	-2.959022263970	0.542383395858	2.622695305172
H	-1.630752501467	0.486863174550	3.790831480263
C	-0.090485849579	2.301362838778	2.466981451573
H	0.187199153967	2.259942597096	3.535908084359
H	0.060592158800	3.332102851794	2.130009732529
C	0.030238233123	-1.585263146447	2.934803149346
H	-0.123190737383	-1.265926492644	3.966977453578
H	-0.099452946199	-2.667082404443	2.866366825319
C	1.862475179504	0.139088112546	2.774743749797
H	2.948051258617	0.175749376670	2.684646017252
H	1.579792757150	0.421663114179	3.790287326551
C	-2.500775505306	-2.054052786408	1.686115089362
H	-1.991897873216	-2.942349102588	1.282647420117
C	-3.096278329369	-2.413207624344	3.061763885512
H	-2.311259567094	-2.729028904628	3.758010069008
H	-3.571093043048	-1.523943165727	3.496718332816
C	-4.150252627025	-3.527188401925	2.927223084310
H	-3.655191747592	-4.446325254084	2.582823285165
H	-4.581711877418	-3.749366198263	3.910925620071
C	-5.253724866581	-3.144349155153	1.931591061856
H	-5.971514616886	-3.966851973310	1.825557673131
H	-5.812885345751	-2.284089050520	2.326948809979
C	-4.660503633496	-2.771436158931	0.565719787403
H	-5.453060682133	-2.455570018947	-0.123932177309
H	-4.186669144443	-3.658029656870	0.120512333540
C	-3.610700839736	-1.657287917132	0.695916444355
H	-4.102551288201	-0.741505477969	1.048975441939
H	-3.173867686767	-1.432480543594	-0.280933339477
C	2.503506271971	2.455668094457	1.150814940698
H	2.039949008038	3.180893962774	0.464268183409
C	3.670244893514	1.779382797080	0.406139248093
H	4.158805769633	1.055722572783	1.071872342781
H	3.308239560969	1.202872102249	-0.450554184809

C	4.710259696749	2.817646911538	-0.038863444202
H	5.536466830542	2.318726054345	-0.559950556103
H	4.245340035927	3.504325605196	-0.760262204113
C	5.237718889745	3.615485246445	1.162726230595
H	5.947752023347	4.382575739619	0.830947656510
H	5.791264164404	2.935322830031	1.825592937116
C	4.088579197880	4.260005474627	1.951697072136
H	3.602426390802	5.021089203064	1.325210069144
H	4.477229596356	4.779018354063	2.836274695641
C	3.034316324570	3.222102804081	2.378267972294
H	3.491727043687	2.512161778283	3.080873345206
H	2.219798191156	3.722047047134	2.912344724098
C	-2.375023185530	2.986868362678	2.799667802573
H	-2.123691144316	3.961429343981	2.368908211435
H	-2.300867712680	3.067948427748	3.899689902736
C	2.407512664641	-2.234536904219	3.281281206588
H	2.111172008568	-3.259948500256	3.057848670205
H	2.349686747054	-2.046884436806	4.353898593291
H	3.417154021492	-2.046410819580	2.915260560793
H	-3.414444063729	2.757624379683	2.544211573330
H	-3.414563670696	-2.757454426346	-2.544201473218
H	3.417359509903	2.045929371772	-2.915499518618
H	1.532539675138	1.480049627790	-1.562473407741
H	1.532321251895	-1.480250700281	1.562549935418

(Cy, Me) complex in the $^{1}TS_{B1}$ state

E_{tot} = -3171.82006266622
E_{zpve} = 1.08479480321
E_{SP} = -3172.3301678

Ni	0.139159697193	0.110809662800	-0.048234798344
P	-0.986280632269	-0.620434894069	1.733659041497
P	1.057355990183	1.525861061321	1.288211862209
N	-1.556034145415	2.101436162653	2.051049662283
N	1.653632173547	-0.866062113004	2.635597590747
C	-1.719222186382	0.813115830836	2.724429538658
H	-2.789769753559	0.613725694092	2.813527842359
H	-1.301722521573	0.809477458165	3.746219165722
C	-0.197833893009	2.639644621445	2.117873799678
H	0.123070163121	2.858769457260	3.152585259078
H	-0.176450300018	3.580674297038	1.557672763116
C	0.260262079663	-1.343284326584	2.939239868986
H	0.049530435874	-1.103635113071	3.982930036860
H	0.269461830189	-2.427044601208	2.807838131339
C	1.814681674207	0.627131773972	2.776774915164
H	2.884091737769	0.828146625384	2.824761603934
H	1.356720236854	0.920067646530	3.722753329428
C	-2.293181627201	-1.956240315593	1.807519482953
H	-1.791726102893	-2.809481252393	1.327341767789
C	-2.726675404299	-2.372204976355	3.228223701202
H	-1.864698575930	-2.683350986220	3.827943359104
H	-3.179176968354	-1.511609799132	3.738487702646
C	-3.751392754499	-3.518703501758	3.173402806408
H	-3.266886986107	-4.409747033164	2.749599000746
H	-4.064276997343	-3.781214521564	4.191204238383
C	-4.967634755902	-3.148459596930	2.314530197284
H	-5.668055229071	-3.990816057864	2.262889525792
H	-5.504975646465	-2.315998063379	2.790889204257
C	-4.533454051036	-2.726115586930	0.904400436727
H	-5.402856684277	-2.422739969896	0.308278760338
H	-4.081539262524	-3.585986238412	0.390982117441
C	-3.513936172432	-1.577662876960	0.949437480745
H	-3.997751811041	-0.687509227014	1.371944907302
H	-3.187139855676	-1.322964544932	-0.060261005491
C	2.427715307914	2.688209877991	0.797070612044
H	1.976947938223	3.244202955850	-0.037964925619
C	3.625898877145	1.889964215880	0.246554203021
H	4.073247812115	1.293957877404	1.053547965430
H	3.291136435561	1.179685813028	-0.514151187187
C	4.698478644168	2.831493240666	-0.318288630083
H	5.549322656517	2.245756356605	-0.686918622817
H	4.284728507580	3.371123738491	-1.181092057702
C	5.159193587708	3.842338561652	0.741738795188
H	5.900404590755	4.529809716518	0.316907417404
H	5.657987546639	3.302522322187	1.559522488362
C	3.970016658105	4.629541261651	1.310675609281
H	3.531765160469	5.248405191322	0.514996896858
H	4.306217249557	5.316000277864	2.097008306759
C	2.882763965266	3.695287569560	1.870689525698
H	3.286320241481	3.151442033288	2.735300704264
H	2.035061208206	4.287192349514	2.231522559741
C	-2.523126855385	3.083559839853	2.543754273919
H	-2.419490973541	4.013873905840	1.976587357192
H	-2.391540606820	3.317525438409	3.615544276189
C	2.678972716062	-1.620451905917	3.423129228131
H	2.533760590823	-2.686531675357	3.246845931484
H	2.545883671662	-1.386310463382	4.479614565175
P	1.036673851893	-1.466333585255	-1.313054412875
P	-1.223916308744	0.594280527524	-1.734073015719
N	1.335750072992	0.937178188027	-2.604267510442
N	-1.618335416908	-2.181480098951	-1.889361935295
C	1.690022424337	-0.481791883198	-2.802251782188
H	2.776882002189	-0.565601061838	-2.821960648814
H	1.292714370949	-0.868749544068	-3.745051850894
C	-0.036273342245	1.266531450459	-3.034423733752
H	-0.243295197526	0.887861192879	-4.040278466712
H	-0.137217590592	2.354716693819	-3.034721187943
C	-0.233663970952	-2.616045654938	-2.080124256975
H	0.014354717018	-2.779119996286	-3.145021759525
H	-0.114589641817	-3.575625891981	-1.565748394936
C	-1.964790456937	-0.926952695744	-2.561835341491
H	-3.050096397038	-0.811910336523	-2.502623245845
H	-1.697175758118	-0.930521997866	-3.633304488886
C	2.447585034806	-2.640322610762	-0.944905927192
H	2.039398508810	-3.277621527858	-0.146053177839
C	2.876036519256	-3.542932627828	-2.120299267903
H	2.022084946643	-4.103532716496	-2.513968492660
H	3.251831893213	-2.914688536894	-2.939331225009
C	3.981470915337	-4.521559330992	-1.686188992017
H	3.570830302611	-5.210771031887	-0.934763430388
H	4.289952151121	-5.133672428484	-2.542411788370
C	5.189808638420	-3.785977790884	-1.090189676332
H	5.943100418727	-4.505958977904	-0.748352194418
H	5.663746268608	-3.177793166446	-1.873878399301
C	4.764216549667	-2.868843803870	0.065079014792
H	5.626499884401	-2.311612229497	0.450777585287
H	4.381719647740	-3.479684937787	0.895077519063
C	3.669356249179	-1.887844355480	-0.379975787153
H	4.079810371037	-1.222983651275	-1.151774027563
H	3.384183647383	-1.239757724852	0.455203660045
C	-2.593390231165	1.862486337208	-1.784226551831
H	-2.075905585216	2.779271556518	-1.463556098396
C	-3.684753873465	1.547862555512	-0.744288809373
H	-4.187494826505	0.610536726021	-1.016322754455
H	-3.227013065287	1.398398396882	0.237567941999
C	-4.727043878814	2.674799260475	-0.685023336335
H	-5.507707248533	2.420354145340	0.042501158267
H	-4.241921015125	3.592044152918	-0.322110467193
C	-5.345255289500	2.938527181592	-2.065333877604
H	-6.059228464532	3.769469948381	-2.012137233992
H	-5.914062050671	2.051334522345	-2.379000924890
C	-4.260719100079	3.235620546074	-3.110579966799
H	-3.757863735217	4.178370628268	-2.852210597653
H	-4.711387774320	3.377923991467	-4.100391146639
C	-3.211802445897	2.111448249945	-3.174153885466
H	-3.695555538722	1.191098200820	-3.527520862334
H	-2.436803449496	2.366927761292	-3.905189279390
C	2.342814171989	1.890340152672	-3.097752276813
H	2.091029574371	2.889626805488	-2.734142439035
H	2.374107826046	1.902943128832	-4.194128611149
C	-2.541605098669	-3.247140562370	-2.284643562544
H	-2.316196020572	-4.157242153681	-1.719824461984
H	-2.484312150301	-3.488437923096	-3.361746951909
H	-3.568363830765	-2.947157351535	-2.055100950816
H	3.324885444214	1.607947476521	-2.716563138196
H	3.668541662062	-1.311475004860	3.085015401396
H	-3.538729510560	2.703610643097	2.396909296015
H	1.799267024684	-1.073350273990	1.632900257206
H	1.068441262237	0.873477675930	-1.187707559950

(Cy, Me) complex in the $^{1}B1$ state

E_{tot} = -3171.82779908633
E_{zpve} = 1.086961571885
E_{SP} = -3172.33872577

Ni	-0.121125938225	0.227248647077	0.014930956031
P	0.992019728782	-0.721546388747	-1.681996014896
P	-1.050742822863	1.448161404915	-1.501015313935
N	1.542950398064	1.938650785522	-2.328214774768
N	-1.683731701583	-1.087379486201	-2.515714172489
C	1.688998116425	0.584059610794	-2.853850557093
H	2.755495692502	0.372141884101	-2.951836612021
H	1.237409146804	0.459256177371	-3.853347412264
C	0.188354872710	2.467320414841	-2.454334460863
H	-0.141519076649	2.560947383785	-3.504860224542
H	0.160964144439	3.464276412753	-2.003040223688
C	-0.285744735313	-1.595556165432	-2.743857340851
H	-0.077609144225	-1.504320146670	-3.811143370003
H	-0.292201028631	-2.649492406253	-2.459835533463
C	-1.850942979512	0.376763095056	-2.833726343545
H	-2.922098540889	0.575362213109	-2.864070364551
H	-1.422059319969	0.552005888332	-3.821724620334
C	2.311350140052	-2.045647100261	-1.635157445374
H	1.851312857639	-2.836711936944	-1.027712690542
C	2.670172793130	-2.622301537446	-3.021962183748
H	1.784428036171	-3.029591124120	-3.519789076632
H	3.060892906180	-1.822144593438	-3.664007025236
C	3.736331668845	-3.724292077532	-2.900471394953
H	3.314073938219	-4.571146246638	-2.341202086573
H	3.989361237932	-4.098051311937	-3.899859586512
C	4.990781139367	-3.216504696477	-2.179254392561
H	5.727148121427	-4.023232375958	-2.081394920176

H	5.460883937735	-2.427007498673	-2.782928047745
C	4.628883438751	-2.653532978836	-0.799241759944
H	5.519993392929	-2.260658370922	-0.294793639259
H	4.240573218868	-3.466209135509	-0.171481918329
C	3.567725326758	-1.546294614338	-0.898419627678
H	3.996875987584	-0.689908617701	-1.434780713192
H	3.291193274470	-1.200852578558	0.098368188180
C	-2.380124463279	2.660981332303	-1.040471826347
H	-1.847184744551	3.327205249634	-0.346394359002
C	-3.515948856414	1.969344801287	-0.262138502822
H	-4.023887666705	1.249882737697	-0.918666407535
H	-3.104644906237	1.403779607188	0.579249474639
C	-4.538017743320	2.998608356464	0.241633451246
H	-5.352713556071	2.481968318394	0.763018212008
H	-4.051492755177	3.652109061750	0.979291858464
C	-5.090444547798	3.851495033902	-0.909132505548
H	-5.788123865876	4.604736981218	-0.524076701284
H	-5.663242310987	3.207208448928	-1.591558063802
C	-3.956068630195	4.529457335956	-1.689870252265
H	-3.443440221918	5.246846680286	-1.033796812744
H	-4.359294889915	5.102061878107	-2.533442441873
C	-2.930616242276	3.505414564071	-2.206570133332
H	-3.418074425948	2.849613597141	-2.940059130403
H	-2.117593138530	4.022046395327	-2.727534703741
C	2.518588984569	2.856467868289	-2.919420180729
H	2.420665193278	3.840721082264	-2.451347606746
H	2.390394623402	2.977305182534	-4.009551808992
C	-2.692137023985	-1.929735019389	-3.237452790507
H	-2.540452177672	-2.970359404590	-2.950721430232
H	-2.543316999492	-1.803231171131	-4.309925981136
P	-1.045803501491	-1.345447140172	1.386611250139
P	1.177275039126	0.686477168560	1.783150037128
N	-1.348587403036	0.982258738604	2.946710259912
N	1.626449452524	-2.089583814407	1.855620605598
C	-1.638247894450	-0.448544659498	2.945397993814
H	-2.723472383699	-0.570191511093	2.980300935349
H	-1.223658266918	-0.962270223728	3.828818066630
C	0.059400481792	1.303079319847	3.163622886895
H	0.432377176344	0.927215232988	4.131158062704
H	0.159333078477	2.393075965295	3.169767164226
C	0.244774476558	-2.529210471566	2.054344269561
H	0.021388820361	-2.744381742921	3.115329757780
H	0.111749512593	-3.463629071293	1.498607207313
C	1.978514542331	-0.842669344150	2.535454338404
H	3.058406696388	-0.705893353414	2.433700102155
H	1.755851901863	-0.870510397689	3.616740377508
C	-2.465657721474	-2.506963189400	1.007891903427
H	-2.071107449732	-3.110932287069	0.176542508619
C	-2.877069382696	-3.459944716523	2.148091998201
H	-2.017171419389	-4.037303332969	2.502862600757
H	-3.241274912540	-2.869610888115	2.999069253993
C	-3.989512197114	-4.416607552335	1.684426440202
H	-3.590445989693	-5.071858512603	0.897174591288
H	-4.287113210474	-5.066464416990	2.516193039048
C	-5.204866133128	-3.652702678568	1.140347382903
H	-5.965080599790	-4.355121625064	0.778165135772
H	-5.664785412656	-3.079603223967	1.957918998756
C	-4.795429415263	-2.685469849620	0.020513357248
H	-5.662107063617	-2.110139608221	-0.326582814676
H	-4.427548709883	-3.259778891375	-0.841509226980
C	-3.690771336004	-1.727784323857	0.490244746327
H	-4.085663388270	-1.090556576356	1.292746297579
H	-3.411741151960	-1.047434621370	-0.321941951110
C	2.522760998816	1.979037173071	1.778358911793
H	1.971517785035	2.890097717703	1.501049587468
C	3.560023848604	1.697177977464	0.676139429900
H	4.097571083157	0.770575235776	0.914868442583
H	3.052611025198	1.543486163375	-0.281472380149
C	4.572920772711	2.847430087181	0.571428397457
H	5.316616438227	2.617764631111	-0.201535391567
H	4.049072639437	3.758165366280	0.247885166869
C	5.262528601765	3.107681184334	1.918440176850
H	5.953924112484	3.955199447981	1.836712352569
H	5.867420176089	2.229366873799	2.186143934011
C	4.233626029737	3.366271928006	3.028333447308
H	3.697135997891	4.301258210567	2.813331009299
H	4.737294629412	3.504499777783	3.992797176351
C	3.212901005187	2.219647328545	3.135575595742
H	3.732827398158	1.303333844328	3.445505083141
H	2.476608070168	2.449704344931	3.913022510315
C	-2.215183641000	1.715892621265	3.869477135109
H	-2.019970201333	2.788829908461	3.776142453515
H	-2.063902411842	1.428160587796	4.924835964124
C	2.549176155160	-3.157831597110	2.244311612698
H	2.329938756635	-4.062593880091	1.668588353747
H	2.482986721109	-3.410522218623	3.318440711207
H	3.576841057898	-2.853404273416	2.028083920149
H	-3.262850205226	1.533847892590	3.610547991003
H	-3.687808483049	-1.595675535598	-2.945434366992
H	3.530146447458	2.485189466847	-2.729692079477
H	-1.860804268849	-1.186353611509	-1.505261440627
H	-0.952144720231	1.101100363244	0.794198121950

(Cy, Me) complex in the $^{1}TS_{B2}$ state

E_{tot} = -3171.82617496639
E_{zpve} = 1.084660842216
E_{SP} = -3172.33802243

Ni	-0.270064677448	-0.065359253909	0.012830367153
P	0.857152454685	0.901638292194	1.691230614448
P	-0.975642346083	-1.451761033701	1.504003604544
N	1.658848039071	-1.679821556719	2.352420838477
N	-1.822498631234	1.037670468573	2.513985945453
C	1.649151019241	-0.323441119099	2.888825991514
H	2.682149754533	-0.002380127632	3.032159657629
H	1.149888716819	-0.254942239872	3.870652832629
C	0.363378154744	-2.339063123364	2.454843890292
H	0.034596726067	-2.473907149286	3.501445680520
H	0.439995692008	-3.329913013038	1.995568252275
C	-0.478992221631	1.676566999417	2.758390107948
H	-0.270103700789	1.606262344138	3.827167281688
H	-0.580926990747	2.725261459327	2.472574806792
C	-1.871204764383	-0.439750862939	2.824093048003
H	-2.923888460379	-0.721863429959	2.836611162074
H	-1.446550037706	-0.587237979057	3.818330436753
C	2.111047007190	2.280743025849	1.583417103823
H	1.652463345304	3.000812211676	0.891776788851
C	2.398420447522	2.988042939436	2.924642577760
H	1.479930955907	3.404289881917	3.351614315319
H	2.787125459352	2.258220318503	3.647065384224
C	3.432575175343	4.111510190572	2.739420150365
H	3.001171559384	4.890741228836	2.095399608790
H	3.641294409681	4.579880612648	3.708750962110
C	4.726901900240	3.589593474009	2.101493848192
H	5.436371205744	4.412830928645	1.954576500450
H	5.205008320578	2.875188736262	2.786787000873
C	4.433245336888	2.891610520128	0.767112225892
H	5.355579393135	2.491761684118	0.328812081678
H	4.036192502725	3.627314801873	0.055289670687
C	3.412678354497	1.755853530320	0.939535419633
H	3.856376115558	0.982871180031	1.580307469511
H	3.182738628316	1.298316130144	-0.025791740477
C	-2.163304070668	-2.826689826700	1.100840073241
H	-1.568547052492	-3.455210703405	0.421697907838
C	-3.405274537251	-2.334265388210	0.333547761287
H	-3.981528435640	-1.649219949696	0.971113776085
H	-3.098486837843	-1.772209195325	-0.554466155691
C	-4.299383649628	-3.516817610612	-0.073884550030
H	-5.189504883533	-3.140470985408	-0.591759115581
H	-3.753553119301	-4.143570127578	-0.793036717275
C	-4.700188718917	-4.368245415480	1.138468182549
H	-5.306951063827	-5.223664196935	0.818420397795
H	-5.327850868145	-3.765671418640	1.810472546062
C	-3.461549057529	-4.849970638514	1.904257630788
H	-2.879699635200	-5.528549843548	1.264925977902
H	-3.755742085679	-5.422707106164	2.791704559477
C	-2.566262207948	-3.673589482044	2.327072374673
H	-3.115075548546	-3.045324779080	3.040939364964
H	-1.682362805925	-4.054857301021	2.847539732391
C	2.708653910649	-2.500275356424	2.959226221305
H	2.717991268740	-3.486797130073	2.485920680064
H	2.570209629308	-2.639275053738	4.045926505850
C	-2.917033610687	1.785393005257	3.214694672698
H	-2.859735461407	2.833505046441	2.920610623469
H	-2.775089465977	1.680588145901	4.290505198844
P	-0.849512542104	1.440251064623	-1.460402745680
P	0.970307578602	-0.917513820662	-1.731147631025
N	-1.564467098950	-0.857899525110	-2.912025149300
N	1.866237175463	1.733144563283	-2.040280482311
C	-1.482367862358	0.594318217194	-3.031454262458
H	-2.487015700259	0.982067583358	-3.214262894062
H	-0.854892991077	0.919642053981	-3.877403934571
C	-0.266799842364	-1.533920134254	-2.998701162513
H	0.172030606579	-1.452561014051	-4.009379773251
H	-0.421502328144	-2.598092986050	-2.788839411481
C	0.588125648239	2.440013873819	-2.101058115883
H	0.339353836821	2.777207463904	-3.124122994065
H	0.658702674710	3.332069440764	-1.469112723070
C	1.883392723709	0.425799934595	-2.700707660686
H	2.926936989655	0.110329920762	-2.773364536983
H	1.490721249553	0.465386373572	-3.731150317395
C	-2.139357816827	2.725406989588	-1.060197987463
H	-1.746988036368	3.194929983476	-0.145995707393
C	-2.320131190490	3.817255321519	-2.134318676378
H	-1.368282340976	4.312466098593	-2.349992550417
H	-2.661679341981	3.352758446619	-3.069190689572
C	-3.351950205978	4.864304476654	-1.679637220430
H	-2.955610117235	5.395216636533	-0.802546411104
H	-3.487356989087	5.613315628516	-2.469036126617
C	-4.695696686600	4.219686665603	-1.314788101973
H	-5.397986152584	4.981560582973	-0.956007418006
H	-5.140274841077	3.776411458749	-2.217088982665
C	-4.512580921080	3.122811502602	-0.257242846815

H	-5.470571914453	2.637559102291	-0.035838856413
H	-4.158601693901	3.573834645236	0.680608789393
C	-3.495335741492	2.069850106793	-0.723178694564
H	-3.889385793844	1.566219659368	-1.615703818010
H	-3.378597836631	1.290635905150	0.038741133325
C	2.202713567117	-2.317113824149	-1.638404278865
H	1.631160395311	-3.116184527533	-1.143182476441
C	3.370863263979	-1.923726064367	-0.709990163568
H	3.917589341609	-1.078121836302	-1.149457281566
H	2.977595339129	-1.593096989207	0.256210941479
C	4.342772994134	-3.097843832431	-0.515763099973
H	5.179179619801	-2.784685514704	0.121143231032
H	3.823518814702	-3.906568933899	0.018052205065
C	4.859009225203	-3.629127938974	-1.860398034400
H	5.523770559774	-4.487224551710	-1.702942692167
H	5.458335447848	-2.847897569946	-2.349694623987
C	3.694518482511	-4.019555332339	-2.781172595941
H	3.149762687699	-4.863864099193	-2.335300387268
H	4.071494418072	-4.363096961164	-3.752230130492
C	2.717956176223	-2.848956781541	-2.989023888878
H	3.234701655102	-2.042518917680	-3.526021698190
H	1.884879623454	-3.173000089864	-3.621606899619
C	-2.511154369376	-1.432091575017	-3.867847786619
H	-2.600551728493	-2.508206271287	-3.688001180111
H	-2.206522500837	-1.283851903592	-4.919566472154
C	2.934254096981	2.592258902453	-2.552434167762
H	2.921323161919	3.548707159305	-2.021758989273
H	2.835076573837	2.799436755105	-3.633418380017
H	3.905087062986	2.117587194624	-2.380015057066
H	-3.497059761122	-0.977819120568	-3.727626889019
H	-3.872660620285	1.358044049307	2.910519610994
H	3.682056953844	-2.029717736760	2.791741851348
H	-1.984728894908	1.119456644373	1.497809696594
H	-1.563716917269	-0.449326206352	-0.510809690787

(Cy, Me) complex in the ^{1}B2 state

E_{tot} = -3171.83071725405
E_{zpve} = 1.086518078198
E_{SP} = -3172.34155078

Ni	-0.005521841317	-0.041235852021	-0.305781718547
P	-1.324305102015	-1.481018300625	0.862394726091
P	1.314317309287	-1.766051803568	-0.394927841435
N	0.945372860914	-1.944563499627	2.379326358546
N	-0.922453814662	-2.955128767404	-1.511418596371
C	-0.502367430658	-2.128944127252	2.406530517949
H	-0.908019463529	-1.549121635592	3.241127567653
H	-0.789242508895	-3.185603644011	2.558447692378
C	1.581068295486	-2.633735868578	1.261446292564
H	1.253784087052	-3.683162612499	1.165062970000
H	2.660064359311	-2.642938895141	1.433860646490
C	-1.567184126671	-3.029889746260	-0.203012773064
H	-1.259538463587	-3.917749979800	0.376603167988
H	-2.643292381060	-3.108532745771	-0.374121402735
C	0.531570714682	-3.073267115246	-1.462954704923
H	0.926954024301	-2.921060792736	-2.472449311981
H	0.867403916106	-4.065328427092	-1.112049068015
C	-3.078746969766	-1.174198797001	1.430191506841
H	-3.614725299471	-1.020155038121	0.483824898641
C	-3.732787173978	-2.366004808253	2.161556440825
H	-3.651779942456	-3.288442058695	1.577989576105
H	-3.210924780536	-2.539703685389	3.111118781726
C	-5.214111051087	-2.063481214795	2.449004588195
H	-5.749331880244	-1.966367318224	1.493861622956
H	-5.666218756237	-2.908957560267	2.981443491384
C	-5.381016544285	-0.770196751529	3.259245685742
H	-6.444858677473	-0.552109260278	3.412274911281
H	-4.939223679442	-0.912266971260	4.255734567577
C	-4.685820689129	0.411452770903	2.568959270300
H	-4.760391239994	1.314751950503	3.186558150781
H	-5.195018784976	0.631751851148	1.619643174110
C	-3.209735859314	0.100288151034	2.279716572992
H	-2.675944012874	-0.038434588546	3.229726867164
H	-2.731961017310	0.942729472090	1.781884360903
C	3.059322586944	-1.594652317524	-1.023586938779
H	3.573563342029	-1.117957073692	-0.179123228054
C	3.182050004852	-0.668443499991	-2.243151733716
H	2.621746218076	-1.091899017040	-3.087449884197
H	2.739678822732	0.303750792850	-2.017101572352
C	4.654973143011	-0.475224005919	-2.635025422664
H	4.718832469516	0.165279753952	-3.522828273320
H	5.168030288547	0.056387565312	-1.820787489522
C	5.357166973330	-1.816144199849	-2.885711310788
H	6.415998703936	-1.653196966859	-3.119322838162
H	4.907219105247	-2.299392390331	-3.764543845050
C	5.215815682813	-2.747555542420	-1.673671177330
H	5.747910907349	-2.313311400052	-0.815574440725
H	5.681634337512	-3.718632576866	-1.879196619428
C	3.739649961587	-2.954471623535	-1.295749136709
H	3.226934770818	-3.458980809007	-2.124357631571
H	3.664085385764	-3.611416384487	-0.422695188163
C	1.543862133259	-2.334821058486	3.656840646142
H	2.614806594201	-2.110059959860	3.644548411681
H	1.416328554814	-3.408843695386	3.880161372775
C	-1.487963216556	-3.937247966137	-2.437440164391
H	-2.563826829463	-3.765430692697	-2.542411009261
H	-1.333523248066	-4.979858624239	-2.106017106476
P	-1.383180095765	1.574129607642	-0.824563517286
P	1.342765398875	1.672830677403	0.413896162968
N	0.840344488946	2.313510270603	-2.328823798793
N	-0.907439442629	2.618974339011	1.726391796327
C	-0.640174564863	2.537131348606	-2.243162236964
H	-1.070214888354	2.159364732354	-3.172321511094
H	-0.820747236752	3.610084179502	-2.161071411800
C	1.602698609371	2.774430101089	-1.111747455458
H	1.323308424062	3.812513022525	-0.923920376629
H	2.658001467971	2.733496307305	-1.383535118055
C	-1.626099883709	2.903297308804	0.489272778842
H	-1.360845116029	3.881532928057	0.052748701661
H	-2.697063034250	2.923110809235	0.706933433539
C	0.531783989588	2.845499981535	1.620377155951
H	0.984520803247	2.626556947657	2.592376160597
H	0.781296708634	3.889980142850	1.360364399636
C	-3.101698136885	1.189878813400	-1.409905440851
H	-3.610863103969	0.902264589659	-0.481685972207
C	-3.853598433702	2.393588110438	-2.013004457563
H	-3.855159746830	3.241220097364	-1.319083034500
H	-3.345648063206	2.724465382596	-2.927987773485
C	-5.298117282886	1.992145985570	-2.357472867254
H	-5.831008653240	1.745846864187	-1.428352760306
H	-5.819246038946	2.845926000084	-2.806467160009
C	-5.336334454193	0.782363340838	-3.301936413191
H	-6.374321658620	0.493956177116	-3.506183517690
H	-4.888937175274	1.064948496151	-4.265522679923
C	-4.558418422806	-0.405091987408	-2.717118004140
H	-4.553418545231	-1.246102503402	-3.420791953947
H	-5.062201575281	-0.755845557851	-1.804894251925
C	-3.114261538793	-0.015944527430	-2.366095553911
H	-2.570732772838	0.246671737695	-3.284675174849
H	-2.586094326836	-0.862943385942	-1.918151447774
C	3.110805815607	1.576393008012	1.008261027452
H	3.624967429884	1.114561402396	0.154202018303
C	3.243851040394	0.633195200484	2.215361806764
H	2.715848799957	1.066087082317	3.076457997956
H	2.762361665784	-0.321746718725	2.004042143292
C	4.721728155704	0.424739285544	2.576749324198
H	4.800363722396	-0.235121018243	3.449154145415
H	5.221290282172	-0.089140224631	1.742746880037
C	5.427001093231	1.760624261416	2.848713898301
H	6.490657808219	1.595967645810	3.058750408919
H	4.991854101234	2.217882956670	3.748683378613
C	5.262609337256	2.726677799087	1.666259873955
H	5.789060981449	2.321747995362	0.790337513961
H	5.724693584142	3.694483914514	1.895148442494
C	3.781111565116	2.933430127026	1.304748179607
H	3.268112710785	3.415370283730	2.146791576477
H	3.699758024391	3.612226077304	0.449028430890
C	1.409423362627	2.893760900777	-3.585978217421
H	2.460268905593	2.611240699519	-3.650724185893
H	1.306445710594	3.978601692578	-3.547385713149
C	-1.473687552526	3.366552356039	2.851399877747
H	-2.532517974786	3.114024561361	2.962059760556
H	-1.387767051840	4.460152698210	2.726351130781
H	-0.954461723627	3.083628848430	3.772090414248
H	0.856244562829	2.486581048542	-4.432539087233
H	-1.023606370277	-3.819824114581	-3.421626883253
H	1.080872602156	-1.759317439436	4.464445183985
H	0.154042021559	-0.200195885428	-1.810153514759
H	0.940347613173	1.279841754941	-2.369285545894

(Cy, Me) complex in the $^{1}TS_{C}$ state

E_{tot} = -3171.81623781011
E_{zpve} = 1.079649376315
E_{SP} = -3172.32656531

Ni	-0.018514510721	-0.037424028057	-0.269890405539
P	-1.336322429688	-1.528557103305	0.776732947148
P	1.377072940377	-1.752422658522	-0.295098678177
N	0.885800320924	-1.910752219092	2.426515890556
N	-0.797759945303	-2.926161697475	-1.579073099215
C	-0.546863484437	-2.167203877458	2.347535862375
H	-1.034973622239	-1.640850335924	3.171746596052
H	-0.791084783821	-3.240942764491	2.443210190082
C	1.627462614749	-2.582412622156	1.366427549791
H	1.376756523158	-3.653316358316	1.275114303946
H	2.694706929693	-2.510672928472	1.589513874569
C	-1.541950559379	-3.039089746543	-0.328598286718
H	-1.289834086216	-3.947926159873	0.244662377179
H	-2.604890146111	-3.087501201017	-0.577042523173
C	0.644778920445	-3.058951613537	-1.407289895155
H	1.123120716927	-2.925819274781	-2.382111439848
H	0.940041909953	-4.049025513724	-1.016720886134

C	-3.090477229588	-1.216009373282	1.334750007143
H	-3.612966362620	-0.992804277847	0.396789576067
C	-3.775433850685	-2.443509490329	1.975310997758
H	-3.701303721416	-3.325839754171	1.332468157855
H	-3.276153133320	-2.689546943356	2.920652139439
C	-5.257212420364	-2.129594432364	2.249924975612
H	-5.767675015898	-1.963378989828	1.290772423165
H	-5.735102824719	-2.998671336135	2.717554451635
C	-5.421795575780	-0.887168737579	3.136430124333
H	-6.485253435440	-0.658432162313	3.274876275815
H	-5.010041872522	-1.100942594619	4.132931765000
C	-4.686877223393	0.323273102964	2.543722313410
H	-4.757804965938	1.183753990045	3.219728554901
H	-5.165947204036	0.619330216877	1.599679455321
C	-3.210197118748	-0.002073110919	2.273424878080
H	-2.714124527570	-0.228970426232	3.226114917937
H	-2.696693296875	0.866301741527	1.860258493642
C	3.118285102647	-1.547222452854	-0.920252719372
H	3.609180649952	-1.003091975585	-0.104874752809
C	3.209819936689	-0.701335511819	-2.200472298556
H	2.686739065831	-1.215366207804	-3.017652722464
H	2.710937708469	0.257384446346	-2.058210253612
C	4.677506765550	-0.466301031702	-2.588457753445
H	4.723486813781	0.117539694229	-3.515467180378
H	5.156128742062	0.140029686071	-1.806188122247
C	5.441002414780	-1.787895984369	-2.745441761082
H	6.495281724263	-1.593534962631	-2.975425330417
H	5.025981681766	-2.344253689740	-3.597815599829
C	5.322525587643	-2.647810728119	-1.479450694078
H	5.825363209318	-2.139993896802	-0.644514443761
H	5.830264568927	-3.609459571304	-1.619371478478
C	3.851967188159	-2.895275887163	-1.101973051340
H	3.369724337332	-3.470964054549	-1.901558655369
H	3.797381116320	-3.499775026144	-0.190582571549
C	1.407233476517	-2.268496828856	3.748243069493
H	2.465744931823	-1.998048598643	3.808183293528
H	1.309204429101	-3.345724727764	3.968963685615
C	-1.288097480586	-3.881137893954	-2.575932254787
H	-2.353311549541	-3.706563059460	-2.755877468510
H	-1.154917568514	-4.932313689230	-2.264823722249
P	-1.451931042103	1.601772764798	-0.730104466173
P	1.359062511098	1.707122046816	0.325435128573
N	0.716323276943	2.157142616445	-2.358127443274
N	-0.834465112976	2.651232330306	1.764264336576
C	-0.697973586187	2.519218520854	-2.188724342425
H	-1.235418751625	2.203873893894	-3.084636605567
H	-0.821099239480	3.598789298306	-2.056892110578
C	1.583323125046	2.682075238359	-1.281349607787
H	1.396551611889	3.750205743608	-1.122926578923
H	2.620641826126	2.553501444495	-1.594989787559
C	-1.665320201042	2.907957035692	0.592946114390
H	-1.484935719831	3.900302648766	0.144963954118
H	-2.715990385010	2.869061077159	0.891927652488
C	0.580758540007	2.917919351201	1.518541342349
H	1.119625163420	2.791584146475	2.461246983865
H	0.764517488428	3.947587689755	1.162544800881
C	-3.178074308309	1.215721979188	-1.289893557938
H	-3.681431378434	0.902515662824	-0.368280007894
C	-3.940544086386	2.435580730694	-1.848413166740
H	-3.939921916408	3.258649859251	-1.125878119549
H	-3.444226907424	2.799086012291	-2.756673210518
C	-5.386828157864	2.037175570394	-2.189927753230
H	-5.909658765445	1.762076736967	-1.263351150065
H	-5.915061156949	2.901913003967	-2.608534983065
C	-5.430618951990	0.855446810914	-3.169002710624
H	-6.469708175247	0.569562141675	-3.370825126015
H	-4.993813430887	1.167251772483	-4.128316161657
C	-4.643413158479	-0.346090807861	-2.626908529895
H	-4.643802031926	-1.166755536168	-3.354035239377
H	-5.134916260651	-0.723526563115	-1.718741809878
C	-3.196655674669	0.039685433214	-2.283988212807
H	-2.667752560725	0.330872337556	-3.201828537024
H	-2.659653609976	-0.819834877832	-1.871584591396
C	3.123942338005	1.590381508240	0.911188973500
H	3.615951056438	1.056737492138	0.088953601682
C	3.251616417586	0.744742333286	2.190385338512
H	2.758770473321	1.266308352727	3.022344149279
H	2.735306783509	-0.209515571114	2.073643885431
C	4.730727781825	0.520881038274	2.538436322109
H	4.809905643746	-0.068566290518	3.459733051149
H	5.197438993686	-0.072990103980	1.739502934428
C	5.478565924281	1.853389885682	2.687617022295
H	6.541670745579	1.674038213069	2.887919417650
H	5.077622422535	2.393185192703	3.557237099593
C	5.314916905046	2.727743927196	1.435866781278
H	5.810965025518	2.240434562073	0.584568267594
H	5.808242557200	3.696636647241	1.578445135273
C	3.832508312355	2.949527750798	1.086476771354
H	3.350456854248	3.512520437287	1.895868794224
H	3.752772342221	3.557735507637	0.179279598243
C	1.227326828124	2.443078648824	-3.711320530246
H	2.248638923528	2.064848802577	-3.789170505457
H	1.219073202827	3.519357754583	-3.916992943950
C	-1.316444206891	3.406607517524	2.924423687665
H	-2.355700630764	3.132810452106	3.129798224350
H	-1.266319852428	4.499247911827	2.775843917787
H	-0.713064722238	3.148805406542	3.799767607532
H	0.597265001802	1.927353673714	-4.439913642470
H	-0.750607937965	-3.731838525290	-3.517451966531
H	0.864240848558	-1.708801188733	4.515734099271
H	0.255518509880	-0.123966649888	-1.939209782292
H	0.574292213981	0.783946764572	-2.142938356486

(Cy, Me) complex in the ^{1}C state

E_{tot} = -3171.83469827523
E_{zpve} = 1.077675279084
E_{SP} = -3172.34642985

Ni	-0.001946025605	0.015453176202	-0.025310069374
P	1.391210622445	1.533626175548	0.922572572560
P	-1.407093857632	1.784896228673	-0.017353029074
N	-0.807020586882	1.760986803371	2.664637451841
N	0.765860148772	3.011414024568	-1.315853200945
C	0.616100331404	2.064485431576	2.545473325459
H	1.146966221853	1.513379376654	3.325124793474
H	0.835940203809	3.138264702741	2.683636850028
C	-1.618904826495	2.493825138360	1.695499133327
H	-1.404458708773	3.575898527055	1.680186423809
H	-2.673211237631	2.367489476362	1.953445280044
C	1.573444744382	3.084211542637	-0.103323373141
H	1.347906432842	3.966367388647	0.520012110128
H	2.625552364710	3.141478599742	-0.393531334591
C	-0.663011560769	3.150380184432	-1.064711331203
H	-1.182630938994	3.108084347270	-2.024619346147
H	-0.921371090939	4.110909293640	-0.584944595561
C	3.128371650549	1.110878833228	1.434127689087
H	3.639586513247	0.931092585248	0.482216301980
C	3.862752292012	2.263479948988	2.152354426888
H	3.834145859273	3.181114046855	1.555972932442
H	3.366267559896	2.480436356985	3.106229600690
C	5.324208631739	1.862038731323	2.419888784203
H	5.839114073367	1.733738199392	1.457530618652
H	5.835697147310	2.675379411477	2.948020497902
C	5.417898574279	0.558242771584	3.224983522263
H	6.467677720846	0.273816648744	3.363755085292
H	4.997832843277	0.724331526760	4.227086474809
C	4.643151834282	-0.577622002429	2.541812965053
H	4.671020156396	-1.485310159641	3.156208525546
H	5.122522194367	-0.825385563443	1.584198157492
C	3.184046842372	-0.175217585678	2.280128720262
H	2.682343771110	-0.001673658319	3.240994225875
H	2.642726698399	-0.988849676920	1.792724645438
C	-3.138602190359	1.643352372010	-0.679386438410
H	-3.638460705049	1.007306556100	0.059597651712
C	-3.179681992164	0.941269387757	-2.049615796225
H	-2.687342983369	1.574637636444	-2.798676502903
H	-2.623173570851	0.000793482927	-2.024364108750
C	-4.633835540356	0.692790669357	-2.475853949618
H	-4.652213752177	0.207200373911	-3.458765363935
H	-5.099685672406	-0.005384794810	-1.766157041032
C	-5.434810420199	2.001907064289	-2.507187086263
H	-6.480702332960	1.802513560290	-2.769000142677
H	-5.027608155225	2.651425277478	-3.294931156746
C	-5.357559771004	2.737306609957	-1.160943948339
H	-5.860101154999	2.137408671384	-0.389369283088
H	-5.889945070941	3.694162338298	-1.216418741945
C	-3.900959235588	2.984466924686	-0.728788138435
H	-3.420082846621	3.657917251280	-1.448880129221
H	-3.879693244003	3.482437131654	0.246433659999
C	-1.272616706048	2.006329872343	4.033332770851
H	-2.320474800529	1.704104761872	4.119277334147
H	-1.188539153259	3.066592966187	4.328265911059
C	1.213473505788	3.981254812326	-2.318156455510
H	2.270006013324	3.808262617983	-2.543710838005
H	1.093376514800	5.027001226973	-1.986079550030
P	1.416265323878	-1.524411924898	-0.872259893328
P	-1.391861604185	-1.774639989269	0.040290169774
N	-0.743365035804	-1.932315186308	-2.631249848116
N	0.791010500686	-2.839533772715	1.457682118390
C	0.682206005025	-2.188510203670	-2.464684338719
H	1.214277972703	-1.680790366184	-3.272468988678
H	0.932993191580	-3.262396190671	-2.521132397123
C	-1.569190197201	-2.595545171561	-1.628937684558
H	-1.362196109049	-3.675526689011	-1.534707442370
H	-2.617445220616	-2.480308993616	-1.915845703178
C	1.621235994508	-2.990774354501	0.265105984537
H	1.421282203048	-3.924120295380	-0.288333587541
H	2.670728248908	-3.002917951556	0.569536267848
C	-0.626456938028	-3.049909162251	1.181801976115
H	-1.171551425584	-2.974539803839	2.125205911991
H	-0.834209534986	-4.046608581903	0.752807408608
C	3.151398476938	-1.076831542225	-1.367336430948
H	3.646576274979	-0.887350700136	-0.408822503768

C	3.909118086309	-2.226107760713	-2.066261404921
H	3.883366373422	-3.137298319247	-1.459285463521
H	3.427098455291	-2.459620054868	-3.023430710020
C	5.368225410802	-1.810300838693	-2.322736702106
H	5.872900766213	-1.667617720101	-1.357018432779
H	5.893907730401	-2.621982991305	-2.839346065379
C	5.452687850837	-0.512127037578	-3.137672466245
H	6.500119938494	-0.217856449526	-3.273519552701
H	5.039080221560	-0.689989741088	-4.140460059692
C	4.663643098698	0.620241507496	-2.465375935709
H	4.689091203509	1.526307630573	-3.082338138748
H	5.134615954298	0.874534308798	-1.505244426708
C	3.205080167134	0.210078919389	-2.212127896716
H	2.704917593638	0.038851777029	-3.173669382159
H	2.662930153611	1.022294377698	-1.720378510172
C	-3.140875772182	-1.672136260639	0.668541023179
H	-3.645660854642	-1.062040077309	-0.089108830948
C	-3.234645011076	-0.960037152849	2.030445703562
H	-2.746084950353	-1.574206781122	2.798288170373
H	-2.702691094486	-0.006556028498	2.012537575140
C	-4.704906634176	-0.747290585814	2.419248099006
H	-4.760755242050	-0.249853928536	3.394743300363
H	-5.172775594059	-0.071812742710	1.689333555298
C	-5.469575479295	-2.078053820310	2.449457979312
H	-6.527295340889	-1.904862945393	2.680631675601
H	-5.065337462870	-2.704168813015	3.257396721286
C	-5.335227489356	-2.829933787249	1.117026678660
H	-5.836913690669	-2.257744557005	0.324146454482
H	-5.838510766322	-3.802344320110	1.174016490080
C	-3.861547565925	-3.037038219796	0.723579432054
H	-3.375499657595	-3.681615706824	1.466523219240
H	-3.803216168687	-3.552707378783	-0.240409576648
C	-1.174991873855	-2.271666410883	-3.989390449740
H	-2.228472083366	-2.003381564389	-4.113050657206
H	-1.058112320456	-3.344793665771	-4.220165453816
C	1.245450293558	-3.725331546098	2.534015776800
H	2.290721965284	-3.503323455881	2.768561074598
H	1.165111200468	-4.793970380571	2.269965813506
H	0.645012906195	-3.544087811636	3.430270705208
H	-0.586000491970	-1.697813719751	-4.711143231438
H	0.637800016181	3.843126607070	-3.238232495546
H	-0.681774237354	1.406120404534	4.731399812558
H	0.230061101836	1.079069976023	-2.758987569267
H	-0.153014837934	0.468873572826	-2.957625998200

(Cy, Me) complex in the ^{1}P state

E_{tot} = -3170.65201037165
E_{zpve} = 1.06473093591
E_{SP} = -3171.16311842

Ni	0.00190225	-0.00068869	0.03352647
P	1.39342402	1.54047907	0.92735174
P	-1.41454291	1.76575899	-0.00038234
N	-0.78951159	1.81623673	2.67572924
N	0.75732443	2.90382313	-1.37255074
C	0.62004278	2.16419259	2.51789538
H	1.17913684	1.69010777	3.32741373
H	0.80103313	3.25184369	2.57764528
C	-1.64305079	2.49217063	1.70056483
H	-1.48167553	3.58333195	1.66778226
H	-2.68759161	2.31661281	1.96978936
C	1.60752189	3.01843028	-0.18944786
H	1.43454867	3.94719931	0.38089619
H	2.65239545	3.01300327	-0.51004203
C	-0.65551868	3.10968358	-1.06682896
H	-1.21110538	3.09859991	-2.00681646
H	-0.84920710	4.07974549	-0.57577633
C	3.12179191	1.10994628	1.45497696
H	3.63209697	0.90063181	0.50872607
C	3.87011182	2.27227553	2.14174666
H	3.85899059	3.16955409	1.51414447
H	3.37227801	2.52885247	3.08489952
C	5.32293946	1.85613238	2.43243882
H	5.84346000	1.69126351	1.47872999
H	5.84312383	2.67643425	2.94091155
C	5.38881570	0.57489978	3.27579183
H	6.43282893	0.28056046	3.43568304
H	4.95872318	0.77505995	4.26735668
C	4.60758942	-0.56933048	2.61420599
H	4.62029529	-1.46225836	3.25032005
H	5.09323610	-0.84441632	1.66712604
C	3.15548353	-0.15818298	2.32848984
H	2.64230651	0.03717248	3.27934446
H	2.61312778	-0.97585248	1.84718237
C	-3.14443827	1.62783573	-0.66659066
H	-3.64152767	0.97779186	0.06180621
C	-3.18416562	0.94954075	-2.04846646
H	-2.69618349	1.59769235	-2.78822575
H	-2.62378188	0.01154148	-2.03984009
C	-4.63780999	0.70284540	-2.47814578
H	-4.65521347	0.23462184	-3.46941709
H	-5.09990254	-0.00962328	-1.78030103
C	-5.44397824	2.00920221	-2.48573079
H	-6.48950064	1.81003504	-2.74918786
H	-5.04074200	2.67364681	-3.26298030
C	-5.36738990	2.72175510	-1.12732220
H	-5.86620088	2.10665129	-0.36532433
H	-5.90368973	3.67726403	-1.16551908
C	-3.91130324	2.96720928	-0.69245561
H	-3.43284733	3.65452138	-1.40107131
H	-3.89186945	3.44861497	0.29092667
C	-1.23939394	2.09468782	4.04377460
H	-2.27543864	1.76245353	4.15841622
H	-1.18498716	3.16690201	4.29969764
C	1.19798650	3.82430724	-2.42581699
H	2.23992669	3.60983123	-2.68119656
H	1.12228325	4.88362637	-2.12551540
P	1.42946340	-1.52145269	-0.84117805
P	-1.38732215	-1.78647121	0.04684689
N	-0.72313394	-1.82794494	-2.62152528
N	0.78037777	-2.89657219	1.44835227
C	0.68944059	-2.15251134	-2.44463578
H	1.25192711	-1.66683231	-3.24498166
H	0.88972463	-3.23679894	-2.50475372
C	-1.57822318	-2.51733777	-1.65760117
H	-1.39841303	-3.60548086	-1.62063741
H	-2.62139477	-2.36068326	-1.94280601
C	1.64519121	-2.99994484	0.27501110
H	1.48815815	-3.92912365	-0.29924710
H	2.68599347	-2.98522115	0.60813047
C	-0.62619942	-3.11820223	1.12613699
H	-1.19306188	-3.10943514	2.05937613
H	-0.80365151	-4.09184281	0.63593315
C	3.16011162	-1.06906386	-1.34475806
H	3.65697451	-0.85459400	-0.39259128
C	3.93203847	-2.22042731	-2.02394175
H	3.92474739	-3.11884998	-1.39792768
H	3.44932098	-2.48190182	-2.97359358
C	5.38280329	-1.78455552	-2.29557839
H	5.88879900	-1.61423610	-1.33503747
H	5.92027908	-2.59717134	-2.79837412
C	5.44270370	-0.50132032	-3.13638164
H	6.48472135	-0.19298457	-3.28227894
H	5.02823719	-0.70572310	-4.13371887
C	4.63788729	0.63156096	-2.48359241
H	4.64640455	1.52525759	-3.11869554
H	5.10776405	0.91222526	-1.53024026
C	3.18814563	0.20018155	-2.21671592
H	2.69037027	-0.00196776	-3.17433935
H	2.62755078	1.00948059	-1.74233285
C	-3.13005949	-1.68048427	0.68515942
H	-3.62649630	-1.03652513	-0.04884022
C	-3.20658108	-1.01074443	2.06975815
H	-2.72119118	-1.65498445	2.81465768
H	-2.66254496	-0.06292808	2.07680577
C	-4.67178769	-0.79169057	2.47460901
H	-4.71487147	-0.32960550	3.46798877
H	-5.13352405	-0.08310821	1.77260902
C	-5.45588064	-2.11169616	2.46043578
H	-6.50930933	-1.93154127	2.70543490
H	-5.05592190	-2.77355183	3.24159199
C	-5.34174593	-2.81517713	1.10014270
H	-5.83671946	-2.20457210	0.33205854
H	-5.86230219	-3.77984321	1.12332507
C	-3.87401204	-3.03356294	0.69152864
H	-3.39678393	-3.71582209	1.40589290
H	-3.82900533	-3.51089550	-0.29295306
C	-1.14996527	-2.11423535	-3.99528356
H	-2.19012000	-1.80034519	-4.12363785
H	-1.07318873	-3.18533839	-4.25027300
C	1.21932198	-3.81301953	2.50579127
H	2.25503449	-3.58560226	2.77498442
H	1.16110741	-4.87271995	2.20290601
H	0.59335939	-3.67096207	3.39152308
H	-0.52912442	-1.54723816	-4.69502019
H	0.58551522	3.67276347	-3.31936316
H	-0.61792487	1.53869775	4.75171898

(NH_2, H) complex in the ^{1}R state

E_{tot} = -2296.2334398256
E_{zpve} = 0.408836901077
E_{SP} = -2296.59440459

Ni	0.002647615840	-0.415845470965	0.106404810193
P	1.458573968006	0.622634544724	-1.136356096193
P	1.677599478081	-1.106467151484	1.284292837765
N	2.839483340635	-1.772137533764	-1.184759376690
N	3.259817981987	1.173773154977	0.869386803042
C	2.894244296045	-0.417065105870	-1.761774784646
H	2.769606869419	-0.507415109883	-2.845707324653
H	3.848289062698	0.099273768636	-1.564447729813

C	3.079112060314	-1.860998937579	0.264683399637
H	4.033409115460	-1.403374667848	0.573745174014
H	3.106949693081	-2.922282006673	0.531132255806
C	2.409487714331	1.863643326718	-0.101705086069
H	3.057138783767	2.467602453239	-0.750274813902
H	1.659258948750	2.534295656949	0.355521919025
C	2.588459318473	0.420737717422	1.933455126672
H	1.844927349452	1.015647113541	2.493154696145
H	3.360118553083	0.088398115743	2.639274413068
P	-1.382643871494	1.035007058075	0.945107044527
P	-1.711135750781	-1.295518824879	-0.871106025157
N	-3.702732595383	-0.461144992147	0.894743811705
N	-2.077374605247	1.354318097294	-1.758103046563
C	-2.850371112174	0.275023252604	1.828479744346
H	-2.445855302162	-0.346923386142	2.647246354216
H	-3.452459851810	1.075832621563	2.275697522730
C	-3.134347080032	-1.677274635739	0.307962082609
H	-3.928859926955	-2.179289244051	-0.257896386977
H	-2.745255438097	-2.389769326393	1.058138936764
C	-2.304699863453	1.952398775455	-0.429916200967
H	-3.371568079352	2.029973066430	-0.165541856839
H	-1.885158920033	2.964645708657	-0.451179083744
C	-2.583658140261	-0.016972357655	-1.957306324061
H	-2.362564763965	-0.299785837966	-2.991482547035
H	-3.671463646720	-0.096771821016	-1.803717336537
H	-4.575349513938	-0.695173741066	1.359823688685
H	3.545996551290	-2.338908747318	-1.649933441291
H	3.894205758802	1.845840928173	1.291643397777
H	-2.534723295690	1.950177719899	-2.446597384291
N	-0.990073692400	2.248437009635	2.116004151001
H	-0.091615962001	2.677923719242	1.913622815604
H	-1.694048449232	2.977886607396	2.227591763548
N	1.699182695313	-2.182286005626	2.653478746174
H	2.536066554934	-2.116679609376	3.233804147392
H	0.882748629504	-2.026930111005	3.238672132388
N	0.913055652171	1.513680945537	-2.497997614480
H	-0.097165358444	1.662082577052	-2.382317136159
H	1.408983027958	2.377674756444	-2.706517063748
N	-1.718592428719	-2.653019129116	-1.954084992480
H	-1.114094337549	-3.391912681472	-1.606327386411
H	-2.641626863511	-3.042457580370	-2.146853356143

(NH_2, H) complex in the ^{1}A state

E_{tot} = -2297.2030489201
E_{zpve} = 0.439315792408
E_{SP} = -2297.55175286

Ni	-0.000526287700	-0.015382267243	-0.064979084849
P	1.595808156972	0.914900613640	-1.196329473317
P	1.479046159386	-0.889245320606	1.248929206724
N	2.477453686064	-1.748376821978	-1.224415299613
N	3.476484003558	0.955518644834	0.829034928760
C	2.807674973222	-0.397303045578	-1.811844585930
H	2.691786365004	-0.496289819134	-2.892698507693
H	3.844618940308	-0.174709028491	-1.558127045155
C	2.699334435842	-1.933444111945	0.257966808464
H	3.741648639480	-1.688680120823	0.464931987456
H	2.506731791663	-2.987376548832	0.467372897150
C	2.775105308789	1.860777945152	-0.080373878741
H	3.522369889317	2.361115538322	-0.706962530414
H	2.177651281012	2.634150065505	0.433253993805
C	2.692835313564	0.374515838180	1.918019470652
H	2.108568306709	1.113858107423	2.492747086579
H	3.382326657617	-0.129425232046	2.605111897384
P	-1.493365796389	1.201888350923	0.919222393630
P	-1.588789652438	-1.241394264967	-0.880890210137
N	-3.491823538272	-0.679940010468	1.044229560804
N	-2.466790159637	1.288143199507	-1.718040243454
C	-2.709454118081	0.191134787577	1.918383676430
H	-2.126543795825	-0.349325175498	2.684225436738
H	-3.399705910435	0.871783827557	2.429640479072
C	-2.789645810692	-1.814753570812	0.445747321503
H	-3.534633444147	-2.469682544671	-0.020391222870
H	-2.207739299346	-2.411353709679	1.169466652159
C	-2.704298133368	1.904823145373	-0.360074674271
H	-3.749928397836	1.737572434498	-0.099125614325
H	-2.508024726566	2.973653058971	-0.473021838384
C	-2.787023255762	-0.177935177462	-1.880813154245
H	-2.652552063414	-0.404981321063	-2.940156294260
H	-3.828722098639	-0.318739463459	-1.591232967765
H	-4.308231099885	-1.015175451611	1.546186254992
H	3.011676187733	-2.464909472119	-1.727841716915
H	4.290469721840	1.426054823424	1.212794426791
H	-2.993736887544	1.819011432339	-2.420034306080
H	1.479168291661	-1.926713005772	-1.412122312698
H	-1.465853196688	1.408173548798	-1.935689695572
N	-1.191151587239	2.474397224103	2.020911692496
H	-0.411703269601	3.060945726242	1.738867577700
H	-1.992582841346	3.045040160065	2.284530841512
N	1.140721776631	-1.937319234284	2.562456767032
H	1.869913920032	-2.020256944351	3.268760816542
H	0.252685601428	-1.711254757277	2.999494336289
N	1.457129621436	1.882349179557	-2.610717392130
H	0.615540835529	2.450474847123	-2.594735046149
H	2.264203016687	2.464432302907	-2.830776184604
N	-1.421096151029	-2.586515877249	-1.938054829229
H	-0.591584641056	-3.129327869534	-1.716523635316
H	-2.230207458554	-3.202939425086	-2.002963781572

(NH_2, H) complex in the ^{1}P state

E_{tot} = -2296.01193808869
E_{zpve} = 0.412846735653
E_{SP} = -2296.35838032

Ni	0.019848892709	-0.006325619776	0.045375604721
P	1.671686130808	0.098254061771	-1.438326641866
P	1.655088965263	-0.181030828146	1.545408520633
N	2.395139300895	-2.214137977049	-0.188270692802
N	3.529627447740	1.252474722288	0.216980066147
C	2.755377094135	-1.404735079222	-1.354518481060
H	2.547337325734	-1.978185492678	-2.262492970847
H	3.807151285448	-1.078472957847	-1.374571652014
C	2.760150482538	-1.623063713055	1.103565623311
H	3.805425922046	-1.283234087436	1.155199431846
H	2.590536754388	-2.369149828714	1.883865423106
C	2.744787989769	1.548319079613	-0.972014582376
H	3.424396132172	1.716779621019	-1.813018422248
H	2.081328034954	2.424217536457	-0.877603815758
C	2.810167921163	1.274551786660	1.485709023883
H	2.214252117711	2.186447850878	1.658152893816
H	3.528475055664	1.167876085507	2.305104345015
P	-1.651337197260	0.620932013103	1.383376383652
P	-1.608395714324	-0.556638706339	-1.354081285409
N	-3.501206896977	-1.143902346991	0.543817804488
N	-2.367966498241	2.017656762425	-0.914176661396
C	-2.799317479843	-0.780673777311	1.768536275449
H	-2.201122919536	-1.591606274981	2.213964908141
H	-3.527976503300	-0.433752344061	2.508366762860
C	-2.706141831126	-1.797157408603	-0.487367102171
H	-3.377599705686	-2.205935010089	-1.248752201183
H	-2.055841010278	-2.608521461747	-0.120382991557
C	-2.734040549067	1.870021681145	0.497894880211
H	-3.782690818835	1.577539921234	0.661819274240
H	-2.551857097823	2.823730918346	1.001180317179
C	-2.714810729205	0.872636005056	-1.763977409066
H	-2.510215447509	1.135065570913	-2.805714181536
H	-3.761472609544	0.545396492504	-1.675249132339
H	-4.317092939729	-1.706373058615	0.763655356159
H	2.855260911681	-3.118228167523	-0.268044332808
H	4.335486169344	1.868438049164	0.259826344890
H	-2.838780504400	2.841898044823	-1.281781048706
N	-1.238249117066	1.227718587804	2.921991157917
H	-0.608240946592	2.026066042007	2.861628625083
H	-2.046333935827	1.473852310944	3.493057933641
N	1.181193591582	-0.505825931977	3.133215396802
H	1.935485981644	-0.476561139872	3.816209919747
H	0.367539240177	0.033018089091	3.434688729080
N	1.377970120134	0.401729305018	-3.084577193658
H	0.499223228313	-0.018890234133	-3.398832092414
H	1.379365878730	1.397934982698	-3.296540785189
N	-1.153416997241	-1.217361962747	-2.859831515071
H	-0.568581624094	-2.044583471594	-2.744628943297
H	-1.947688731238	-1.447703959953	-3.456671487238

(^{t}Bu, Me) complex in the ^{1}R state

E_{tot} = -2861.13543797513
E_{zpve} = 0.905385938485
E_{SP} = -2861.58467695

Ni	-0.020873237999	0.004601424461	0.036923996586
P	-0.778271757930	-1.877258326201	0.862379998188
P	1.345768844197	-1.261020423314	-1.119990163098
N	1.936286163361	-2.345870000454	1.411357132544
N	-1.025955191074	-2.599557898829	-1.848086739184
C	0.609289848476	-2.921579320045	1.623943093813
H	0.438071242298	-2.953862862649	2.702650327618
H	0.544842813086	-3.963074706206	1.256339015860
C	2.382935054220	-2.369391585446	0.017830200700
H	2.417406103507	-3.398588986101	-0.386934758915
H	3.406211761581	-1.984983782630	-0.000626421386
C	-1.377148763575	-3.051458925306	-0.500538697874
H	-1.014644857052	-4.078364396460	-0.303106116502
H	-2.468396201645	-3.082525122409	-0.443514660453
C	0.412606710627	-2.580767595550	-2.117515099814
H	0.542387164332	-2.336295534658	-3.174694300381
H	0.875469003031	-3.573019632679	-1.957693526459
C	-2.162885163892	-2.081086710129	2.164117049712
C	2.661732991299	-0.698686691573	-2.390431939589
C	2.925602182659	-2.969452431431	2.285417122684
H	3.891046034728	-2.465144338624	2.169110580973
H	3.075922249594	-4.046266198108	2.077887573245

C	-1.734272938185	-3.383626796770	-2.856117941519
H	-2.813827043487	-3.321031355917	-2.681573353735
H	-1.450620542128	-4.453637327894	-2.855106297851
P	-1.311550521397	1.508996743757	-0.868425307630
P	0.728614333243	1.638508432458	1.284300808894
N	1.098094102730	2.915098059068	-1.201982857819
N	-2.013977578900	2.034756274884	1.801968301205
C	-0.325908079038	2.986194305857	-1.531488617895
H	-0.408622805998	2.969444386132	-2.621024962793
H	-0.783616449099	3.933434739638	-1.189085602923
C	1.391959508909	3.065919121612	0.224892495321
H	1.026467538525	4.032529310105	0.620698736720
H	2.479746362776	3.067823742615	0.333190129780
C	-2.391915523637	2.370194327060	0.427483320706
H	-2.395581460981	3.463505322024	0.256610818129
H	-3.416341460213	2.019866561414	0.276516615027
C	-0.688709077620	2.518645612499	2.194493347301
H	-0.570162290061	2.307332276376	3.260191055885
H	-0.594976625450	3.614226117634	2.071837255460
C	-2.565402650403	1.224453344303	-2.283147287831
C	2.054473894295	1.543233657872	2.657496278115
C	1.866151828775	3.872759402521	-1.992633553035
H	2.935542259600	3.740964038691	-1.795560064622
H	1.606345305764	4.926568438267	-1.775610150954
C	-3.035477973859	2.488118455474	2.742042329721
H	-4.002317342904	2.046369746323	2.477399638661
H	-3.156560193777	3.588285708387	2.758199389593
H	-2.778048142893	2.161562468077	3.755238936337
H	1.692612614446	3.695024253243	-3.059414120167
H	-1.526157753067	-2.980182413817	-3.852920752865
H	2.612421563338	-2.867103959500	3.330064112081
C	-3.333115963228	2.493395537162	-2.688864788030
H	-2.662150679874	3.286550589210	-3.035208372542
H	-4.023431052302	2.265365373912	-3.512377700981
H	-3.931263373044	2.891902289616	-1.862495013648
C	-1.775705465933	0.669861502233	-3.484019778019
H	-1.196883302783	-0.211647472342	-3.191295091313
H	-2.472279424008	0.376034496237	-4.280195964489
H	-1.086796758765	1.406965092226	-3.909132816088
C	-3.548002903433	0.134805522994	-1.815275890590
H	-3.009831461944	-0.775078930068	-1.534938457880
H	-4.152541439603	0.454463321269	-0.960380055250
H	-4.238411956527	-0.113125333818	-2.632202094338
C	-1.719655597415	-1.324845300394	3.431344293361
H	-0.849544996158	-1.784167006804	3.911421439152
H	-1.472422027249	-0.284382878860	3.199059040408
H	-2.536587190797	-1.327161127856	4.164814522999
C	-3.419921322122	-1.385389377396	1.607978471246
H	-4.212569104383	-1.394718588294	2.367595785713
H	-3.203476245490	-0.344040666498	1.350549363029
H	-3.814589852495	-1.884401596474	0.717197711930
C	-2.472587127937	-3.548512022560	2.503522805166
H	-1.599834769918	-4.067764289674	2.912978314424
H	-3.267854735747	-3.597977053533	3.259591582140
H	-2.819091970065	-4.106940229088	1.627924611641
C	2.341571423232	2.897076046856	3.327577750252
H	1.450982826538	3.314439542953	3.809070351081
H	3.106964718919	2.775204001144	4.106052302920
H	2.718379110521	3.636064043815	2.612690414514
C	3.338175943023	0.984548176525	2.016171009248
H	3.768738624021	1.670700107010	1.280150589313
H	4.096371597944	0.818554900270	2.792798245859
H	3.140635541101	0.029712986654	1.521425289831
C	1.554837631984	0.525216417658	3.699593870999
H	0.659032384316	0.871932430866	4.224504969064
H	1.323903277980	-0.432466237551	3.223049863218
H	2.333489158791	0.355948432760	4.454893231823
C	3.622755190248	0.262954403536	-1.667618425324
H	4.327728755333	0.692685665754	-2.391550767305
H	3.071830948379	1.083728787213	-1.199626616706
H	4.214593490363	-0.238621886440	-0.895561881499
C	1.928823226875	0.094462757222	-3.488632135400
H	2.660073779238	0.500374637107	-4.199850105824
H	1.227583573677	-0.524772893382	-4.057629624185
H	1.373291222324	0.931880570812	-3.055131451339
C	3.450686924584	-1.863343483790	-3.011170316558
H	2.801710268438	-2.557653025615	-3.554956766469
H	4.186722995089	-1.475699458319	-3.728645141702
H	4.001851374978	-2.433969193633	-2.256354997797

(^{t}Bu, Me) complex in the ^{1}A state

E_{tot} = -2862.10909861394
E_{zpve} = 0.939222970312
E_{SP} = -2862.55007112

Ni	0.000005920187	-0.056884864105	-0.000014142137
P	0.831242897650	1.088264711357	1.692708182773
P	1.961994833798	-1.043741563017	-0.147238639818
N	2.944477335213	1.593488829780	-0.070348208682
N	1.326029721448	-1.534240980204	2.557905229846
C	2.478813419325	1.910586938551	1.277066469661
H	2.310239929383	2.989057363216	1.320975509290
H	3.228748428490	1.667303126396	2.050826232783
C	3.376204891695	0.210682260760	-0.252461212736
H	4.179602302409	-0.076011740023	0.449968072544
H	3.787931955844	0.127477825166	-1.261032881448
C	1.381582817860	-0.111325313155	3.050086702571
H	2.394693897103	0.071600573781	3.411759798810
H	0.688167625865	-0.051898482956	3.888518576445
C	2.368829943501	-1.857532348922	1.519610634621
H	2.356369658828	-2.940597941023	1.405885068075
H	3.335936170296	-1.553350080214	1.922613460371
C	-0.055973222665	2.407688308391	2.738587610231
C	2.579266544815	-2.415892657175	-1.328582951720
C	3.984931549255	2.526155021719	-0.501556112818
H	4.249725947053	2.324297390846	-1.544433924009
H	4.905526805265	2.459832625813	0.106669905787
C	1.347009213418	-2.513011543727	3.688481147578
H	0.531869025501	-2.273410786597	4.371874911491
H	2.307461186174	-2.433801228303	4.198828535959
P	-1.961953068719	-1.043778962122	0.146894529886
P	-0.831230028035	1.088883352062	-1.692315622268
N	-1.326060325931	-1.533323903895	-2.558442400836
N	-2.944440380219	1.593530197733	0.070920486666
C	-2.368852134419	-1.856962605317	-1.520242343369
H	-2.356423596201	-2.940072463605	-1.406910719416
H	-3.335957594372	-1.552603633275	-1.923117206885
C	-1.381603713322	-0.110232051677	-3.050114130746
H	-2.394721685746	0.072834307842	-3.411698386201
H	-0.688203205293	-0.050525536211	-3.888538208412
C	-3.376140179616	0.210649490046	0.252587741694
H	-4.179584720826	-0.075808333529	-0.449887689618
H	-3.787810865649	0.127106976051	1.261154289093
C	-2.478774817100	1.911096994885	-1.276379378581
H	-2.310181030259	2.989581135662	-1.319907233694
H	-3.228715285336	1.668099867865	-2.050226170512
C	-2.579297084494	-2.416358089154	1.327731751808
C	0.055990086274	2.408650592380	-2.737765247047
C	-1.347073381489	-2.511685428309	-3.689366321330
H	-0.531963345913	-2.271834132979	-4.372707699775
H	-2.307545865303	-2.432312245527	-4.199650830257
C	-3.984931856001	2.526013131202	0.502431996113
H	-4.249712242760	2.323809971693	1.545246802383
H	-4.905526150440	2.459839149382	-0.105811693358
H	-3.607457561320	3.551610112262	0.439835766180
H	-1.213753607534	-3.514974077468	-3.283655414986
H	1.213720412935	-3.516156083823	3.282406095571
H	3.607405761667	3.551712937214	-0.438636050299
H	0.417513640543	-1.613949049477	2.066090281775
H	-0.417536811973	-1.613228656964	-2.066681622150
C	2.482823076230	-1.876626939404	-2.768911246627
H	2.765111123722	-2.670197259136	-3.470917537942
H	1.472192008231	-1.550518228002	-3.028366783091
H	3.155728917359	-1.031545538299	-2.941652614928
C	4.033042614637	-2.838359594587	-1.043545825874
H	4.329484187528	-3.622537432910	-1.751706596003
H	4.733207032958	-2.006473701200	-1.165058538838
H	4.158022478552	-3.246882905450	-0.035795034330
C	1.648032450720	-3.633969988758	-1.179758878300
H	1.899754742817	-4.380109518465	-1.942789505510
H	1.750640432318	-4.120009111991	-0.204539683214
H	0.594513176977	-3.368064570397	-1.306059177088
C	-4.033078858404	-2.838656998433	1.042481094973
H	-4.329581486091	-3.623084709391	1.750339928361
H	-4.733218445230	-2.006790988850	1.164274909298
H	-4.158038723315	-3.246802426420	0.034575037663
C	-2.482871219167	-1.877649433666	2.768269967422
H	-2.765267136585	-2.671461654538	3.469959506021
H	-1.472215800219	-1.551750224644	3.027894118113
H	-3.155699112972	-1.032567721567	2.941312325204
C	-1.648105007660	-3.634414839324	1.178494015398
H	-1.899946404505	-4.380861657495	1.941185492198
H	-1.750621564509	-4.120047291612	0.203063049439
H	-0.594589429186	-3.368608033141	1.305033176179
C	0.809679803662	2.936753320341	3.895322912049
H	1.727452756517	3.414726669505	3.538197143970
H	0.244371778105	3.691314412864	4.456935469162
H	1.087656579222	2.146033293080	4.599810901419
C	-0.439069432130	3.559780882657	1.790903237555
H	-1.063160103216	3.196249173449	0.970613305814
H	-1.012969959067	4.309120717749	2.349955981615
H	0.435770932547	4.063502425660	1.368502307238
C	-1.350486479859	1.774434597733	3.283138865568
H	-1.968946680510	1.394585502971	2.465731303246
H	-1.156458222537	0.956913405673	3.984643228129
H	-1.926326827236	2.537045589727	3.821863459216
C	-0.809677470209	2.938117336586	-3.894308723091
H	-1.727462912884	3.415935838970	-3.537008913723
H	-0.244387251992	3.692899728735	-4.455642011412
H	-1.087634123785	2.147653333426	-4.599093150690
C	0.439100760513	3.560410224449	-1.789685323002
H	-0.435734493743	4.063969422062	-1.367081519294
H	1.063213524328	3.196585148323	-0.969542930855

H	1.012981499242	4.309954596392	-2.348484110926
C	1.350498988803	1.775583285106	-3.282551825003
H	1.968968046824	1.395419935673	-2.465294224517
H	1.156452486010	0.958331460947	-3.984365501829
H	1.926342490957	2.538392378717	-3.820993068834

(^{t}Bu, Me) complex in the $^1TS_{B1}$ state

E_{tot} = -2862.08905031977
E_{zpve} = 0.931440965314
E_{SP} = -2862.52900372

Ni	-0.068921445057	0.110046627395	-0.145195513387
P	-0.307216495167	-0.452101615722	2.028311722152
P	-2.075894444387	0.938313944799	0.012666137119
N	-2.723977428487	-1.540083309331	1.123385014559
N	-0.820628758705	2.316795364177	2.106908557182
C	-1.912027207457	-1.394887500615	2.326372278036
H	-1.624835491452	-2.396150726103	2.654486273814
H	-2.466797415028	-0.926426448221	3.158455291328
C	-3.335699127098	-0.295145088595	0.673832664722
H	-3.950122128702	0.184177500926	1.456985120699
H	-3.999818071296	-0.530886737749	-0.160459561723
C	-0.617188911403	1.130777150280	3.010528872022
H	-1.488280148491	1.066462068007	3.664411828145
H	0.262118895766	1.353254428942	3.612914493285
C	-2.119751685791	2.273630086108	1.350836244291
H	-2.238024285985	3.250597146370	0.883773720286
H	-2.919586033637	2.125527000036	2.077706422033
C	-3.730968925627	-2.588531918014	1.291858326710
H	-4.272218093382	-2.729236120252	0.351106189373
H	-4.466168681486	-2.356746601545	2.083091025199
C	-0.653638643150	3.607767623372	2.846153230744
H	0.330423100984	3.612968251894	3.315156659226
H	-1.437905675942	3.678689305525	3.600444322913
P	1.882409528075	1.060932172766	-0.653545221742
P	0.529225898344	-1.633338114715	-1.385487713253
N	0.543234491084	0.571637634084	-3.003632965968
N	2.994994356736	-1.461773843356	-0.058175803894
C	1.768309640538	1.250557836842	-2.532529051200
H	1.670962238686	2.310450135789	-2.767529002091
H	2.657193620388	0.862207592771	-3.037913888555
C	0.683932699689	-0.895616224418	-3.117867302194
H	1.626412667883	-1.170070173262	-3.600325810003
H	-0.139621557226	-1.262303612286	-3.730281177206
C	3.353341847124	-0.107690051457	-0.473119249650
H	3.937319120077	-0.094464272554	-1.410573255106
H	3.990987541799	0.313721607904	0.307600460045
C	2.281919228700	-2.243351610115	-1.067743798991
H	2.199153001512	-3.266437092780	-0.693741920073
H	2.829183074964	-2.294277280715	-2.025900187619
C	0.000983903124	1.185052920906	-4.232375058766
H	-0.934918910360	0.693742567771	-4.501768131146
H	0.711955369856	1.079608768271	-5.060624888268
C	4.178915356763	-2.184727500505	0.409713240853
H	4.651181489792	-1.627035039187	1.224403374685
H	4.931368077786	-2.339448655896	-0.384348363250
H	3.881508009191	-3.164899834311	0.795246328765
H	-0.188001451977	2.244992054151	-4.056102295981
H	-0.734021653461	4.427243613104	2.131680416040
H	-3.237645427829	-3.531683461043	1.546324638259
C	-3.016613316459	1.794530049463	-1.389263963455
C	-0.454420589368	-3.225692234792	-1.683175941896
C	0.908302377934	-1.334903676825	3.199998324739
C	2.640577048428	2.731294806124	-0.140117953492
H	-0.077924388272	2.253137054917	1.389000221970
H	-0.165967387619	0.592614208708	-1.762606733899
C	0.359135232994	-1.424405135587	4.635827137161
H	-0.578202577764	-1.986529575537	4.687815130966
H	1.090641539959	-1.947970106702	5.263781953110
H	0.195046691120	-0.438385986828	5.081672579634
C	2.236116280122	-0.555785051424	3.189318528593
H	2.139865462234	0.447548150307	3.614787305462
H	2.971477449761	-1.097085086434	3.796886330609
H	2.628190512860	-0.473968511835	2.174473257225
C	1.155690463415	-2.750799809054	2.646693413723
H	1.911122973270	-3.246354495870	3.268338729218
H	0.255223213802	-3.371281581751	2.669190178476
H	1.530995560317	-2.715877312163	1.622466938749
C	2.921827224486	2.685310269310	1.373120998108
H	3.683478671245	1.942930772716	1.629181891694
H	3.290341438622	3.663673554422	1.703526280976
H	2.025147858494	2.453319409744	1.954137829692
C	1.615188091087	3.841749952078	-0.434148452484
H	1.990769111171	4.794280520604	-0.042174664171
H	1.443190680451	3.975044139517	-1.506420513249
H	0.645752491954	3.648799550323	0.033863028497
C	3.947339145194	3.028398029025	-0.900232218704
H	4.339360908908	4.001864332363	-0.579927365805
H	4.720892345035	2.281148519134	-0.699567386983
H	3.790842435248	3.077170559378	-1.982429213839
C	-4.396138796161	2.313301859696	-0.944357728301
H	-4.887801010103	2.799621798546	-1.795886761925
H	-5.050995457098	1.504773215606	-0.605581658713
H	-4.320894805124	3.054787222611	-0.142701954257
C	-3.190173135923	0.755639562900	-2.514115308187
H	-3.598209192679	1.249586048907	-3.403479022641
H	-2.241311779915	0.289622960794	-2.786328807808
H	-3.885262275203	-0.040779164564	-2.230918467681
C	-2.149643393088	2.966971363313	-1.884886655630
H	-2.110578566018	3.784797909076	-1.158222266700
H	-1.124068420526	2.653658217647	-2.092211055280
H	-2.578074065239	3.372063053466	-2.808881352997
C	-0.526085289456	-3.998207114788	-0.351817658831
H	-1.168085555713	-4.877795730208	-0.482825217302
H	0.454480438464	-4.354180131271	-0.021840602335
H	-0.955016645833	-3.379986404772	0.440468400983
C	-1.883027336756	-2.819346693187	-2.094345444486
H	-2.342250187829	-2.181130432083	-1.334854008826
H	-1.909398791646	-2.295683793637	-3.055222457004
H	-2.496175025015	-3.723003701594	-2.198041578196
C	0.183449427311	-4.106385405814	-2.772358505123
H	-0.419082615750	-5.014212720985	-2.902369959216
H	0.224270378458	-3.598006333369	-3.741361184558
H	1.197791507364	-4.420572210194	-2.506365295398

(^{t}Bu, Me) complex in the ^{1}B1 state

E_{tot} = -2862.09737493778
E_{zpve} = 0.933377707756
E_{SP} = -2862.53769051

Ni	-0.076788949339	-0.101743983965	0.144798377942
P	-0.782900327839	-0.190131306413	-1.986795941319
P	-2.023789958780	-0.895556839936	0.668306170986
N	-3.009017447535	1.150194146629	-0.926899928408
N	-1.160513472576	-2.856613731941	-1.129316685562
C	-2.485679639757	0.600333895305	-2.171911812047
H	-2.360816450300	1.429241249023	-2.870670922846
H	-3.172221915104	-0.123669295471	-2.643506860832
C	-3.448398969444	0.140117819168	0.027194966199
H	-4.229393978271	-0.526305810024	-0.380167453413
H	-3.872471169148	0.652802032977	0.893549669820
C	-1.153269639572	-1.998798307971	-2.366110174276
H	-2.117669829635	-2.133031109373	-2.859104893334
H	-0.365817960048	-2.392616584872	-3.007320016990
C	-2.297635018628	-2.547206292940	-0.193790618382
H	-2.320327670159	-3.346618425565	0.545068654566
H	-3.220735385604	-2.568491997611	-0.774949457672
C	0.114267552917	0.327357972496	-3.578416288696
C	-2.474744491781	-1.268977456858	2.464695685688
C	-4.069070424906	2.128208892107	-1.177732324889
H	-4.376961130212	2.580703012006	-0.230139501133
H	-4.959479919801	1.684048601306	-1.656373306919
C	-1.113910175923	-4.313907551966	-1.478205673675
H	-0.238493853050	-4.490565710992	-2.103241316615
H	-2.027481824021	-4.569486071740	-2.015402345783
P	2.044894686386	-0.806371266626	0.501482986463
P	0.703162983760	1.943229424800	0.771722061191
N	1.380631429451	0.359417099201	2.976041547534
N	2.790524639837	1.430932506217	-1.036375643430
C	2.431111949407	-0.407613307453	2.315336538233
H	2.522705681927	-1.360932883969	2.839223642595
H	3.412299525646	0.090935398581	2.365866802882
C	1.291931320069	1.755462221483	2.554838078563
H	2.246142513792	2.292654820138	2.679687933838
H	0.552906752233	2.245945792668	3.192048758902
C	3.331943580506	0.237492098273	-0.392240236466
H	4.146303592526	0.474494677475	0.315431620872
H	3.762615116478	-0.392275066765	-1.174106001291
C	2.289854189401	2.444074656938	-0.108234262307
H	2.062698194160	3.338943058414	-0.691722504351
H	3.044553129577	2.732591432332	0.644603155709
C	2.746657685493	-2.566060389500	0.312487198264
C	-0.303056899611	3.550675500209	0.810490841757
C	1.431689481890	0.230424717602	4.432095470426
H	0.566519302092	0.735751010291	4.871657858041
H	2.346596067132	0.664797849737	4.870800809399
C	3.780068240335	2.009161664283	-1.947649182467
H	4.084647411382	1.257953005326	-2.682853937532
H	4.684914178268	2.369206833268	-1.426240938050
H	3.335630188882	2.852216443412	-2.485503837278
H	1.387496396122	-0.827700210457	4.707432013214
H	-1.040014778981	-4.886941998422	-0.553776553892
H	-3.688066798228	2.921811655187	-1.827357182035
H	-0.296846570623	-2.621327353115	-0.612604665214
H	0.000701327956	-0.271788664467	1.568679789810
C	4.211863064161	-2.643454108644	0.784349450802
H	4.321893140967	-2.381906116909	1.840986440958
H	4.576810331987	-3.670697827788	0.660913903847
H	4.866017276956	-1.990281150772	0.198781385576
C	1.880155694180	-3.524817263597	1.149037509963
H	0.830951362946	-3.508852208419	0.839941139478
H	2.247144924945	-4.549615522996	1.017867584970

H	1.915844606927	-3.297650853165	2.218773089236
C	2.666335170817	-2.979888469239	-1.168883134575
H	1.644040344436	-2.951070516917	-1.555108786429
H	3.284404647460	-2.349392270601	-1.814215698353
H	3.027650672034	-4.009968171022	-1.271842335398
C	0.359218058260	1.846987161083	-3.509060004654
H	-0.572047654242	2.420949439952	-3.505080526593
H	0.941679891022	2.114110009698	-2.625338927534
H	0.928235112243	2.150774994741	-4.395618315256
C	1.469650644331	-0.399643393804	-3.625532671995
H	2.029165033139	-0.048044029975	-4.500390795211
H	2.058049401179	-0.179473909272	-2.734934778764
H	1.363625650368	-1.483824792534	-3.722462370631
C	-0.711165618014	-0.017385520245	-4.832570424260
H	-1.676857110866	0.496450692095	-4.852551244056
H	-0.151183737003	0.302944735992	-5.719387024788
H	-0.890023423514	-1.092746513395	-4.930128205808
C	0.486575050117	4.713958041860	1.435693759474
H	1.398944090350	4.941313312447	0.875287399289
H	-0.135803880053	5.617714700713	1.431181874336
H	0.763313019924	4.510231510244	2.474887999411
C	-1.585412363299	3.281017045214	1.620050815460
H	-1.376458571245	3.051223682992	2.669238684082
H	-2.218294845658	4.176601690417	1.598214779830
H	-2.153885593795	2.453309368454	1.188639707770
C	-0.699194992844	3.889062512586	-0.638775844120
H	0.166567587445	4.114440064943	-1.268381923533
H	-1.255553603459	3.065140640385	-1.092606857301
H	-1.344946111912	4.775795334718	-0.636904604340
C	-2.368881543973	0.050082429441	3.254305150292
H	-2.576406685351	-0.155829145890	4.310717109405
H	-1.366167590457	0.479156234413	3.182478713127
H	-3.092860821344	0.796642416109	2.915187003868
C	-1.449531705848	-2.271615961247	3.025849848770
H	-1.669679692960	-2.443919840884	4.085674057339
H	-1.493238948589	-3.242130064853	2.521618484325
H	-0.429159565816	-1.884986248579	2.951224081808
C	-3.899081155646	-1.841849899781	2.578516103585
H	-4.012380212864	-2.785072107232	2.034778251098
H	-4.117812233641	-2.042879877434	3.633792676491
H	-4.655054280892	-1.140177347114	2.213627766542

(^{t}Bu, Me) complex in the $^1TS_{B2}$ state

E_{tot} = -2862.09532920849
E_{zpve} = 0.9312944481
E_{SP} = -2862.53591701

Ni	-0.024550799558	0.050415746000	-0.276547823707
P	-0.977438510209	1.551730544333	1.147463464165
P	-2.042079791949	-0.259093550495	-1.028776909601
N	-2.844096819927	-0.502830806102	1.611372615701
N	-1.725252082704	2.521808830714	-1.393875522494
C	-2.569990070215	0.895344652930	1.925737917408
H	-2.438790537943	0.968284028799	3.006262376036
H	-3.406397696440	1.563088538844	1.656906859397
C	-3.325142426941	-0.713203726670	0.253267920963
H	-4.259018839476	-0.164056967933	0.037147023040
H	-3.534043759903	-1.777431738435	0.124065609671
C	-1.651972145448	2.928424661697	0.055527507648
H	-2.652035213281	3.250451641408	0.350509350925
H	-0.973500205130	3.779938164766	0.097669589439
C	-2.689576609834	1.395692830996	-1.658000107836
H	-2.825580554183	1.346566914960	-2.737203437187
H	-3.639679753034	1.662025248897	-1.192311959783
C	-0.206850001105	2.551157562257	2.567909743221
C	-2.383952434350	-1.431535193192	-2.474900619219
C	-3.767082903414	-1.097804719159	2.580522938982
H	-3.886727089945	-2.163043667877	2.361155418863
H	-4.764901685790	-0.625472496394	2.567141762830
C	-2.011960751019	3.704203568777	-2.270917667390
H	-1.261845960332	4.469536957458	-2.070984702802
H	-3.009865929033	4.074636699970	-2.035584376495
P	2.037851571085	0.437971325908	-0.925072789316
P	0.957713414426	-1.764074893262	0.851734680766
N	1.775104753901	-2.244994133844	-1.772517160620
N	2.777658311168	0.204065708558	1.750622667366
C	2.676989867606	-1.099875604828	-1.821427538714
H	2.796416413689	-0.818346840489	-2.869286547250
H	3.682159022398	-1.332896947442	-1.430184338047
C	1.702700129537	-2.884440293897	-0.459688580288
H	2.687519064313	-3.249403311238	-0.118226202313
H	1.046576101556	-3.753614762303	-0.549633121716
C	3.286123871861	0.643973113591	0.457907577054
H	4.214771782816	0.120917899423	0.166588233392
H	3.522147117372	1.708384169631	0.525976745913
C	2.498195566530	-1.229919011038	1.812514719930
H	2.323617011763	-1.480069351278	2.859948795055
H	3.358183807945	-1.836034718425	1.477016256231
C	2.465718650690	1.844731205937	-2.122738418587
C	0.169574777184	-3.002263709833	2.065433853082
C	2.109304616431	-3.222426083382	-2.809275713638
H	1.370318723488	-4.029666668733	-2.799333955698
H	3.110417576184	-3.670462423621	-2.674686717163
C	3.690617518705	0.607561961958	2.821864039965
H	3.827344190457	1.693086912723	2.795278371402
H	4.683807049297	0.131223538051	2.740877178319
H	3.259494794484	0.341070286302	3.791467040822
H	2.078404850128	-2.741426861951	-3.791960957715
H	-1.957636573735	3.382782245198	-3.311130040025
H	-3.352487246566	-1.000901503555	3.588120849902
H	-0.789256254173	2.167578054084	-1.641883596930
H	0.121607458460	-0.178095897592	-1.696929977746
C	3.961205329298	1.808964873176	-2.497316885946
H	4.240532871315	0.886854091860	-3.015271581600
H	4.178383784467	2.643955283461	-3.174241128434
H	4.606232880060	1.920467999267	-1.620425926968
C	1.606370616677	1.693993985954	-3.390613502992
H	0.536586185923	1.758711186188	-3.174232504523
H	1.854741929152	2.503859692661	-4.086371851403
H	1.786184013014	0.747939069487	-3.910260220967
C	2.143595138723	3.189496397235	-1.447360559124
H	1.111935907719	3.233841281509	-1.091186515549
H	2.798188561759	3.393620446070	-0.595700575566
H	2.285580111191	3.997929539800	-2.173858775457
C	0.193408910709	1.551283687427	3.671270970784
H	-0.672152709628	1.062255650527	4.127914970315
H	0.867484431765	0.784185102616	3.283878253859
H	0.717916489791	2.096171258149	4.464999757803
C	1.057773872981	3.247820808908	2.038640065130
H	1.553225435044	3.764250476387	2.869298880291
H	1.757173683327	2.522633688699	1.623226244460
H	0.834684910830	3.997710045299	1.274555917086
C	-1.186529057576	3.598926844394	3.128381483301
H	-2.103603403656	3.148898279130	3.520143283999
H	-0.700889949747	4.127803892947	3.957474156121
H	-1.462066833825	4.347993371024	2.379315177539
C	1.153195933786	-4.112766276133	2.477353966033
H	2.047659734054	-3.716954459818	2.967777307821
H	0.658905231743	-4.784539249036	3.190551233568
H	1.469730453066	-4.717276853981	1.621773859917
C	-1.062319398588	-3.628505688890	1.388604828645
H	-0.798860291701	-4.216359057189	0.504512425711
H	-1.557215873003	-4.303315788308	2.097796217994
H	-1.782239270110	-2.863561864200	1.093977471745
C	-0.293874575197	-2.213576957416	3.305602970919
H	0.541250890356	-1.785176679710	3.867769489068
H	-0.975595528849	-1.407730870238	3.023801329175
H	-0.829144252602	-2.891364621387	3.982054481793
C	-1.932490175153	-2.838173526782	-2.038991909658
H	-2.066471299929	-3.527234638072	-2.880999177835
H	-0.876198802314	-2.845527479614	-1.757608512909
H	-2.521723353386	-3.221338555353	-1.201531260450
C	-1.551837770040	-0.998473202549	-3.695166175589
H	-1.741605271351	-1.703147219602	-4.512908792159
H	-1.814996906967	0.000036022054	-4.057320985183
H	-0.480610656694	-1.018554737568	-3.476682774550
C	-3.882222234046	-1.438594730001	-2.834265102154
H	-4.234828046349	-0.456797869127	-3.166466934827
H	-4.042590110460	-2.141145098376	-3.660354349403
H	-4.507846236533	-1.765890190820	-1.998750330190

(^{t}Bu, Me) complex in the ^{1}B2 state

E_{tot} = -2862.09714419465
E_{zpve} = 0.934570362545
E_{SP} = -2862.53782441

Ni	-0.098117293252	-0.047233288864	0.266971097182
P	1.357909502932	1.729713802230	-0.314313885916
P	1.765336002276	-0.988835076483	1.023603154570
N	3.013866275311	-0.300966283164	-1.363295976397
N	1.805023889305	1.480516158338	2.474119073337
C	2.953162936477	1.142471543167	-1.142722281461
H	2.983466790799	1.621254483178	-2.123118460716
H	3.820292309800	1.518647886996	-0.573594808534
C	3.220339948248	-1.086635577433	-0.153060203757
H	4.140118450562	-0.805023153654	0.388990142440
H	3.326250619937	-2.135676509717	-0.440333715060
C	2.052877231839	2.400133332322	1.305714191830
H	3.128688716502	2.579063408188	1.260015074673
H	1.548349958245	3.334609870811	1.550672489885
C	2.477415709574	0.137023993605	2.370168125228
H	2.335118142951	-0.345851243023	3.336463016327
H	3.542506891290	0.317375803598	2.219918957411
C	0.959366789825	3.338512010258	-1.242582836730
C	1.714099793422	-2.668066262396	1.898090549349
C	4.025353824484	-0.634194461202	-2.368747793410
H	3.994124522190	-1.708389134558	-2.575030868203
H	5.049363048517	-0.371201428074	-2.050178405981
C	2.142880736772	2.154536351517	3.770586157165
H	1.586273660394	3.090164201130	3.826176074959
H	3.216085397770	2.345553860691	3.793291875958
P	-2.184083805253	0.279832156259	0.798445747723

P	-0.964552685544	-1.057924099472	-1.582394353753
N	-2.203167562087	-2.489068908291	0.476647804515
N	-2.447818805671	1.328562764818	-1.757876839282
C	-3.049317128417	-1.384675511325	0.917834215437
H	-3.293030675500	-1.541677681233	1.970266294905
H	-4.002293425206	-1.327421507825	0.363320834799
C	-1.988076002613	-2.510388360523	-0.968008610178
H	-2.935643014156	-2.550448258479	-1.533680533219
H	-1.426083419133	-3.415669682545	-1.207250274445
C	-3.160388240403	1.221037833865	-0.492937127959
H	-4.151398469607	0.744170374662	-0.599181562906
H	-3.319077956670	2.229531661741	-0.106104542936
C	-2.255010629543	0.040682726371	-2.414709027094
H	-1.895744257436	0.238488987386	-3.424887022563
H	-3.195647968663	-0.529897101660	-2.510388068312
C	-2.643503356580	1.160148019625	2.415211416327
C	-0.074615668985	-1.797213582392	-3.097405101861
C	-2.723324270834	-3.774048366275	0.946855717281
H	-2.029425859949	-4.571659238213	0.664252000603
H	-3.717506269301	-4.016127095507	0.530926254810
C	-3.111112198900	2.283945165656	-2.646982739987
H	-3.177181833325	3.256947774605	-2.150506906497
H	-4.130822568437	1.969703710365	-2.931719750882
H	-2.520354430728	2.405150032483	-3.559782855231
H	-2.801527570770	-3.759521267786	2.038350598256
H	1.853677909228	1.493377361729	4.587876827393
H	3.806557602564	-0.101785941200	-3.298972325401
H	0.790815927761	1.289545601763	2.474055518219
H	-0.173597731602	0.127865280250	1.699837459530
C	-4.176445720472	1.267422278205	2.545364241084
H	-4.658425140619	0.285048481236	2.565184089710
H	-4.417491991683	1.772218534660	3.488240640464
H	-4.621791354720	1.854699384276	1.737400374731
C	-2.083054290597	0.378338689580	3.617063017316
H	-0.991411373180	0.307485076405	3.593081945093
H	-2.360859516202	0.904733133620	4.537406954905
H	-2.488130750763	-0.635886083248	3.683637184092
C	-2.017324708892	2.565267576973	2.391236791372
H	-0.933112224384	2.519969148224	2.254534126796
H	-2.429626741711	3.192811979052	1.596284295992
H	-2.217885382599	3.064304144297	3.346347482789
C	0.608754505656	2.948184329200	-2.692876667914
H	1.470753579615	2.555661001605	-3.240487761453
H	-0.192558445302	2.205505871879	-2.722063068759
H	0.262376096732	3.841948229362	-3.225407148452
C	-0.288607441939	3.952335665348	-0.581148923362
H	-0.596353289587	4.837818490708	-1.150247401296
H	-1.119228113980	3.242050938026	-0.579570257665
H	-0.102432781156	4.273987199551	0.448105625326
C	2.125291585115	4.341235926181	-1.231042436828
H	3.029735598762	3.927020006719	-1.687643216419
H	1.841517067391	5.229816741958	-1.808649953982
H	2.373233893418	4.674645593529	-0.218556384122
C	-1.083115897387	-2.475301954588	-4.046034453741
H	-1.834714717871	-1.778224274507	-4.428250804878
H	-0.538894220911	-2.875541264188	-4.910336609168
H	-1.601064462648	-3.312527077847	-3.568850673982
C	0.950319117609	-2.835225644531	-2.612184843255
H	0.476841073993	-3.684150399962	-2.111760381915
H	1.499804130240	-3.226302107172	-3.476938299660
H	1.669974028289	-2.386640311143	-1.928726322734
C	0.660596523440	-0.664312219457	-3.837665161081
H	-0.025415816923	0.075590413219	-4.259568023522
H	1.365378635321	-0.154956099459	-3.178946359643
H	1.227035311627	-1.096072670673	-4.671750201945
C	1.276752253495	-3.712842868515	0.855545086677
H	1.155294658273	-4.680871719068	1.355927664870
H	0.319811319346	-3.441496019565	0.404163211367
H	2.015136585730	-3.842499471443	0.060152119214
C	0.638533675136	-2.587392928148	2.997688326714
H	0.562150352391	-3.564322241253	3.489311344088
H	0.879353830157	-1.850030531793	3.770011396868
H	-0.339111008110	-2.341696855001	2.572627528928
C	3.081673630862	-3.049036402865	2.490625501868
H	3.417544874399	-2.339614215986	3.253017103279
H	3.003059324839	-4.032659974189	2.969072094978
H	3.854394854613	-3.117749453964	1.718554894013

(tBu, Me) complex in the S_0/T_1 minimum energy intercrossing point

E_{tot} = -2862.06405164062
E_{SP} (S_0) = -2862.50153623
E_{SP} (T_1) = -2862.50361782

Ni	-0.022659081493	-0.189346197377	-0.358247182915
P	1.464770134965	-1.038446526746	1.317542771344
P	2.078770988507	0.683235011178	-1.159170487177
N	3.744500941422	0.582094816218	1.002004064183
N	2.557472499075	-2.102141184794	-1.067388599859
C	2.694943577039	0.235124983893	1.957707498156
H	2.122660193086	1.110695665631	2.296800954053
H	3.169625068407	-0.207805028630	2.835387737900
C	3.317381686949	1.514419483997	-0.040593511414
H	4.190380707074	1.781961716830	-0.642574537411
H	2.886046484137	2.443112098582	0.360703497842
C	2.606808144945	-2.235850638969	0.429258859640
H	3.641919848425	-2.113974455150	0.751304443802
H	2.265389794149	-3.247129816902	0.654860107266
C	3.116038227509	-0.819418955028	-1.615958657538
H	3.124581394727	-0.930098778176	-2.699766173339
H	4.137601487654	-0.708932307111	-1.252101953064
P	-2.263038410625	-1.123681373228	-0.643715934880
P	-1.317558003082	1.547349869915	0.769610794418
N	-2.161766979051	1.343398579374	-1.875778137191
N	-3.771902784729	0.209999239370	1.221390057856
C	-2.989479469090	0.128790607594	-1.824891439542
H	-2.987000725317	-0.316985747678	-2.820461272497
H	-4.035439840173	0.338647174286	-1.552884718061
C	-2.295243261346	2.198354618852	-0.687083177890
H	-3.343250155823	2.374318141483	-0.403059271507
H	-1.842458120454	3.162892234057	-0.926814994395
C	-3.545882538652	-1.138209867536	0.709313585097
H	-4.486706829745	-1.497840097602	0.286818512570
H	-3.230688451456	-1.850670929278	1.491999553243
C	-2.615986129929	0.778555852806	1.908201215721
H	-2.107750887928	0.040158674557	2.547216648464
H	-2.974195217486	1.573398900209	2.562829831613
H	1.524714147657	-2.044432674636	-1.317458116450
H	0.301825600438	-1.327832532932	-1.470100590599
C	-2.435125289747	-2.815863038007	-1.490304807388
C	-0.856343923515	3.127959570970	1.730520857079
C	1.988256342753	1.702357362757	-2.741900281999
C	0.963607533772	-1.986251537176	2.878700282096
C	3.166006983457	-3.288860619905	-1.740973969064
H	4.231464270032	-3.321583903056	-1.507476849341
H	2.669540866152	-4.188543002144	-1.376044902227
H	3.015335461493	-3.190326543177	-2.816826047756
C	4.946771627015	1.071996802197	1.684254730408
H	4.771249046967	2.009733179846	2.239271822089
H	5.302193977105	0.314233093396	2.388803534783
H	5.735235899069	1.250338906294	0.946988607810
C	-2.447923085386	2.111338580151	-3.092990681489
H	-2.283116134099	1.479982037669	-3.970744979896
H	-3.485025380043	2.487284497382	-3.124111577364
H	-1.767465872481	2.965266780619	-3.154703628129
C	-4.968460608187	0.250590467178	2.067002715412
H	-5.828407883980	-0.119692337093	1.500462539551
H	-4.868419919302	-0.359964138160	2.981958591766
H	-5.174218356582	1.283796988590	2.362601915693
C	-3.909998221557	-3.237752412719	-1.627252761152
H	-3.961342219422	-4.160976642162	-2.217104031788
H	-4.374564608515	-3.443535110921	-0.658760585310
H	-4.506006582631	-2.478579287285	-2.145361920513
C	-1.673583568874	-3.830120542157	-0.617207895372
H	-1.759924648260	-4.828900808285	-1.061926753845
H	-0.610513082843	-3.577792772524	-0.552087634973
H	-2.078885232024	-3.880019442689	0.397898941185
C	-1.797715219905	-2.771980537896	-2.893152491965
H	-0.773935067959	-2.389728970190	-2.865480110453
H	-1.772047700204	-3.792171117157	-3.294596597764
H	-2.378522060994	-2.162890298559	-3.592563318293
C	-0.181248037941	-2.930794098173	2.480432151661
H	0.138146068834	-3.662177345368	1.732209287853
H	-1.032990264786	-2.377390900540	2.074593994038
C	-2.059006212447	4.078620834121	1.882955577425
H	-2.902322179303	3.617528325748	2.405336185193
H	-1.746832511914	4.948933566236	2.473640023737
H	-2.414601023780	4.449327102020	0.917106201606
C	0.266840124520	3.855965183917	0.975087338011
H	0.579602474652	4.734138772155	1.552844762577
H	1.140229903058	3.215193415502	0.841092736889
H	-0.056557589041	4.206290051605	-0.008925385905
C	-0.344085540918	2.704763827449	3.120675193649
H	0.496135590573	2.009252154694	3.048757955257
H	0.002982385339	3.593994253645	3.660485869019
H	-1.123045438540	2.235622582780	3.729140294898
C	1.327587489289	3.040355459477	-2.357770814902
H	1.946752405328	3.620668586827	-1.666813305485
H	1.183240800796	3.640709362472	-3.263701825212
H	0.349365725720	2.881042175726	-1.896139904553
C	1.069088696059	0.946085271835	-3.721332978822
H	0.932785008883	1.556196981989	-4.621994009802
H	1.494528525424	-0.014010154393	-4.031703871056
H	0.085691665530	0.762219715322	-3.279088862454
C	3.365158980164	1.951983150119	-3.377962936438
H	3.229102885238	2.537886324368	-4.295023882674
H	4.026628829990	2.522217108453	-2.718660376436
H	3.870707589201	1.021586080365	-3.655144411404
C	2.123414501404	-2.790823819518	3.492088224433
H	1.776561883732	-3.248422524201	4.426485885536
H	2.458663345604	-3.601269787738	2.838101473591
H	2.987300593834	-2.164370870584	3.735110342842
H	-0.522351332132	-3.482781303051	3.364418019018

C	0.456514081363	-0.946877044882	3.897243678782
H	-0.318028654476	-0.302536781316	3.474030268549
H	0.023305935487	-1.473692612107	4.755368788709
H	1.264153884210	-0.311062317818	4.271512122585

(*t*Bu, Me) complex in the ^1TS_C state

E_{tot} = -2862.03445439684
E_{zpve} = 0.928016691201
E_{SP} = -2862.47611697

Ni	-0.015720441561	-0.086633632336	-0.367489809694
P	1.084516627983	1.948008127009	0.654874482038
P	-1.670572502056	1.475662918868	-0.841526820840
N	-1.457618322795	2.369253267917	1.789806149334
N	0.621664564471	2.517480277481	-2.023905557494
C	-0.059038121578	2.761112369275	1.891069407447
H	0.303420699704	2.448086712432	2.874372930807
H	0.069815077548	3.856088783814	1.818698339443
C	-2.068581721235	2.711517932257	0.512949616438
H	-1.796879122271	3.719120900384	0.155609637883
H	-3.151486106685	2.698779050269	0.635241760976
C	1.081512930698	3.167875246630	-0.789942734866
H	0.476673621897	4.050911958450	-0.545768706151
H	2.104133902418	3.507172575929	-0.942145421720
C	-0.827444025444	2.507677412536	-2.165478632334
H	-1.063510118840	2.028854365754	-3.117849075537
H	-1.258851498443	3.517143892171	-2.157790232133
C	2.777994391700	2.313166926033	1.475263431518
C	-3.327119763921	1.067962841686	-1.697714442453
C	-2.223723807684	2.917169714218	2.911954639073
H	-3.250790119902	2.542602190576	2.874383206601
H	-2.253688355862	4.020896218338	2.911328356331
C	1.303412672491	2.989376608740	-3.235588084936
H	2.382686541993	2.881837177346	-3.105175790671
H	1.072748279919	4.041848503986	-3.454685243792
P	1.532211801793	-1.721875287864	-0.702759249235
P	-0.970312952034	-1.737009541512	0.944727987937
N	-0.776048039835	-3.033380426273	-1.501496018533
N	1.617322783150	-1.927331050963	2.026578550965
C	0.652611590888	-2.957463247552	-1.780749314675
H	0.791649880338	-2.622874382325	-2.812777203801
H	1.158636758411	-3.931728353493	-1.661332936285
C	-1.056955360754	-3.309141997026	-0.096382387448
H	-0.406780859385	-4.085381377512	0.337255765185
H	-2.077291163847	-3.678152509756	-0.015988849673
C	2.039991175606	-2.655204335467	0.837894904539
H	1.659059889014	-3.687896816338	0.789437101837
H	3.126785979674	-2.722190461123	0.830095416894
C	0.211076926353	-2.145388636938	2.333881591028
H	-0.068578826693	-1.498528728317	3.170600514845
H	0.016834009293	-3.191121776235	2.627283290541
C	3.170753149290	-1.416330917423	-1.632312150554
C	-2.642760436339	-1.802688095556	1.883587398510
C	-1.425784854907	-3.994989260154	-2.394665910961
H	-2.506163011699	-3.978521119660	-2.223776436720
H	-1.063270793869	-5.027671447975	-2.249800561202
C	2.449689995894	-2.244593488783	3.189054017777
H	3.494334916949	-2.008919045428	2.966630423568
H	2.383961154273	-3.306460757028	3.483773105942
H	2.133080680985	-1.631895095848	4.038475284730
H	-1.237356038419	-3.707805833198	-3.433808917247
H	0.990954181774	2.374964065390	-4.084600177168
H	-1.773839613443	2.587822119919	3.853411429218
H	0.606370102678	0.840247211271	-1.808559336392
H	0.302911465117	0.038257816235	-1.943173924399
C	3.615756206896	-2.717072089558	-2.333449142802
H	2.969357788993	-2.988524567690	-3.171579643602
H	4.623669862728	-2.554700448480	-2.733055340410
H	3.668257404734	-3.561891925922	-1.639010963096
C	2.968091262004	-0.307594456412	-2.676822597839
H	2.767428607823	0.656991067675	-2.205685616834
H	3.884860996581	-0.209892806524	-3.268907080423
H	2.147572450622	-0.536913301642	-3.364469648067
C	4.269173329349	-1.013993444841	-0.626885385088
H	3.910355875232	-0.336128783475	0.145620393948
H	4.704870540923	-1.887703055510	-0.134934577328
H	5.076539203043	-0.510837612590	-1.169273782814
C	3.028647789746	1.196716250210	2.503128785161
H	2.239663531203	1.163676169495	3.263147214236
H	3.071914058431	0.213912998748	2.031599955787
H	3.979183875160	1.380771525269	3.018225974623
C	3.879611532315	2.318753241962	0.394025045198
H	4.848623596715	2.110606814208	0.861466277857
H	3.718669420862	1.575421052282	-0.386814647951
H	3.958404528784	3.295954009507	-0.091137504548
C	2.803236474906	3.683679186989	2.180464066931
H	2.159236828374	3.719890951140	3.062644193917
H	3.829142912195	3.878377683185	2.517386520918
H	2.518184370246	4.497413263430	1.505109452892
C	-2.631725210667	-2.937757310838	2.931019954847
H	-1.965886509906	-2.733522849682	3.772715976014
H	-3.648005306602	-3.026883074836	3.333712772462
H	-2.365423028586	-3.906382728535	2.496115007409
C	-3.789438351465	-2.101091337347	0.893258299033
H	-3.874682259929	-3.172044371620	0.690373133003
H	-4.736678765792	-1.783699968665	1.342364168712
H	-3.684160148290	-1.590701736327	-0.062463837330
C	-2.857573780670	-0.462833396774	2.598547028470
H	-2.061546515649	-0.260187576843	3.322305189952
H	-2.882237888808	0.373010762375	1.902281692775
H	-3.807037562104	-0.495979611789	3.145613214843
C	-4.451128545470	0.906763904840	-0.655290879013
H	-5.279888847122	0.353490047142	-1.110505488932
H	-4.144601333532	0.371658636631	0.239714097652
H	-4.841005800004	1.880433886180	-0.345142813091
C	-3.094705182905	-0.227186627903	-2.496973307541
H	-4.020031019921	-0.505041523639	-3.014582225034
H	-2.315467917650	-0.089804526614	-3.255164755479
H	-2.789533229057	-1.059215397655	-1.857970181959
C	-3.759033643228	2.199237369240	-2.657260447187
H	-3.119809089224	2.282182483451	-3.539028356060
H	-4.771123060332	1.969390486693	-3.010891530366
H	-3.797352950819	3.171224062443	-2.155063689174

(*t*Bu, Me) complex in the ^1C state

E_{tot} = -2862.04568820305
E_{zpve} = 0.928989519105
E_{SP} = -2862.48736387

Ni	0.004332928719	0.014783038883	-0.036809345695
P	-1.016560344601	-2.008714298172	0.481390270650
P	1.673267355391	-1.422288164181	-0.814522019860
N	1.501346799014	-2.405009512515	1.720174656326
N	-0.563993717879	-2.485677473275	-2.168505372642
C	0.095053872947	-2.765145391454	1.792211378110
H	-0.295673480893	-2.419602938263	2.752239189877
H	-0.056342859225	-3.857710315743	1.735210074540
C	2.097044897677	-2.746359141769	0.434687231729
H	1.793700845678	-3.736623737786	0.056716614263
H	3.180654558106	-2.763471288766	0.540944986998
C	-0.990636375047	-3.192207132816	-0.965588778343
H	-0.375620220343	-4.075374102182	-0.727346180588
H	-2.011492187739	-3.543898177600	-1.110403060270
C	0.879943898355	-2.335135109836	-2.248301927558
H	1.116574053805	-1.756413417996	-3.142773496849
H	1.396694103370	-3.308195604391	-2.323273462686
C	-2.718154330566	-2.293190094228	1.305367262213
C	3.357266328606	-0.942582560663	-1.587525640056
C	2.242462094528	-2.977304256930	2.845516129404
H	3.277377076389	-2.624300426601	2.815197153775
H	2.246671932216	-4.081226968632	2.839549202535
C	-1.088946320857	-3.102391316882	-3.388277436343
H	-2.181406648478	-3.132319337431	-3.338743405213
H	-0.716170741528	-4.129580891770	-3.545141389123
P	-1.641816832985	1.582945334903	-0.549743974809
P	0.981618375336	1.874714436836	0.955222108152
N	0.637259916682	2.920249428225	-1.540516845987
N	-1.586062031691	1.986881292788	2.147160911488
C	-0.801246992284	2.796152947920	-1.709954756459
H	-0.997777774564	2.433005073342	-2.719930595741
H	-1.320510889644	3.762625859680	-1.584859460565
C	1.000831056407	3.349528690871	-0.194491867692
H	0.355573463149	4.150432349519	0.201745816464
H	2.018732469875	3.736996684277	-0.210687673106
C	-2.142177423508	2.590729860571	0.942426881865
H	-1.851426976048	3.644507977446	0.800799937197
H	-3.229185438282	2.564685576762	1.006465800037
C	-0.187547261321	2.328822529990	2.351841522153
H	0.166985323700	1.788374977360	3.233070813676
H	-0.047037805214	3.408942324290	2.534152792151
C	-3.272697481298	1.263014689367	-1.497207724335
C	2.649358839467	1.963779331488	1.880314531512
C	1.209512947847	3.783275740541	-2.575604413669
H	2.298027438480	3.803742565160	-2.469523506764
H	0.832034723945	4.819499563311	-2.528336669122
C	-2.374871792479	2.306432962525	3.338652465855
H	-3.404061347948	1.964551363363	3.195217982630
H	-2.391187353213	3.386922116237	3.564529612865
H	-1.955948758866	1.781436071235	4.202495107507
H	0.967365483765	3.374061852961	-3.561145045425
H	-0.800828781953	-2.496721854305	-4.252890806224
H	1.792586587736	-2.640676740703	3.784548979188
H	-0.132857236470	0.165818793186	-3.110826861753
H	0.371975744222	0.619030984860	-2.808426363450
C	-3.735683848064	2.536090086177	-2.239595883771
H	-3.088206402551	2.806643621465	-3.076071411185
H	-4.732366491092	2.335401665014	-2.650704956010
H	-3.823600364636	3.395098195268	-1.566631116430
C	-3.006651469155	0.143832613919	-2.519664341274
H	-2.582716858508	-0.751642891716	-2.061252016336
H	-3.947204358968	-0.126332794861	-3.013251803535
H	-2.309546271927	0.479317803446	-3.292978099164

C	-4.395776803588	0.891564501060	-0.506475096701
H	-4.072831621275	0.235104671063	0.296207910392
H	-4.827277912898	1.786375039745	-0.049008439811
H	-5.199328035421	0.387291999014	-1.053547724543
C	-2.946258790267	-1.155193328841	2.315054574341
H	-2.190908078562	-1.169146488067	3.108323036876
H	-2.907602643453	-0.168758708064	1.853042409132
H	-3.926751860565	-1.284096902397	2.787644443466
C	-3.822256164712	-2.346116742869	0.228613029145
H	-4.792229638463	-2.159741100478	0.701998936846
H	-3.692069324358	-1.618833759217	-0.568650457526
H	-3.872721001900	-3.336644590083	-0.232239000840
C	-2.751550854342	-3.647140600858	2.049086006860
H	-2.124746191440	-3.663442568226	2.943137241152
H	-3.784748467645	-3.820917779922	2.373059330687
H	-2.465408696533	-4.480794981206	1.399927891966
C	2.655246633286	3.132217957730	2.891114340237
H	1.986506681761	2.970146669791	3.738966132367
H	3.672697360974	3.221987061897	3.290409373574
H	2.400989450516	4.086117796474	2.417696699802
C	3.791881269620	2.228536038083	0.879373439625
H	3.853039844702	3.290046181868	0.623889153518
H	4.744062409643	1.951823254356	1.344518206705
H	3.696347867399	1.669798396037	-0.047693083580
C	2.831822962386	0.633949529817	2.631714576639
H	2.067456566707	0.506792500916	3.405959867898
H	2.764834854010	-0.230926345245	1.970648098539
H	3.809810435953	0.627512680235	3.126381334358
C	4.426885857726	-0.807449578480	-0.482907481423
H	5.269432622184	-0.229778114108	-0.877450144320
H	4.072556043530	-0.309984181061	0.415416912424
H	4.815054077734	-1.786493504692	-0.188819668541
C	3.168024474703	0.369047696192	-2.370704868920
H	4.141778673114	0.714399046195	-2.736425115054
H	2.522048202636	0.217272377983	-3.239843601094
H	2.724641804153	1.161375953181	-1.763487204027
C	3.849855224304	-2.045112066513	-2.551903379914
H	3.248668966444	-2.121927407190	-3.460133767442
H	4.870789826256	-1.786867171311	-2.857814391971
H	3.889085920078	-3.026629614544	-2.068476525980

(^{t}Bu, Me) complex in the ^{1}P state

E_{tot} = -2860.86522294227
E_{zpve} = 0.913734534976
E_{SP} = -2861.30719375

Ni	0.00153984	0.00287929	0.03855691
P	-1.00302385	-2.02872774	0.52263848
P	1.63796893	-1.40246130	-0.81573757
N	1.53991098	-2.44841162	1.69290371
N	-0.63122933	-2.46609924	-2.13752636
C	0.13253361	-2.79732324	1.80289280
H	-0.23035262	-2.44926782	2.77301918
H	-0.03154363	-3.88812782	1.74823053
C	2.09934241	-2.76045779	0.38315426
H	1.79001436	-3.74263785	-0.00985799
H	3.18600959	-2.77439148	0.45882011
C	-0.99923715	-3.19982848	-0.93247865
H	-0.35460642	-4.06930717	-0.72487718
H	-2.01474419	-3.57728586	-1.04772961
C	0.80326309	-2.25942476	-2.26092764
H	0.98225061	-1.62551439	-3.13296146
H	1.34772020	-3.20857760	-2.41085904
C	-2.69433483	-2.29344057	1.36678928
C	3.28337372	-0.88437394	-1.63074225
C	2.30977001	-3.04629618	2.78459800
H	3.34584081	-2.69950416	2.73023022
H	2.30625909	-4.14992662	2.75843160
C	-1.16842795	-3.08678664	-3.34963969
H	-2.25758671	-3.15321292	-3.27167945
H	-0.76602320	-4.09937257	-3.52702739
P	-1.60780244	1.56044603	-0.55261875
P	0.96768340	1.87869026	1.00348916
N	0.71202094	2.87005073	-1.51478349
N	-1.62478578	2.04616460	2.12713667
C	-0.71555089	2.70867963	-1.73963753
H	-0.85580448	2.28239971	-2.73599663
H	-1.25268534	3.67302735	-1.70136133
C	1.02100709	3.33337876	-0.16772957
H	0.35791732	4.13857282	0.18943072
H	2.03671853	3.72712826	-0.15450997
C	-2.13807564	2.62316999	0.88982502
H	-1.83408708	3.66990658	0.72643497
H	-3.22705387	2.60753243	0.92182568
C	-0.22577127	2.36599401	2.36555789
H	0.09780554	1.82911268	3.26091623
H	-0.07002159	3.44518518	2.54058367
C	-3.20593017	1.22507669	-1.53965409
C	2.62235919	1.94351964	1.95068626
C	1.30442792	3.72186601	-2.54720782
H	2.38852924	3.76151192	-2.40674765
H	0.90957723	4.75271707	-2.53189135
C	-2.44144766	2.40791369	3.28710866
H	-3.47261339	2.08256596	3.12105938
H	-2.44313253	3.49331965	3.48863884
H	-2.05831540	1.89446745	4.17428192
H	1.10113882	3.29099364	-3.53236815
H	-0.92317011	-2.46345370	-4.21492474
H	1.89012815	-2.72425116	3.74260020
C	-3.64637397	2.48025668	-2.32473026
H	-2.97545091	2.72840889	-3.14962842
H	-4.63216024	2.27132352	-2.75742345
H	-3.74999872	3.35564128	-1.67537918
C	-2.90137569	0.08275585	-2.52357831
H	-2.54260586	-0.81578869	-2.02068481
H	-3.80894043	-0.16460869	-3.08627543
H	-2.13146460	0.37620157	-3.24593856
C	-4.35460512	0.87067696	-0.57235818
H	-4.04641848	0.24451466	0.26101587
H	-4.80940145	1.77436725	-0.15677543
H	-5.13544994	0.33823635	-1.12570876
C	-2.90488483	-1.13519013	2.35695573
H	-2.13157499	-1.13057007	3.13341789
H	-2.88019947	-0.15887603	1.87198788
H	-3.87429712	-1.25701053	2.85364678
C	-3.80934910	-2.35583805	0.30170880
H	-4.77290630	-2.15356494	0.78176594
H	-3.68189543	-1.64237792	-0.50870749
H	-3.87185847	-3.35343891	-0.14214508
C	-2.72468426	-3.63435692	2.13280966
H	-2.08581563	-3.63832345	3.01857189
H	-3.75425686	-3.79825681	2.47291074
H	-2.45019881	-4.47959768	1.49369176
C	2.61960320	3.08937475	2.98633922
H	1.94022458	2.91006295	3.82247697
H	3.63237974	3.16751804	3.39961298
H	2.37364231	4.05406277	2.53061412
C	3.77852822	2.22282583	0.96942142
H	3.85380483	3.29034277	0.74434555
H	4.72256648	1.92169414	1.43609632
H	3.68577407	1.69151306	0.02545807
C	2.78830108	0.59548738	2.67170297
H	1.98870423	0.43575379	3.40352255
H	2.76715175	-0.24985737	1.98248173
H	3.74168481	0.58789329	3.21237043
C	4.38633232	-0.75815846	-0.55952290
H	5.20308583	-0.14915139	-0.96168778
H	4.04817074	-0.29607452	0.36458231
H	4.80516829	-1.73666838	-0.30857379
C	3.03422680	0.44583333	-2.36130399
H	3.96502721	0.77925494	-2.83445302
H	2.28206509	0.33317294	-3.14992807
H	2.68253812	1.22954601	-1.68957191
C	3.75806323	-1.94856787	-2.64475310
H	3.12860621	-2.00479578	-3.53532419
H	4.76507307	-1.66637233	-2.97476466
H	3.82566249	-2.94433496	-2.19445796

(^{t}Bu, Me) complex in the ^{3}A state

E_{tot} = -2862.02153317018
E_{zpve} = 0.934102840863
E_{SP} = -2862.46180915

Ni	-0.002150391249	0.015631992946	0.208144380018
P	0.738878035265	1.885181314803	-0.887032163989
P	2.300915726892	-0.102471864005	0.882931704794
N	2.963102523122	0.309007682371	-1.734913081249
N	1.414805570483	2.465547843977	1.739504329269
C	2.419604968767	1.665613439089	-1.756385250856
H	2.247807747713	1.932185372695	-2.799526268647
H	3.127893939585	2.409185450665	-1.351800266402
C	3.612349243635	-0.041643849426	-0.469278180890
H	4.450591077021	0.635841945534	-0.221847037156
H	4.016978101090	-1.053642660758	-0.562077106161
C	1.169142291557	3.160874760421	0.428706371275
H	2.052634092148	3.760368821843	0.201239104706
H	0.310159394126	3.816945852804	0.573414213861
C	2.633727816886	1.582303339968	1.709058108290
H	2.940100802484	1.429148002875	2.741877318757
H	3.423520442471	2.131933503109	1.195690256675
C	3.868894832057	0.084206347826	-2.863312475850
H	4.206448072201	-0.956907414063	-2.854484946599
H	4.760418577220	0.736053988524	-2.838091064570
C	1.440536259501	3.423599697066	2.883752877737
H	0.522076120052	4.011406758184	2.863592723633
H	2.311193791564	4.072385522661	2.782018073539
P	-2.330915303217	0.074818269302	0.855335375993
P	-0.703751740230	-1.846347262882	-0.947789238279
N	-1.393900247213	-2.496357959397	1.668922936169
N	-2.943886904866	-0.278611921462	-1.783796752021
C	-2.618371976581	-1.616634133490	1.681962258863
H	-2.884015039232	-1.467666543952	2.726807304290

H	-3.422955193164	-2.172666545985	1.197952754516
C	-1.151848923668	-3.148184646448	0.334292553994
H	-2.040063813653	-3.731450571552	0.085715239072
H	-0.298778330709	-3.815284775465	0.461226851042
C	-3.624042186007	-0.011498851471	-0.515305581373
H	-4.422520493371	-0.744328791240	-0.298709644657
H	-4.085585777764	0.977953277686	-0.578034697153
C	-2.359704046374	-1.615077897054	-1.857719424829
H	-2.145309551079	-1.818952908466	-2.907151645879
H	-3.058412580576	-2.399233700103	-1.517828387590
C	-1.412797985789	-3.500303814453	2.775830974120
H	-0.488865676594	-4.078243635612	2.732449418649
H	-2.277369422069	-4.152172944022	2.646222740425
C	-3.831838002790	-0.024902569836	-2.920218769823
H	-4.202838050786	1.003404687995	-2.866948635195
H	-4.702460440054	-0.704360531618	-2.950266293516
H	-3.274467069736	-0.138238274970	-3.855516757987
H	-1.477021083552	-2.964850643641	3.724403428342
H	1.498548361208	2.849614073107	3.809844294585
H	3.338565630926	0.262738961883	-3.804131862703
C	2.993119913062	-1.306266389525	2.184349392749
C	0.289785584435	-2.845034559944	-2.225028130648
C	-0.236451021274	2.900095469993	-2.162150858394
C	-3.084429897663	1.246262808515	2.151538972185
H	0.599277606173	1.803551053396	1.878693093772
H	-0.581899009277	-1.844898248995	1.832598078665
C	0.504530380366	4.182419163910	-2.578139141227
H	1.480900500599	3.977137139854	-3.026910252887
H	-0.093639551446	4.717280338934	-3.326441814692
H	0.651640176283	4.859633851865	-1.730302578833
C	-1.601733670560	3.261054051122	-1.555257820968
H	-1.512501572885	3.942712461511	-0.705018806738
H	-2.210601678039	3.763517471055	-2.316846655867
H	-2.138075269369	2.367129344947	-1.230020790410
C	-0.469389193334	1.984870835447	-3.382315032884
H	-1.084612082741	2.521085464403	-4.114952223984
H	0.462004043543	1.702243093982	-3.882615119356
H	-1.002122680344	1.073117511824	-3.096079304555
C	-2.930384778945	2.678684157121	1.609916780360
H	-3.495876624667	2.835132779999	0.687291623400
H	-3.306603704449	3.389972535864	2.355084634417
H	-1.879860105346	2.917762153370	1.414269330291
C	-2.287573324827	1.135492965400	3.466216032854
H	-2.683187245824	1.865608598784	4.184055457953
H	-2.367148030121	0.146700894582	3.929003703460
H	-1.224382102578	1.345923770772	3.308186779400
C	-4.570164335517	0.941078906659	2.413182794094
H	-4.953973606908	1.639703462069	3.167509088676
H	-5.178874354756	1.063284151239	1.512339579092
H	-4.725631972319	-0.072331760202	2.797453440245
C	4.485086817381	-1.057133851716	2.465891490995
H	4.836614558539	-1.777970806639	3.215000707720
H	5.099423694963	-1.186686392028	1.569911146704
H	4.668399173567	-0.054641135474	2.866397242871
C	2.787829663376	-2.727487387451	1.630865686310
H	3.098197543269	-3.460378125516	2.385509399826
H	1.733431462882	-2.910096354451	1.394848878508
H	3.379297283069	-2.908148426685	0.729302793444
C	2.184628977843	-1.175015389874	3.489977817490
H	2.279642942646	-0.188276599841	3.954197793320
H	1.119582223578	-1.365221469688	3.320868366153
H	2.556214080468	-1.913493097784	4.212193481184
C	0.581681351018	-1.895362820348	-3.405458111363
H	1.196257289693	-2.425730610163	-4.143011034437
H	-0.326535201204	-1.562208364026	-3.917162771069
H	1.136878949988	-1.013487351360	-3.072360090414
C	1.626310175080	-3.261682096750	-1.591360480816
H	2.172887862040	-2.395469406914	-1.214620096454
H	1.495116609272	-3.973906798604	-0.772146369452
H	2.247229815871	-3.748312266696	-2.353388459777
C	-0.463978306854	-4.097640466341	-2.707209109054
H	0.153356482815	-4.623470371299	-3.446226566697
H	-0.660964584698	-4.795761114839	-1.886843579200
H	-1.416020327219	-3.858091659235	-3.189349444559

(*t*Bu, Me) complex in the $^3TS_{B1}$ state

E_{tot} = -2862.01661692449
E_{zpve} = 0.928826879879
E_{SP} = -2862.4561269

Ni	0.036167636168	0.138882757921	-0.276174720310
P	0.782286650123	-1.677341372543	1.146359581994
P	2.336418437817	0.164912043652	-0.835653774801
N	2.776593276070	0.202229719910	1.881259375527
N	1.698589726265	-2.541289160006	-1.376905333456
C	2.352752181486	-1.179153845166	2.088116310166
H	2.108443211825	-1.291257455279	3.145024902873
H	3.152887211325	-1.906385972121	1.868591423739
C	3.483336027185	0.411135216523	0.620958498434
H	4.381085082584	-0.224030007908	0.521064971834
H	3.813642840798	1.452320778350	0.585413102889
C	1.423545879516	-3.039518762529	0.019887512586
H	2.342882365790	-3.505184695037	0.379787479406
H	0.649775084529	-3.801415762525	-0.071439313349
C	2.839207139554	-1.551534039707	-1.439143634160
H	3.152250465193	-1.504823275126	-2.480414298681
H	3.657528693776	-1.967043850724	-0.849418035518
C	3.580110477932	0.684600312323	3.007281019552
H	3.821619805599	1.740890409401	2.854064070634
H	4.525452705301	0.127136427337	3.130962955396
C	1.920367691647	-3.686714625663	-2.320362317733
H	1.058305605815	-4.351882875518	-2.265814421756
H	2.828454459456	-4.209927553420	-2.019908677692
P	-2.130402945224	-0.334666154431	-1.062144890195
P	-1.013221732654	1.864359118891	0.802189184290
N	-1.691531132677	2.341895160286	-1.849811852365
N	-2.967933448279	-0.040480728694	1.568971834551
C	-2.701792255143	1.266578450329	-1.932790272437
H	-2.862216733999	1.048121207264	-2.987687569646
H	-3.645195613984	1.614952056382	-1.505592548837
C	-1.643761018925	3.032188190684	-0.542683358294
H	-2.629944442763	3.439648764241	-0.304079476112
H	-0.933244905486	3.854754018616	-0.635332410675
C	-3.461703183454	-0.461761000090	0.256753543810
H	-4.363592173146	0.099239482568	-0.051831227804
H	-3.743464326755	-1.515987602728	0.323157697855
C	-2.639074440782	1.384341677502	1.650785816025
H	-2.517164356482	1.631986985924	2.706517259442
H	-3.451890925813	2.023892176743	1.263137934818
C	-1.726781627764	3.269024805725	-2.996211716973
H	-0.899887191205	3.976566911305	-2.899756912727
H	-2.676685828931	3.810512783985	-3.026572888510
C	-3.921628172871	-0.414210461363	2.615724671729
H	-4.097568685406	-1.493861915310	2.579930840560
H	-4.895005164815	0.098164324487	2.513726991534
H	-3.505334110058	-0.168830887380	3.597657095702
H	-1.598822133100	2.690976812406	-3.915232837368
H	2.025037586585	-3.286094797356	-3.329004377554
H	3.004745857423	0.597557156340	3.933558815607
C	3.048428932018	1.291004605204	-2.185847451628
C	-0.198457633055	3.037137509694	2.048094841263
C	-0.147085836173	-2.697675246372	2.462150033118
C	-2.591557822315	-1.673398250721	-2.323978318009
H	0.847340980631	-2.047218573571	-1.687519730329
H	-0.559636373330	1.688419462991	-1.908193178771
C	0.715344296046	-3.829157189988	3.048248341060
H	1.629777340073	-3.457436363611	3.519887593103
H	0.139142035699	-4.354850563114	3.820219170041
H	0.994271970548	-4.567625282901	2.289598991761
C	-1.411884915878	-3.289550171732	1.816077142189
H	-1.182054079060	-4.018728961404	1.034002545166
H	-1.994549160198	-3.810178359010	2.585818165124
H	-2.040356635483	-2.507784272978	1.388992944328
C	-0.572605890059	-1.716292632726	3.574438366049
H	-1.191960538555	-2.254304121200	4.301985655394
H	0.280890928000	-1.300964696459	4.118968007649
H	-1.165466333278	-0.891785279196	3.170344441319
C	-2.290078966875	-3.043756106173	-1.689218355820
H	-2.895706067636	-3.234219487675	-0.798939410648
H	-2.512399920484	-3.833761260610	-2.416759207470
H	-1.236091748648	-3.135072171516	-1.409579250881
C	-1.686558899192	-1.492426847318	-3.556313435168
H	-1.873982042609	-2.307890929340	-4.265708327561
H	-1.872718133287	-0.550329624641	-4.081074363902
H	-0.626400032752	-1.520750632387	-3.281796021884
C	-4.074481212840	-1.605810808460	-2.729630375850
H	-4.287203935186	-2.395691879037	-3.460827122835
H	-4.736935168142	-1.762672927123	-1.872429299788
H	-4.337060693041	-0.650590016637	-3.194844668802
C	4.567128755513	1.106791509331	-2.350233603868
H	4.924300672946	1.773081596591	-3.145220999105
H	5.113477149079	1.360284537627	-1.436780328147
H	4.831132714075	0.083507569890	-2.636760640379
C	2.727610615510	2.740499784060	-1.773725626607
H	3.056904385314	3.419895484010	-2.568837253585
H	1.649876869462	2.876799162084	-1.630810283193
H	3.239277375150	3.035333047697	-0.852838381898
C	2.326148387258	0.980770919620	-3.510994698807
H	2.539253134284	-0.025729367285	-3.885593469490
H	1.240014134619	1.086432197810	-3.397435790878
H	2.663402593885	1.691643658725	-4.274929659343
C	0.134079964910	2.210547742214	3.306839614856
H	0.707804851858	2.834838428176	4.002583172565
H	-0.763157711434	1.874085978473	3.835514546539
H	0.741404219852	1.335762283349	3.057863757189
C	1.116842806608	3.532181901526	1.419464223539
H	1.764113980707	2.693427627641	1.151610522549
H	0.948411175596	4.135633117590	0.522273165980
H	1.650901716130	4.158960455090	2.144148264490
C	-1.099055857764	4.228952582683	2.412704006341
H	-0.590339246191	4.854258669331	3.157167016328
H	-1.313616915828	4.860837699703	1.544868687149
H	-2.051312353484	3.908514707759	2.847413630313

(^{t}Bu, Me) complex in the ^{3}B1 state

E_{tot} = -2862.05081153246
E_{zpve} = 0.93112411078
E_{SP} = -2862.49046419

Ni	-0.029960713259	-0.184687617209	0.448015800233
P	1.062644424931	1.680850962928	-0.873559683822
P	2.409500194093	-0.621790136935	0.803875065373
N	2.790037664062	-0.329747060929	-1.902479564282
N	2.160597501330	2.040939228359	1.756086889088
C	2.551854887694	1.108405088811	-1.898958569070
H	2.322463860032	1.404333567840	-2.922878613882
H	3.438574320709	1.687881966047	-1.592605398453
C	3.479844268448	-0.812024542479	-0.710438506552
H	4.459409876935	-0.328146706568	-0.551135784178
H	3.658261783502	-1.883516363283	-0.831162659693
C	1.889996121229	2.739591659363	0.445940518650
H	2.846340342369	3.137479299303	0.102591042189
H	1.223605617622	3.570021341975	0.676837620511
C	3.143426382076	0.897379788726	1.634768512562
H	3.459529577124	0.650831997548	2.647150819909
H	4.004546641738	1.279882357233	1.084339695848
C	3.505453879833	-0.747027393625	-3.111796464754
H	3.598922880073	-1.837212896830	-3.118371980985
H	4.517026271148	-0.310893315707	-3.183743683217
C	2.627253331596	3.031720445129	2.786403562829
H	1.883589892763	3.824809344957	2.863813750414
H	3.587569307357	3.434473920671	2.463770751514
P	-2.241870447615	0.388906426250	1.072705359685
P	-1.286832135694	-1.524685568921	-1.037065324963
N	-2.419257307143	-2.432776594866	1.380312101264
N	-2.849542644513	0.807734619238	-1.573765255427
C	-3.154642821182	-1.188755261297	1.580998012966
H	-3.365650585065	-1.099269318343	2.649027019569
H	-4.129172802493	-1.190666813548	1.061612174634
C	-2.205008820964	-2.802006521474	-0.015958494344
H	-3.151324640135	-3.031882125727	-0.538467509791
H	-1.597386404160	-3.711317659887	-0.029898532047
C	-3.381349059323	1.072642280341	-0.235627078464
H	-4.398894931467	0.665004872711	-0.098277235891
H	-3.444752714985	2.154970277822	-0.097739763240
C	-2.722053682076	-0.616995590375	-1.883366352803
H	-2.545304602487	-0.703094918792	-2.956485332324
H	-3.649167073164	-1.175217455093	-1.666700121362
C	-3.060380253841	-3.533920037510	2.099744339543
H	-2.457001810339	-4.441145927775	1.993988609485
H	-4.079139984945	-3.754421986495	1.730425116992
C	-3.669435029812	1.483504097731	-2.582541609273
H	-3.702436359852	2.555444960582	-2.364667626036
H	-4.706238644128	1.104330650000	-2.612665306025
H	-3.224155717477	1.348914320771	-3.572733897116
H	-3.127836082409	-3.288285733422	3.164277747490
H	2.728917431150	2.511210885544	3.738830679041
H	2.936325502302	-0.448271337099	-3.996528961386
C	2.924967347100	-2.036634732372	1.952636235847
C	-0.541977601997	-2.547295559873	-2.449504037898
C	0.325564071465	3.042853356681	-1.987069392523
C	-2.414027523046	1.556464277052	2.547979042600
H	1.267831582827	1.664717306972	2.102809812171
H	-0.162577006237	-1.088401632706	1.745328863310
C	1.362998709912	4.114084092172	-2.367923784020
H	2.227965544309	3.694131533426	-2.889710324588
H	0.890329712781	4.835753359899	-3.045634528688
H	1.720463770993	4.671835002850	-1.496827267433
C	-0.849450592894	3.696552072876	-1.239643331798
H	-0.530011521784	4.232220868558	-0.341291785420
H	-1.327644537831	4.427422730653	-1.902591533258
H	-1.596609496875	2.955494079634	-0.957467962240
C	-0.210840013070	2.347578195541	-3.255730991044
H	-0.755579527882	3.085676999295	-3.855659306408
H	0.588627267934	1.944629922166	-3.884573812019
H	-0.901924954496	1.540095343868	-3.004436099454
C	-1.807737184841	2.910420234316	2.134765588158
H	-2.394304319568	3.409832416235	1.358966306790
H	-1.776527529798	3.573843430173	3.007152839269
H	-0.785849097278	2.793402140237	1.760810937270
C	-1.583580137419	0.975577165978	3.707537496772
H	-1.661699317001	1.643785119624	4.573582551064
H	-1.931353148108	-0.014834883857	4.018244324172
H	-0.525520197301	0.892820510020	3.435344555005
C	-3.880561377308	1.743573023937	2.971721702257
H	-3.929643330510	2.458812906071	3.802173906924
H	-4.491405457148	2.140942963067	2.154649378189
H	-4.333246993745	0.808821633106	3.316436705835
C	4.456313767155	-2.157060230376	2.053645307805
H	4.703952691734	-2.984018336376	2.730344705426
H	4.919427831925	-2.373092918335	1.086482114988
H	4.913430867540	-1.249257505315	2.461449789426
C	2.320819542271	-3.324579421776	1.359717460301
H	2.549532480223	-4.164112191165	2.026623409178
H	1.232714397242	-3.247455109959	1.269172296694
H	2.733794013834	-3.562291899679	0.375082168116
C	2.326212888183	-1.799401609580	3.351983153242
H	2.745476212096	-0.916960889121	3.845727727036
H	1.238993981046	-1.691352677774	3.309213209036
H	2.560967623799	-2.665064599972	3.982339876358
C	-0.023328589414	-1.571966480748	-3.524687563073
H	0.528118186221	-2.141844831042	-4.282128934305
H	-0.833321722727	-1.047560000134	-4.040267491410
H	0.659952406888	-0.833862263742	-3.098441193785
C	0.648128636123	-3.332024241734	-1.868084636971
H	1.394406103919	-2.657712985660	-1.442818037993
H	0.340746580879	-4.039463653261	-1.091830306482
H	1.127377219383	-3.907358627884	-2.669486472003
C	-1.567924813742	-3.514437518039	-3.065347189215
H	-1.099306098825	-4.046832706734	-3.902352896067
H	-1.908084818992	-4.266326510149	-2.346975694486
H	-2.445641005372	-2.991063471677	-3.458353999969

(^{t}Bu, Me) complex in the $^3TS_{B2}$ state

E_{tot} = -2862.04942904659
E_{zpve} = 0.930120650003
E_{SP} = -2862.48921663

Ni	0.054665523999	-0.092930893249	-0.469583303828
P	-0.942741794294	1.814329831322	0.711247570355
P	-2.378330232167	-0.486906390146	-0.867923651901
N	-2.932603360897	0.075721589738	1.759385863476
N	-1.868277709499	2.079045667724	-1.999466732275
C	-2.579469749798	1.482789358513	1.611171760696
H	-2.447273788415	1.895370906850	2.611210493054
H	-3.371893580253	2.075642470573	1.124274516774
C	-3.562926910923	-0.494492141727	0.572843586111
H	-4.503437823835	0.016547876428	0.300220466070
H	-3.807301344021	-1.539832765448	0.779728264037
C	-1.509984227894	2.851915677305	-0.751114677299
H	-2.387554847782	3.448709657849	-0.495759508432
H	-0.693045491200	3.516805657314	-1.032475314697
C	-2.955255353164	1.048388408242	-1.795097723345
H	-3.308766792878	0.772551351005	-2.787139123457
H	-3.766901314808	1.548149610103	-1.264651785098
C	-3.766160605893	-0.147727761507	2.943261868981
H	-3.941751293056	-1.221166360895	3.061991162862
H	-4.744421566980	0.360003281472	2.881271754531
C	-2.247046649348	3.034204107821	-3.098276225988
H	-1.434018415217	3.748365965231	-3.229985986264
H	-3.165055872561	3.545239291205	-2.807312652505
P	2.397120489469	0.153144277621	-0.929199532047
P	0.985540504618	-1.579098355562	1.148705606839
N	2.108500336383	-2.641724599012	-1.190207889343
N	2.801068165324	0.542846366976	1.756984722815
C	3.100065375918	-1.568569725454	-1.268314789818
H	3.509423723114	-1.555570859554	-2.280038660645
H	3.949580814539	-1.735780340593	-0.584349202087
C	1.740016718487	-2.994010958460	0.180997170453
H	2.597785379610	-3.390784755250	0.753560267913
H	0.982876131549	-3.781857704876	0.136451283857
C	3.474454895503	0.742419947042	0.472604665612
H	4.458012212757	0.240845838742	0.430830980850
H	3.650737379165	1.812750571953	0.336633902307
C	2.497451288515	-0.859161114612	2.040968622837
H	2.281267261975	-0.937537572369	3.106810038726
H	3.356037512287	-1.523004176629	1.843021797085
C	2.563963229094	-3.833244072699	-1.910218557610
H	1.781281248843	-4.597758079657	-1.880611368246
H	3.485981867157	-4.267740155634	-1.482925609918
C	3.592027984431	1.138169128332	2.837054130386
H	3.745670739128	2.201396841719	2.628413394868
H	4.581186802653	0.661689943751	2.955555626152
H	3.051291060965	1.050555887491	3.784056169920
H	2.756101750701	-3.578770937285	-2.957065201624
H	-2.399300736476	2.459214761755	-4.011643982424
H	-3.244178317005	0.213353651412	3.834016047374
C	-2.969382556370	-1.902777396385	-1.986539547480
C	0.065574302968	-2.453210971682	2.562244889586
C	-0.144018500169	3.146382312597	1.809963451710
C	2.907148245041	1.207047362757	-2.416993381357
H	-1.027917607262	1.585441225287	-2.331987224847
H	0.068293574791	-0.605643889495	-2.012706915993
C	-1.079546966918	4.344040966339	2.050737921766
H	-2.025747391325	4.053718895684	2.516262255906
H	-0.581919582907	5.045726101120	2.731187468508
H	-1.299977742300	4.885641453785	1.125691028712
C	1.152725842062	3.629505044488	1.139080855510
H	0.964500173288	4.158916821823	0.200824931771
H	1.658530207491	4.330557335202	1.813436423803
H	1.832050633336	2.799774953631	0.945601331215
C	0.205715215704	2.455163852484	3.142999089133
H	0.762718212772	3.160747424543	3.770354123815
H	-0.682217768902	2.149890962274	3.704461957939
H	0.836093859040	1.579088017929	2.974410653532

C	2.273204923902	2.597502995182	-2.219916019614
H	2.655692473063	3.102770394230	-1.329043762980
H	2.504947268228	3.228538215132	-3.086297411567
H	1.181697371913	2.529237003180	-2.134647272109
C	2.343351954548	0.582283782716	-3.707085787863
H	2.612766806642	1.224024648796	-4.554616115655
H	2.754533799802	-0.412200320499	-3.906040536165
H	1.253747830136	0.498961379678	-3.673132546133
C	4.438241889459	1.323235611390	-2.522811986340
H	4.694504452054	1.923093978538	-3.404882404527
H	4.875946430082	1.816729590270	-1.649953879877
H	4.912729426005	0.343387412922	-2.639191684809
C	-4.502508092229	-1.893907185686	-2.135860392168
H	-4.799267534243	-2.726527118180	-2.785237165651
H	-5.013131596262	-2.025998343597	-1.177876846110
H	-4.867596344439	-0.970798849433	-2.598072623885
C	-2.505860700680	-3.216272581340	-1.328016074114
H	-2.777958614082	-4.055528842762	-1.978847301730
H	-1.420150544897	-3.233130869758	-1.187920116362
H	-2.982009625906	-3.382333293663	-0.357820801231
C	-2.310994595943	-1.782725759855	-3.373782935250
H	-2.582335255366	-0.857963729403	-3.892857768479
H	-1.221657326065	-1.831228308789	-3.307901852018
H	-2.657143198048	-2.618075944927	-3.993877649286
C	-0.373320537797	-1.379820473418	3.577907607368
H	-0.990859614652	-1.855122100271	4.349550573479
H	0.474422068060	-0.909408805580	4.084472732661
H	-0.974748061625	-0.603861679648	3.098398772086
C	-1.184712620706	-3.123549653738	1.970171932877
H	-1.818868488622	-2.392720142840	1.468100507063
H	-0.934736533141	-3.913462987631	1.256296574685
H	-1.767924587108	-3.579801348389	2.779176558838
C	0.949553709609	-3.508222442780	3.251067315563
H	0.386895626104	-3.956957596867	4.079260815427
H	1.230686364453	-4.316066189644	2.569097775928
H	1.863818545210	-3.077642416689	3.671361693168

(*t*Bu, Me) complex in the ^{3}B2 state

E_{tot} = -2862.06384592963
E_{zpve} = 0.933223026751
E_{SP} = -2862.50351951

Ni	0.005920483225	-0.209329071574	-0.376431991105
P	1.462573446968	-1.194740074022	1.270891417021
P	2.168835260042	0.647929628889	-1.110635901895
N	3.771364204484	0.396680634748	1.089733364256
N	2.594549853103	-2.143488740559	-1.150509177825
C	2.698305336118	0.017176714286	2.006350252112
H	2.135743973106	0.882574371104	2.385296075065
H	3.147940700457	-0.485865391915	2.864846729748
C	3.382236973899	1.392247652525	0.091533497595
H	4.274094618670	1.681485809430	-0.471657561145
H	2.952659131380	2.302804403065	0.533074119003
C	2.601262435173	-2.358682858413	0.335897598633
H	3.628458187615	-2.276436127847	0.694172747846
H	2.234325753942	-3.373473435623	0.495907323381
C	3.186798608203	-0.847480614078	-1.621463865536
H	3.212968487104	-0.901051199769	-2.709449087691
H	4.203463447251	-0.771766154975	-1.234969354765
P	-2.350698901210	-1.004407107835	-0.722574207606
P	-1.266862545629	1.573097673122	0.861790031932
N	-2.100356127134	1.524497153227	-1.798434089980
N	-3.776402286609	0.316189532127	1.226129053501
C	-3.003562233841	0.362719053684	-1.814905620363
H	-3.032036921599	-0.021621948017	-2.835503064099
H	-4.033137263897	0.620985696531	-1.523889044758
C	-2.199471385830	2.331112470489	-0.572694346878
H	-3.239462453564	2.546482282211	-0.286908987050
H	-1.697779176917	3.281817179442	-0.765716999198
C	-3.602718889117	-1.017419781651	0.660326196268
H	-4.561753159762	-1.331404646582	0.243348252231
H	-3.297354210091	-1.768058409182	1.410268995814
C	-2.606233771010	0.802745300678	1.949239473726
H	-2.135520466574	0.014557271456	2.556538661860
H	-2.938015899059	1.579712918059	2.638273066937
H	1.555658511219	-2.053705413502	-1.428638915766
H	0.343969047102	-1.464279712951	-1.629697218094
C	-2.649525610155	-2.631464037702	-1.655693055887
C	-0.749215739235	3.081542077734	1.903031265317
C	2.114950869616	1.759762107224	-2.627834353977
C	0.888661840336	-2.218172316279	2.752663248550
C	3.201777671899	-3.303766126103	-1.867230556095
H	4.261490900012	-3.365632609585	-1.613399891895
H	2.684693233665	-4.212903469435	-1.558762849440
H	3.077188794958	-3.151442014977	-2.940279917282
C	4.968442396740	0.826362431995	1.820499497123
H	4.798220536611	1.735612071058	2.422282986190
H	5.295969785140	0.024995100150	2.489306807661
H	5.774552214956	1.029621317640	1.109419029543
C	-2.317358640488	2.362694573960	-2.983559834307
H	-2.172925514165	1.762217606119	-3.886186027824
H	-3.329473841631	2.801226785459	-3.014928455046
H	-1.586657590320	3.176191547494	-2.994854096507
C	-4.981679597108	0.376409344264	2.058579076276
H	-5.849937970466	0.072096576772	1.466233849956
H	-4.923315756071	-0.276354901062	2.947543094652
H	-5.142948187352	1.404720288927	2.395747387359
C	-4.150141225048	-2.947225769364	-1.796991663623
H	-4.267240655430	-3.827775846853	-2.440253944623
H	-4.618279155990	-3.181335396617	-0.836714816941
H	-4.700491828194	-2.121258975557	-2.260505519343
C	-1.948469432530	-3.739065931371	-0.846642022333
H	-2.103325092013	-4.704734374984	-1.343007633443
H	-0.871228236129	-3.558827146575	-0.779628092543
H	-2.348568296721	-3.817500716499	0.169065771847
C	-2.022714683212	-2.548605476365	-3.061321312602
H	-0.977447344254	-2.231384120408	-3.023977475857
H	-2.062125679162	-3.544374196324	-3.519166048441
H	-2.571859908126	-1.867068887521	-3.718095721071
C	-0.272249507953	-3.099064051670	2.261994832172
H	0.042998740790	-3.784413632993	1.468949520817
H	-1.099180591970	-2.493918638245	1.879489052730
C	-1.911572987616	4.074753497912	2.091392992880
H	-2.779415034242	3.625752592335	2.583168791820
H	-1.568847143487	4.901031520094	2.726592399644
H	-2.240281504431	4.506487499905	1.141546083409
C	0.412640028057	3.795082174931	1.194156261445
H	0.753858111208	4.632638891871	1.814539052817
H	1.259745927096	3.123742116363	1.041910404088
H	0.117614845067	4.201880388278	0.222921048262
C	-0.270826715188	2.566752419076	3.274394632549
H	0.536950894243	1.836586934833	3.174542548921
H	0.112006323577	3.410961967511	3.860181694848
H	-1.076830735414	2.105603346928	3.852900958043
C	1.480954044263	3.087219929046	-2.168462192115
H	2.110871216302	3.614314969397	-1.445462287637
H	1.351021279185	3.740366645088	-3.039270142227
H	0.498462070685	2.923452406017	-1.717348073664
C	1.183774292438	1.078351165427	-3.650014956324
H	1.053781134832	1.743955551204	-4.511308373161
H	1.596421624730	0.133111118515	-4.017996665466
H	0.198482130164	0.879913937524	-3.216727021600
C	3.500630547474	2.015506333164	-3.241498025605
H	3.383230958351	2.658598851693	-4.122043846423
H	4.170206052016	2.529648957332	-2.545211432122
H	3.986726158991	1.092014978714	-3.571417645747
C	2.006725250856	-3.094081911541	3.345020264224
H	1.622037732630	-3.594491249697	4.241803453091
H	2.333792939630	-3.874277214248	2.651539510649
H	2.882442184542	-2.509997596698	3.644891889997
H	-0.648438583786	-3.702921743624	3.096340295335
C	0.383949557561	-1.226000446053	3.818877961657
H	-0.349872858614	-0.524954975499	3.412564955915
H	-0.099530787542	-1.788345503976	4.626029102437
H	1.201531256391	-0.648124025373	4.260156550673

(*t*Bu, Me) complex in the ^3TS_C state

E_{tot} = -2862.06084967449
E_{zpve} = 0.927526883214
E_{SP} = -2862.50075335

Ni	0.000034475007	-0.189702979509	0.311195947317
P	-1.491389953837	-1.114460279269	-1.313507054372
P	-2.110001743913	0.609747171645	1.174721078476
N	-3.773704379435	0.487071064772	-0.994751385847
N	-2.528265336365	-2.160544339441	1.091958671691
C	-2.732911281886	0.139497190618	-1.959362767197
H	-2.174894717576	1.016184814161	-2.318708638627
H	-3.212774275767	-0.323062288190	-2.824548010480
C	-3.341620767159	1.423334454566	0.041594752291
H	-4.212997823699	1.691363788705	0.646199836809
H	-2.915063292936	2.352174044133	-0.363191698698
C	-2.604231406361	-2.310639730070	-0.379489262807
H	-3.636429306779	-2.202070111918	-0.725760890326
H	-2.251292601339	-3.316567791828	-0.612361075729
C	-3.110190397372	-0.916280542861	1.643034560674
H	-3.096712943115	-1.011020138066	2.729416420658
H	-4.143290927376	-0.792407042077	1.307364758423
P	2.279230693628	-1.041114473233	0.680024625605
P	1.270404417676	1.589378813284	-0.825706521008
N	2.146240321757	1.468706750140	1.818661275775
N	3.727052146544	0.267307610756	-1.262735155980
C	3.001916979947	0.273766653444	1.790870625322
H	3.029471911705	-0.139442308573	2.800437918443
H	4.036845189322	0.495163670953	1.488640422723
C	2.243272405053	2.295854165432	0.608027789343
H	3.282334573276	2.491593854447	0.304467254364
H	1.768867524957	3.254327128386	0.829573963166
C	3.492969600802	-1.072406324500	-0.734390084808
H	4.441472397787	-1.449182158125	-0.346854007503
H	3.134751600614	-1.783167757115	-1.498477250383
C	2.576422397713	0.833079887111	-1.958274820380

H	2.077104005707	0.096838179816	-2.606501220908
H	2.935492482208	1.635307468556	-2.603608711740
H	-1.239116930321	-1.958242411413	1.343072960252
H	-0.333867434618	-1.620045176688	1.485225592245
C	2.536564715045	-2.696822081814	1.570969200202
C	0.741402323653	3.126329934175	-1.814011188640
C	-1.983558481516	1.650191255090	2.735275303422
C	-0.959506934836	-2.076777254064	-2.849269957265
C	-3.008352810104	-3.363517321260	1.801971432976
H	-4.081220729129	-3.512591803272	1.637578884471
H	-2.458051057776	-4.233178016767	1.436022438093
H	-2.815662912767	-3.241225911373	2.870196958174
C	-4.982051912975	0.973614658463	-1.668632441292
H	-4.812196744685	1.910202062108	-2.227316429799
H	-5.341461349138	0.213657615702	-2.368668609733
H	-5.765173389469	1.152569626872	-0.925963110665
C	2.421996375709	2.273582051264	3.014286586639
H	2.275664591376	1.660719384893	3.908230660965
H	3.450135360380	2.673748444716	3.030089522014
H	1.722375823921	3.112801203915	3.058053663868
C	4.923521220647	0.288979481402	-2.110547602938
H	5.782042964811	-0.076622951314	-1.539234029742
H	4.818446548960	-0.334585871160	-3.015791812265
H	5.133324542288	1.316598091921	-2.421596461664
C	4.032214280592	-3.028808606917	1.731257766632
H	4.128473052151	-3.930342288406	2.348101401195
H	4.518192638099	-3.236461149417	0.774090336210
H	4.579160638962	-2.223220387227	2.232753898805
C	1.842917078369	-3.775731232260	0.717838184413
H	1.982134265902	-4.754990102899	1.191319453570
H	0.768378863470	-3.589122176446	0.632584993615
H	2.264693607116	-3.829307866715	-0.290640416724
C	1.889724719643	-2.643404294987	2.969232516317
H	0.845753684819	-2.323091532684	2.931337070031
H	1.919468455800	-3.649780376575	3.403549455883
H	2.433472059012	-1.980243516146	3.648758196447
C	0.198418098229	-2.992031575058	-2.418194073666
H	-0.113607887724	-3.711904207676	-1.654979817089
H	1.034921006529	-2.412994164576	-2.017160810920
C	1.907086222945	4.117106614412	-1.996019451289
H	2.762408428896	3.677405248744	-2.517012943634
H	1.556222130178	4.962008162817	-2.601301925819
H	2.256289335623	4.520973434023	-1.041367867826
C	-0.401006846893	3.825539588358	-1.061395716462
H	-0.748878025076	4.682667461806	-1.650306329054
H	-1.248488235881	3.154429229375	-0.912112857359
H	-0.082978155991	4.201536041966	-0.084954354600
C	0.236168259218	2.652449818712	-3.189996365752
H	-0.572600466393	1.922950728225	-3.096398797698
H	-0.153233363606	3.514732012433	-3.744093725195
H	1.030519098864	2.205062116979	-3.794764625096
C	-1.354638131214	2.993646414769	2.319143075520
H	-2.007911281385	3.560632344565	1.648902614829
H	-1.184167043693	3.601377409218	3.215460679733
H	-0.392885285373	2.845078737371	1.821403641336
C	-1.022360175601	0.905007104042	3.682947927061
H	-0.838154243450	1.527409520165	4.566270833503
H	-1.439488899185	-0.047251163873	4.026990665511
H	-0.061621070397	0.708147980638	3.196373153507
C	-3.341791764862	1.885948941292	3.414853239880
H	-3.182730779540	2.476463951772	4.325209986428
H	-4.029365031807	2.447207649768	2.774728379615
H	-3.827745876555	0.950690518188	3.709758466851
C	-2.102802911256	-2.913811430037	-3.450611391703
H	-1.742456557332	-3.387762862808	-4.371589810425
H	-2.430395063776	-3.712785834803	-2.779249487286
H	-2.973694500575	-2.305181855414	-3.713464118229
H	0.558646532691	-3.557945391218	-3.285457513364
C	-0.465688917124	-1.051268018107	-3.887645480623
H	0.296126916313	-0.384603844200	-3.475893913942
H	-0.021440007944	-1.587968997973	-4.733939970645
H	-1.283806360971	-0.437008078227	-4.275784479603

(*t*Bu, Me) complex in the ^{3}C state

E_{tot} = -2862.08276688087
E_{zpve} = 0.921297609083
E_{SP} = -2862.52441824

Ni	-0.033226221227	0.004875315651	-0.113058347354
P	1.412849078275	-0.971068224560	1.460192824339
P	1.982360866734	0.603588346974	-1.177402762402
N	3.719330432156	0.537576785923	0.937807955111
N	2.107134413193	-2.151581863607	-0.966201058357
C	2.701427880252	0.302511097938	1.960413492881
H	2.166297449578	1.220156945497	2.246423994244
H	3.197006656594	-0.081324768667	2.854286079545
C	3.292183939137	1.421709832134	-0.145006979909
H	4.151726612498	1.606302021076	-0.795336958523
H	2.920445098212	2.396662212151	0.207070511014
C	2.359316053061	-2.247711422809	0.476605594761
H	3.428234314496	-2.193461600512	0.733841893034
H	1.980211690268	-3.215682968079	0.809966289495
C	2.779957678398	-1.024160324710	-1.621583512419
H	2.665051867779	-1.146139817885	-2.700434304979
H	3.857495048074	-0.975447589740	-1.400039869739
P	-2.084840553628	-1.027904715612	-0.606614482822
P	-1.402585778706	1.626743386098	0.893188663725
N	-2.172154552726	1.466939969026	-1.778541808257
N	-3.747301537792	0.125591030773	1.236609886097
C	-2.899039748072	0.190253591190	-1.761129646971
H	-2.847089479540	-0.237562349544	-2.764430884587
H	-3.960850308204	0.301474992933	-1.494276583725
C	-2.354882757411	2.287356178458	-0.574118167543
H	-3.411844749931	2.427181054274	-0.300773635424
H	-1.925348212725	3.269595875780	-0.784487261860
C	-3.341274737651	-1.192161397534	0.754046061741
H	-4.216330637498	-1.687778149983	0.325338332852
H	-2.940707854371	-1.841536199197	1.548214085558
C	-2.708688981615	0.831011978604	1.983044252101
H	-2.197267011883	0.183616212877	2.711850940679
H	-3.181285114408	1.642261859206	2.540714646949
H	1.104080186913	-2.608853739272	-4.716446279888
H	1.384688335126	-2.540781762386	-5.401981613013
C	-1.973922178693	-2.672252670270	-1.509479078551
C	-0.858305347403	3.156442359600	1.862485757394
C	1.794252078100	1.581146700451	-2.770765622072
C	0.946803925182	-1.837656187028	3.075113271878
C	2.444276046922	-3.414194740850	-1.631815538158
H	3.514205211677	-3.669167875974	-1.543902902179
H	1.856626771567	-4.225679506196	-1.192738637150
H	2.192173997265	-3.344250879793	-2.693497078939
C	4.972405294177	1.013650800600	1.533315195238
H	4.864408641735	1.993450703759	2.030272110889
H	5.325108811649	0.287940868917	2.271864786509
H	5.733854250494	1.105289307962	0.753041035373
C	-2.517821173375	2.238401385444	-2.977946712768
H	-2.305348720803	1.641429623157	-3.869164126458
H	-3.580469600852	2.533595308908	-2.999922633063
H	-1.904560782469	3.142627432215	-3.018409412551
C	-4.988661546312	0.036925790053	2.012914972435
H	-5.766167656219	-0.431983708633	1.402745574503
H	-4.871631079872	-0.551420425382	2.939505320048
H	-5.323832928272	1.042891093346	2.281600094517
C	-3.346371193806	-3.150943344796	-2.009742951574
H	-3.211379651601	-4.080013417400	-2.576515873267
H	-4.033670815923	-3.364069738510	-1.185472775516
H	-3.817619995869	-2.421369525368	-2.676144099267
C	-1.366621002632	-3.692228899225	-0.528488135612
H	-1.233312081371	-4.649829285912	-1.045236831416
H	-0.388485958871	-3.362877535594	-0.167899528357
H	-2.014856463497	-3.864806522617	0.336099350496
C	-1.010679636590	-2.465480880395	-2.696135691512
H	-0.046649989987	-2.072770260217	-2.359542664010
H	-0.834870644598	-3.433428615464	-3.179296047903
H	-1.422590322242	-1.789527945295	-3.451202784804
C	-0.194323931888	-2.822243271428	2.771897922017
H	0.120898788863	-3.623341252582	2.097489572396
H	-1.051672700610	-2.313252224309	2.324519277399
C	-1.997457156009	4.187468299058	1.969516912487
H	-2.878953621764	3.784575490536	2.477777854444
H	-1.640099696652	5.041088922801	2.557906888231
H	-2.306263452453	4.568924251770	0.992301521090
C	0.342592435540	3.769211715528	1.123681986145
H	0.693953998806	4.649382720292	1.674756340951
H	1.169276994000	3.056791745514	1.059984238035
H	0.083971929685	4.089752503090	0.110622611704
C	-0.422883831165	2.716098820158	3.271467055734
H	0.381392786469	1.978349332163	3.236535899744
H	-0.049051837788	3.592433999973	3.813894303545
H	-1.250573586601	2.297120605362	3.851182954752
C	1.193443942703	2.948212254781	-2.393812800522
H	1.870893306883	3.530435818582	-1.761172119023
H	1.011121090793	3.524032570138	-3.308662997586
H	0.240791518830	2.831367480927	-1.870240609171
C	0.800787945182	0.801740892574	-3.654893719170
H	0.573343517603	1.400166369801	-4.544679038214
H	1.213693003512	-0.152242808457	-3.994771154499
H	-0.136690675625	0.609485428639	-3.125280912402
C	3.132367207503	1.763674854738	-3.504971184965
H	2.947329206773	2.287921838244	-4.450242270070
H	3.839656132357	2.366125251018	-2.927236602039
H	3.605382637602	0.805814929142	-3.744853103290
C	2.146372585060	-2.593910132679	3.675490083880
H	1.829369099405	-3.057937225907	4.617280036548
H	2.504717391673	-3.393420492624	3.020556296628
H	2.986216931513	-1.931024772066	3.903855028809
H	-0.525122501496	-3.285989865497	3.708741585437
C	0.453906183901	-0.767836842125	4.065097690545
H	-0.396909862970	-0.210949412345	3.665278143007
H	0.127866000870	-1.260955855687	4.988418606306
H	1.240282458347	-0.054922199677	4.330216833175

(*t*Bu, Me) complex in the ^{3}P state

E_{tot} = -2860.90281013295
E_{zpve} = 0.90964123083
E_{SP} = -2861.34374517

Ni	-0.032232495889	0.004017417462	-0.113143615022
P	1.413664591275	-0.971129434612	1.461315174677
P	1.983383012217	0.604680785039	-1.176085695652
N	3.719696539786	0.538977126720	0.938847959860
N	2.105983396357	-2.149251136675	-0.965003799285
C	2.702266152596	0.302410690435	1.961655814172
H	2.167015619757	1.219639875174	2.248813034620
H	3.198442941908	-0.082051604070	2.854935027563
C	3.291930265811	1.424100987907	-0.143035689824
H	4.151449039829	1.610448982906	-0.792910017399
H	2.918838926308	2.398118213292	0.210125504729
C	2.359023336169	-2.248602336128	0.477299908444
H	3.428023492329	-2.196186707404	0.734462275113
H	1.978419380466	-3.216297358292	0.809726490644
C	2.782442168981	-1.022879072382	-1.618074851299
H	2.671659149115	-1.144538634262	-2.697455142546
H	3.859117876647	-0.975077969605	-1.392610005638
P	-2.084383801943	-1.028144392828	-0.606751510875
P	-1.401722113768	1.626373495580	0.892888181061
N	-2.172194610424	1.466709471152	-1.778696808823
N	-3.746488000556	0.124881694744	1.236680114515
C	-2.899171189879	0.190118616578	-1.760800916733
H	-2.848058880046	-0.237590558161	-2.764167410348
H	-3.960770746877	0.301455042295	-1.493172709975
C	-2.354370937242	2.287060219056	-0.574193217419
H	-3.411229661884	2.426944824441	-0.300450090395
H	-1.924848497905	3.269277130057	-0.784665050570
C	-3.340582649046	-1.192897831878	0.753991903109
H	-4.215706927007	-1.688486859041	0.325373852379
H	-2.939854530827	-1.842307550534	1.548044565472
C	-2.707731722549	0.830430191319	1.982852504449
H	-2.196154237486	0.183121867016	2.711625770380
H	-3.180338432243	1.641644751849	2.540567296540
C	-1.972460473813	-2.672063533625	-1.510704152048
C	-0.857986608866	3.156365756205	1.862100787225
C	1.794644850582	1.579370020706	-2.771194662201
C	0.947234133500	-1.837516115768	3.076171398458
C	2.433868068225	-3.411551206008	-1.635534623407
H	3.503670851810	-3.670224928966	-1.557680168424
H	1.847504948334	-4.222087179397	-1.193072801278
H	2.171826303203	-3.338665562194	-2.695024977022
C	4.972779405794	1.014892151027	1.534490462377
H	4.864566586849	1.994091254836	2.032581836812
H	5.325992705564	0.288493048586	2.272120865320
H	5.733958202513	1.107665324543	0.754087583687
C	-2.518385030499	2.238109986199	-2.977978578833
H	-2.306071628245	1.641160491602	-3.869258947312
H	-3.581092522522	2.533130159842	-2.999659275971
H	-1.905296634486	3.142442597081	-3.018651104162
C	-4.987682181499	0.036230012639	2.013253269342
H	-5.765287496595	-0.432780069655	1.403288545054
H	-4.870445945797	-0.552009151121	2.939884388299
H	-5.322855395663	1.042205987205	2.281900899826
C	-3.344527754858	-3.151383307723	-2.011312135852
H	-3.208958237988	-4.080146075727	-2.578475087655
H	-4.031761383985	-3.365285540606	-1.187182075434
H	-3.816218835359	-2.421805619615	-2.677399185299
C	-1.364707753064	-3.692118886761	-0.530085274813
H	-1.230525965983	-4.649385810335	-1.047219354821
H	-0.387003965145	-3.362185356931	-0.168885395207
H	-2.013194162993	-3.865605350571	0.334134015716
C	-1.009243869012	-2.462505447352	-2.696579811359
H	-0.046140423121	-2.068322499508	-2.358919391706
H	-0.830874312805	-3.429158595335	-3.181986858385
H	-1.422563370219	-1.785682826422	-3.450285392896
C	-0.194053568242	-2.821882162269	2.772717252943
H	0.120992014339	-3.622858121590	2.098075880061
H	-1.051367063249	-2.312660994791	2.325521055345
C	-1.997407764373	4.187131913459	1.968866979911
H	-2.878980234100	3.783994556815	2.476803805982
H	-1.640438428232	5.040778800296	2.557455518564
H	-2.305993947023	4.568632191101	0.991601525657
C	0.342820190879	3.769370656059	1.123313662310
H	0.693420822484	4.650166301560	1.673875386340
H	1.170005000256	3.057466529376	1.060437209036
H	0.084422436712	4.089012992663	0.109894878333
C	-0.422676332676	2.716407810695	3.271244597828
H	0.381483182592	1.978515358888	3.236670688861
H	-0.048790104091	3.592856515652	3.813451689414
H	-1.250475865587	2.297706177201	3.851006599349
C	1.192193033207	2.946460998794	-2.396632814304
H	1.869351746808	3.530373877823	-1.765237100796
H	1.008958585216	3.520555441000	-3.312385685733
H	0.239883176208	2.829554887604	-1.872461401292
C	0.801433022546	0.797855188209	-3.654164097436
H	0.575760849093	1.393543052440	-4.546179658518
H	1.214027694246	-0.158318686556	-3.990342849537
H	-0.136689683657	0.608393074756	-3.124771250870
C	3.132701894667	1.762426789088	-3.505315702986
H	2.947502463237	2.285774892621	-4.451052393382
H	3.839235481774	2.365957569933	-2.927780962280
H	3.606701304423	0.804815376404	-3.744242311061
C	2.146626501442	-2.594041373328	3.676564684505
H	1.829490497846	-3.058186763987	4.618253722502
H	2.504937745404	-3.393475451662	3.021510514355
H	2.986535981081	-1.931292822867	3.905106490531
H	-0.524877968163	-3.285826367489	3.709454097806
C	0.454412405207	-0.767678101414	4.066173841190
H	-0.396255583924	-0.210611189643	3.666280927597
H	0.128169674136	-1.260780076532	4.989434946931
H	1.240883641674	-0.054906084312	4.331407348891

(CF_3, H) complex in the ^1R state

E_{tot} = -3422.88173654956
E_{zpve} = 0.36033961042
E_{SP} = -3423.65143893

Ni	0.000786554826	-0.032782987292	-0.197949580560
P	1.672410769592	0.863098319729	-1.226928560419
P	1.487629334481	-0.874250673834	1.118556308196
N	2.719826379307	-1.691757882431	-1.200509109637
N	3.313028572854	1.144114722967	0.947431989451
C	3.012072407658	-0.359415256099	-1.742862682249
H	2.990254834985	-0.435000797775	-2.834175497204
H	3.990153399799	0.042036743768	-1.439154120636
C	2.847195053160	-1.845920494419	0.253076610353
H	3.820297387989	-1.525548497214	0.654277326877
H	2.705922100771	-2.904935435528	0.489000732236
C	2.624447367408	1.976768642003	-0.040502781773
H	3.379202823990	2.549751388122	-0.587106798309
H	1.894044082798	2.673620018226	0.398635104160
C	2.482380001442	0.485235870952	1.956800639178
H	1.763166813530	1.158147418585	2.447656107588
H	3.142817460310	0.055052058674	2.715651404864
P	-1.485296450043	1.178915037719	0.789586658655
P	-1.669974951726	-1.197544092361	-0.908046255469
N	-3.306768703085	-0.797534170947	1.252695392568
N	-2.717548343796	1.237614259823	-1.670105781802
C	-2.475267052031	0.141175818341	2.007236733601
H	-1.752101753060	-0.345873538546	2.679340651567
H	-3.135416462802	0.782523676610	2.598794754927
C	-2.619265917635	-1.892537177390	0.565741628689
H	-3.374984663708	-2.604695016387	0.221671049436
H	-1.887870970622	-2.421843925912	1.195374644565
C	-2.845731823751	1.834271203043	-0.334982432251
H	-3.818751927213	1.651564092038	0.145108894108
H	-2.705893304424	2.914772078817	-0.437734007628
C	-3.014752944759	-0.196288331176	-1.769903884809
H	-3.004173985524	-0.464744943022	-2.830426701882
H	-3.989724809907	-0.480094700230	-1.346812049590
H	-4.015796851559	-1.183405526780	1.868485006155
H	3.339791119524	-2.362295873755	-1.648702830047
H	4.025632875405	1.698324809534	1.412030095140
H	-3.337534597331	1.737095457771	-2.303446877478
C	-1.686035099326	-2.740350936126	-1.995190799931
C	-1.193169206372	2.694689607258	1.871208360992
C	1.682273482078	1.986169488742	-2.743109663904
C	1.188184639202	-1.982992127623	2.613756278355
F	-1.013340625067	-3.748506831214	-1.383948928558
F	-1.072897831450	-2.509898164779	-3.182011652048
F	-2.926421763815	-3.213942457533	-2.282387727854
F	-0.497867384498	3.646021540196	1.198611716465
F	-0.456612401419	2.356799232726	2.961949286649
F	-2.322703194850	3.289657979593	2.331346130561
F	2.921194567343	2.322991983607	-3.186886072744
F	1.032838283130	3.146965269479	-2.472911630941
F	1.041473813984	1.400505472338	-3.784585431253
F	2.314336176596	-2.449175705327	3.210146499552
F	0.491513277094	-1.307108209398	3.564812305518
F	0.449029596972	-3.069333767535	2.274158178562

(CF_3, H) complex in the ^1A state

E_{tot} = -3423.83002341904
E_{zpve} = 0.391699237822
E_{SP} = -3424.58357612

Ni	0.004063878381	0.002961258345	-0.039054671787
P	1.576658488934	0.944131459821	-1.148007450939
P	1.516596688598	-0.832122707331	1.223559694480
N	2.507680751887	-1.679507227232	-1.237415118584
N	3.412786077112	1.050709804531	0.845653364509
C	2.802009621242	-0.315038384225	-1.817415236033
H	2.695153707295	-0.414613713781	-2.899404623374
H	3.829714573253	-0.059828243283	-1.559092275614
C	2.736222322076	-1.873533447276	0.244711841870
H	3.769299731700	-1.599278399649	0.458284924719
H	2.577518946958	-2.935732301623	0.440660839677
C	2.699309161907	1.967621033671	-0.041345336890

H	3.432609638087	2.505640034942	-0.647614780962
H	2.058781003575	2.693003188278	0.482230814601
C	2.661702204219	0.460791029359	1.952123541275
H	2.032987083864	1.177186449602	2.502296478610
H	3.367327606359	-0.005579669752	2.644578754261
P	-1.513320104995	1.179441803625	0.905951929228
P	-1.561871750102	-1.244438425436	-0.795013621188
N	-3.400530237233	-0.741240753556	1.137887746864
N	-2.515873229132	1.216419470015	-1.691854098762
C	-2.647868606652	0.164735377319	2.002693079095
H	-2.015401854495	-0.341688624604	2.747480901792
H	-3.353840996062	0.821281122862	2.518116342039
C	-2.682086489752	-1.881032541562	0.571864230267
H	-3.412155511883	-2.580944985916	0.157071269831
H	-2.040935166397	-2.411214510840	1.291636935643
C	-2.750236829446	1.850966261833	-0.340369400613
H	-3.777533555827	1.635286338077	-0.047265158218
H	-2.611892171938	2.924875825060	-0.480308201943
C	-2.788998314065	-0.263603729866	-1.827743333454
H	-2.664643591880	-0.500924948447	-2.886393327080
H	-3.818915916231	-0.439622643205	-1.518170125094
H	-4.234478854890	-1.063214088166	1.617928383266
H	3.085293458327	-2.366377681995	-1.736924073433
H	4.254305653970	1.494918113342	1.197815548084
H	-3.101880842756	1.708460965450	-2.376988704661
H	1.525948252459	-1.914196797437	-1.448897093591
H	-1.537253325467	1.391447351375	-1.965661277405
C	-1.413340598883	-2.776087795626	-1.883197829707
C	-1.238783325237	2.721863966265	1.952161129646
C	1.421972813722	2.056908749062	-2.660555054700
C	1.209560958627	-1.979583338528	2.685010225918
F	-0.739339149993	-3.737528656653	-1.208128552867
F	-0.706725370229	-2.494391160592	-3.003723496847
F	-2.592514758475	-3.307776905668	-2.275725743986
F	-0.508797158522	3.633821526492	1.268223529296
F	-0.538918330453	2.386926413486	3.062035630770
F	-2.372783593638	3.336524362113	2.356217947383
F	2.597917366248	2.439587540216	-3.205485589855
F	0.746407918701	3.178976075017	-2.315449500588
F	0.712146911791	1.438556425113	-3.634972468430
F	2.325040814330	-2.504199576757	3.238078749255
F	0.555798405145	-1.296686495492	3.654104162063
F	0.420800685862	-3.015698960786	2.311998422162

(CF_3, H) complex in the $^1TS_{B1}$ state

E_{tot} = -3423.79917235532
E_{zpve} = 0.384464932674
E_{SP} = -3424.54878452

Ni	-0.099350738973	0.077009213132	-0.141310983278
P	-2.205769658263	0.368890620179	-0.078600485029
P	-0.222451323042	-0.336380074407	1.989438113876
N	-1.513337493743	2.130599848434	1.956623135522
N	-2.771719528471	-1.201414376143	2.018972155047
C	-2.700839083391	1.722814393361	1.117048917453
H	-3.017190916910	2.616411382188	0.575629420524
H	-3.488874279549	1.388181062525	1.791464702930
C	-0.954552540889	1.111024879347	2.921381499993
H	-1.765028622647	0.793254108549	3.577290657523
H	-0.179551522828	1.622467883408	3.495386586754
C	-3.126675815891	-1.105303383914	0.606405061079
H	-4.203550511164	-0.940288013418	0.521259360711
H	-2.841729497913	-1.979115781696	0.003060852394
C	-1.443893967659	-1.706693878084	2.352575423483
H	-1.126454840009	-2.580301294332	1.765365424980
H	-1.416080336712	-1.954385582746	3.416421445601
P	0.937942425632	-1.564456899840	-1.149012780906
P	1.583332133930	1.369551296070	-0.675700193624
N	3.438768194716	-0.560814462927	-1.091718428502
N	0.592124225945	0.381738232026	-2.987433433647
C	2.733658656340	-1.768987571499	-0.670212898479
H	2.771690366456	-1.959895688476	0.411043820466
H	3.156623983208	-2.625770439553	-1.201277122183
C	3.262844181186	0.642143482653	-0.281160995764
H	4.035058712732	1.364636798540	-0.557542925391
H	3.307756509383	0.465915905409	0.802140261544
C	1.024167829179	-1.022461593602	-2.954075634852
H	2.036089345025	-1.168986748575	-3.339211246982
H	0.313171164189	-1.613739765886	-3.535025255085
C	1.558073998470	1.403950164072	-2.563220871242
H	1.187899684036	2.379057473472	-2.888110800684
H	2.563388006889	1.235344746146	-2.956651382135
H	4.426799919741	-0.759026057220	-1.207631901068
H	-1.785351932695	2.957740162901	2.502565362252
H	-3.485382686021	-1.707166499582	2.532259441767
H	0.131485536079	0.623524071577	-3.861065909262
H	-0.756410585185	2.435705667310	1.326732155908
H	-0.274179138091	0.365570821465	-1.677067880796
C	1.824326723557	3.177819079982	-0.219682902916
C	0.302604293272	-3.329705735812	-1.234629456407
C	-3.163224654579	0.841991863216	-1.624657369804
C	1.254437526646	-0.766942916003	3.071054531218
F	1.942630875214	3.296502238085	1.124804243013
F	0.748472936401	3.905861601466	-0.599629768565
F	2.914790233340	3.747859126685	-0.775421816310
F	-0.984688453345	-3.339356918063	-1.651529373008
F	0.335660628188	-3.887053925827	-0.000356590530
F	1.003417358416	-4.132772546558	-2.062428471738
F	-4.474882687087	1.056786613438	-1.399929323081
F	-3.054041397731	-0.152072476025	-2.532666865901
F	-2.650958794248	1.963353536910	-2.176162840766
F	0.947134697177	-0.873159113881	4.378911679464
F	1.770714765213	-1.949700512800	2.669901954525
F	2.220877096483	0.169990984327	2.954935699906

(CF_3, H) complex in the $^1B1$ state

E_{tot} = -3423.80134358726
E_{zpve} = 0.386731148596
E_{SP} = -3424.55052349

Ni	-0.144521194539	0.099343210496	-0.180591266769
P	-2.261261451768	0.354221462491	-0.024795843007
P	-0.197792416964	-0.316868220441	1.973303002955
N	-1.515852873693	2.142222683116	1.959461131498
N	-2.739606054803	-1.220514738685	2.076452455291
C	-2.728379263849	1.712299023467	1.171617505970
H	-3.081377418487	2.593967948954	0.633722273401
H	-3.483810241629	1.368858295271	1.878465955994
C	-0.922096511107	1.132256716565	2.908885484955
H	-1.711307086641	0.806269823420	3.586578057664
H	-0.136248113922	1.649805889732	3.461484936135
C	-3.141629778439	-1.131862182805	0.678691574915
H	-4.221585339047	-0.975450619288	0.623950783814
H	-2.864814473672	-2.000157917306	0.063495887666
C	-1.394620728534	-1.705031431893	2.357663099398
H	-1.082842984224	-2.567152774120	1.751003284614
H	-1.322828518604	-1.960249074144	3.417527356877
P	0.944236136212	-1.548934300277	-1.202985413957
P	1.603398513172	1.343721373749	-0.711995580319
N	3.461386033612	-0.614711104724	-0.941096427905
N	0.872134360372	0.361617332672	-3.168492551967
C	2.686939949415	-1.793973906474	-0.566403584719
H	2.629037270949	-1.974140329566	0.514463259322
H	3.125013415858	-2.669652963798	-1.052605860500
C	3.240412160409	0.608054293178	-0.174767299380
H	4.033307647367	1.319598872499	-0.420344456397
H	3.216741147267	0.461968682962	0.911390611077
C	1.215331623636	-1.048630283564	-2.995971158426
H	2.251478679607	-1.293515955133	-3.260051245974
H	0.536313685948	-1.641998363378	-3.614402048793
C	1.769509952025	1.356890164628	-2.587812650544
H	1.459064696848	2.342927452128	-2.943891568268
H	2.828007492942	1.203551905120	-2.831216169442
H	4.450438222552	-0.838757981289	-0.962963314743
H	-1.776710266300	2.970556353087	2.509741612571
H	-3.428385270782	-1.730837818686	2.618428116259
H	0.696119921933	0.568493535427	-4.147087981778
H	-0.784129533714	2.451280819516	1.301708356417
H	-0.444734501546	0.432060421374	-1.558579262615
C	1.821622055754	3.148740285680	-0.236741669963
C	0.282315999701	-3.300384934199	-1.320984951790
C	-3.239169682961	0.820598825088	-1.560561418437
C	1.270534854946	-0.718322037682	3.081792397111
F	1.901416546101	3.254358885389	1.112006837128
F	0.757624279709	3.879432214247	-0.641004363867
F	2.927676624387	3.722154376588	-0.755663412561
F	-0.990009508221	-3.285319204240	-1.780440441958
F	0.266080919638	-3.867187228324	-0.090587201682
F	0.997547058786	-4.108392091549	-2.131405634716
F	-4.539906246317	1.046692124017	-1.293929486166
F	-3.163785396099	-0.180360236493	-2.460620911962
F	-2.733093335888	1.933669428767	-2.128586217212
F	0.913427696787	-0.794507806112	4.379161760435
F	1.805786024894	-1.905046627771	2.730967068607
F	2.228649365068	0.225595395090	2.980965326114

(CF_3, H) complex in the $^1TS_{B2}$ state

E_{tot} = -3423.80058895842
E_{zpve} = 0.384880873313
E_{SP} = -3424.55008003

Ni	0.058372931117	-0.050608001866	-0.295919904067
P	1.587647056186	-1.493372129703	-0.671554084868
P	-0.713190008797	-1.480836364163	1.185478105423
N	-0.622945318736	-3.100389861380	-1.091182529085
N	1.611602495203	-2.755795591102	1.674203103975
C	0.873385141358	-3.108226477930	-1.274855559643
H	1.060562456978	-3.234223155085	-2.342871011569
H	1.278613697102	-3.949843465872	-0.712770049475
C	-1.142079883826	-3.088902524232	0.325841626934

H	-0.709468265187	-3.942935783452	0.847180472064
H	-2.225810956013	-3.197354667414	0.253176622117
C	2.540436427114	-1.992620263749	0.852134753567
H	3.380222442049	-2.630129558994	0.565651635475
H	2.916527072388	-1.070878070830	1.316777618350
C	0.600574296212	-2.009157226056	2.410699295582
H	0.973113980930	-1.097841399768	2.899211256860
H	0.148888066656	-2.664078140896	3.159246972113
P	0.709913443597	1.863240234955	0.741274263818
P	-1.558048757369	1.171471869062	-1.080500348928
N	-1.714496758366	3.060345187125	0.837778097089
N	0.570307206479	2.711636001686	-1.874585770120
C	-0.665645385558	2.631004768266	1.758534093157
H	-0.993296435222	1.890423611877	2.500961701136
H	-0.279929618829	3.511616815991	2.279172177658
C	-2.560939220898	2.030972285949	0.246089084998
H	-3.419720142567	2.512813380290	-0.229123310014
H	-2.921897944700	1.272608841747	0.949491662928
C	1.032641974797	3.176171212242	-0.565794962782
H	0.562808499268	4.110250645118	-0.229543153792
H	2.114316882547	3.325173527212	-0.626203430059
C	-0.871182853424	2.604222022337	-2.078575388025
H	-1.050541124658	2.387508066416	-3.135319783931
H	-1.439249452788	3.499376492259	-1.794550014745
H	-2.286098061102	3.781408441922	1.264179232509
H	-1.002284678056	-3.937214658332	-1.553347205821
H	2.106327904937	-3.396707581808	2.284717080050
H	0.975839627379	3.288703394858	-2.605966594003
H	-1.014860888518	-2.290029434080	-1.596307303623
H	0.575608982711	0.432238502804	-1.558124903970
C	-2.851367267823	0.382429871728	-2.192776608718
C	2.179800547011	2.039759912320	1.894712988756
C	2.884570310258	-1.099761231138	-1.973336442675
C	-2.238280678201	-1.264345585078	2.273322461753
F	-3.584460553652	-0.501202077792	-1.478676751608
F	-2.251062578217	-0.299045735785	-3.195205033928
F	-3.700290986746	1.267262234236	-2.752558360214
F	3.293516905393	1.513402244553	1.333283725134
F	1.942703826713	1.359228947527	3.042209324603
F	2.464954058944	3.315603594833	2.232480234804
F	3.746543556895	-2.117125083999	-2.162111805736
F	3.587692558070	-0.018164980729	-1.582646439545
F	2.308918123131	-0.819592832650	-3.158461144688
F	-2.512780968263	-2.376245202297	2.984502527696
F	-2.036579596740	-0.255744913466	3.145590471773
F	-3.330174087148	-0.973876107669	1.536410305312

(CF_3, H) complex in the $^1B2$ state

E_{tot} = -3423.80297627798
E_{zpve} = 0.3858936525
E_{SP} = -3424.55175807

Ni	0.000013713007	-0.025086136016	-0.392172274760
P	1.619915934050	-1.416747629214	-0.579815848929
P	-0.799536371409	-1.480016139870	1.176709817220
N	-0.503210346160	-3.179129444712	-1.021981129152
N	1.559179784557	-2.639114904396	1.800052710816
C	0.993340908257	-3.079125758319	-1.157347425368
H	1.219454421268	-3.210704188081	-2.217419285740
H	1.439568694591	-3.882529035520	-0.571878468870
C	-1.086455821256	-3.146982979334	0.370882118618
H	-0.624771388992	-3.952187383062	0.942021199750
H	-2.156439749808	-3.328910229874	0.255761985958
C	2.500936900141	-1.864104401460	1.003947742271
H	3.363892323076	-2.488945725534	0.759843952166
H	2.847855537013	-0.937538719441	1.474312175504
C	0.485776291957	-1.915140464704	2.471856987370
H	0.801777363613	-0.978021922808	2.949034457243
H	0.036156862603	-2.570503152941	3.221493894667
P	0.739789838634	1.821057652811	0.620420104812
P	-1.567847358907	1.256610964855	-1.081613962968
N	-1.643009804888	3.050490696827	0.913162180853
N	0.542635539139	2.743534771209	-1.954236214215
C	-0.558609681491	2.553826363115	1.753219739315
H	-0.858600311111	1.775814675921	2.470121918137
H	-0.124411381646	3.395583309028	2.298158119342
C	-2.521298472341	2.060961893452	0.304243613267
H	-3.390657845167	2.568882874127	-0.121849613176
H	-2.864903188753	1.267868985299	0.980393275181
C	1.032749501209	3.175478291229	-0.643257653858
H	0.566682618826	4.095903848378	-0.264421247948
H	2.112053002044	3.329590092453	-0.719833142424
C	-0.911122809882	2.670632133556	-2.105670172871
H	-1.141843776678	2.455408411842	-3.152750981478
H	-1.450874276800	3.577399582127	-1.797533492543
H	-2.185776902811	3.751020565987	1.406708950421
H	-0.794967688001	-4.061478768208	-1.461581369612
H	2.049804427570	-3.244312233666	2.449068536200
H	0.913506841478	3.366720191213	-2.667157753411
H	-0.934310995447	-2.426470892642	-1.579847200290
H	-0.209900025453	-0.480446702318	-1.770777974124
C	-2.899323170105	0.415956494533	-2.107612301290
C	2.281446167793	1.915887410605	1.692362097945
C	2.982927411010	-1.039039293577	-1.823578044808
C	-2.365864668042	-1.336816094560	2.209521267179
F	-3.431991071400	-0.602433472315	-1.396460989002
F	-2.391213028569	-0.097220743036	-3.245015070879
F	-3.897842231222	1.255915076997	-2.442333257397
F	3.372984111776	1.486139418491	1.022947525939
F	2.137230811358	1.137223224455	2.790565569655
F	2.536469281028	3.171818401435	2.115843452074
F	3.817522673006	-2.080429472380	-2.012306316141
F	3.707153268208	0.005277081959	-1.375989610930
F	2.458662902287	-0.705762920465	-3.018414925759
F	-2.655652345628	-2.453407210717	2.909119781041
F	-2.219184525271	-0.325223000792	3.094148041072
F	-3.434127311701	-1.056358973178	1.432605166055

(CF_3, H)) complex in the S_0/T_1 minimum energy intercrossing point

E_{tot} = -3423.77262805955
E_{SP} (S_0) = -3424.51885444
E_{SP} (T_1) = -3424.52084784

Ni	0.035935721008	0.045673560825	-0.663915432201
P	-2.242556607272	0.800582409678	-0.742182017981
P	0.089688639211	1.805463927817	0.912797383066
N	-0.844326727614	3.130081136483	-1.353571646545
N	-2.531033419360	2.085090873609	1.582152513955
C	-2.227960068659	2.543475133480	-1.416654536742
H	-2.513699310525	2.536560420813	-2.469937452251
H	-2.901880728708	3.183503256715	-0.847407999849
C	-0.261174666338	3.401436901724	0.005265328894
H	-0.961181809788	4.021973805135	0.565030468210
H	0.676136178918	3.937655839283	-0.159445384456
C	-3.202764854883	1.022782117234	0.846969450728
H	-4.220351294948	1.340304431118	0.605894711472
H	-3.239201423454	0.056916325427	1.364155883673
C	-1.262820882065	1.759291932355	2.215717917892
H	-1.228763240528	0.767133148226	2.683705117386
H	-1.036064628183	2.518224705026	2.967884978953
P	-0.132982596395	-1.872184457853	0.785212725618
P	2.263208590010	-0.789979297353	-0.792939444394
N	2.497677596920	-2.308278578059	1.408029767054
N	0.641498469552	-2.768154495094	-1.661649008155
C	1.237002656127	-1.998719272217	2.066947067069
H	1.241519320895	-1.058042257001	2.634533217648
H	0.987476920629	-2.818836009489	2.744682978362
C	3.198496744284	-1.203782771949	0.766922088835
H	4.206918373986	-1.529279346529	0.498571099625
H	3.268467278902	-0.290131072135	1.372798711653
C	0.147660976105	-3.301154560613	-0.387703680785
H	0.812455386073	-4.036029446156	0.084876913530
H	-0.826209337933	-3.765002503311	-0.567295915642
C	2.064286653772	-2.409600649537	-1.695695099205
H	2.353170735533	-2.247191124463	-2.737186867186
H	2.731937471926	-3.159345282858	-1.250947888125
H	3.118027093068	-2.796382982498	2.045189497013
H	-0.845554805133	4.011300701359	-1.881187204162
H	-3.163098396314	2.526454799850	2.240844787480
H	0.448552062261	-3.439168284340	-2.400775528466
H	-0.207939071423	2.461737743272	-1.851183302155
H	0.294022814248	0.913951160972	-1.997792679964
C	3.503307856632	0.179022371670	-1.816426134202
C	-1.615322974008	-2.438950699872	1.796225420521
C	-3.416391168905	-0.079062847607	-1.914931706804
C	1.601588877242	2.275353967277	1.928882887014
F	3.713101003493	1.392192394244	-1.263109182182
F	3.035297665485	0.371429878959	-3.067576764690
F	4.699874977324	-0.437276769024	-1.917846253907
F	-2.756194026515	-2.366889748014	1.076072590880
F	-1.761892941491	-1.645368822844	2.882654006381
F	-1.495751763270	-3.708984762292	2.235944858878
F	-4.581716259213	0.569433436669	-2.100667001214
F	-3.692042953932	-1.303272967769	-1.422713509508
F	-2.832835615099	-0.234192942546	-3.120816791790
F	1.490449244888	3.494724985214	2.492039338522
F	1.757501367977	1.373834234632	2.922800702854
F	2.720132985462	2.270492012361	1.179444639396

(CF_3, H) complex in the ^1TS_C state

E_{tot} = -3423.76657548247
E_{zpve} = 0.380407713708
E_{SP} = -3424.51361569

Ni	-0.000703477093	-0.089318048777	-0.392717758031
P	-1.780302497546	0.986905480651	-0.935484360939
P	0.597987930635	1.861557751172	0.663697667433
N	0.118485672522	2.777621760136	-1.940604589701
N	-1.890162606135	2.875439208970	0.955325559028

C	-1.352794915461	2.457940424281	-2.004788644346
H	-1.575153179414	2.229687905534	-3.049000698171
H	-1.903603580208	3.341603822461	-1.683230944087
C	0.693319453501	3.226784022398	-0.617859494245
H	0.141797668080	4.111338915010	-0.299677217741
H	1.736621911082	3.481065114552	-0.814892129897
C	-2.720491195199	1.783566281256	0.467478789438
H	-3.655518733317	2.190752941959	0.074441567542
H	-2.945840490608	1.008535120399	1.208805045083
C	-0.745070167388	2.531808263340	1.790408665297
H	-0.953145493360	1.768141653642	2.551340773524
H	-0.384308047912	3.438971051861	2.281238334186
P	-0.529050969787	-1.625623411619	1.130145705402
P	1.702959512656	-1.342051908971	-0.729891495417
N	1.973202494023	-2.467697290015	1.690930780673
N	-0.233369800149	-3.222390170686	-1.079772763577
C	0.844047177858	-1.855158617973	2.380697109889
H	1.061436794061	-0.871043840027	2.820945060096
H	0.502964323673	-2.533329543551	3.166733039134
C	2.741156461841	-1.615501401703	0.793456406760
H	3.654635251432	-2.139314866586	0.499525454013
H	3.006495907667	-0.631927995273	1.202008504558
C	-0.677365091634	-3.308405365828	0.313138235571
H	-0.117030470360	-4.028646002902	0.925720667864
H	-1.734772333081	-3.586094945046	0.315041450662
C	1.204561551720	-3.051150652473	-1.288031017364
H	1.413761131285	-3.125301035274	-2.358843258038
H	1.836439087661	-3.770279204472	-0.747702770186
H	2.588113695217	-2.928611714951	2.353155834648
H	0.300444345718	3.522001520685	-2.626211696873
H	-2.458503123732	3.591196368688	1.394384239840
H	-0.539418305105	-4.055283718661	-1.576529174764
H	0.648881177004	1.954912514431	-2.265011559759
H	0.235084762799	0.051366607988	-1.833930146292
C	2.932740234027	-0.689897078823	-1.994973548454
C	-2.038160234839	-1.558873422264	2.247714765469
C	-3.064383405266	0.090755657875	-1.979969053557
C	2.146925881808	2.244698393655	1.658885797715
F	3.293558777741	0.570428287155	-1.665346731881
F	2.386080540479	-0.653247052693	-3.226116016944
F	4.050544445003	-1.438172460160	-2.059807180358
F	-3.178235383023	-1.416456862787	1.538291331809
F	-1.941108287944	-0.502235047119	3.088122972466
F	-2.168578840723	-2.668399802311	3.003742173182
F	-4.074132099775	0.890752096040	-2.373053902815
F	-3.585922944504	-0.924787171379	-1.264508699140
F	-2.488275585489	-0.424467148212	-3.083890977417
F	2.299687145926	3.557367841269	1.932985172833
F	2.088053976263	1.584842996410	2.837053825632
F	3.255523259359	1.835734275908	1.005826068700

(CF_3, H) complex in the ^{1}C state

E_{tot} = -3423.76820477818
E_{zpve} = 0.38148868856
E_{SP} = -3424.51562291

Ni	-0.007966374636	0.014600251158	0.504795132570
P	-1.670728125250	-1.453464913212	0.664002122676
P	0.917750131892	-1.661263197092	-0.746530455663
N	0.431966444013	-2.949914595483	1.587368087636
N	-1.386880089852	-2.875258213963	-1.545635176705
C	-1.024283755563	-2.981343422590	1.533183450527
H	-1.413577224089	-2.962169529815	2.553802475493
H	-1.447010066740	-3.842536233947	0.998034445380
C	1.120652140874	-3.182247412295	0.318708860770
H	0.762502513351	-4.055272479219	-0.243649662269
H	2.189076321065	-3.295649700297	0.520375818436
C	-2.421911390577	-2.077725402699	-0.906928738967
H	-3.262859621816	-2.723267438604	-0.638987121900
H	-2.792790129240	-1.230814515003	-1.492901162504
C	-0.260631815029	-2.159689724221	-2.124215752289
H	-0.533167957741	-1.248028353356	-2.674059833556
H	0.274116432407	-2.835281000588	-2.795569070140
P	-0.906060216086	1.677885899892	-0.728546387396
P	1.652505523735	1.483132945355	0.683672490009
N	1.426082811402	2.818467011240	-1.573303675249
N	-0.414020907900	3.055789999582	1.553168726246
C	0.303323069676	2.072112915849	-2.116041714698
H	0.577178211113	1.115944511103	-2.584190990034
H	-0.211824671549	2.696498511283	-2.848896167324
C	2.449780042777	2.048004340921	-0.885709592558
H	3.283259931378	2.703450589007	-0.619142794635
H	2.832948082359	1.180414869018	-1.432232457592
C	-1.083130511326	3.244950191863	0.267462595795
H	-0.696513994978	4.079626709422	-0.333865020513
H	-2.150982670979	3.394208516767	0.448585979646
C	1.044306443844	3.047781704068	1.506813613466
H	1.429491297707	3.048502701057	2.528923713052
H	1.491449011573	3.882555631199	0.948713921576
H	1.837696181521	3.425833638256	-2.273646053382
H	0.759830288457	-3.611090871931	2.286137895668
H	-1.790772728465	-3.524130497744	-2.212237289330
H	-0.723871167462	3.769943942629	2.207144495983
H	0.307514905251	-0.496431009795	2.120428663645
H	-0.115093242612	0.176881200350	2.217463040914
C	3.009609763301	0.809006662916	1.817385146408
C	-2.485127808775	1.606768853748	-1.759543505006
C	-3.074487648015	-0.833406203202	1.769575686852
C	2.518207347510	-1.605851745825	-1.742976013455
F	3.244908015354	-0.491573752687	1.566219017089
F	2.627046845432	0.916997361554	3.103868005520
F	4.159965997995	1.484172098793	1.660300008966
F	-3.579121758739	1.260399117744	-1.065173714949
F	-2.319558936887	0.707889499833	-2.758202523953
F	-2.726331770954	2.806622741938	-2.326824342852
F	-4.138573988194	-1.652776452665	1.705087948612
F	-3.469270936556	0.401385907227	1.418024021237
F	-2.658581876002	-0.783693688539	3.050241106595
F	2.781163972916	-2.811435073114	-2.288374759284
F	2.389936374246	-0.718161253365	-2.757428144946
F	3.588231131459	-1.250035956908	-1.012375727405

(CF_3, H) complex in the ^{1}P state

E_{tot} = -3422.58438504679
E_{zpve} = 0.363346640938
E_{SP} = -3423.33068883

Ni	-0.006671708719	-0.009801149474	-0.178749203409
P	1.748292805106	1.161783086921	-0.864257474020
P	1.544664535041	-1.220190107824	0.859441551568
N	2.607963646345	-1.372624747410	-1.582571164908
N	3.293644278158	0.851782220574	1.220826202587
C	2.976284754698	0.032025301705	-1.732692109877
H	2.950696543424	0.280551942889	-2.795681810229
H	3.964725305398	0.290340495999	-1.327382490509
C	2.833894974306	-1.944752569020	-0.254612396768
H	3.826077630313	-1.746961714494	0.175151721811
H	2.663011414948	-3.023969596088	-0.295705444697
C	2.722373248946	1.971477333375	0.486858637439
H	3.524146969615	2.569014248209	0.046217781318
H	2.051753561512	2.620916130915	1.064912981957
C	2.388896900684	0.026507653202	1.997079960576
H	1.591305759336	0.580534358726	2.512572935063
H	2.962473051687	-0.540786766168	2.733576737944
P	-1.574537891462	1.435742691194	0.435628695775
P	-1.740356784775	-1.364023275804	-0.478982622286
N	-3.253983225152	-0.468126491831	1.439606687342
N	-2.681450134980	0.802451399210	-1.916098161713
C	-2.368492119328	0.575454774962	1.917315673211
H	-1.551939319713	0.224174311546	2.563166778240
H	-2.952479985101	1.325313128988	2.456187122309
C	-2.668109769076	-1.749997986755	1.078296287775
H	-3.461654108480	-2.469301078456	0.863726811654
H	-1.970990856475	-2.171700222129	1.812478468100
C	-2.903703629308	1.743020669715	-0.817289908283
H	-3.881052523039	1.656564460417	-0.321941897950
H	-2.771397699221	2.763229033505	-1.187698252711
C	-3.008809830170	-0.590904878111	-1.626911681805
H	-2.980501944923	-1.150999601018	-2.563944816706
H	-3.986059422783	-0.740998968188	-1.146930041262
H	-4.071355723204	-0.575883324770	2.028492039279
H	3.107411269074	-1.923623729565	-2.276351422746
H	4.102856652459	1.132893624300	1.762688471951
H	-3.211373078699	1.105526647463	-2.729588652972
C	-1.267379035711	-2.928175654965	-1.419494845617
C	-1.110577190172	3.080451840022	1.237398436807
C	1.367291940242	2.410523675691	-2.224527308653
C	1.044253840523	-2.513048580213	2.145539175363
F	-0.300904953012	-3.631395870237	-0.812272618180
F	-0.809673790438	-2.564024511860	-2.637258666235
F	-2.330449691591	-3.733332498091	-1.597027512283
F	-0.560574550737	3.960685040114	0.393791697859
F	-0.217645565200	2.827559956837	2.223630069714
F	-2.190020399896	3.662309140602	1.797682697700
F	2.507964159058	2.885448216189	-2.761781111439
F	0.641124790540	3.452460276834	-1.802530968262
F	0.675408623972	1.784813701844	-3.201498696377
F	2.113571136915	-2.920315870819	2.856728572158
F	0.174414240206	-1.928694381853	3.004348319996
F	0.453643059098	-3.599817117165	1.633195393369

(CF_3, H) complex in the ^{3}A state

E_{tot} = -3423.75113079595
E_{zpve} = 0.389586218242
E_{SP} = -3424.51192262

Ni	-0.102922631551	-0.031757715595	0.188586557262
P	1.445865143234	0.724847112289	-1.230807649647
P	1.620622835684	-1.035218932471	1.593631054497
N	2.352009109695	-1.906544680682	-1.016414921361

N	3.287080449810	1.078690475263	0.779465261069
C	2.585923312254	-0.643563175778	-1.796998597799
H	2.387109562458	-0.884001627038	-2.843847439078
H	3.633774679983	-0.367008314380	-1.675303047068
C	2.797055644070	-1.924213019975	0.421364995074
H	3.791328839791	-1.479080228908	0.458354257714
H	2.854472331398	-2.977090411136	0.707314059769
C	2.558397342318	1.846528401727	-0.220640006377
H	3.278794818333	2.324228366448	-0.891097933808
H	1.905366712045	2.624621947564	0.207490761628
C	2.507381295002	0.613055988064	1.923557372976
H	1.723543646633	1.323721729754	2.229427173246
H	3.192253473092	0.478001305208	2.765479851681
P	-1.782671764740	1.127587350849	1.083225037376
P	-1.745694303216	-1.122040376435	-0.898440787457
N	-3.763730268214	-0.674634267993	0.836970697782
N	-2.395360417347	1.503180554730	-1.609152047265
C	-3.103777500877	0.078854179624	1.895909301437
H	-2.612357260760	-0.541802429397	2.659054132048
H	-3.836250857361	0.730914673673	2.378083345253
C	-3.043927777020	-1.805564538153	0.266619709081
H	-3.744629085603	-2.425547967077	-0.297946815141
H	-2.520207363664	-2.431761924937	1.003400374979
C	-2.776241415255	2.008637015249	-0.239201381584
H	-3.844739514221	1.839803367021	-0.106747180362
H	-2.554547817610	3.077230007683	-0.232771101762
C	-2.739359459458	0.071927275851	-1.948402395764
H	-2.487085843152	-0.058257227855	-3.003032575217
H	-3.810007732763	-0.061552843938	-1.793543108393
H	-4.697791360732	-0.949964359424	1.121016316423
H	2.840676502072	-2.666583303213	-1.505015398324
H	4.076359783921	1.628004075036	1.107638247726
H	-2.855094949502	2.105827431965	-2.303416352516
H	1.351638811463	-2.139407848528	-1.056419039141
H	-1.379241641130	1.634775415274	-1.741230328281
C	-1.448476647803	-2.495962228094	-2.151009502641
C	-1.463730115341	2.519380466896	2.308587848641
C	1.499035321742	1.705455308303	-2.889009357629
C	2.523092291421	-1.967019524315	3.129405632917
F	-0.951314288788	-3.579232210161	-1.515423690571
F	-0.534230484820	-2.095992947754	-3.064846087183
F	-2.552963371908	-2.878005209498	-2.820365533085
F	-0.660943603307	3.450932745634	1.748017855133
F	-0.826180951636	2.018955380796	3.388703416603
F	-2.578169231152	3.143517347208	2.736534699611
F	2.727582490033	1.872376813752	-3.440170830786
F	0.981459866151	2.943370279491	-2.685467373658
F	0.725390697162	1.095226109530	-3.821454138657
F	3.890849762046	-1.970931598601	3.148279836810
F	2.129552684539	-1.378759786820	4.291299284769
F	2.139725342589	-3.270797196725	3.198957809014

(CF_3, H) complex in the $^3TS_{B1}$ state

E_{tot} = -3423.72408122417
E_{zpve} = 0.382906548855
E_{SP} = -3424.47851581

Ni	-0.626033872373	0.091571239303	-0.666041271851
P	0.216672556113	-1.795705622516	-1.422620660158
P	2.228951812827	0.102462576741	0.630592695785
N	1.269938575812	-2.570635921930	1.055479885398
N	2.806946641629	-1.162673093241	-1.838905327256
C	0.833234177429	-3.126875435818	-0.269609497179
H	0.021216671623	-3.825012604327	-0.059734206873
H	1.679488386009	-3.660086826962	-0.704190777430
C	2.503769922005	-1.706779627690	1.077301515108
H	3.234753984837	-2.173111914973	0.417041359611
H	2.863130197215	-1.727103555481	2.107995696571
C	1.633745282494	-1.546238569249	-2.606358525557
H	1.831668754250	-2.487039031287	-3.127573813715
H	1.303291607853	-0.793053386099	-3.338667848401
C	2.765035903133	0.148279398361	-1.209167044101
H	2.062340670429	0.834425906699	-1.701793449933
H	3.756994406947	0.603114284065	-1.250804341160
P	-0.946414820490	2.284628462304	-0.354674519086
P	-2.047994390049	-0.124526907378	1.069800026324
N	-1.955276121209	2.360598186130	2.142031229814
N	-3.371518451200	1.189874847116	-0.904957535109
C	-0.870401046085	2.970797691515	1.383439355690
H	0.135231832156	2.771564272922	1.781542496047
H	-1.030760432546	4.051634125895	1.343225556711
C	-1.783947911093	0.976500897686	2.563868764532
H	-2.542042179431	0.741208940365	3.315813558820
H	-0.789424404423	0.745081675208	2.973052198595
C	-2.759289087098	2.528978693263	-0.821631282768
H	-3.254709016219	3.159042373913	-0.081221544519
H	-2.810298631716	2.989882181270	-1.809687894589
C	-3.680589965807	0.506285814350	0.363760062246
H	-4.317475996433	-0.351957345582	0.141394462945
H	-4.172230169412	1.170662493554	1.076439237037
H	-2.208573830663	2.944041514212	2.932021716779
H	1.459050845966	-3.379024251580	1.661729194023
H	3.644084837066	-1.271436879839	-2.400828715205
H	-4.156630706970	1.166577817969	-1.553578856409
H	0.483129951585	-2.080807692170	1.499888656138
H	-2.286110666899	0.408302395770	-1.402249081932
C	-2.479310344206	-1.771524393802	1.864951808032
C	-0.004017963412	3.550013348299	-1.370413590736
C	-0.960502165669	-2.837213600890	-2.465636278336
C	3.915029963951	0.600442037135	1.359917373079
F	-1.557748305601	-2.054221705281	2.822332056877
F	-2.440719471772	-2.771628572699	0.959681899581
F	-3.691644442436	-1.794014766348	2.450797798128
F	0.136252613631	3.119083919312	-2.643105456746
F	1.233219922854	3.704634451437	-0.847530547895
F	-0.589796370602	4.763217564366	-1.411177232645
F	-0.456405373403	-4.054294328262	-2.763426573216
F	-1.211084750545	-2.198970777221	-3.627878365331
F	-2.136522634993	-3.026632522001	-1.833914538779
F	4.964595499805	-0.151240296188	0.942458009726
F	4.190443326026	1.881896067324	1.020996333325
F	3.898514179134	0.529785452329	2.712294830002

(CF_3, H) complex in the $^3B1$ state

E_{tot} = -3423.76242174646
E_{zpve} = 0.383579057214
E_{SP} = -3424.51122358

Ni	0.004287891863	-0.074020408332	0.720705813994
P	2.231194646683	0.954233848975	0.719142255083
P	-0.284773210750	1.862330650772	-0.753249269709
N	0.746081247826	3.174988699557	1.523020341278
N	2.292106405189	2.321783294729	-1.559993489893
C	2.161668569431	2.653075709356	1.487721942580
H	2.503543860684	2.613624837611	2.523055197456
H	2.761982549049	3.363631417691	0.919333269271
C	0.066984703037	3.432873078180	0.201408345545
H	0.709152770716	4.098207591820	-0.376346557657
H	-0.877154824235	3.927474519667	0.436801624472
C	3.096244101545	1.305174691971	-0.898241315387
H	4.090861337539	1.708773544749	-0.694288284613
H	3.195460536168	0.361602152995	-1.447145728979
C	1.014858449487	1.913249785020	-2.119728308429
H	1.017774782518	0.921582404353	-2.589365455549
H	0.697356272984	2.656756291362	-2.853877950247
P	0.247302231710	-1.851057851627	-0.823237912390
P	-2.196068160207	-1.047104681956	0.709867265766
N	-2.316494148507	-2.119879926024	-1.740814668812
N	-0.787886039570	-3.345133291851	1.256728175732
C	-1.002985731221	-1.723316882618	-2.226379956514
H	-0.955630217826	-0.701948277217	-2.626803881383
H	-0.699518236004	-2.421433407396	-3.010320117759
C	-3.059090927946	-1.152183743731	-0.942817668650
H	-4.072654969876	-1.530836335148	-0.784081840180
H	-3.121447868954	-0.147874590538	-1.376819939950
C	-0.144998757342	-3.511791284897	-0.041086010405
H	-0.729951802761	-4.082802262022	-0.776318982018
H	0.810780821191	-4.018590417522	0.119611845199
C	-2.156327533694	-2.836594234086	1.261189295213
H	-2.529848401463	-2.874792000309	2.287927891216
H	-2.852111855121	-3.388547700787	0.613142677988
H	-2.887899270401	-2.466432962569	-2.503782351217
H	0.761391992397	4.067460792762	2.034687645818
H	2.840941226582	2.840018336896	-2.236874305869
H	-0.758173296838	-4.228974071234	1.757762103055
H	0.163078569581	2.543377083949	2.090597984419
H	0.344245400989	-0.713647532290	2.124144970344
C	-3.471456595743	-0.258987008845	1.835889902428
C	1.809857322475	-2.235492684752	-1.801144316235
C	3.503133523227	0.099515811755	1.809838878102
C	-1.835214660983	2.412316468021	-1.678184116420
F	-3.758408320477	0.989707616431	1.406676867445
F	-2.979447369826	-0.159779266910	3.090663134295
F	-4.630117881302	-0.945727178717	1.905516630990
F	2.904891395700	-2.219497458081	-1.013828871144
F	1.988847449353	-1.309033041620	-2.773779245895
F	1.759635548736	-3.442676782027	-2.402018400575
F	4.734533307588	0.634637147166	1.699645452103
F	3.584428830554	-1.202396716555	1.477911348495
F	3.130812003588	0.180960405672	3.103352886728
F	-1.736996736857	3.664042999114	-2.169096155516
F	-2.053719159275	1.580159330062	-2.720105998673
F	-2.915844979111	2.369119852397	-0.875021903943

(CF_3, H) complex in the $^3TS_{B2}$ state

E_{tot} = -3423.76240144865
E_{zpve} = 0.383408999364
E_{SP} = -3424.51128785

Ni	0.006560232252	-0.073006939179	-0.731204168617

P	-2.301250178514	0.755308851150	-0.756492321528
P	0.109256248608	1.884189969530	0.738341906342
N	-1.009216117314	3.096479042897	-1.552112995654
N	-2.505853626073	2.117474542926	1.517464034288
C	-2.373612592902	2.451843444600	-1.529825633865
H	-2.699602470748	2.379981762585	-2.568540780804
H	-3.040261553564	3.108052321681	-0.970096543678
C	-0.369106566906	3.415825224248	-0.224455377469
H	-1.073202542358	4.023349490614	0.344762293959
H	0.530275002684	3.991034959660	-0.451771196289
C	-3.210604600519	1.032568647561	0.850845115041
H	-4.234666329224	1.346683696762	0.635457869793
H	-3.232205450308	0.085197660254	1.401695647653
C	-1.203615530664	1.824177490333	2.091710597800
H	-1.124426984749	0.837289637303	2.564645007006
H	-0.960339271069	2.594547298011	2.826494461096
P	-0.096039521376	-1.860211827827	0.816506239324
P	2.281183094989	-0.851946724327	-0.697820519197
N	2.474254397705	-1.905703402026	1.754981248184
N	1.080598785764	-3.264042888782	-1.251107901584
C	1.127601410769	-1.621935688857	2.228977357946
H	0.989826158889	-0.607350839187	2.625690066789
H	0.878825749637	-2.341442425149	3.012834584293
C	3.138466935023	-0.879310527430	0.961135738586
H	4.182311344763	-1.169725915946	0.812317927589
H	3.109948639499	0.127377421968	1.393169141437
C	0.445055975352	-3.482625569971	0.042552729337
H	1.072002160797	-3.998582280030	0.783838748351
H	-0.461715717360	-4.071183981235	-0.123358509913
C	2.399480480186	-2.638166938055	-1.247905764857
H	2.781207335589	-2.644557747847	-2.272313852769
H	3.136756971686	-3.127104262392	-0.595080898732
H	3.066681935224	-2.201838293318	2.523108348417
H	-1.097724843272	3.983081158786	-2.066553479829
H	-3.105183001896	2.587328499704	2.186779355061
H	1.131619128455	-4.148380098607	-1.749540169746
H	-0.366975041109	2.517508721613	-2.111826002444
H	-0.260260764709	-0.743004707057	-2.136393412845
C	3.485261827053	0.044744710890	-1.820747327742
C	-1.627746086929	-2.376898626946	1.782237414582
C	-3.481499550121	-0.211169043496	-1.856844616058
C	1.597404273413	2.568673083017	1.675590372408
F	3.713438371039	1.291404530606	-1.353090291979
F	2.957242839965	0.160480717835	-3.059550201161
F	4.678164712702	-0.572545467435	-1.942439401316
F	-2.712641239760	-2.458962868528	0.984917802075
F	-1.896164323997	-1.466794825471	2.749688917637
F	-1.477926826806	-3.573315275732	2.388032303327
F	-4.756444808832	0.213222481270	-1.760911631219
F	-3.450769555078	-1.514268435448	-1.520870413940
F	-3.104577616671	-0.100786960232	-3.146871564986
F	1.386241895159	3.807513903424	2.163494861143
F	1.879181709235	1.759764877232	2.720608583719
F	2.684247096382	2.619182414055	0.881202305039

(CF$_3$, H) complex in the ^{3}B2 state

E_{tot} = -3423.77268586821
E_{zpve} = 0.383482252746
E_{SP} = -3424.52088405

Ni	0.039303504089	0.045749749325	-0.669309131757
P	-2.254028045996	0.799244138147	-0.740313430021
P	0.086131579884	1.806916530143	0.904194036615
N	-0.855132384689	3.125771214587	-1.360730244114
N	-2.532477949503	2.086084190750	1.583516935372
C	-2.239933363150	2.541175508826	-1.417818975722
H	-2.529175687978	2.532670957739	-2.470116063938
H	-2.911175482727	3.183416808899	-0.847851887858
C	-0.268219730874	3.400908770020	-0.004541858895
H	-0.966787181715	4.021807946572	0.556691821965
H	0.668502207328	3.937134113130	-0.172722702600
C	-3.209411669881	1.024908220963	0.851247017461
H	-4.226985515171	1.344953759960	0.613569040155
H	-3.246653532281	0.059450031342	1.369416757405
C	-1.262157073126	1.758064553969	2.211287265623
H	-1.226608593659	0.764944538402	2.677173086698
H	-1.031733598741	2.515273589300	2.964136356076
P	-0.129409807967	-1.870258835885	0.790970775160
P	2.280963633874	-0.784711261378	-0.780358503804
N	2.501345899174	-2.317344105419	1.412375235812
N	0.642460502998	-2.740957596305	-1.661322560199
C	1.241291980356	-2.005352379119	2.071434021655
H	1.248715832300	-1.066067211969	2.641336523541
H	0.988931821498	-2.826136540241	2.747332977879
C	3.209995023134	-1.212733096739	0.779706780304
H	4.217775627759	-1.541782676888	0.513438297808
H	3.281963834448	-0.302701880702	1.391064010079
C	0.146248796455	-3.288920416473	-0.394273367609
H	0.808190272596	-4.032489943953	0.068289894864
H	-0.829509489355	-3.746320775655	-0.579769335257
C	2.069252951528	-2.397491594896	-1.692326303132
H	2.360618364817	-2.231550157392	-2.732482141196
H	2.727946515987	-3.157570414798	-1.252135278971
H	3.117734382404	-2.813276352044	2.047325770422
H	-0.856000974351	4.004846765548	-1.891802944552
H	-3.160712575380	2.528748011127	2.245003552568
H	0.439464661502	-3.396537017048	-2.411387350965
H	-0.220554698193	2.453397954885	-1.857180214161
H	0.295232119098	0.918442434680	-2.006825063385
C	3.529603980637	0.173927635875	-1.803496980874
C	-1.613404087470	-2.439194921943	1.798020742663
C	-3.432729179587	-0.079815948112	-1.908413144852
C	1.601867214614	2.273721759995	1.915936623920
F	3.751392749207	1.385523399868	-1.252218332410
F	3.060130388625	0.369330978386	-3.054057802433
F	4.720735565944	-0.452442833940	-1.907836891633
F	-2.752555415092	-2.366141151461	1.075009754428
F	-1.762881911438	-1.647034388710	2.885109148379
F	-1.495089864809	-3.709804695848	2.236349993375
F	-4.598586644420	0.568935415344	-2.090500578705
F	-3.707433057514	-1.303901726622	-1.415322898857
F	-2.853369114767	-0.235282023056	-3.116441799315
F	1.494223881348	3.493185253670	2.479067999909
F	1.757884650475	1.371722717721	2.909378494979
F	2.718490792119	2.266378673737	1.163861458108

(CF$_3$, H) complex in the ^3TS_C state

E_{tot} = -3423.76317575683
E_{zpve} = 0.379467907841
E_{SP} = -3424.51105122

Ni	0.042988799372	0.048764417663	-0.617514969322
P	-2.241487021317	0.811100225401	-0.776653434558
P	0.077990461345	1.837719325333	0.946560959188
N	-0.799188551817	3.084432914650	-1.360726344772
N	-2.549152506676	2.084533469319	1.564231960542
C	-2.182747891695	2.561864428898	-1.441583049630
H	-2.467506236302	2.545646203577	-2.495678206568
H	-2.879368855999	3.185958018360	-0.877017604792
C	-0.264993804115	3.408105476854	-0.017441415245
H	-0.960248636420	4.033928610741	0.547276148464
H	0.681614454292	3.934980688052	-0.159883407622
C	-3.188748392128	1.009869425296	0.821705571691
H	-4.212858456438	1.303612580103	0.577663025964
H	-3.206383596740	0.041702875459	1.334954271954
C	-1.290336985991	1.784422302259	2.226453103234
H	-1.251367644743	0.799691127408	2.709902009219
H	-1.087104932118	2.557094874761	2.971644797063
P	-0.125258493797	-1.909013840504	0.814778027033
P	2.263871937539	-0.792364363528	-0.823186034694
N	2.510510371115	-2.310075237885	1.382567879128
N	0.618692397978	-2.764464094207	-1.655538629050
C	1.262401586655	-2.027216518616	2.073950155611
H	1.263485903588	-1.090716118379	2.648321212601
H	1.036683838330	-2.857061705997	2.748187225709
C	3.188637008453	-1.190286747884	0.747622485665
H	4.204323712812	-1.492684994299	0.480329257278
H	3.237224471130	-0.276872394594	1.354816150900
C	0.141290352014	-3.317831921609	-0.383203015595
H	0.812229638245	-4.059390535975	0.069313819855
H	-0.834890761412	-3.778974492734	-0.556856796441
C	2.043670248078	-2.416044120111	-1.711479493995
H	2.318769300872	-2.257989246338	-2.757463934470
H	2.713386311484	-3.166977796595	-1.272779083605
H	3.148153626282	-2.815725711967	1.987691084938
H	-0.700910301224	3.896032260556	-1.972759484419
H	-3.202007787803	2.523183792451	2.203878528107
H	0.404444607126	-3.417428047832	-2.404710572419
H	-0.051581852202	2.095508411288	-1.828422769873
H	0.424026613843	1.237440272578	-2.057305346539
C	3.492645462636	0.186057471543	-1.851065393791
C	-1.613805439832	-2.449814035226	1.826808652811
C	-3.409224750981	-0.079509075865	-1.943836135289
C	1.596121607587	2.282077800485	1.959452781928
F	3.701532172692	1.393576299594	-1.287040725880
F	3.011580128927	0.386001092643	-3.095064387022
F	4.687431250980	-0.428489343092	-1.965347438289
F	-2.750673972033	-2.367343391718	1.102252023941
F	-1.750621533150	-1.646871342923	2.906162939366
F	-1.507688669781	-3.717616626189	2.272571770479
F	-4.570095559018	0.574087913284	-2.137324905788
F	-3.692848488254	-1.299697918600	-1.445676569155
F	-2.821091508283	-0.244546070192	-3.146242250527
F	1.504914816054	3.500425387689	2.526569258941
F	1.749029917591	1.375328314868	2.949294378744
F	2.709257633247	2.265355711749	1.200360919001

(CF$_3$, H) complex in the ^{3}C state

E_{tot} = -3423.77727865428

E_{zpve} = 0.376028099716
E_{SP} = -3424.52461646

Ni	-0.004680983161	0.028429351838	-0.503002953960
P	-2.188462374571	0.810604577068	-0.800265420451
P	0.101622991489	1.825495145858	0.997207025681
N	-0.556494917523	2.874391913206	-1.412137498546
N	-2.530990529440	2.146059624529	1.504912010039
C	-1.967219586853	2.499824599243	-1.555781696803
H	-2.194289334220	2.409918629203	-2.621221653723
H	-2.675970620052	3.199034987320	-1.093467703365
C	-0.135725966740	3.327353309724	-0.083135720225
H	-0.834978752233	4.015143363602	0.409736631574
H	0.838368128684	3.814085993985	-0.178683661835
C	-3.162615771536	1.061336727871	0.772622121609
H	-4.179101795592	1.354813356044	0.499682940730
H	-3.197748655645	0.104398826052	1.307007773897
C	-1.308616830780	1.841068408879	2.229579660634
H	-1.312902866346	0.874351537486	2.750214685387
H	-1.114833065942	2.635595915944	2.954202631524
P	-0.129807910361	-1.910006115648	0.834953539398
P	2.186642389600	-0.733440168465	-0.847485489144
N	2.503035215431	-2.297105137865	1.311424752867
N	0.544739874465	-2.715386836169	-1.666745277978
C	1.283243445055	-2.041426010630	2.058388257589
H	1.295813982804	-1.118390091632	2.652560663262
H	1.083146557321	-2.889209182177	2.718302320584
C	3.159150798554	-1.161088044516	0.686842808558
H	4.163384753492	-1.456340786774	0.372195508550
H	3.232473986129	-0.265396295253	1.316044258464
C	0.108857400846	-3.295909842729	-0.393257816759
H	0.797032859951	-4.038290910066	0.030835970460
H	-0.868728967340	-3.760905906812	-0.547278098618
C	1.958323137948	-2.338358212243	-1.767470404589
H	2.189328991770	-2.150039341297	-2.818885033278
H	2.660027444359	-3.083862522424	-1.371822881367
H	3.160181405842	-2.843312511248	1.856772030326
H	-0.325701705972	3.586849867215	-2.099483541631
H	-3.199977254679	2.620350779779	2.100931959929
H	0.308793278391	-3.348696082181	-2.425734969052
H	0.365127425558	0.298969547377	-3.294290017016
H	-0.321856906155	0.012653038613	-3.287185805726
C	3.352296507927	0.315925848405	-1.881489592251
C	-1.634224117283	-2.411148286262	1.842840383979
C	-3.332008980715	-0.137788518051	-1.949106594127
C	1.607836401554	2.223439479168	2.046567602127
F	3.554912632928	1.502453605296	-1.274675914190
F	2.817604370717	0.557308877183	-3.096332121037
F	4.551037265692	-0.268497368000	-2.065108606994
F	-2.762601062106	-2.317932614200	1.108809820423
F	-1.763939115202	-1.596577824587	2.912290295540
F	-1.545185550641	-3.675791279243	2.296975401640
F	-4.517148879111	0.473686424330	-2.131632391999
F	-3.567580171418	-1.364900608519	-1.442505897677
F	-2.755906655833	-0.291508435231	-3.159892663443
F	1.513967354652	3.433266499222	2.631762843084
F	1.744191937237	1.301602523632	3.025117542871
F	2.733672376160	2.214574744779	1.304564772472

(CF_3, H) complex in the ^{3}P state

E_{tot} = -3422.59315243086
E_{zpve} = 0.361435643448
E_{SP} = -3423.341671

Ni	-0.041952849501	-0.112581547234	-0.473830009414
P	1.773947287138	1.219319162005	-1.136912469622
P	1.579072332658	-1.007388059529	0.984036420758
N	2.575664783934	-1.344743858523	-1.512914839693
N	3.308939038082	1.055154461075	1.060172728032
C	2.999906729120	0.016663509004	-1.863120826346
H	2.951844387289	0.121856045510	-2.950100010371
H	4.006925462574	0.284359406749	-1.517582078435
C	2.850641960282	-1.782205487471	-0.138284503595
H	3.854584457277	-1.532120375968	0.228414069115
H	2.710310374646	-2.865347913463	-0.088943683006
C	2.668213163093	2.082612540224	0.255705138785
H	3.434881392732	2.726948620724	-0.181668049389
H	1.941807251803	2.703394085597	0.795035764214
C	2.470547288511	0.302390850583	1.979928630146
H	1.709174893455	0.902057652150	2.497761533828
H	3.102676944978	-0.192585037792	2.721197398326
P	-1.490643886780	1.229134370103	0.818114864867
P	-1.873233251326	-1.597727281028	-0.568081020559
N	-3.448389271501	-0.563186202734	1.339005785244
N	-2.160121130841	0.773972274219	-1.723181361549
C	-2.594211673946	0.367413528831	2.058312672807
H	-1.946896983015	-0.098508437431	2.814739915115
H	-3.216030600097	1.125542086889	2.540763159268
C	-2.889102379410	-1.854764406086	0.973680032053
H	-3.700759343879	-2.548457001082	0.741778926630
H	-2.235303080442	-2.305710770410	1.732858470376
C	-2.537646518059	1.707549523549	-0.646290840496
H	-3.605221399384	1.701693834569	-0.401793000218
H	-2.235460337449	2.714339849196	-0.944847645331
C	-2.855031255886	-0.527670860079	-1.732260610189
H	-2.774721175323	-0.956214121605	-2.734362834407
H	-3.909315862073	-0.475103250884	-1.441067549230
H	-4.343479112315	-0.669915837696	1.801991557363
H	2.997628328718	-2.002916376619	-2.162668416785
H	4.130709148960	1.419136069872	1.528913612379
H	-2.238267312046	1.227214448244	-2.629319939282
C	-1.718528519216	-3.279001580923	-1.389382320136
C	-1.019660347180	2.845090822442	1.651159355008
C	1.465493487595	2.534832993219	-2.437269532566
C	1.079803235977	-2.320894455607	2.226592645652
F	-1.117524815241	-4.150999638731	-0.557685709595
F	-0.960421585913	-3.172276694835	-2.502234821402
F	-2.908410808050	-3.797028113724	-1.748474923416
F	-0.323012969458	3.629314029121	0.800653624703
F	-0.243018799638	2.602269621731	2.728088404353
F	-2.092191343858	3.545194809077	2.066976968365
F	2.590770029450	3.122636562861	-2.883301197541
F	0.667574187482	3.495143164738	-1.924678416442
F	0.831693582368	1.991006798231	-3.498076933085
F	2.118950955436	-2.861852123142	2.889077123697
F	0.243867777975	-1.774859323348	3.138059614465
F	0.418928290284	-3.322077694560	1.608073756570

[m3, Me] complex in the ^{1}R state

E_{tot} = -2465.46039162662
E_{zpve} = 0.582310615629
E_{SP} = -2465.81974761

Ni	0.017703630439	0.013118516501	0.248712562249
P	-1.584009167023	-1.302017294070	0.811757397391
P	1.615744463227	-1.431960449295	0.067261980208
P	-1.555584040071	1.330319396685	-0.427811318854
P	1.581426342603	1.479133514765	0.383433891659
C	-1.187978843268	-3.072647969218	1.227677451657
H	-0.765361261357	-3.100886185859	2.239062079283
C	1.286728410825	-3.261730832341	0.331185865663
H	1.658683706466	-3.521913905549	1.329891786619
C	1.232818194310	3.324086801673	0.329446531011
H	1.766819097685	3.754268717398	-0.525804001492
C	-1.158913750728	3.109635778144	-0.813704228394
H	-2.081655042729	3.696049302531	-0.898971681099
C	2.837371729371	-1.180138948810	-1.335139698792
H	3.881221463943	-1.218690749418	-0.978352378764
H	2.689684881709	-1.990140936614	-2.057798267712
C	2.675585148779	1.305130156023	-1.189815632106
H	3.725519934911	1.456378556293	-0.880645384811
H	2.398544325940	2.145974210024	-1.832271090146
C	-2.748878209060	-1.600656995407	-0.684011355539
H	-2.584120182527	-2.635973059364	-0.995412063151
H	-3.785460381089	-1.530933382041	-0.312029552355
C	-2.795786851120	0.676348476115	-1.666752809268
H	-3.832357957567	0.881129307561	-1.346533397236
H	-2.621252179074	1.193366528468	-2.616846037562
C	-2.703715637072	1.602318464473	1.063105482414
H	-3.350168142700	2.453844484908	0.822642318563
H	-2.063974164792	1.892148624099	1.914989489624
C	-2.912537221451	-0.702255619287	1.987804780565
H	-3.672755532569	-1.477652813972	2.148248002520
H	-2.437445956373	-0.477004636412	2.959818807152
C	2.802405699045	-1.184118750849	1.527511068969
H	3.466929749531	-2.054863858369	1.546257294161
H	2.185842120369	-1.210990553587	2.446345793608
C	2.966309235721	1.290727199489	1.633745178944
H	3.704616687587	2.092020249768	1.502176892542
H	2.522612453181	1.395402972772	2.641352766957
N	-2.544017080537	-0.760414639637	-1.873651369696
N	2.516579159319	0.090487721094	-2.005906366162
N	-3.577712645362	0.469379562191	1.396754417305
N	3.648735842165	0.007637509509	1.444419570941
C	3.283094563013	0.221764151733	-3.244696127774
H	3.099556085387	-0.647438796433	-3.885459000155
H	2.964923875805	1.119073556567	-3.786625140290
H	4.374098413270	0.296448862136	-3.075535086452
C	-3.352256032703	-1.262875801291	-2.984731091432
H	-3.149065547397	-0.673393414938	-3.885129806170
H	-3.090352889953	-2.306048487048	-3.192306380544
H	-4.439682488262	-1.218136810663	-2.785529417227
C	-4.687743392887	0.903355315429	2.244072388316
H	-5.253390246066	1.695259091152	1.741937276155
H	-5.364124324483	0.060872883056	2.423792412131
H	-4.355263138996	1.288665575807	3.226282928629
C	4.804242355791	-0.098584232274	2.333587226459
H	5.370793290468	-1.006787622508	2.102493939043
H	5.463961990119	0.762760359276	2.183651121270
H	4.523486194595	-0.130965195510	3.402929490135
C	-0.256907103355	3.738607689564	0.283272551426
H	-0.277120755839	4.826809474429	0.143314990210

H	-0.654489454421	3.136046060138	-1.787123325452
H	-0.704645560277	3.556962788027	1.266305394180
H	1.679054121362	3.762314218365	1.230132873935
C	-0.190890449274	-3.689702511732	0.210086132712
H	-0.223490651309	-4.779116917777	0.337070046037
H	1.888084939246	-3.829716444286	-0.388376511997
H	-0.538836184082	-3.493938551850	-0.811605571140
H	-2.112712830385	-3.663328049767	1.236261991112

[m3, Me] complex in the ^{1}A state

E_{tot} = -2466.43603023172
E_{zpve} = 0.613476575323
E_{SP} = -2466.78521875

Ni	0.012667892423	-0.001154937370	0.255175751345
P	1.560628348336	1.479490769191	0.028258804747
P	-1.606076011343	1.309861471903	0.795810412859
P	1.591138335103	-1.462082674280	0.436770534256
P	-1.486559485277	-1.401623885049	-0.394474521636
C	1.204024638721	3.296648645948	0.245468526743
H	1.585662699464	3.562687656776	1.238402529890
C	-1.257950688904	3.095249867340	1.165643159146
H	-0.838798704199	3.154149447499	2.176187779386
C	-1.024229709447	-3.157300269022	-0.763901775367
H	-0.467060443939	-3.162161048301	-1.707623342606
C	1.300777558969	-3.306163435422	0.495644489448
H	1.696150010362	-3.647764007400	1.458838872536
C	-2.665913965337	1.566225472849	-0.783702653936
H	-3.717256615182	1.365183773726	-0.579262642363
H	-2.548426121431	2.595740582723	-1.120820163517
C	-2.605304225030	-0.767681675348	-1.756321836566
H	-3.669031821538	-0.837024564774	-1.525918195209
H	-2.377338562926	-1.283020272623	-2.691536747858
C	2.607118128962	1.185306880332	-1.507860567524
H	2.403530407120	1.955858737758	-2.253962845296
H	3.676110553642	1.106608776733	-1.307891034537
C	2.488131451246	-1.350665936373	-1.258663765843
H	3.572309506400	-1.367649182345	-1.137665974322
H	2.168656206672	-2.206213456974	-1.854561221311
C	3.094643667519	-1.178686985103	1.506657288565
H	3.813756081998	-1.987802125673	1.335239778964
H	2.766078602238	-1.224915184991	2.560249378045
C	2.900624902234	1.277424857306	1.338650894745
H	3.545579151794	2.158727772582	1.263313898784
H	2.393979453820	1.310983678698	2.320171658890
C	-3.003607655562	0.688364114653	1.867637427822
H	-3.768701362245	1.464611969126	1.983482499034
H	-2.590347256375	0.450216454938	2.862599629040
C	-2.743151501334	-1.628681668626	0.997401772433
H	-3.371220717795	-2.482391982202	0.722990183177
H	-2.174532267711	-1.899680741673	1.903195527895
N	2.142450165667	-0.132056970051	-2.085913001683
N	-2.261865043963	0.691129341250	-1.947602134480
N	3.737535654443	0.091401971865	1.163058694720
N	-3.624265143132	-0.474027329004	1.212899162864
C	-2.780219830486	1.221830771483	-3.244972160359
H	-2.418711338471	2.242975583628	-3.371502672247
H	-2.409963269112	0.587661983923	-4.051352599839
H	-3.870228637157	1.205789846624	-3.219248624897
C	2.577503338158	-0.300211657647	-3.506181021254
H	2.124548478606	-1.209118270085	-3.904211889140
H	2.242896673671	0.568079571318	-4.075251310880
H	3.664921670796	-0.375180082022	-3.534497041652
C	5.016393011747	0.232718228015	1.863152878899
H	5.648662826227	-0.633685599094	1.644829450910
H	5.532534367290	1.131605267841	1.511920605703
H	4.897625498859	0.305965610381	2.958690648469
C	-4.839813030574	-0.874035016487	1.926634701428
H	-5.513626000238	-0.015452055385	2.008624834372
H	-5.354127476102	-1.661761442889	1.367357783128
H	-4.633369082075	-1.249551048580	2.944452798539
C	-0.173186053607	-3.758524564505	0.385122582669
H	-0.165053540906	-4.846760622074	0.255847070458
H	1.908691982328	-3.782358655405	-0.282135043097
H	-0.674675580944	-3.573145884967	1.340864711609
H	-1.931261140721	-3.755631458302	-0.900402618378
C	-0.275980777642	3.713021164068	0.135447921967
H	-0.311236362564	4.800901121591	0.265979487393
H	-2.198537284494	3.658407429957	1.157277326807
H	-0.632893962813	3.520875071601	-0.883317941445
H	1.800258755109	3.854508055930	-0.484950212700
H	-1.216943592035	0.717416302726	-1.961000927366
H	1.096149897689	-0.080033161370	-2.051473656846

[m3, Me] complex in the $^{1}TS_{B1}$ state

E_{tot} = -2466.42202196932
E_{zpve} = 0.606155473886
E_{SP} = -2466.77077315

Ni	0.022218148877	0.010253916236	0.115959363065
P	-1.550931845275	-1.457908735680	0.088628362696
P	1.632646542045	-1.326741386773	0.757178040650
P	-1.541700343556	1.487001272612	0.404368759454
P	1.549758774116	1.413829893416	-0.416464338350
C	-1.200454088898	-3.273217156416	0.294815061218
H	-1.571537907677	-3.536239990854	1.292057537509
C	1.265318539921	-3.095000081008	1.171073660218
H	0.862026662997	-3.141376953609	2.188536727146
C	1.092761864649	3.170852560471	-0.753413050333
H	0.532201571922	3.193120748684	-1.694794503647
C	-1.224085916615	3.324528480517	0.471203881678
H	-1.653282452282	3.675775008009	1.415722277712
C	2.606310135232	-1.585646004277	-0.880201475323
H	3.666234460554	-1.384802346364	-0.712585383825
H	2.481728783774	-2.621990055604	-1.192533545305
C	2.531044902908	0.681543773841	-1.847164004678
H	3.607530462806	0.741400065606	-1.674447463113
H	2.262223095986	1.214654257414	-2.761749137345
C	-2.659208869836	-1.179360321385	-1.400248133436
H	-2.485565484618	-1.964532636025	-2.138068503361
H	-3.717235937500	-1.114245360126	-1.144202605963
C	-2.565877429623	1.351623108260	-1.201134213092
H	-3.636383474686	1.337132846758	-0.992076644031
H	-2.320225274284	2.208792490000	-1.828673823619
C	-2.929476723871	1.222474361490	1.618869683334
H	-3.645049654020	2.045077253043	1.511686897013
H	-2.497183877374	1.268936357782	2.634346770976
C	-2.793057844452	-1.228241973116	1.477073692102
H	-3.449611544280	-2.103490070685	1.447574786600
H	-2.221797658443	-1.263932449390	2.422301067126
C	3.047620239445	-0.720471375608	1.804552687486
H	3.794475212255	-1.516403795192	1.905412289597
H	2.663879161905	-0.474145132674	2.809644974194
C	2.836816705361	1.601589360212	0.941436365778
H	3.480884522924	2.436084456847	0.645026704233
H	2.299784808380	1.898505940696	1.858663813790
N	-2.242092833953	0.128065285876	-2.026488192717
N	2.063613911872	-0.712490001853	-1.940625188516
N	-3.622557713947	-0.034863994056	1.344614292793
N	3.688616746207	0.424205006712	1.139779275486
C	2.079146854909	-1.288925056339	-3.292179199993
H	1.563707542213	-2.252555439617	-3.266625833078
H	1.542509302252	-0.616371503009	-3.966650250780
H	3.101271572531	-1.433250622859	-3.658527676250
C	-2.751444051295	0.261428156404	-3.426083995577
H	-2.333703390491	1.169746741467	-3.861746011430
H	-2.428953186231	-0.611823667892	-3.994394775661
H	-3.840177540967	0.317644793272	-3.400775936429
C	-4.848634251552	-0.144088768749	2.139416351423
H	-5.481182353343	0.729155593668	1.952561803770
H	-5.401989235516	-1.039638464360	1.840591852520
H	-4.649939026956	-0.202730163154	3.223787708280
C	4.924200093085	0.791589661668	1.836221387667
H	5.576532324604	-0.084346258157	1.906335331678
H	5.450320688549	1.566909534560	1.270689589127
H	4.742429574075	1.169987394745	2.857741607455
C	0.252626374837	3.761945841056	0.404492693230
H	0.261334884274	4.852973375250	0.303570402059
H	-1.797296810768	3.797136737378	-0.333673669594
H	0.738126090482	3.541904678949	1.361127810718
H	2.008656689166	3.755307390171	-0.888800679170
C	0.267238256107	-3.715951996571	0.163053241855
H	0.284501656697	-4.801938467846	0.309568325506
H	2.203692478824	-3.661511812667	1.153154015263
H	0.610333160046	-3.544189148155	-0.863055932918
H	-1.819371315981	-3.813444306243	-0.429767634715
H	0.757788117343	-0.520793888752	-1.457151771621
H	-1.192509921990	0.088022349616	-2.042987752372

[m3, Me] complex in the ^{1}B1 state

E_{tot} = -2466.46546907684
E_{zpve} = 0.607447350556
E_{SP} = -2466.81591117

Ni	0.004200947370	0.058816195241	-0.351522751992
P	1.598961621438	1.415938500521	0.332762030357
P	-1.615588530146	1.432157898706	0.214275193350
P	1.600851455539	-1.448003295006	-0.205164582401
P	-1.619532395735	-1.423023513025	-0.169686280171
C	1.262024721427	3.204599698785	0.638451874043
H	1.109048783021	3.312012035636	1.718548497535
C	-1.275687369671	3.240263061556	0.456476132623
H	-1.293039732637	3.420739515081	1.537153097185
C	-1.291366219556	-3.203676807569	-0.527946573509
H	-1.253363418187	-3.298952599118	-1.618965228051
C	1.260134862149	-3.219176337824	-0.578674765656
H	2.146380078979	-3.784997775026	-0.273240502296
C	-3.059746546739	1.382890583732	-0.970111896835
H	-3.982577376251	1.432502905799	-0.364744463467
H	-2.991545404901	2.299762546869	-1.562362349343
C	-3.118076022642	-1.050863745808	-1.223216959762

H	-4.012110528722	-1.156781906770	-0.585693265787
H	-3.151137004440	-1.828826505740	-1.991137731507
C	2.931681890173	1.451052028923	-0.986937887088
H	2.847597823807	2.395858094209	-1.525646817538
H	3.922555873776	1.361290961608	-0.537809888595
C	2.959442440396	-1.015907228203	-1.419475829388
H	3.939906679792	-1.055085641068	-0.942382030938
H	2.914712826720	-1.725320068161	-2.247172098839
C	2.522010214331	-1.500246291527	1.411591739805
H	3.186936689731	-2.368169158279	1.368924283169
H	1.783031019639	-1.669205211270	2.213608127149
C	2.557938417191	0.910029363832	1.841463358092
H	3.246316620339	1.730116313323	2.070347162649
H	1.836302585890	0.823363270877	2.672913073996
C	-2.370932290013	0.983340905000	1.857324385472
H	-3.056283183207	1.795566861912	2.121437226909
H	-1.546914312616	0.979869243308	2.594244681831
C	-2.319355410425	-1.450533042451	1.556201481144
H	-2.965782871303	-2.331041264981	1.628018524925
H	-1.480817218182	-1.578893728137	2.262848195311
N	2.753782136431	0.353933100774	-1.990534067338
N	-3.036752882885	0.247375806610	-1.888084760147
N	3.334608635015	-0.303111827866	1.611849781666
N	-3.125947596832	-0.262678128532	1.823894540640
C	-4.095642807665	0.381295849031	-2.892502522458
H	-3.975093138599	1.329345915015	-3.425408236978
H	-4.016170406938	-0.434361384614	-3.617389836325
H	-5.108090866819	0.357271422605	-2.452039029682
C	3.582740492111	0.580630762666	-3.212285543867
H	3.350970161066	-0.203053992767	-3.934229220160
H	3.327511420068	1.558066041454	-3.623022372973
H	4.637904526129	0.546019195964	-2.937794711309
C	4.327224067468	-0.507693353676	2.671140041995
H	4.949845969651	-1.372354244626	2.422823370754
H	4.971446465297	0.373806367833	2.738707801687
H	3.865960481183	-0.680318910322	3.658350643933
C	-3.918938287347	-0.428428645736	3.044251439902
H	-4.574284141158	0.438183844431	3.173367279086
H	-4.543181866467	-1.322667491179	2.955942045288
H	-3.292624676173	-0.529093528541	3.948042528813
C	-0.006803033869	-3.768137965976	0.093075400462
H	-0.013963787155	-4.854831859356	-0.042744432727
H	1.169892255935	-3.297544718563	-1.667576338035
H	0.015120076691	-3.589583709821	1.174583026619
H	-2.169996938265	-3.755538712427	-0.176707717637
C	0.048372330672	3.750390641558	-0.124506651423
H	0.056435261202	4.842293163661	-0.040753310825
H	-2.125064011996	3.777691129533	0.023543645410
H	0.122079175786	3.514412784459	-1.192732456335
H	2.176069065074	3.749980481896	0.380996220407
H	0.194681789330	0.337144714540	-2.042825077215
H	1.716731256651	0.389265832746	-2.252487322744

[m3, Me] complex in the ^1TS_C state

E_{tot} = -2466.46266048054
E_{zpve} = 0.602580930367
E_{SP} = -2466.81310175

Ni	0.006342839388	-0.035736568521	0.208827811988
P	1.620754065916	-1.408598156908	-0.396586721846
P	-1.618779219721	-1.422072428569	-0.313350241731
P	1.626343990055	1.462522528884	0.198229346640
P	-1.627873837420	1.441632737655	0.151630588507
C	1.273687338895	-3.178826069320	-0.769349804828
H	1.130060387840	-3.252961737885	-1.853409449161
C	-1.270121627657	-3.218467268653	-0.609390476032
H	-1.277375245770	-3.365176279731	-1.695455967568
C	-1.286080115965	3.215973215107	0.523583016836
H	-1.239182236483	3.302286225842	1.614835150633
C	1.268358558510	3.230673532579	0.574336892217
H	2.150922282339	3.806440142030	0.277135588790
C	-3.004576940670	-1.405595622357	0.938157297662
H	-3.955752057877	-1.438673320104	0.377378040678
H	-2.909094904577	-2.338297175086	1.501310632316
C	-3.066358321327	1.022528897308	1.265887132622
H	-3.994754561173	1.137306798164	0.681263061047
H	-3.066219742821	1.775957033411	2.058613597267
C	2.837229382560	-1.479997721398	1.031492005444
H	2.689639121718	-2.437635715562	1.535424904906
H	3.860439695291	-1.418246946519	0.650281210298
C	2.875124312088	0.954592315432	1.506056671337
H	3.885381939388	0.997248429829	1.090368482287
H	2.788115755395	1.655796347436	2.337642963919
C	2.655081430369	1.526693792106	-1.349769127430
H	3.333480851876	2.377748422800	-1.236744395168
H	1.979667107685	1.735683040057	-2.197261021782
C	2.673267115372	-0.875223182336	-1.828496797844
H	3.361223451857	-1.701162398329	-2.036902271356
H	2.006381406512	-0.756829488023	-2.700752557505
C	-2.439426639016	-0.932608905727	-1.910791989203
H	-3.124851462582	-1.745066728823	-2.172762648226
H	-1.645145222810	-0.894822440918	-2.678455277643
C	-2.392950984806	1.489672440511	-1.545558509661
H	-3.044782574241	2.368218209761	-1.575511791008
H	-1.582779449328	1.634821076389	-2.281151097824
N	2.579370762301	-0.405144410650	2.017703041931
N	-2.933773195374	-0.295151202770	1.885387368732
N	3.454803738745	0.317649157408	-1.523917580424
N	-3.203742490342	0.302807309950	-1.802438599594
C	-3.929511587142	-0.467965127395	2.947501179609
H	-3.767652606107	-1.428394481125	3.446022746112
H	-3.814138703035	0.329055588728	3.688127037555
H	-4.966543931940	-0.443516927929	2.569038070146
C	3.213310287545	-0.673011010787	3.328332932554
H	2.902163443111	0.102709131074	4.030811764127
H	2.871909308228	-1.645839373316	3.687572328453
H	4.303062507933	-0.673481522625	3.234598387420
C	4.514936261027	0.523649287802	-2.515517613395
H	5.134633157232	1.373140207847	-2.213437306327
H	5.148276451671	-0.367228670689	-2.559784837725
H	4.118870708690	0.721818751929	-3.526067145757
C	-4.064447416114	0.494109335048	-2.972713424860
H	-4.718944948764	-0.374893595701	-3.088851640672
H	-4.689883599790	1.379447453610	-2.824662767102
H	-3.490032844322	0.625153171506	-3.906347441211
C	0.000188246902	3.774589638100	-0.101129161120
H	-0.007292303361	4.862077981047	0.027991139962
H	1.171872031693	3.306719563894	1.662835374387
H	0.022086890417	3.589736308819	-1.181650984771
H	-2.162177336939	3.778375165950	0.182948856035
C	0.051212586923	-3.741100990053	-0.031290969577
H	0.059672065909	-4.830705840716	-0.139929188589
H	-2.121253993920	-3.772670881114	-0.201096153911
H	0.116663125684	-3.531062366371	1.043091780612
H	2.182688593585	-3.735888674066	-0.518100445795
H	0.245435073203	-0.377367783191	2.002664280390
H	1.327220827527	-0.412194224747	2.127487714227

[m3, Me] complex in the ^1C state

E_{tot} = -2466.48559106471
E_{zpve} = 0.600532916241
E_{SP} = -2466.83804304

Ni	-0.001104318436	-0.015839357008	-0.092144628823
P	-1.644357583938	-1.444646050945	0.287722981164
P	1.645325454191	-1.444816489689	0.287070403952
P	-1.653119752609	1.458079443120	-0.198169541767
P	1.652778851921	1.460140261327	-0.193868725617
C	-1.279120137647	-3.230131627595	0.588024917542
H	-1.205965873559	-3.360701034444	1.673743401903
C	1.278716935330	-3.230868736567	0.585593580277
H	1.206887424089	-3.361609057566	1.671429648548
C	1.281873630300	3.234837053004	-0.530523825325
H	1.215370904292	3.339231994343	-1.619036918993
C	-1.281393206091	3.228472048161	-0.549154113216
H	-2.158679418629	3.803680267464	-0.234239313089
C	2.927465999460	-1.426738810140	-1.067060251209
H	3.920297229746	-1.457203622810	-0.584828546106
H	2.789517391523	-2.360907483136	-1.618758545053
C	2.987989227096	1.002846953372	-1.413146157416
H	3.962832393096	1.101850658580	-0.907186868086
H	2.940044866433	1.749752207143	-2.210283847315
C	-2.938067133646	-1.429346301108	-1.055219871523
H	-2.805394687261	-2.364621005332	-1.606909743832
H	-3.926359800412	-1.459072707052	-0.563685526084
C	-3.002320308862	0.998152514451	-1.401039990567
H	-3.970125553903	1.093210472170	-0.880974577348
H	-2.967013295148	1.745899829760	-2.197933479418
C	-2.504954879959	1.482161273123	1.458826251939
H	-3.163125624008	2.355970798498	1.461896264960
H	-1.735468153576	1.627546543539	2.236730438598
C	-2.547852223660	-0.941542515989	1.835326062664
H	-3.237347102413	-1.757611035905	2.073549311722
H	-1.798114530948	-0.882399218774	2.644852960485
C	2.565074696156	-0.947051185052	1.826527720317
H	3.253693751437	-1.766265402584	2.055836575941
H	1.823556424609	-0.884984962459	2.643186708930
C	2.522839875763	1.475301911397	1.454087695965
H	3.182321598763	2.348110988414	1.451240390436
H	1.763838202635	1.619805997459	2.242196890862
N	-2.794291196166	-0.321233690149	-1.997858310623
N	2.773888707778	-0.317143944434	-2.006620104068
N	-3.316453879256	0.284829096733	1.660950492215
N	3.335255557570	0.276603921250	1.642860246875
C	3.645028658029	-0.508455979308	-3.170528046484
H	3.413531013408	-1.467305550737	-3.643838358307
H	3.461270625339	0.288593021669	-3.897012567117
H	4.717232533497	-0.499787496413	-2.908240964352
C	-3.681762379460	-0.513377455512	-3.149515635463
H	-3.507741233466	0.282811579325	-3.879226295973
H	-3.457351864559	-1.472940533150	-3.624755377732
H	-4.750172020297	-0.504055101524	-2.872065160658

C	-4.266406835276	0.472006083461	2.762184356940
H	-4.886687747291	1.350579973266	2.562173795102
H	-4.919827393832	-0.402443689579	2.831357699575
H	-3.765913572843	0.611828570890	3.735854780666
C	4.302136341887	0.460621204005	2.729846645177
H	4.951354471027	-0.417535755228	2.790871588977
H	4.924381024444	1.335194471953	2.518854077061
H	3.816988862553	0.605139969153	3.710518300374
C	-0.005777951938	3.760351469667	0.124255188030
H	-0.007290965043	4.851036755977	0.026999514840
H	-1.196000228350	3.320856917799	-1.637483065083
H	-0.013488380494	3.545288397708	1.199436162460
H	2.152205950467	3.808578429236	-0.194452374075
C	-0.001076362103	-3.748212737134	-0.088915254142
H	-0.001054669639	-4.840869179879	-0.016581768575
H	2.156013916894	-3.791438770903	0.245990651052
H	-0.002504675595	-3.505302319493	-1.158857437682
H	-2.157504751966	-3.790090586602	0.250337634901
H	0.385511536277	-0.496115315650	-2.766681281832
H	-0.365767169573	-0.501229485535	-2.775674320115

[m3, Me] complex in the ^{1}P state

E_{tot} = -2465.30351636275
E_{zpve} = 0.587606783704
E_{SP} = -2465.65508938

Ni	0.00064306	0.04434304	0.19011664
P	-1.64380053	-1.40984931	0.44927892
P	1.64557782	-1.41280180	0.43029697
P	-1.66289703	1.49585407	-0.03986481
P	1.66385690	1.49252528	-0.05926652
C	-1.27936538	-3.17836029	0.81801500
H	-1.20805055	-3.26595811	1.90833636
C	1.28284146	-3.18153578	0.80068083
H	1.22645336	-3.27091852	1.89172247
C	1.28201668	3.28071953	-0.32237268
H	1.18961742	3.42957896	-1.40375489
C	-1.28220293	3.28486393	-0.30183055
H	-2.14845836	3.85217683	0.05407423
C	2.68960045	-1.43966764	-1.11152938
H	3.74938353	-1.49736751	-0.80855536
H	2.43121714	-2.36970433	-1.62620277
C	2.77778225	0.98618466	-1.46675909
H	3.82286822	1.02852018	-1.11870453
H	2.64528755	1.73721183	-2.24954285
C	-2.69566535	-1.43602251	-1.08738314
H	-2.44073411	-2.36632209	-1.60330528
H	-3.75406763	-1.49253992	-0.77965749
C	-2.78326225	0.99026361	-1.44300681
H	-3.82697325	1.03374496	-1.09105263
H	-2.65296219	1.74077990	-2.22665192
C	-2.76745511	1.48341245	1.45995603
H	-3.44729233	2.33319738	1.34913448
H	-2.14119735	1.66075228	2.35184819
C	-2.78542631	-0.93656145	1.83650902
H	-3.47247532	-1.77805022	1.97034367
H	-2.17242388	-0.84884673	2.75150931
C	2.79359381	-0.93948138	1.81244501
H	3.48115819	-1.78093820	1.94374886
H	2.18386538	-0.85181711	2.72966544
C	2.77457872	1.48091802	1.43602105
H	3.45411259	2.33059399	1.32249458
H	2.15183610	1.65834559	2.33031389
N	-2.41485729	-0.31878213	-1.99395828
N	2.40611792	-0.32220247	-2.01674105
N	-3.56043596	0.26047616	1.53670846
N	3.56760056	0.25768363	1.51023194
C	3.07519951	-0.53923978	-3.30575255
H	2.74006018	-1.48845885	-3.73357108
H	2.80523629	0.26529936	-3.99573852
H	4.17467540	-0.56684183	-3.21440804
C	-3.08814452	-0.53566010	-3.28077055
H	-2.81993698	0.26863559	-3.97173661
H	-2.75473929	-1.48506995	-3.70953134
H	-4.18733888	-0.56275866	-3.18605927
C	-4.67849959	0.41312492	2.47291732
H	-5.29222252	1.26685676	2.17101968
H	-5.30132748	-0.48570463	2.44506578
H	-4.34363082	0.57376972	3.51213762
C	4.68769072	0.41033543	2.44411065
H	5.31046891	-0.48848768	2.41508711
H	5.30076277	1.26409900	2.14104331
H	4.35483959	0.57095494	3.48400436
C	0.00620812	3.77157596	0.38197429
H	0.00752932	4.86615369	0.34908197
H	-1.20824544	3.43839594	-1.38392587
H	0.01410174	3.49321609	1.44282150
H	2.15535499	3.84782727	0.01620926
C	-0.00329316	-3.72156612	0.15734311
H	-0.00384998	-4.81088134	0.26813436
H	2.15719620	-3.75176782	0.46904902
H	-0.01028810	-3.51794429	-0.92089665
H	-2.15929017	-3.74746074	0.49929790

NEt_3

E_{tot} = -292.453083966569
E_{zpve} = 0.205638853744
E_{SP} = -292.530484904

N	-0.925957250527	2.196027130153	0.007905523382
C	-0.737304198576	2.850724946309	-1.296399005403
C	-1.781164814498	3.933272593230	-1.573162279791
H	-0.819516461849	2.087446298113	-2.076079835636
H	0.281804053168	3.273201538898	-1.385627460341
H	-1.623542921502	4.370086503298	-2.565757118892
H	-2.789706939073	3.507017674003	-1.539030266223
H	-1.735423036983	4.748438895882	-0.843394622555
C	-0.418746459297	3.028310218589	1.110509045632
C	-1.094673132159	2.714420281977	2.445557364480
H	-0.613208816430	4.076697371115	0.865266187648
H	0.679592930519	2.933755939674	1.207656113021
H	-0.710166443183	3.372961679949	3.232585686911
H	-2.177098097683	2.863374742782	2.367444038200
H	-0.919398936583	1.681524249095	2.764697150253
C	-0.327225998223	0.851481599877	0.026368317643
C	-1.206895806354	-0.196583402616	-0.655675545629
H	-0.184196692818	0.554309878665	1.069606375639
H	0.680141152920	0.859457074094	-0.431699973084
H	-0.736128224805	-1.184639347844	-0.600553189618
H	-2.184274049439	-0.251176002788	-0.164081594379
H	-1.374921046626	0.030405828544	-1.713793321256

H^+NEt_3

E_{tot} = -292.938245275184
E_{zpve} = 0.221720218906
E_{SP} = -293.008633694

N	-0.682528247874	2.242887511183	-0.068656028441
C	-0.470048484936	2.722120700301	-1.491681992492
C	-1.442642285004	2.077885291994	-2.466555291687
H	0.572609680033	2.501197178729	-1.731197493460
H	-0.601426134914	3.803868551664	-1.486163477285
H	-1.252892831427	2.483335965477	-3.464583101112
H	-1.328214583884	0.991974348318	-2.518617972031
H	-2.4780860336640	2.310765279096	-2.196476502775
C	-0.147060236122	3.236230360611	0.945040688270
C	-1.060878637842	4.439795462274	1.114480368805
H	0.850347268961	3.513576147501	0.596476547690
H	-0.044871897964	2.701842368566	1.889600282710
H	-0.634245822834	5.091910220166	1.882143662968
H	-1.161222039307	5.025198292175	0.196572051212
H	-2.057609392075	4.131001761354	1.446924997924
C	-0.104604627630	0.856865191127	0.146701596176
C	-0.772218812006	0.127657867057	1.302061039597
H	0.967330509448	0.998102265566	0.302345454021
H	-0.248444802244	0.304329949152	-0.781743806504
H	-0.327370793246	-0.867926379619	1.387729330142
H	-0.635887465966	0.639426630843	2.258453904663
H	-1.844886606693	0.005706087476	1.118111307544
H	-1.696307022812	2.173852108987	0.081495534061

[DMF(H)][OTf]

E_{tot} = -1210.58418368374
E_{zpve} = 0.143772780046
E_{SP} = -1210.93209254

C	0.088300624075	0.584350947802	-0.367961577242
O	0.926213630411	0.131141528456	0.502779932272
H	0.007345131535	0.101120191910	-1.340977664475
N	-0.660727478219	1.623017759194	-0.124213578270
C	-0.611555001705	2.354582999531	1.145688866625
H	-0.330287229207	3.391420234014	0.943228404409
H	-1.604965231445	2.333882992506	1.601272159907
H	0.113783231979	1.897801177321	1.815628326535
C	-1.602798978064	2.123715919316	-1.129884157810
H	-1.343588031656	3.156245192305	-1.378036408269
H	-1.555436278407	1.506868264863	-2.027388127940
H	-2.614168538761	2.095056612006	-0.716222731693
H	1.430740641137	-0.696239784559	0.120575922169
S	2.529422625870	-1.756410904391	-1.886154466341
O	2.795591125386	-3.058321350702	-2.511998573625
O	2.090561507366	-1.863902928830	-0.442004952048
O	1.735998914692	-0.775309939233	-2.655221286603
C	4.192751089272	-0.950728648431	-1.716798540196
F	5.028833372376	-1.731464098028	-1.019250874810
F	4.067071613882	0.221927863966	-1.075300468145
F	4.726249528724	-0.723042747370	-2.925206673841

[DMF][OTf]$^{-}$

E_{tot} = -1210.11310741876
E_{zpve} = 0.1307898371
E_{SP} = -1210.47672933

C	0.844272273539	1.767093474589	0.900774228454
O	0.697392561334	1.587775082106	2.110393697267
H	1.837794746482	1.846365015470	0.428124897938
N	-0.159809515685	1.889063671263	-0.003022808886
C	-1.549349638225	1.772554893787	0.404927190629
H	-2.090596182481	2.706545874854	0.208203179214
H	-2.042371592450	0.965847038889	-0.150966563135
H	-1.585986251952	1.552754763020	1.472234408924
C	0.096452843587	2.170601743022	-1.406826945691
H	-0.357015617249	3.128570557318	-1.691871997568
H	1.172001935360	2.206435768804	-1.584933584941
H	-0.315161187737	1.374909827802	-2.035530032178
S	2.096469468516	-0.841673249572	-1.351965976150
O	0.881501405532	-0.918653411875	-2.197673602473
O	1.926356688113	-1.296475045124	0.047224366858
O	2.910324307940	0.387568309669	-1.521457347233
C	3.190330198792	-2.152772885256	-2.089127664201
F	2.607316336820	-3.365205586351	-2.020593695898
F	4.368668350993	-2.225413076352	-1.440665491949
F	3.450004498764	-1.893941696053	-3.385268658993

References

1. M. Dolg, U. Wedig, H. Stoll and H. Preuss, Energy-adjusted ab initio pseudopotentials for the first row transition elements, *J. Chem. Phys.*, 1987, **86**, 866-872.

2. S. Grimme, J. Antony, S. Ehrlich and H. Krieg, A consistent and accurate ab initio parametrization of density functional dispersion correction (DFT-D) for the 94 elements H-Pu, *J. Chem. Phys.*, 2010, **132**, 154104.

3. S. Chen, S. Raugei, R. Rousseau, M. Dupuis and R. M. Bullock, Homogeneous Ni Catalysts for H2 Oxidation and Production: An Assessment of Theoretical Methods, from Density Functional Theory to Post Hartree−Fock Correlated Wave-Function Theory, *J Phys Chem A*, 2010, **114**, 12716-12724.

4. S. Raugei, S. Chen, M.-H. Ho, B. Ginovska-Pangovska, R. J. Rousseau, M. Dupuis, D. L. DuBois and R. M. Bullock, The Role of Pendant Amines in the Breaking and Forming of Molecular Hydrogen Catalyzed by Nickel Complexes, *Chem. Eur. J.*, 2012, **18**, 6493-6506.

5. S. Maeda, K. Ohno and K. Morokuma, Systematic exploration of the mechanism of chemical reactions: the global reaction route mapping (GRRM) strategy using the ADDF and AFIR methods, *Phys. Chem. Chem. Phys.*, 2013, **15**, 3683-3701.

6. S. Maeda, T. Taketsugu, K. Morokuma and K. Ohno, Anharmonic Downward Distortion Following for Automated Exploration of Quantum Chemical Potential Energy Surfaces, *B Chem Soc Jpn*, 2014, **87**, 1315-1334.

7. S. Chiodo and N. Russo, Determination of spin-orbit coupling contributions in the framework of density functional theory, *J. Comput. Chem.*, 2008, **29**, 912-920.

8. S. G. Chiodo and M. Leopoldini, MolSOC: A spin–orbit coupling code, *Computer Physics Communications*, 2014, **185**, 676-683.
